# *Euphorbiambuinzauensis*, a new succulent species in Kenya from the *Synadenium* group in Euphorbiasect.Monadenium (Euphorbiaceae)

**DOI:** 10.3897/phytokeys.183.70285

**Published:** 2021-10-11

**Authors:** Neng Wei, Fredrick Munyao Mutie, Geoffrey Mwachala, Olwen M. Grace, Guang-Wan Hu, Qing-Feng Wang

**Affiliations:** 1 Key Laboratory of Plant Germplasm Enhancement and Specialty Agriculture, Wuhan Botanical Garden, Chinese Academy of Sciences, Wuhan, CN-430074, China Royal Botanic Gardens Richmond United Kingdom; 2 Royal Botanic Gardens, Kew, Richmond, Surrey, TW9 3AE, UK Wuhan Botanical Garden, Chinese Academy of Sciences Wuhan China; 3 University of Chinese Academy of Sciences, Beijing, CN-100049, China University of Chinese Academy of Sciences Beijing China; 4 East African Herbarium, National Museums of Kenya, P.O. Box 451660-0100, Nairobi, Kenya National Museums of Kenya Nairobi Kenya; 5 Center of Conservation Biology, Core Botanical Gardens, Chinese Academy of Sciences, Wuhan, CN-430074, China Core Botanical Gardens, Chinese Academy of Sciences Wuhan China; 6 Sino-Africa Joint Research Center, Chinese Academy of Sciences, Wuhan, CN-430074, China Sino-Africa Joint Research Center, Chinese Academy of Sciences Wuhan China

**Keywords:** East Africa, endangered, molecular phylogeny, succulents, *Synadenium*, taxonomy

## Abstract

*Euphorbiambuinzauensis*, a succulent new species of the *Synadenium* group in Euphorbiaceae from Makueni County, Kenya, is described and illustrated. Morphologically, it is most similar to *E.pseudomollis*, but differs mainly by its shrubby habit (up to 4 m), abaxial leaves surfaces with densely stellate hairs, 2–4-forked cymes, smaller bracts (ca. 2.5 × 3.0 mm), smaller cyathia (6 mm wide), crimson glands without narrow smooth margin, smaller fruits (ca. 8 × 7 mm) and ovoid seeds (ca. 1.8 × 2.2 mm). Furthermore, we performed a molecular phylogenetic analysis of the *Synadenium* group in Euphorbiasect.Monadenium, based on complete nuclear ribosomal DNA (nrDNA) datasets. This phylogenetic inference also supports it to be a distinct species. The new species is assessed as Endangered using the IUCN criteria.

## Introduction

*Synadenium* Boiss. (in de Candolle (1862: 187)), was a previously recognized genus of Euphorbiaceae, confined to east and southern tropical Africa, with 14 closely related species (Carter and Leach 2001). *Synadenium* is readily distinguishable from other genera in Euphorbieae subtribe Euphorbiinae by the unique ring-shaped fused glands in the cyathium (Jones and Smith 1969; Carter 1988; Carter and Leach 2001). It has been regarded as a taxonomically difficult genus (Govaerts et al. 2000), since the differences between the species appear to be slight, especially when observing dried herbarium specimens only. Moreover, Brown et al. (1909) assumed that *Synadenium* species are locally endemic and probably more numerous than previously known (13 spp. at that time), given their great resemblance to one another, which may have resulted in some of them being overlooked.

Besides emphasizing leaf and cyme-branching characters, Carter and Leach (2001) suggested that emphasis should also be placed upon the habit, the shape and size of the cyathium, the color and furrowing of the involucral glands, and features of the capsules and seeds. Unfortunately, relatively few specimens have been prepared and deposited in herbaria, especially fruiting ones (Carter 1988). Although regional taxonomic treatments have been done, *Synadenium* has never been comprehensively monographed, and was regarded as a poorly known group.

Webster (1967) and Croizat (1972) questioned the delimitation of *Synadenium* and *Monadenium* as distinct genera and suggested, based on morphological similarities, that they were closely related to Euphorbiasect.Tirucalli. Analysis of molecular data over the past two decades (Steinmann and Porter 2002; Bruyns et al. 2006, 2011; Zimmermann et al. 2010; Horn et al. 2012; Dorsey et al. 2013; Wei et al. 2021), have all shown that the genera *Endadenium*, *Monadenium*, and *Synadenium* were deeply nested in *Euphorbia*. Consequently, they were merged under *Euphorbia* and treated as subgenus Euphorbiasect.Monadenium, to maintain the monophyly of *Euphorbia* (Bruyns et al. 2006). However, the *Synadenium* group has been poorly sampled to date (e.g., three species in Dorsey et al. 2013).

In a field investigation targeting Kenyan *Euphorbia* in 2018, a shrubby Euphorbia (Synadenium) species attracted the authors’ attention. At first sight, it was roughly identified as *E.pseudomollis* Bruyns in Bruyns et al. (2006: 414), due to its densely pubescent leaves. More specimens of this *Euphorbia* were collected during the following field trip at the same area, and then detailed morphological studies were conducted. Based on the floral records and the examination of *Synadenium* specimens deposited in herbaria worldwide, we found that this *Euphorbia* differs from *E.pseudomollis* by a combination of several morphological characters. Furthermore, the molecular phylogeny of the *Synadenium* group based on complete sequences of the nuclear ribosomal DNA (nrDNA) revealed that our *Euphorbia* collection is sister to E.bicompactaBruynsvar.bicompacta Bruyns in Bruyns et al. (2006: 412). Hence, both morphological studies and phylogenetic analyses indicate that our *Euphorbia* collections represent a hitherto undescribed species, which we now proceed to describe and name as *Euphorbiambuinzauensis*.

## Materials and methods

### Morphological observations

The morphological description of the new species is based on measurements on living specimens collected during the field trips, except for the seeds which were obtained by observations of specimens in herbaria. Comparisons with other relevant *Euphorbia* species were based on previous regional floristic accounts (Brown et al. 1909; Carter 1988; Carter and Leach 2001) as well as the examination of herbarium specimens and digitized images which are mainly from AMD, BR, BM, EA, HIB, K, P, S, and WAG (herbarium acronyms following Thiers (2021)).

### Sampling, genomic DNA extraction, and sequencing

To delineate the phylogenetic placement of our *Euphorbia* collection, a total of 17 sequences, which were all newly generated in this study, were used to infer the phylogenetic tree for the *Synadenium* group in *Euphorbia*. Amongst these sequences, 14 accessions representing nine accepted species of the *Synadenium*-group were included. The remaining three accessions from traditionally recognized *Euphorbia*, *Monadenium* and *Endadenium*, were treated as outgroups, according to Dorsey et al. (2013) and Wei et al. (2021).

Sources of DNA were silica-dried leaves collected from field trips, or from dried voucher specimens in herbaria. Total genomic DNA was extracted with the MagicMag Genomic DNA Micro Kit (Sangon Biotech Co., Shanghai, China) following the instructions of the manufacturer’s protocol. DNA quality was assessed by 1% agarose gel electrophoresis. Short inserts (350 bp) were used to construct paired-end 150-bp sequencing libraries using the NEBNext Ultra DNA Library Prep Kit for Illumina (NEB, United States). Libraries were sequenced at Beijing Genomics Institute (Shenzhen, China) using the Illumina HiSeq 2000 Platform (Illumina, San Diego, CA, United States).

### Nuclear ribosomal DNA (nrDNA) assembly and annotation

Raw sequences were quality filtered using software Trimmomatic v.0.33 (Bolger et al. 2014), to avoid any potential sequencing artefacts, improve uniformity in the read length (> 50 bp) and warrant quality (Phred score > 30) in the following assemblies. FastQC 0.11.8 (http://www.bioinformatics.babraham.ac.uk/projects/fastqc/) was used to assess the trimming quality. The remaining high-quality trimmed sequences were then *de novo* assembled in GetOrganelle (Jin et al. 2020). The produced scaffolds were viewed and then exported as the complete nrDNA in Bandage v.0.7.1 (Wick et al. 2015). The derived nrDNA sequences were annotated in Geneious v.8.0.2 (Kearse et al. 2012) against the annotated nrDNA from other members of Malpighiales in GenBank (*Linumusitatissimum*EU307117; *Hirtellaphysophora*KJ414478) as references. The annotated accessions were prepared with GB2sequin (Lehwark and Greiner 2019) for GenBank submission. The complete nrDNA repeat sequence, including its seven constituent loci, i.e., external transcribed spacer (ETS), 18S, internal transcribed spacer 1 (ITS1), 5.8S, internal transcribed spacer 2 (ITS2), 26S, and intergenic spacer (IGS), was used to perform phylogenetic analyses. The sampled species, voucher information, and GenBank accession numbers are provided in Suppl. material 1.

### Molecular phylogenetic analyses

The complete nrDNA sequences were aligned by MAFFT v. 7 (Katoh and Standley 2013) with the default setting. TrimAl v.1.2 (Capella-Gutierrez et al. 2009) was used to trim the alignment sequence with automatd1 mode to reduce potentially poorly aligned regions. Besides, the trimmed alignments were also visually inspected in Geneious 8.0.2 (Kearse et al. 2012) and manually adjusted if necessary. PartitionFinder 2 (Lanfear et al. 2012, 2016) was used for best-fit substitution model selection for each region under the Akaike Information Criterion: the general time reversible model with a gamma distribution of substitution rates (GTR+G) was chosen for the ETS region; the GTR+I+G model with a proportion of invariant sites was selected for the 5.8S and 28S regions; the Hasegawa-Kishino-Yano model with a proportion of invariant sites (HKY+I) was selected for the ITS1, ITS2, and IGS regions; and the HKY+I+G model with a gamma distribution of substitution rates was selected for the 18S region. The ML tree was inferred by IQ-TREE v.1.6.8 (Nguyen et al. 2015) with 10,000 bootstrap replicates. The BI phylogenetic analysis was performed with MrBayes v.3.2.7 (Ronquist et al. 2012). Two independent Markov Chain Monte Carlo analyses (MCMC) were run with four simultaneous chains of 10 million generations, sampling one tree every 100 generations, and setting the burnin fraction as 0.25. The remaining trees were then used to construct a majority-rule consensus tree. The average deviation of split frequencies was verified by reaching a value below 0.01 at the end of the MCMC analyses. The effective sample sizes (ESS values > 200) for all parameters and statistics were also assessed using Tracer v.1.7.1 (Rambaut et al. 2018). The final phylogenetic tree was shown using the online tool iTOL (Letunic and Bork 2007).

## Results

### Phylogenetic relationships

**Figure 1.**
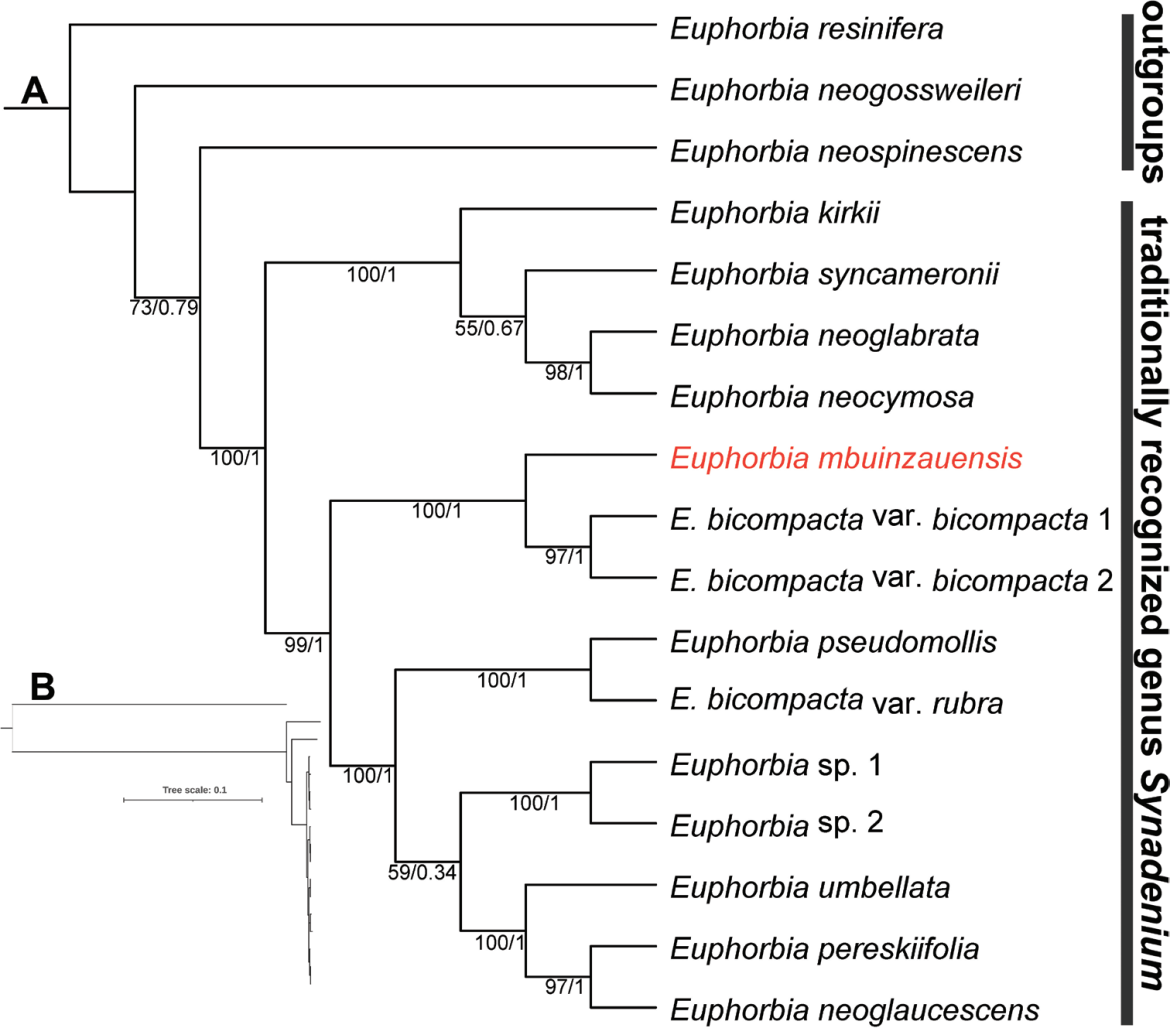
**A** The maximum likelihood tree inferred from the complete nuclear ribosomal DNA sequences to elucidate the phylogenetic position of *Euphorbiambuinzauensis*. Bootstrap and Bayesian posterior probability values are shown below the branches (BS/PP). The new species is highlighted in red **B** same tree as A showing branch lengths proportional to nucleotide substitutions per site.

The 17 complete nrDNA repeat sequences have average coverage ranging from 430.5 to 524.9 (Suppl. material 1). The aligned length of the seven concatenated nrDNA constituent loci dataset prior to trimming is 11,671 bp, whereas the trimmed alignment dataset consisted of 10,605 bp (Suppl. material 2) with 293 parsimony-informative sites. The ML and BI trees are identical, and the ML tree with both posterior probabilities and ML bootstrap values for each clade is shown as Figure 1. For the ML analysis of the combined seven loci of nrDNA, likelihood score (-ln*L*) is 23609.7. Overall, the 14 accessions of the *Synadenium* group clustered together and formed a strongly supported monophyletic group (BS = 100%, PP = 1). The new species, *Euphorbiambuinzauensis*, is sister to the clade that consists of two accessions of E.bicompactavar.bicompacta with robust support (BS = 100%, PP = 1). Despite the new species being morphologically closest to *E.pseudomollis*, they were not sister taxa in our phylogenetic tree. In addition, the accession of E.bicompactavar.rubra did not form a clade with E.bicompactavar.bicompacta as expected. Instead, it is sister to another species, *E.pseudomollis*, with robust support (BS = 100%, PP = 1).

### Morphological comparisons

Morphologically, *Euphorbiambuinzauensis* is closest to *E.pseudomollis*, a species that occurs in Kenya and Tanzania, but differs by a shorter habit (up to 4 m), abaxial surfaces of leaves densely covered with stellate hairs, 2–4-forked cymes, smaller bracts (ca. 2.5 × 3.0 mm) with dentate margin, smaller cyathia (6 mm wide), crimson gland without narrow smooth margin, smaller fruits (ca. 8 × 7 mm) and smaller ovoid seeds (ca. 1.8 × 2.2 mm). Based on the results of our phylogenetic analyses, the sister taxon of *Euphorbiambuinzauensis* is E.bicompactavar.bicompacta. However, E.bicompactavar.bicompacta can be easily distinguished from *E.mbuinzauensis* by its glabrous leaves, shallowly and minutely grooved yellow to greenish red gland, half fused style, and larger seeds (ca. 2.8 × 2.5 mm). Further detailed morphological differences among the three species are presented in Table 1.

**Table 1.** Characters distinguishing *Euphorbiambuinzauensis* from *E.pseudomollis* and E.bicompactavar.bicompacta.

Character	*E.mbuinzauensis*	*E.pseudomollis*	E.bicompactavar.bicompacta
Habit	Shrub to 4 m	Shrubby tree to 9 m	Shrubby tree to 7 m
Leaf apex and margin	Apex rounded to obtuse, margin slightly undulate, crenate	Apex rounded to obtuse, margin serrate	Apex abruptly acute, margin entire, or minutely toothed
Lamina surface	Densely pubescent on adaxial surface, abaxial surfaces with densely stellate hairs, occasionally tinged purplish	Densely pubescent on both sides, abaxial surfaces without flecks	Glabrous, abaxial surfaces flecked or tinged purplish
Inflorescence	In pseudo-umbels of 2–5 cymes; cymes 2–4-forked	In pseudo-umbels of 3–5 cymes; cymes 1–2-forked	In pseudo-umbels of up to 6 cymes; cymes 2–3-forked
Bract	Ca. 2.5 × 3.0 mm, dentate	Ca. 3.5 × 3.5 mm, entire or with a few teeth	Ca. 3.0 × 3.5 mm, obscurely dentate
Cyathium	Ca. 6 mm wide	Ca. 8 mm wide	Ca. 7 mm wide
Gland	Deeply furrowed and wrinkled, crimson	Distinctly grooved, with a very narrow smooth margin, crimson to light red	Shallowly and minutely grooved, yellow to greenish red
Female flower	Styles ca. 2.0 mm long, connate at the base	Styles ca. 1.5 mm. long, connate at the base	Styles ca. 1.8 mm. long, connate to ± halfway
Capsule	To 8 × 7 mm	To 10 × 10 mm	To 8 × 7 mm
Seed	Ovoid, ca. 1.8 × 2.2 mm	Subglobose, ca. 2.5 × 2.5 mm	Ovoid, ca. 2.8 × 2.5 mm

## Discussion

*Euphorbiambuinzauensis* belongs to the Synadenium group of subg. Euphorbia, which is characterized by tree-like or shrubby habit, fleshy leaves with prominent midrib, pseudo-umbels of 2–5 cymes on peduncles, cymes that are forked several times, involucral [cyathial] glands that fused in a ring-like structure, funnel-shaped involucres, and rudimentary caruncles (Brown et al. 1909; Carter 1988; Carter and Leach 2001). *Euphorbiambuinzauensis* is distinct, however, from other species in *Synadenium* group with strong morphological and phylogenetic support.

We show the monophyly of the previously segregated genus *Synadenium*, using phylogenetic inference based on a nrDNA dataset. Nevertheless, a more representative sampling of sect. Monadenium, especially of those species that belonged traditionally to *Monadenium*, is needed to draw this conclusion with greater confidence. It is worth mentioning that the phylogenetic relationships among the species in *Synadenium* group exhibited extremely short branches (Figure 1B), indicating that this lineage is likely to have radiated very recently. Interestingly, E.bicompactavar.rubra did not form a clade with E.bicompactavar.bicompacta as expected, suggesting a problem in its taxonomic placement. A comprehensive monograph based on an extensive study of specimens and a broad phylogenetic sampling is needed before the *Synadenium* group can be fully understood.

### Taxonomic treatment

#### Euphorbia mbuinzauensis

Taxon classificationPlantaeMalpighialesEuphorbiaceae

N. Wei, Mwachala, G.W. Hu & Q.F. Wang sp. nov.

1EA02345-886A-56E8-920D-05ABDF9806C2

urn:lsid:ipni.org:names:77220553-1

Figures 2 , 3

##### Type.

Kenya. Makueni County, Mbuinzau, 2°23'25.56"S, 37°54'42"E, 970 m, 29 Sep. 2018, *Sino-Africa Joint Investigation Team (SAJIT) 007200* (holotype HIB!; isotypes EA!, HIB!, K!)

##### Diagnosis.

*Euphorbiambuinzauensis* is most similar to *E.pseudomollis*, from which it differs by its shorter habit, up to 4 m (vs. to 9 m), abaxial leaves surfaces with densely stellate hairs (vs. simple hairs), 2- to 4-forked cymes (vs. 1- to 2-forked), bracts ca. 2.5 × 3.0 mm (vs. ca. 3.5 × 3.5 mm), dentate margin on bract (vs. entire or with a few teeth), cyathia 6 mm wide (vs. 8 mm wide), gland without narrow smooth margin (vs. with a narrow smooth margin), fruits ca. 8 × 7 mm (vs. ca. 10 × 10 mm), and ovoid seeds ca. 1.8 × 2.2 mm (vs. subglobose, ca. 2.5 × 2.5 mm).

**Figure 2.**
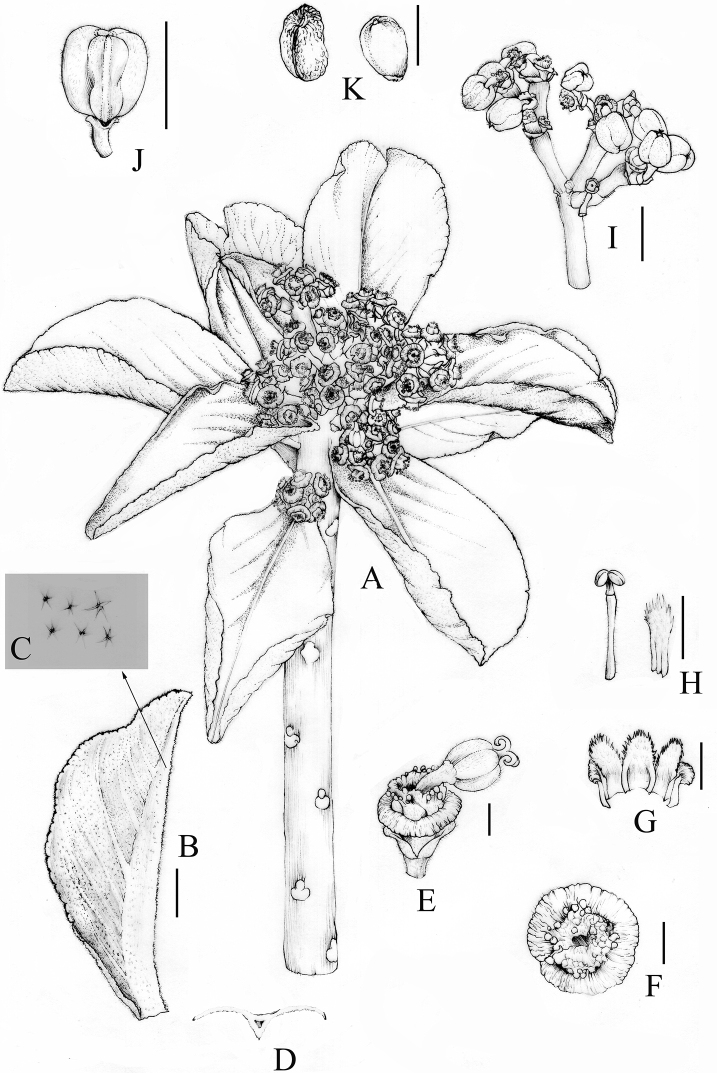
*Euphorbiambuinzauensis***A** flowering branch **B** leaf **C** the close-up of abaxial leaf to show densely stellate hairs **D** section through leaf to show the distinct keel on midrib beneath **E** cyathium, lateral view **F** cyathium, top view **G** dissected involucre to show lobes **H** male flower and bracteole **I** infructescence **J** fruit. K. Seeds. Scale bars: 2 mm (**C–H, K**); 1 cm (**B, I, J**). Voucher specimen: *SAJIT 007200*. Drawn by Nan Jia.

##### Description.

Many-branched shrub to 4 m high. Branches cylindrical, fleshy, and marked with prominent elliptic leaf scars. **Leaves** fleshy, subsessile, deciduous at fruiting stage, with a pair of reduced stipules; lamina subrotund to obovate, to ca. 14 × 6 cm, apex rounded to obtuse with a recurved tip, margin slightly undulate, crenate, midrib distinctly keeled beneath, blade inflated, incurved, adaxial surfaces densely pubescent, green, turning to purplish grey green during dry season, abaxial surfaces densely stellate hairy, occasionally tinged purplish, with stellate hairs along the midrib. **Inflorescences** monoecious, in dense pseudo-umbels of 2–5 cymes on pubescent peduncles to 4 cm long; cymes 2–4-forked, with pubescent branches to 1.8 cm long; bracts subquadrate, ca. 2.5 × 3.0 mm, dentate, densely pubescent. **Cyathia** ca. 2.5 × 6.0 mm, with broadly funnel-shaped involucres, pubescent below; glandular rim ca. 1.2 mm wide, deeply furrowed and wrinkled, purplish red, mostly shallowly notched on the one side, but with a deep notch when young; lobes subquadrate, ca. 2.0 × 2.5 mm, purplish red, pubescent. **Male flowers**: staminate flowers 0.8 mm long, enclosed by involucral lobes and bracteoles; bracteoles fan-shaped, laciniate, plumose, 3 mm long, with pedicels minutely pubescent; pedicels 3 mm long. **Female flowers**: styles ca. 2 mm long, connate at the base, pubescent, with distinctly bifid thickened apices, deciduous in fruit. **Capsules** obovoid, deeply acutely lobed, apex depressed, to 8 × 7 mm, from purplish red to yellowish green, pubescent, explosively dehiscing septicidally and loculicidally into 3 2-valved cocci; pedicel recurved, pubescent, to 8 mm long; columella persistent, 6–7 mm long. **Seeds** ovoid, obscurely 4-angled, ca. 1.8 × 2.2 mm, pale brown to dark brown, shallowly tuberculate; caruncle rudimentary.

**Figure 3.**
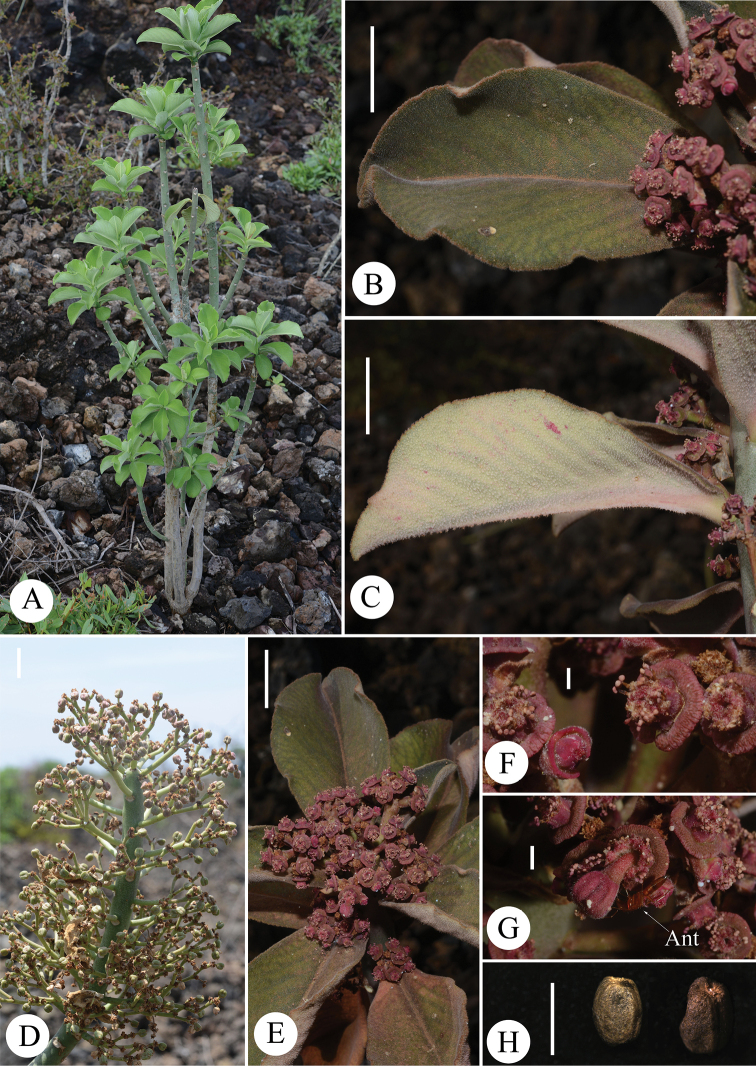
Morphological features of *Euphorbiambuinzauensis***A** habit **B** adaxial surface of leaf **C** abaxial surface of leaf **D** a branch with numerous infructescences **E** apical portion of flowering branch **F, G** Cyathium **H** seeds. Scale bars: 2 mm (**F–H**); 2 cm (**B–E**). Voucher specimens: *SAJIT 007411* (**A**), *SAJIT 007200* (**B–H**). Photo credit: Neng Wei.

##### Distribution and ecology.

**Figure 4.**
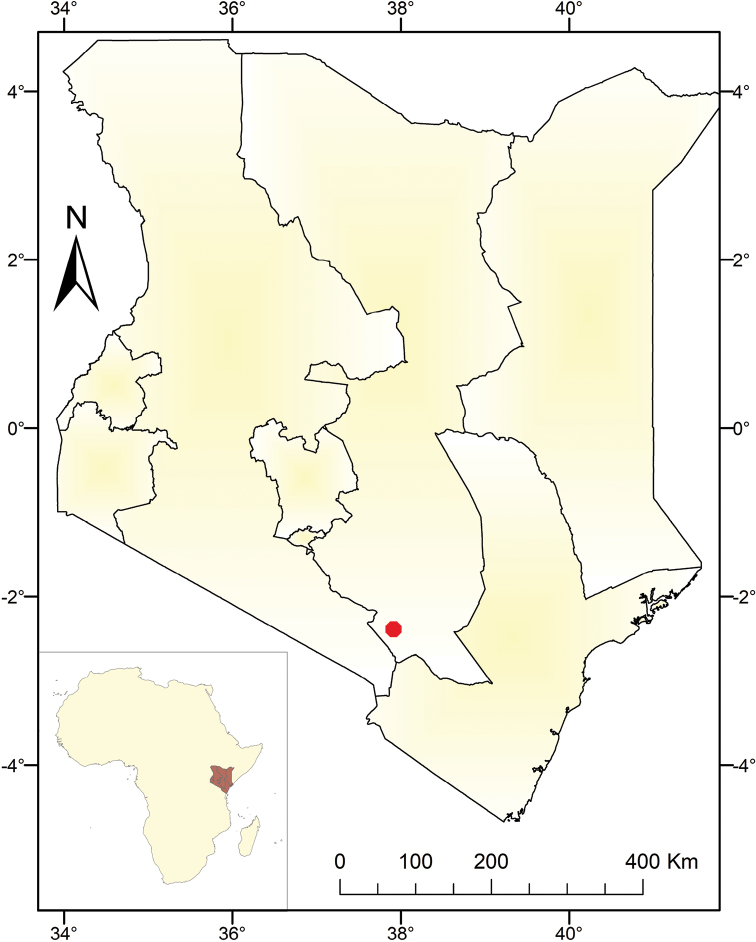
Known distribution of *Euphorbiambuinzauensis* in Kenya. The red dot indicates its only known locality.

Only one population of the new species was found at the foot of Mbuinzau hill, Makueni County, Kenya (Figure 4). Here it grows in open deciduous woodlands covered by lava outcrops at an elevation of ca. 970 m.

##### Conservation status.

Only one population of the new species was found in an isolated woodland covered by lava outcrops (Figure 3A) in Mbuinzau area. We assess the species as Endangered (criteria D1) according to the IUCN Red List Categories and Criteria (IUCN 2001), due to the number of mature individuals (< 250) in a very small and restricted population. Unfortunately, the habitat is threatened by human activities. For instance, its habitat has been fragmented by the Mombasa–Nairobi Railway and Mombasa–Road, which are the busiest traffic routes in Kenya. Moreover, the population is under threat from habitat loss, due to the exploitation of lava rocks in its habitat for construction.

##### Phenology.

*Euphorbiambuinzauensis* was found with flowers in September and with fruits from September to December.

##### Etymology.

The epithet “*mbuinzauensis*” refers to its type locality, Mbuinzau in Makueni County, Kenya.

##### Notes.

The conspicuous latex is extremely poisonous to cattle as well as human beings, according to the comments from local farmers. We observed ants visiting the flowers and they are a possible pollinator to this species (Figure 3G).

##### Additional specimens examined (*Paratypes*).

KENYA. Makueni County, Mbuinzau, 2°23'25.56"S, 37°54'42"E, elev. 970 m, 5 Dec. 2018, *SAJIT 007411* (EA!, HIB!).

**Specimens examined for *Euphorbiapseudomollis*. Kenya**. Makueni, Kibwezi, c. 1000 m, 2°26'S, 38°1'E, 22 Mar. 1906, *G.Scheffler 137* (isotypes AMD [AMD.66883] image!, BM [BM000911307]!, K [K000238424]!, S [S13-12929] image!, WAG [WAG0004308] image!); Taita-Taveta, Mwatate, 3°31'S, 38°24'E, 29 Apr. 1963, *P.R.O. Bally B12725* (BR [BR0000016225411] image!, K [K000238420]!); Taita-Taveta, Mwatate West of Voi, 3°30'S, 38°23'E, 15 Jul. 1960, *L.C. Leach & R. Bayliss 10258* (K [K000238421]! & [K000238422]!); Kitui, Mutomo Hill Plant Sanctuary, 900–1000 m, 1°51'S, 38°13'E, 2 May 1970, *J.B. Gillett 19141* (EA!, K [K000238423]!); Taita-Taveta, Taita, Sisal Estate, Senbi Hill, 1050 m, 3°31'S, 38°24'E, 17 Aug. 2000, *P.A. Luke & W.R.Q. Luke 6432* (EA!). **Tanzania**. Kilimanjaro, above Mwembe, 1005 m, 4°8'S, 37°51'E, 9 Apr. 1972, *B.J. Harris BJH6342* (K [K000238425]!); Kilimanjaro, Mwembe, 4°10'S, 37°51'E, unknown date, *P.R.O. Bally B11499 (E54)* (K [K000238426]! & [K000238427]!); Dodoma, Mpwapwa, 1128 m, 6°21'S, 36°29'E, 30 Jun. 1938, *Hornby 911* (K [K000238428]!); Tanga, Handeni, Kideleko, 609 m, 5°29'S, 38°1'E, 1 Jul. 1965, *M.E. Archbold 471* (K [K000238430]!); Tanga, Handeni, Chanika Village, 700 m, 5°25'S, 38°1'E, 23 Sep. 1979, *O. Hedberg et al. TMP194* (K [K000238431]!); Morogoro, Kilombero, Lugoda, 1800 m, 8°42'S, 35°49'E, Aug. 1988, *E. Adiheysen 224* (K [K000238432]!); Iringa, a little north of Morogoro road, 1554 m, 7°30'S, 36°10'E, 27 Feb. 1962, *R.M. Polhill & S. Paulo 1618* (BR [BR0000016225435] image!, EA!, K [K000238433]!, P [P00581481] image!); Lindi, 22 Apr. 1933, *H.J.Schlieben n6383* (BR [BR0000016225428] image!).

**Specimens examined for Euphorbiabicompactavar.bicompacta: Kenya**. Machakos, 1°31'S, 37°16'E, 7 Jun. 1902, *T. Kassner 956* (Holotype K [K000237846]!; Isotype BM [BM000911306]!); Taita-Taveta, Wusi, 1371 m, 3°27'S, 38°21'E, May 1931, *ERN 1322* (K [K000237843]!); Machakos, 1°31'S, 37°16'E, 27 Mar. 1940, *P.R.O. Bally E144* (K [K000237845]!); Makueni, Kibwezi, Sisal Estate, 914 m, 2°26'S, 38°1'E, Jun. 1943, *P.R.O. Bally B2573* (K [K000237847]!); Kitui, Migwani, 10 miles N of Migwani on Tharaka road, 0°57'18"S, 38°1'9"E, 3 May 1960, *D.M. Napper 1596* (BR [BR0000016224810] image!, EA!, K [K000237848]!); Nairobi, Nairobi arboretum, 1768 m, 1°17'S, 36°49'E, Aug. 1932, *I.R. Dale 2887* (K [K000237849]!); Nairobi, Langata, 1°20'S, 36°46'E, 20 March 1963, *P.R.O. Bally B12659* (K [K000237850]!); Kiambu, Muguga, 1°11'S, 36°38'E, 8 Jun. 1962, *J. Gichuru 14* (K [K000237851]!); Nyeri, Karatina, 0°29'S, 37°8'E, 24 Apr. 1943, *P.R.O. Bally B2541* (EA!, K [K000237852]!); Embu, Thuchi, crossing on Embu-Meru road, 760 m, 0°25'S, 37°52'E, 4 Apr. 1970, *J.B. Gillett & B. Mathew 19063* (BR [BR0000016224803] image!, K [K000237853]!); Laikipia, Kisima farm, 1700 m, 0°30'S, 36°30'E, 14 Jun. 1972, *P.R.O. Bally B15106* (K [K000237854]!); Taita-Taveta, Msau River Valley, 800–950 m, 3°24'S, 38°24'E, 18 May 1985, *C.H.S. Kabuye et al. 743* (K [K000237855]!); Taita-Taveta, Kasigau Mountain, 1000 m, 3°50'S, 38°40'E, 31 May 1998, *W.R.Q. Luke et al. 5344* (K [K000237856]!); Elgeyo Marakwet, Arror lower, Pt 203, 1050 m, 1°0'55"S, 35°37'27"E, 30 Jul. 2017, *Mwadime N 1861* (EA!).

## Supplementary Material

XML Treatment for Euphorbia mbuinzauensis

## Supplementary materials

Supplementary material 1 Table S1 Data type docx. file Explanation note Accessions of nuclear ribosomal DNA (nrDNA) in *Euphorbia* used in this study. (SCBG indicates Shanghai Chenshan Botanical Garden, Chinese Academy of Sciences). https://binary.pensoft.net/file/597942This dataset is made available under the Open Database License (http://opendatacommons.org/licenses/odbl/1.0/). The Open Database License (ODbL) is a license agreement intended to allow users to freely share, modify, and use this Dataset while maintaining this same freedom for others, provided that the original source and author(s) are credited.Neng Wei, Fredrick Munyao Mutie, Geoffrey Mwachala, Olwen M. Grace, Guang-Wan Hu, Qing-Feng Wang

Supplementary material 2 File 2 Data type docx. file Explanation note The trimmed alignment of the 17 complete nuclear ribosomal DNA (nrDNA) sequence dataset in nexus format. https://binary.pensoft.net/file/597943This dataset is made available under the Open Database License (http://opendatacommons.org/licenses/odbl/1.0/). The Open Database License (ODbL) is a license agreement intended to allow users to freely share, modify, and use this Dataset while maintaining this same freedom for others, provided that the original source and author(s) are credited.Neng Wei, Fredrick Munyao Mutie, Geoffrey Mwachala, Olwen M. Grace, Guang-Wan Hu, Qing-Feng Wang
