# Supplementary material for: Euphorbiambuinzauensis, a new succulent species in Kenya from the Synadenium group in Euphorbiasect.Monadenium (Euphorbiaceae)
Source: PhytoKeys. 2021 Oct 11;183:21–35. doi: 10.3897/phytokeys.183.70285 (PMC8523493; doi:10.3897/phytokeys.183.70285)
Supplement: Supplementary material 1 — Table S1 [file phytokeys-183-021-s001.docx]

**Supplementary File 1.** Accessions of nuclear ribosomal DNA (nrDNA) in *Euphorbia* used in this study. (SCBG indicates Shanghai Chenshan Botanical Garden, Chinese Academy of Sciences)

| Species | Voucher | Coverage times | NrDNA length (bp) | GenBank accession |
| --- | --- | --- | --- | --- |
| *Euphorbia bicompacta var. bicompacta* 1 | *V.Kathambi 0038* (EA; HIB) | 476.0 | 10,548 | MZ351238 |
| *Euphorbia bicompacta* var. *bicompacta* 2 | *SAJIT 007410* (EA; HIB) | 467.9 | 10,539 | MZ351242 |
| *Euphorbia bicompacta* var. *rubra* | *SAJIT 007451* (EA; HIB) | 489.8 | 10,629 | MZ351237 |
| *Euphorbia kirkii* | *D.J.Goyder, A.Massingue & J.Timberlake 4088* (K) | 495.7 | 10,665 | MZ351247 |
| *Euphorbia mbuinzauensis* | *SAJIT 007411(*EA;HIB*)* | 479.0 | 10,547 | MZ351232 |
| *Euphorbia neocymosa* | *SAJIT 007481*(EA; HIB) | 490.0 | 10,656 | MZ351235 |
| *Euphorbia neoglabrata* | *P.Lovett & C.Kaymbo 183* (EA; K) |  | 10,539 | MZ351244 |
| *Euphorbia neoglaucescens* | *P.R.O.Bally 1941* (EA; K) | 472.6 | 10,633 | MZ351239 |
| *Euphorbia neogossweileri* | *N.Wei 1036* (HIB), from cultivation at SCBG | 497.8 | 10,660 | MZ351236 |
| *Euphorbia neospinescens* | *N.Wei 1001* (HIB), from cultivation at SCBG | 472.5 | 10,643 | MZ351231 |
| *Euphorbia pereskiifolia* | *SAJIT 007428* (EA; HIB) | 484.0 | 10,634 | MZ351234 |
| *Euphorbia pseudomollis* | *M.B.Watuma et al. 0104* (EA; HIB) | 488.9 | 10,641 | MZ351233 |
| *Euphorbia resinifera* | *N.Wei 1048* (HIB) | 430.5 | 10,306 | MZ351243 |
| *Euphorbia* sp. 1 | *Bally 11499* (E54) (K) | 482.3 | 10,677 | MZ351246 |
| *Euphorbia* sp. 2 | *S.Bidgood et al. 1187* (EA; K) | 524.9 | 10,678 | MZ351245 |
| *Euphorbia syncameronii* | *L.C.Leach 5111* (K) | 457.6 | 10,624 | MZ351240 |
| *Euphorbia umbellata* | *SAJIT 007469* (EA; HIB) | 494.2 | 10,639 | MZ351241 |
