# Supplementary material for: Euphorbiambuinzauensis, a new succulent species in Kenya from the Synadenium group in Euphorbiasect.Monadenium (Euphorbiaceae)
Source: PhytoKeys. 2021 Oct 11;183:21–35. doi: 10.3897/phytokeys.183.70285 (PMC8523493; doi:10.3897/phytokeys.183.70285)
Supplement: Supplementary material 2 — File 2 [file phytokeys-183-021-s002.docx]

**Supplementary File 2.** The trimmed alignment of the 17 complete nuclear ribosomal DNA (nrDNA) sequence dataset in nexus format.

#NEXUS

BEGIN DATA;

dimensions ntax=17 nchar=10605;

format missing=?

datatype=DNA gap= -;

matrix

Euphorbia sp.2 TTTTTTCAAGTTTTCCGGGCCTTCCTCAAAATGGAAAAAGATATCGCCCATGTCCGAAAAGTCCCGGTATTACATGTAAATGTCCCTTTGAAAATGTTCGAACGGTGGTCGAAATTTCAACAAAAAGTGAAGGCTTTACATTTTTTTTTCCATTTTTTCCGAAACCCTTAAATATGGAAAAAATAACCCCCATGTCCGGAAAGCCCCGGTATTACATGTAAATGTCCCCTAAATAATGCTCGAACCGTGGTAAAAATTTCAGCTAAAAATGAAGGCTTTACATATTTTTTGCCATTTCCCCCGTAATTCCTAAAAATGGAAAAAATGCCCCCGTCGGGCGGATTGGCCCGATATTACACGTCAATTTCCCCCAAATCATGCTCGAACTGGGGTCGGAATTTCGGCAAAAAAGGAAGGCTCTGCATATTTTTTTCCATTTTTCCCGGAATTCTTAAAAATGGGAAAAATATCCCCCACGTCCGGAAACTCCCGATATCAAGTGTAAATGTCCCTCAAATCATGCTCGAACCGTGGTAAAAATTTCAGCGAAAAATGAAGGCGTTGCATATTTTTTTCCATTTTTCCAGTGCCCGGAATTCACAAAAATGGAAAAAATAGCCCCGTTGTCCGGGAAGTCCCGGGATTGCATGTAAATTTCCCTTAAATCATGCTCGAACTATGGTAAAAATTTCAGAAAAAATCTCCAAGTATAGAGCAGTTTTTGGGGGGGTGTGGCTCCTGGAACAAATCGATGTCCGTTCCTCCCAGAGCTGGATTTGTCATAAGAAATACTATAGGGGCTGCGCCAGCTCTCAACCTTGGCCCGCACTGGGCTGCGGGCCCGTGGTGGGTGCCTCCCGCATGCTCTCCCGCATTCAAGCGCATCACTCGGTCTCCCAGAAAGTCCCGACCCGCCGCCCCACCGAACGGAAGAATATCGTGGAAAAGCTAAGCCCAAAAGCACCACACGCGCGCGCGCGACGGCGCGGCGCGTTCGCGATTTAGGCGATTTAGGCACTTGGCACTTAGCACGTAGCACGAAGCGCGAGGCGCGAGGCCAATCGGCGGAACATAGCATAAAATATCCCGCAAAATTTATAAACGAGTAACGACACGGCATGAAAAAGAAGGTAGCTAGCAAAAATTTTTAAAAAAAGAAAGGAACATGAAGGCCAACCCGAAGTTCCGGCAAGAGTGCAGGGCGGCCTGGCCCGCCTTCGTGTGCGGGAGCAGGCGGGACAGCGTGCAGCGCTGTCCCGCATTCCTGCCTGTGCCTGGCGAGGCTAGGCAGGACGGCCGGCGTGCTGGCTTGCACGCTAGTGCCGCACGTCTAGTGTGCAGGAGGGCTGGCCCGCCTGGGCAGGGCTGGCAAGCTGCCCTGGCTCGCCTGAGTGCATGGCGGGGGAGGGCAAGCTGGCCAGCACGCAGCCCTGCCTGGCTCGCCAGCCAGCACCACGCAGGCAGGCCGCCAACCAGAGGTCAGCATAGGCATGCCAGCACGCCCAGCCGAGCTCTGCCGGCGGTGCTGACCCAGTCCGCGAAAAGGAGGCTAGGTCAGCGTGCTGTCTCGCCTGGCCGAGGCTCAGGATGGCTTCGCTGGGCCCGATCCAAATTTCGTTTGCAAAACCGTCGCACAGATAATAAGTTTTCCCGCACCATATTTTTTATACTGACTTTTACCATTTATTAGGAATTTTGTACCGAGTTCGTGATGCATTTGTTGAGTTTATATATTTTTTTGTATTTTTACGATTCTCGGACTTCGTAAATATGGAAAAAATACCTCCCGTGGGTAAAAGGTTACAATATTATATGGGAAAATCCCTTAATTCATGTTCTATCTACAGTCAAAATTTCAGGAAAAAACTCGAAGGTTTGACCGGTTTTTAAGGGGGTGTGGCTCCTGGAACAAATCGATGTCTGTTCCTCCCAGAGCTGGAAATGCTATAAGGACTACTATAGGGGGGTACACCTGCTCTCAACCCAGGCCACCACTGGGGCTGCGGGCCCGTGTTGGGTGCTTGCCGCATGCTCACCCCCTATCATGCGGGGAATTCAGCCACTAAGAACGTCCCGACCCGCCGCCCCTCCTCCGGCCGCCGGCCGCCGCCCCGCGCGGTGGCCGGAAAATCCAAAATTCTTAGAACGCTGAATTCCGCACCCCGAGAGCCCCTTTTCCCCCTGCCATTGTCGGAACTCGTTCGAATTGGGGGTAAAGTGGGTTTTCGGAGGCAACAACGTGCCCCGCGGCGCTGTCCCGATCGCTAGTGTGTGCCTACTCGTAGTTTTGTCTTGCATCGGATAGCTGATTGAGCTGCTCTCAGTGTGTATGGTGCTCGACTTCAAGCTGCTTTGGAAGCACGCCTAGAGTGAGGGGTAGCCGCTTGCTGCGCACGGTATCGGACGAGGGAAAAAGGAATTTCGGAAAAAAACGTTTCCGTGCCGTGCGCGATGTTGCTACATCCGTAGTTGCTTGCCGCGTGCGGTGTGGGGCGATGTAAAAAATGGAATTTGAAAATAAATTGTTTTCACGGAGTGCGCTTCGGCGTTGCCCCGATCGATAGTGCGTGTTGCTCGGTGGATTTGTTTGGCATTGGATAGCTGATCGAGTTGCTCTCGGTGTGAAGGTGCTGGACGTTGGTCCACTCGGCAGCCCGCCTTGAGTGAGGGGCAGTTGGTTGATGCGTGCGGTGCCAAGTGGCGAAAAAATGGATTTCACTAAAAATGTCTTTCTTGCTGTGCGCTCAGGCACCGTCCCGATCGGTAGTGTGCGTTATGTCCGGTGGTTCTGTTTGGCATCTGATAGCTGATCGAGTCGCTCTCAGTGTGTAAGGTGCTGGGTTTTGGACTATTTGGCTTGCCTGCCTATCGTGAGGAGCGGTTGCTTGGTGCGTACAGTTCCGGACGGCGAAAAATTGAATTCCAATAAAATATTATTCTCTCAACACGTGCTACTTTATCATGCGGTAAGGAATGTTCTCTCGCACACAGCGGTTCGGGCGATGTCTCTACTCGACGTTTCGGCACTGCTTGATTCGTTCTCGAAGACAGCAGTGCAGTTCGGGGGGTGGGGATGTTGCTCAATATACGCGGCGGTGCATGAGTGGTAAATAGGCCATTGGGGTTGGCAGGCTCTGTGCTAGCGCATCGAACTGTCGTACCTTGAGGCCATTCAGTGGTGTCCCGGAGGCGTATTGCTATGTCGGGCGGGGATGGTTTCTGTGTTGCATACCCGCGCAGTGGAATGGAATTTTGTTGCCAAGAAACATTCGTCCCGTGCCCTTTTAGGGGCGTCGGATGAACCATGCAGCAGCTCTCGTGTGCCGGGCATGCCTTTTTGGCTTCTCTGGCACATGTGAAGGTGCTCGTGCTCTCGGATGCGGAATGCTTTTGCGAGAGGAGGGATTGAGTTTCCTTTATGTGTTCTCGCTGTCCCTACATAAGAACCACCGTCCTTTCCGCACAGTGGCCTTGGTTGCTGCGGTGTACTATGTCTGCTTGCGGGTTAGGACGGCATGGAGGAATGCTACCTGGTTGATCCTGCCAGTAGTCATATGCTTGTCTCAAAGATTAAGCCATGCATGTGTAAGTATGAACTAATTCAGACTGTGAAACTGCGAATGGCTCATTAAATCAGTTATAGTTTGTTTGATGGTACCTGCTACTCGGATAACCGTAGTAATTCTAGAGCTAATACGTGCAACAAACCCCGACTTCTGGAAGGGATGCATTTATTAGATAAAAGGTCGACGCGGGCTCTGCCCGTTGCTCTGATGATTCATGATAACTCGACGGATCGCACGGCCATCGTGCTGGCGACGCATCATTCAAATTTCTGCCCTATCAACTTTCGATGGTAGGATAGAGGCCTACCATGGTGGTGACGGGTGACGGAGAATTAGGGTTCGATTCCGGAGAGGGAGCCTGAGAAACGGCTACCACATCCAAGGAAGGCAGCAGGCGCGCAAATTACCCAATCCTGACACGGGGAGGTAGTGACAATAAATAACAATACCGGGCTCTTCGAGTCTGGTAATTGGAATGAGTACAATCTAAATCCCTTAACGAGGATCCATTGGAGGGCAAGTCTGGTGCCAGCAGCCGCGGTAATTCCAGCTCCAATAGCGTATATTTAAGTTGTTGCAGTTAAAAAGCTCGTAGTTGGACCTTGGGTTGGGTCGACCGGTCCGCCTTACGGTGTGCACCTGTCGGCTCGTCCCTTCTGCCGGCGATGCGCTCCTGGCCTTAACTGGCCGGGTCGTGCCTCCGGCGCTGTTACTTTGAAGAAATTAGAGTGCTCAAAGCAAGCCTACGCTCTGTATACATTAGCATGGGATAACATCATAGGATTTCGGTCCTATTCTGTTGGCCTTCGGGATCGGAGTAATGATTAACAGGGACAGTCGGGGGCATTCGTATTTCATAGTCAGAGGTGAAATTCTTGGATTTATGAAAGACGAACAACTGCGAAAGCATTTGCCAAGGATGTTTTCATTAATCAAGAACGAAAGTTGGGGGCTCGAAGACGATCAGATACCGTCCTAGTCTCAACCATAAACGATGCCGACCAGGGATCGGCGGATGTTGCTTTTAGGACTCCGCCGGCACCTTATGAGAAATCAAAGTCTTTGGGTTCCGGGGGGAGTATGGTCGCAAGGCTGAAACTTAAAGGAATTGACGGAAGGGCACCACCAGGAGTGGAGCCTGCGGCTTAATTTGACTCAACACGGGGAAACTTACCAGGTCCAGACATAGTAAGGATTGACAGACTGAGAGCTCTTTCTTGATTCTATGGGTGGTGGTGCATGGCCGTTCTTAGTTGGTGGAGCGATTTGTCTGGTTAATTCCGTTAACGAACGAGACCTCAGCCTGCTAACTAGCTATGCGGAGGTATCCCTCCGCGGCCAGCTTCTTAGAGGGACTATGGCCTTCTAGGCCAAGGAAGTTTGAGGCAATAACAGGTCTGTGATGCCCTTAGATGTTCTGGGCCGCACGCGCGCTACACTGATGTATTCAACGAGTCTATAGCCTTGGCCGACAGGCCCGGGTAATCTTTGAAATTTCATCGTGATGGGGATAGATCATTGCAATTGTTGGTCTTCAACGAGGAATTCCTAGTAAGCGCGAGTCATCAGCTCGCGTTGACTACGTCCCTGCCCTTTGTACACACCGCCCGTCGCTCCTACCGATTGAATGGTCCGGTGAAGTGTTCGGATCGCGGCGACGTGGGCGGTTCGCCGCCGGCGACGTCGCGAGAAGTCCACTGAACCTTATCATTTAGAGGAAGGAGAAGTCGTAACAAGGTTTCCGTAGGTGAACCTGCGGAAGGATCATTGTCGAAACCTGCCAGCAGAATGACCCGCGAACGTGTTTATAAATCGAGGGGCCGCTGCAGGATTCATCCAGCGATGGCACCTCACTAGGGCCCTGGCAGGGGATGCGGTGCGGTGGGATCCACCGTTCCCTGCGATCTCCTGTTTGCGGCCTATTAACAAAACCCCGGCGCCGTACGCGCCAAGGAATTGTAAAAAAAGATTGTGCAGCCCGATCGCACTGGCAACGGTGTGGCGGGTTTCACTGCGCTTTGAGAACCAAAATGACTCTCGGCAACGGATATCTCGGCTCTCGCATCGATGAAGAACGCAGCGAAATGCGATACTTGGTGTGAATTGCAGGATCCCGCGAACCATCGAGTCTTTGAACGCAAGTTGCGCCCGAAGCCTTTCGGCCGAGGGCACGTCTGCCTGGGTGTCACTCAAACGTCGCTCCAAACCCCTTCCATCGGGAGGGGTATGCGGGGCGGATGCTGGCCTCCCGTGTGCGTATCGCTCGCGGTTGGCCGAAATTCCTAGTCCTCGGCACGACGCCACGGAATCGGTGGTTGCAAGACCCTCGGAGAAAGCCTTGTGCGCTTGTAAGCCCTTTCGGACCATGAGACCCCAGAGCGTACCTAGCACTGCGACCCCAGGTCAGGCGGGATTACCCGCTGAGTTTAAGCATATCAATAAGCGGAGGAAAAGAAACTTACCAGGATTCCCCTAGTAACGGCGAGCGAACCGGGAAGAGCCCAGCTTGAGAATCGTGCGCCTGCGGCGTTCGAATTGTAGTCTGGAGAAGCGTCCTCAGCGGCGGACCGGGCCCAAGTCCCCTGGAAGGGGGCGCCGGAGAGGGTGAGAGCCCCGTCGTGCCCGGACCCTGTCGCACCACGAGGCGCTGTCTACGAGTCGGGTTGTTTGGGAATGCAGCCCAAATCGGGCGGTAAATTCCGTCCAAGGCTAAATATGGGCGAGAGACCGATAGCGAACAAGTACCGCGAGGGAAAGATGAAAAGGACTTTGAAAAGAGAGTCAAAGAGTGCTTGAAATTGTCGGGAGGGAAGCGGATGGGGGCCGGCGATGCGCCCCGGTCGGATGTGGAACGGTGACAAGCCGGTCCGCCGATCGGCTCGGGGCGCGGACCGATACGGATTGAGGCGGCGGCGTAAGCCCAGGAATTTGAAACGCCTGTGGAGATGCCGTCGCAGCAATCGTGGAAAGCAGCACGCGCCGTCTCGGCGTGCCTCGGCACCTGCGTGCTACTGGTGTCGGCCAGCGGGCTCCCCATTCGGCCCGTCTTGAAACACGGACCAAGGAGTCTGACATGTGTGCGAGTCAACGGGCGAGTAAACCCGTAAGGCGCAAGGAAGCTGACTGGCGGGATCCCCTAGAGGGTTGCACCGCCGACCGACCTTGATCTTCTGAGAAGGGTTCGAGTGAGAGCATGCCTGTCGGGACCCGAAAGATGGTGAACTATGCCTGAGCGGGGCGAAGCCAGAGGAAACTCTGGTGGAGGCCCGCAGCGATACTGACGTGCAAATCGTTCGTCTGACTTGGGTATAGGGGCGAAAGACTAATCGAACCGTCTAGTAGCTGGTTCCCTCCGAAGTTTCCCTCAGGATAGCTGGAGCTCGGAACGAGTTCTATCGGGTAAAGCCAATGATTAGAGGCATCGGGGGCGCAACGCCCTCGACCTATTCTCAAACTTTAAATAGGTAGGACGGCGCGGCTGCTTCGTTGAGCCGCGCCACGGAATCGAGAGCTCCAAGTGGGCCATTTTTGGTAAGCAGAACTGGCGATGCGGGATGAACCGGAAGCCGGGTTACGGTGCCCAACTGCGCGCTAACCTAGAACCCACAAAGGGTGTTGGTCGATTAAGACAGCAGGACGGTGGTCATGGAAGTCGAAATCCGCTAAGGAGTGTGTAACAACTCACCTGCCGAATCAACTAGCCCCGAAAATGGATGGCGCTTAAGCGCGCGACCTATACCCGGCCGTCGGGGCAAGAGCCAGGCCCCGATGAGTAGGAGGGCGCGGCGGTCGCTGCAAAACCCAGGGCGCGAGCCCGGGCGGAGCGGCCGTCGGTGCAGATCTTGGTGGTAGTAGCAAATATTCAAATGAGAACTTTGAAGGCCGAAGAGGGGAAAGGTTCCATGTGAACGGCACTTGCACATGGGTTAGTCGATCCTAAGAGACGGGGGAAGCCCGTCCGACAGCGCGTCCGCGCGCGAGCTTCGAAAGGGAATCGGGTTAAAATTCCCGAACCGGGACGCGGCGGCTGACGGCAACGTTAGGGAGTCCGGAGACGTCGGCGGGGGCCTCGGGAAGAGTTATCTTTTCTGTTTAACAGCCCGCCCACCCTGGAAACGACTCAGTCGGAGGTAGGGTCCAGCGGCTGGAAGAGCACCGCACGTCGCGCGGTGTCCGGTGCGCCCCCGGCGGCCCGTGAAAATCCGGAGGACCGAGTGCCATCCACGCCCGGTCGTACTCATAACCGCATCAGGTCTCCAAGGTGAACAGCCTCTGGTCGATGGAACAATGTAGGCAAGGGAAGTCGGCAAAATGGATCCGTAACCTCGGGAAAAGGATTGGCTCTGAGGGCTGGGCCCGGGGGTCCCAGTCCCGAACCCGTCGGCTGTCGGCGGACTGCTCGAGCTGCTCCCGCGGCAAGAGCGGGTCGCTGCGTGCCGGCCGGGGGACGGATTGGGAACGGCCCCTCTGGGGGCCTTCCCCGGGCGTCGAACAGTCGACTCAGAACTGGTACGGACAAGGGGAATCCGACTGTTTAATTAAAACAAAGCATTGCGATGGTCCCTGCGGATGCTAACGCAATGTGATTTCTGCCCAGTGCTCTGAATGTCAAAGTGAAGAAATTCAACCAAGCGCGGGTAAACGGCGGGAGTAACTATGACTCTCTTAAGGTAGCCAAATGCCTCGTCATCTAATTAGTGACGCGCATGAATGGATTAACGAGATTCCCACTGTCCCTGTCTACTATCCAGCGAAACCACAGCCAAGGGAACGGGCTTGGCAGAATCAGCGGGGAAAGAAGACCCTGTTGAGCTTGACTCTAGTCCGACTTTGTGAAATGACTTGAGAGGTGTAGTATAAGTGGGAGCCGGAAACGGCGATAGTGAAATACCACTACTTTTAACGTTATTTTACTTATTCCGTGAATCGGAGGCGGGGCATTGCCCCTCTTTTTGGACCAAAGGCCGCTTCGCGGTCGATCCGGGCGGAAGACATTGTCAGGTGGGGAGTTTGGCTGGGGCGGCACATCTGTTAAAAGATAACGCAGGTGTCCTAAGATGAGCTCAACGAGAACAGAAATCTCGTGTGGAACAAAAGGGTAAAAGCTCGTTTGATTCTGATTTCCAGTACGAATACGAACCGTGAAAGCGTGGCCTATCGATCCTTTAGACCTTCGGAATTTGAAGCTAGAGGTGTCAGAAAAGTTACCACAGGGATAACTGGCTTGTGGCAGCCAAGCGTTCATAGCGACGTTGCTTTTTGATCCTTCGATGTCGGCTCTTCCTATCATTGTGAAGCAGAATTCACCAAGTGTTGGATTGTTCACCCACCAATAGGGAACGTGAGCTGGGTTTAGACCGTCGTGAGACAGGTTAGTTTTACCCTACTGATGACGGTGTCGCGATGGTAATTCAACCTAGTACGAGAGGAACCGTTGATTCGCACAATTGGTCATCGCGCTTGGTTGAAAAGCCAGTGGCGCGAAGCTACCGTGCGCTGGATTATGACTGAACGCCTCTAAGTCAGAATCCGGGCCAGAAGCGATGCATGCGTCCGCCGCTCGTTTGCCGACCCTCAGTAGGGGCCATCCGGCCCCCAAAGGCACGTGTCGTTGGCTAAGCCCTCGCGGCAGACAAGCCGTGCGGGCAGCCTTGAAGTACAATTCCCACCGGGCGGCGGGCAGAATCCTTTGCAGACGACTTAAATACGCGACGGGGTATTGTAAGTGGCAGAGTGGCCTTGCTGCCACGATCCACTGAGATTCAGCCCTTTGTCGCTCCGATTCGTCCCTCCCTCCCAAGAAACTTTTTCCATCTCAATAGAATATCGAGCGGAGGCTGGGGTCTCGATTTCCGCTCGTAGAAAGAGGGCCAAGGAGAGAACCCCGGTATTGCATATGGCACGGGGGAAGCAAGAGATTAAGCACACCGCGGGGTGGAAAAAGCATCTGCGCACGGGCAGGTTAGAAAAACATACAAGCGATGCATCCCTTCAATGTCCCTCGGCATTGACCGCTTGTTCATTTTTTTTTCCGGCTTAATGCGTACGTTTCGAACCACTCGTACACCAAGGCTGCCGCGGCCACGCGTGCCTCACCGGTCGATGCGGTCTACTGCCTTCACAGGCGAGGCAGGAGCAGCATCGAATTCCTGCCCCGGCACCACGCACGAGGCCGGCGGTGCCTACCTCTCGTGCGCAAGCGAGACCGATGCCAGCGCGGCTGACTGGCCTGAATAGGAGAGGCCAGTGCTAGTCCGCGCACGCCACATGCCCCTTCAAAGTTTCCCCGCATCGCTCGAGAATTTTACGGCAGGCCCGTTGAACATCTCCATCGGGAGAGAGAATTTGCATCTCCGCCCCAAGCGTCGATGCTAGTCCGCGCACGCCACATGCCCCTTCAAAGTTTCCCCGCACCGCTCGAGAATTTTATGGCAGACCCGTTAAATATCTCCATCGGGAGAGAAAATTTGCATCTCCGCCCCCAACCGTCGAGAAAAGATCAACACTTTTACCACCCCGAGGGTTGTACACACAGCCTCCTGAAACAAAGGGGCGGCAAAAACGCGTTTCGCCTCCAGTCCACGCTCGGTCTTTCGGAACGGGTTCAACGGCAGTTGATTTTATACCGAGTTGCCAAATTCTCACGAGGGCTCTGTATTCTTTTTCGTTTGTCAAGTTCCCGGCAGTCTTAAATACGAGAAAGGCCATCTCGAAGGTCCCGGCATTCTCACGAAGGTTCTAAATATATATTTTCCTTTTTTCAAGAGCTAGGACTTCTTAAAAATGGAAAAAATTATCCCCCGTGTCCGGAAAGTCCCGGTAATGCATGTAGATTTCCCTTGAACCATGCTGGTCAGAATTTCTGCAAAAAAATGAAGGGTCTACGTATCTTCTGTCCATTTTTCCCGGACTCCCTAAAAATGGGAAAAATACCCCCCATGTTCGGAAAGTCCCGATATTACATGTAAATATCCCTTAAATCACACCCGAACTGTGGTCACAATTTCCGCAAAAAAAAAAAATTGAAGTCTTTACATAT

Euphorbia sp.1 TTTTTTCAAGTTTTCCGGGCCTTCCTCAAAATGGAAAAAGATATCGCCCATGTCCGAAAAGTCCCGGTATTACATGTAAATGTCCCTTTGAAAATGTTCGAACGGTGGTCGAAATTTCAACAAAAAGTGAAGGCTTTACATTTTTTTTTCCATTTTTTCCGAAACCCTTAAATATGGAAAAAATAACCCCCATGTCCGGAAAGCCCCGGTATTACATGTAAATGTCCCCTAAATAATGCTCGAACCGTGGTAAAAATTTCAGCTAAAAATGAAGGCTTTACATATTTTTTGCCATTTCCCCCGTAATTCCTAAAAATGGAAAAAATGCCCCCGTCGGGCGGATTGGCCCGATATTACACGTCAATTTCCCCCAAATCATGCTCGAACTGGGGTCGGAATTTCGGCAAAAAAGGAAGGCTCTGCATATTTTTTTCCATTTTTCCCGGAATTCTTAAAAATGGGAAAAATATCCCCCACGTCCGGAAACTCCCGATATCAAGTGTAAATGTCCCTCAAATCATGCTCGAACCGTGGTAAAAATTTCGGCGAAAAATGAAGGCGTTGCATATTTTTTTCCTTTTTTCCAGTGCCCGGAATTCACAAAAATGGAAAAAATAGCCCCGTTGTCCGGGAAGTCCCGGGATTGCATGTAAATTTCCCTTAAATCATGCTCGAACTATGGTAAAAATTTCAGAAAAAATCTCCAAGTATAGAGCAGTTTTTGGGGGGGTGTGGCTCCTGGAACAAATCGATGTCCGTTCCTCCCAGAGCTGGATTTGTCATAAGAAATACTATAGGGGCTGCGCCAGCTCTCAACCTTGGCCCGCACTGGGCTGCGGGCCCGTGGTGGGTGCCTCCCGCATGCTCTCCCGCATTCAAGCGCATCACTCGGTCTCCCAGAAAGTCCCGACCCGCCGCCCCACCGAACGGAAGAATATCGTGGAAAAGCTAAGCCCAAAAGCACCACACGCGCGCGCGCGACGGCGCGGCGCGTTCGCGATTTAGGCGATTTAGGCACTTGGCACTTAGCACGTAGCACGAAGCGCGAGGCGCGAGGCCAATCGGCGGAACATAGCAGAAAATATCCCGCAAAATTTATAAACGAGTAACGACACGGCATGAAAAAGAAGGTAGCTAGCAAAAATTTTAAAAAAAAGAAAGGAACATGAAGGCCAACCCGAAGTTCAGGCAAGAGTGCAGGGCGGCCTGGCCCGCCTTCGTGTGCGGGAGCAGGCGGGACAGCGTGCAGCGCTGTCCCGCATTCCTGCCTGTGCCTGGCGAGGCTAGGCAGGACGGCCGGCGTGCTGGCTTGCACGCTAGTGCCGCACGTCTAACCAGTGGGAGGGCTGGCCCGCCTGGGCAGGGCTGGCAAGCTGCCCTGGCTCGCCTGAGTGCATGGCGGGGGAGGGCAAGCTGGCCAGCACGCAGCCCTGCCTGGCTCGCCAGCCAGCACCACGCAGGCAGGCCGCCAACCAGAGGTCAGCATAGGCATGCCAGCACGCCCAGCCGAGCTCTGCCGGCGGTGCTCACCCAGTCCGCGAAAAGGAGGCTAGGTCAGCGTGCTGTCTCGCCTGGACGAGGCTCAGGATGGCTTCGCTGGGCCCGATCCAAATTTCGTTTGCAAAACCTTCGCACAGATAATAAGTTTTCCCGCACCATATTTTTTATACTGACTTTTACCATTTATTAGGAATTTTGTACCGAGTTCGTGATGCATTTGTTGAGTTTATATATTTTTTTGTATTTTTACGATTCTCGGACTTCGTAAATATGGAAAAAATACCTCCCGTGGGTAAAAGGTTACAATATTATATGGAAAAATCCCTTAATTCATGTTCTATCTACAGTCAAAATTTCAGGAAAAAACTCGAAGGTTTGACCGGTTTTTAAGGGGGTGTGGCTCCTGGAACAAATCGATGTCTGTTCCTCCCAGAGCTGGAAATGCTATAAGGACTACTATAGGGGGGTACACCTGCTCTCAACCCAGGCCACCACTGGGGCTGCGGGCCCGTGTTGGGTGCTTGCCGCATGCTCACCCCCTATCATGCGGGGAATTCAGCCACTAAGAACGTCCCGACCCGCCGCCCCTCCTCCGGCCGCCGGCCGCCGCCCCGCGCGGTGGCCGGAAAATCCAAAATTCTTAGAACGCTGAATTCCGCACCCCGAGAGCCCCTTTTCCCCCTGCCATTGTCGGAACTCGTTCGAATTGGGGGTAAAGTGGGTTTTCGGAGGCAACAACGTGCCCCGCGGCGCTGTCCCGATCGCTAGTGTGTGCCTACTCGTAGTTTTGTCTTGCATCGGATAGCTGATTGAGCTGCTCTCAGTGTGTATGGTGCTCGACTTCAAGCTGCTTTGGAAGCACGCCTAGAGGGAGGGGTAGCCGCTTGCTGCGCACGGTATCGGACGAGGGAAAAAGGAATTTCGGAAAAAAACGTTTCCGTGCCGTGCGCGATGTTGCTACATCCGTAGTTGCTTGCTGCGTGCGGTGTGGGGCGATGTAAAAAATGGAATTTGAAAATAAATTGTTTTCACGGAGTGCGCTTCGGCGTTGCCCCGATCGATAGTGCGTGTTGCTCGGTGGATTTGTTTGGCATTGGATAGCTGATCGAGTTGCTCTCGGTGTGAAGGTGCTGGACGTTGGTCCACTCGGCAGCCCGCCTTGAGTGAGGGGCAGTTGGTTGATGCGTGCGGTGCCAAGTGGCGAAAAAATGGATTTCACTAAAAATGTCTTTCTTGCTGTGCGCTCAGGCGCCGTCCCGATCGGTAGTGTGCGTTATGTCCGGTGGTTCTGTTTGGCATCTGATAGCTGATCGAGTCGCTCTCAGTGTGTAAGGTGCTGGGTTTTGGACTATTTGGCTTGCCTGCCTATCGTGAGGAGCGGTTGCTTGGTGCGTACAGTTCCGGACGGCGAAAAATTGAATTCCAATAAAATATTATTCTCTCAACACGTGCTACTTTATCATGCGGTAAGGAATGTTCTCTCGCACACAGCGGTTCGGGCGATGTCTCTACTCGACGTTTCGGCACTGCTTGATTCGTTCTCGAAGACAGCAGTGCAGTTCGGGGGGTGGGGATGTTGCTCAATATACGCGGCGGTGCATGAGTGGTAAATAGGCCATTGGGGTTGGCAGGCTCTGTGCTAGCGCATCGAACTGTCGTACCTTGAGGCCACTCAGTGGTGTCCCGGAGGCGTATTGCTATGTCGGGCGGGGATGGTTTCTGTGTCGCATACCCGCGCAGTGGAATGGAATTTTGTTGCCAAGAAACATTCGTCCCGTGCCCTTTTAGGGGCGTCGGATGAACCATGCAGCAGCTCTCGTGTGCCGGGCATGCCTTTTTGGCTTCTCTGGCACATGTGAAGGTGCTCGTGCTCTCGGATGCGGAATGCTTTTGCGAGAGGAGGGATTGAGTTTCCTTTATGTGTTCTCGCTGTCCCTACATAAGAACCACCGTCCTTTCCGCACAGTGGCCTTGGTTGCTGCGGTGTACTATGTCTGCTTGCGGGTTAGGACGGCATGGAGGAATGCTACCTGGTTGATCCTGCCAGTAGTCATATGCTTGTCTCAAAGATTAAGCCATGCATGTGTAAGTATGAACTAATTCAGACTGTGAAACTGCGAATGGCTCATTAAATCAGTTATAGTTTGTTTGATGGTACCTGCTACTCGGATAACCGTAGTAATTCTAGAGCTAATACGTGCAACAAACCCCGACTTCTGGAAGGGATGCATTTATTAGATAAAAGGTCGACGCGGGCTCTGCCCGTTGCTCTGATGATTCATGATAACTCGACGGATCGCACGGCCATCGTGCTGGCGACGCATCATTCAAATTTCTGCCCTATCAACTTTCGATGGTAGGATAGAGGCCTACCATGGTGGTGACGGGTGACGGAGAATTAGGGTTCGATTCCGGAGAGGGAGCCTGAGAAACGGCTACCACATCCAAGGAAGGCAGCAGGCGCGCAAATTACCCAATCCTGACACGGGGAGGTAGTGACAATAAATAACAATACCGGGCTCTTCGAGTCTGGTAATTGGAATGAGTACAATCTAAATCCCTTAACGAGGATCCATTGGAGGGCAAGTCTGGTGCCAGCAGCCGCGGTAATTCCAGCTCCAATAGCGTATATTTAAGTTGTTGCAGTTAAAAAGCTCGTAGTTGGACCTTGGGTTGGGTCGACCGGTCCGCCTTACGGTGTGCACCTGTCGGCTCGTCCCTTCTGCCGGCGATGCGCTCCTGGCCTTAACTGGCCGGGTCGTGCCTCCGGCGCTGTTACTTTGAAGAAATTAGAGTGCTCAAAGCAAGCCTACGCTCTGTATACATTAGCATGGGATAACATCATAGGATTTCGGTCCTATTCTGTTGGCCTTCGGGATCGGAGTAATGATTAACAGGGACAGTCGGGGGCATTCGTATTTCATAGTCAGAGGTGAAATTCTTGGATTTATGAAAGACGAACAACTGCGAAAGCATTTGCCAAGGATGTTTTCATTAATCAAGAACGAAAGTTGGGGGCTCGAAGACGATCAGATACCGTCCTAGTCTCAACCATAAACGATGCCGACCAGGGATCGGCGGATGTTGCTTTTAGGACTCCGCCGGCACCTTATGAGAAATCAAAGTCTTTGGGTTCCGGGGGGAGTATGGTCGCAAGGCTGAAACTTAAAGGAATTGACGGAAGGGCACCACCAGGAGTGGAGCCTGCGGCTTAATTTGACTCAACACGGGGAAACTTACCAGGTCCAGACATAGTAAGGATTGACAGACTGAGAGCTCTTTCTTGATTCTATGGGTGGTGGTGCATGGCCGTTCTTAGTTGGTGGAGCGATTTGTCTGGTTAATTCCGTTAACGAACGAGACCTCAGCCTGCTAACTAGCTATGCGGAGGTATCCCTCCGCGGCCAGCTTCTTAGAGGGACTATGGCCTTCTAGGCCAAGGAAGTTTGAGGCAATAACAGGTCTGTGATGCCCTTAGATGTTCTGGGCCGCACGCGCGCTACACTGATGTATTCAACGAGTCTATAGCCTTGGCCGACAGGCCCGGGTAATCTTTGAAATTTCATCGTGATGGGGATAGATCATTGCAATTGTTGGTCTTCAACGAGGAATTCCTAGTAAGCGCGAGTCATCAGCTCGCGTTGACTACGTCCCTGCCCTTTGTACACACCGCCCGTCGCTCCTACCGATTGAATGGTCCGGTGAAGTGTTCGGATCGCGGCGACGTGGGCGGTTCGCCGCCGGCGACGTCGCGAGAAGTCCACTGAACCTTATCATTTAGAGGAAGGAGAAGTCGTAACAAGGTTTCCGTAGGTGAACCTGCGGAAGGATCATTGTCGAAACCTGCCAGCAGAATGACCCGCGAACGTGTTTATAAATCGAGGGGCCGCTGCAGGATTCATCCAGCGATGGCACCTCACTAGGGCCCTGGCAGGGGATGCGGTGCGGTGGGATCCACCGTTCCCTGCGATCTCCTGTTTGCGGCCTATTAACAAAACCCCGGCGCCGTACGCGCCAAGGAATTGTAAAAAAAGATTGTGCAGCCCGATCGCACTGGCAACGGTGTGGCGGGTTTCACTGCGCTTTGAGAACCAAAATGACTCTCGGCAACGGATATCTCGGCTCTCGCATCGATGAAGAACGCAGCGAAATGCGATACTTGGTGTGAATTGCAGGATCCCGCGAACCATCGAGTCTTTGAACGCAAGTTGCGCCCGAAGCCTTTCGGCCGAGGGCACGTCTGCCTGGGTGTCACTCAAACGTCGCTCCAAACCCCTTCCATCGGGAGGGGTATGCGGGGCGGATGCTGGCCTCCCGTGTGCGTATCGCTCGCGGTTGGCCGAAATTCCTAGTCCTCGGCACGACGCCACGGAATCGGTGGTTGCAAGACCCTCGGAGAAAGCCTTGTGCGCTTGTAAGCCCTTTCGGACCATGAGACCCCAGAGCGTACCTAGCACTGCGACCCCAGGTCAGGCGGGATTACCCGCTGAGTTTAAGCATATCAATAAGCGGAGGAAAAGAAACTTACCAGGATTCCCCTAGTAACGGCGAGCGAACCGGGAAGAGCCCAGCTTGAGAATCGTGCGCCTGCGGCGTTCGAATTGTAGTCTGGAGAAGCGTCCTCAGCGGCGGACCGGGCCCAAGTCCCCTGGAAGGGGGCGCCGGAGAGGGTGAGAGCCCCGTCGTGCCCGGACCCTGTCGCACCACGAGGCGCTGTCTACGAGTCGGGTTGTTTGGGAATGCAGCCCAAATCGGGCGGTAAATTCCGTCCAAGGCTAAATATGGGCGAGAGACCGATAGCGAACAAGTACCGCGAGGGAAAGATGAAAAGGACTTTGAAAAGAGAGTCAAAGAGTGCTTGAAATTGTCGGGAGGGAAGCGGATGGGGGCCGGCGATGCGCCCCGGTCGGATGTGGAACGGTGACAAGCCGGTCCGCCGATCGGCTCGGGGCGCGGACCGATACGGATTGAGGCGGCGGCGTAAGCCCAGGAATTTGAAACGCCTGTGGAGATGCCGTCGCAGCAATCGTGGAAAGCAGCACGCGCCGTCTCGGCGTGCCTCGGCACCTGCGTGCTACTGGTGTCGGCCAGCGGGCTCCCCATTCGGCCCGTCTTGAAACACGGACCAAGGAGTCTGACATGTGTGCGAGTCAACGGGCGAGTAAACCCGTAAGGCGCAAGGAAGCTGACTGGCGGGATCCCCTAGAGGGTTGCACCGCCGACCGACCTTGATCTTCTGAGAAGGGTTCGAGTGAGAGCATGCCTGTCGGGACCCGAAAGATGGTGAACTATGCCTGAGCGGGGCGAAGCCAGAGGAAACTCTGGTGGAGGCCCGCAGCGATACTGACGTGCAAATCGTTCGTCTGACTTGGGTATAGGGGCGAAAGACTAATCGAACCGTCTAGTAGCTGGTTCCCTCCGAAGTTTCCCTCAGGATAGCTGGAGCTCGGAACGAGTTCTATCGGGTAAAGCCAATGATTAGAGGCATCGGGGGCGCAACGCCCTCGACCTATTCTCAAACTTTAAATAGGTAGGACGGCGCGGCTGCTTCGTTGAGCCGCGCCACGGAATCGAGAGCTCCAAGTGGGCCATTTTTGGTAAGCAGAACTGGCGATGCGGGATGAACCGGAAGCCGGGTTACGGTGCCCAACTGCGCGCTAACCTAGAACCCACAAAGGGTGTTGGTCGATTAAGACAGCAGGACGGTGGTCATGGAAGTCGAAATCCGCTAAGGAGTGTGTAACAACTCACCTGCCGAATCAACTAGCCCCGAAAATGGATGGCGCTTAAGCGCGCGACCTATACCCGGCCGTCGGGGCAAGAGCCAGGCCCCGATGAGTAGGAGGGCGCGGCGGTCGCTGCAAAACCCAGGGCGCGAGCCCGGGCGGAGCGGCCGTCGGTGCAGATCTTGGTGGTAGTAGCAAATATTCAAATGAGAACTTTGAAGGCCGAAGAGGGGAAAGGTTCCATGTGAACGGCACTTGCACATGGGTTAGTCGATCCTAAGAGACGGGGGAAGCCCGTCCGACAGCGCGTCCGCGCGCGAGCTTCGAAAGGGAATCGGGTTAAAATTCCCGAACCGGGACGCGGCGGCTGACGGCAACGTTAGGGAGTCCGGAGACGTCGGCGGGGGCCTCGGGAAGAGTTATCTTTTCTGTTTAACAGCCCGCCCACCCTGGAAACGACTCAGTCGGAGGTAGGGTCCAGCGGCTGGAAGAGCACCGCACGTCGCGCGGTGTCCGGTGCGCCCCCGGCGGCCCGTGAAAATCCGGAGGACCGAGTGCCATCCACGCCCGGTCGTACTCATAACCGCATCAGGTCTCCAAGGTGAACAGCCTCTGGTCGATGGAACAATGTAGGCAAGGGAAGTCGGCAAAATGGATCCGTAACCTCGGGAAAAGGATTGGCTCTGAGGGCTGGGCCCGGGGGTCCCAGTCCCGAACCCGTCGGCTGTCGGCGGACTGCTCGAGCTGCTCCCGCGGCAAGAGCGGGTCGCTGCGTGCCGGCCGGGGGACGGATTGGGAACGGCCCCTCCGGGGGCCTTCCCCGGGCGTCGAACAGTCGACTCAGAACTGGTACGGACAAGGGGAATCCGACTGTTTAATTAAAACAAAGCATTGCGATGGTCCCTGCGGATGCTAACGCAATGTGATTTCTGCCCAGTGCTCTGAATGTCAAAGTGAAGAAATTCAACCAAGCGCGGGTAAACGGCGGGAGTAACTATGACTCTCTTAAGGTAGCCAAATGCCTCGTCATCTAATTAGTGACGCGCATGAATGGATTAACGAGATTCCCACTGTCCCTGTCTACTATCCAGCGAAACCACAGCCAAGGGAACGGGCTTGGCAGAATCAGCGGGGAAAGAAGACCCTGTTGAGCTTGACTCTAGTCCGACTTTGTGAAATGACTTGAGAGGTGTAGTATAAGTGGGAGCCGGAAACGGCGATAGTGAAATACCACTACTTTTAACGTTATTTTACTTATTCCGTGAATCGGAGGCGGGGCATTGCCCCTCTTTTTGGACCAAAGGCCGCTTCGCGGTCGATCCGGGCGGAAGACATTGTCAGGTGGGGAGTTTGGCTGGGGCGGCACATCTGTTAAAAGATAACGCAGGTGTCCTAAGATGAGCTCAACGAGAACAGAAATCTCGTGTGGAACAAAAGGGTAAAAGCTCGTTTGATTCTGATTTCCAGTACGAATACGAACCGTGAAAGCGTGGCCTATCGATCCTTTAGACCTTCGGAATTTGAAGCTAGAGGTGTCAGAAAAGTTACCACAGGGATAACTGGCTTGTGGCAGCCAAGCGTTCATAGCGACGTTGCTTTTTGATCCTTCGATGTCGGCTCTTCCTATCATTGTGAAGCAGAATTCACCAAGTGTTGGATTGTTCACCCACCAATAGGGAACGTGAGCTGGGTTTAGACCGTCGTGAGACAGGTTAGTTTTACCCTACTGATGACGGTGTCGCGATGGTAATTCAACCTAGTACGAGAGGAACCGTTGATTCGCACAATTGGTCATCGCGCTTGGTTGAAAAGCCAGTGGCGCGAAGCTACCGTGCGCTGGATTATGACTGAACGCCTCTAAGTCAGAATCCGGGCCAGAAGCGATGCATGCGTCCGCCGCTCGTTTGCCGACCCTCAGTAGGGGCCATCCGGCCCCCAAAGGCACGTGTCGTTGGCTAAGCCCTCGCGGCAGACAAGCCGTGCGGGCAGCCTTGAAGTACAATTCCCACCGGGCGGCGGGCAGAATCCTTTGCAGACGACTTAAATACGCGACGGGGTATTGTAAGTGGCAGAGTGGCCTTGCTGCCACGATCCACTGAGATTCAGCCCTTTGTCGCTCCGATTCGTCCCTCCCTCCCAAGAAACTTTTTCCATCTCAATAGAATATCGAGCGGAGGCTGGGGTCTCGATTTCCGCTCGTAGAAAGAGGGCCAAGGAGAGAACCCCGGTATTGCATATGGCACGGGGGAAGCAAGAGATTAAGCACACCGCGGGGTGGAAAAAGCATCTGCGCACGGGCAGGTTAGAAAAACATACAAGCGATGCATCCCTTCAATGTCCCTCGGCATTGACCGCTTGTTCATTTTTTTTTCCGGCTTAATGCGTACGTTTCGAACCACTCGTACACCAAGGCTGCCGCGGCCACGCGTGCCTCACCGGTCGATGCGGTCTACTGCCTTCACAGGCGAGGCAGGAGCAGCATCGAATTCCTGCCCCGGCACCACGCACGAGGCCGGCGGTGCCTACCTCTCGTGCGCAAGCGAGACCGATGCCAGCGCGGCTGACTGGCCTGAATAGGAGAGGCCAGTGCTAGTCCGCGCACGCCACATGCCCCTTCAAAGTTTCCCCGCATCGCTCGAGAATTTTACGGCAGGCCCGTTGAACATCTCCATCGGGAGAGAGAATTTGCATCTCCGCCCCAAGCGTCGATGCTAGTCCGCGCACGCCACATGCCCCTTCAAAGTTTCCCCGCACCGCTCGAGAATTTTATGGCAGACCCGTTAAATATCTCCATCGGGAGAGAAAATTTGCATCTCCGCCCCCAACCGTCGAGAAAAGATCAACACTTTTACCACCCCGAGGGTTGTACACACAGCCTCCTGAAACAAAGGGGCGGCAAAAACGCGTTTCGCCTCCAGTCCACGCTCGGTCTTTCGGAACGGGTTCAACGGCAGTTGATTTTATACCGAGTTGCCAAATTCTCACGAGGGCTCTGTATTCTTTTTCGTTTGTCAAGTTCCCGGCAGTCTTAAATACGAGAAAGGCCATCTCGAAGGTCCCGGCATTCTCACGAAGGTTCTAAATATATATTTTCCTTTTTTCAAGAGCTAGGACTTCTTAAAAATGGAAAAAATTATCCCCCGTGTCCGGAAAGTCCCGGTAATGCATGTAGATTTCCCCTGAACCATGCTGGTCAGAATTTCTGCAAAAAAATGAAGGGTCTACGTATCTTCTGTCCATTTTTCCCGGACTCCCTAAAAATGGGAAAAATACCCCCCATGTTCGGAAAGTCCCGATATTACATGTAAATATCCCTTAAATCACACCCGAACTGTGGTCACAATTTCAGC--AAAAAAAAAAATGAAGTCTTTACATAT

Euphorbia bicompacta var. bicompacta 1 TTTTTTCAAGTTTTCCGGGCCTTCCTCAAAATGGAAAAAGATATCGCCCATGTCCGAAAAGTCCCGGTATTACATGTAAATGTCCCTTTGAAAATGTTCGAACGGTGGTCGAAATTTCAACAAAAAGTGAAGGCTTTACATTTTTTTTTCCATTTTTTCCGAAACCCTTAAATATGGAAAAAATAACCCCCATGTCCGGAAAGCCCCGGTATTACATGTAAATGTCCCCTAAATAATGCTCGAACCGTGGTAAAAATTTCAGCTAAAAATGAAGGCTTTACATATTTTTTGCCATTTTCCCCGTAATTCCTAAAAAAGGAAAAAATACCCCCGTCGGGCGGATTGGCCCGATATTACACGTCAATTTCCCCCAAATCATGCTCGAACTGGGGTCGGAATTTCGGCAAAAAAGGAAGGCTCTGCATATTTTTTTCCATTTTTCCCGGAATTCTTAAAAATGGGAAAAATATCCCCCACGTCCGGAAACTCCCGATATCAAGTGTAAATGTCCCTCAAATCATGCTCGAACCGTGGTAAAAATTTCAGCGAAAAATGAAGGCGTTGCATATTTTTTTCCATTTTTCCAGTGCCCGGAATTCACAAAAATGGAAAAAATAGCCCCGTTGTCCGGGAAGTCCCGGGATTGCATGTAAATTTCCCTTAAATCATGCTCGAACTATGGTAAAAATTTCAGAAAAAATCTCCAAGTATAGAGCAGTTTTTGGGGGGGTGTGGCTCCTGGAACAAATCGATGTCCGTTCCTCCCAGAGCTGGATTTGTCATAAGAAATACTATAGGGGCTGCGCCAGCTCTCAACCTTGGCCCGCACTGGGCTGCGGGCCCGTGTTGGGTGCCTCCCGCATGCTCTCCCGCATTCAAGCGCATCACTCGGTCTCCCAGAAAGTCCCGACCCGCCGCCCCACCGAACGGAAGAATATCGTGGAAAAGCTAAGCCCAAAAGCACCACACGCGCGCGCGCGACGGCGCGGCGCGTTCGCGATTTAGGCGATTTAGGCACTTGGCACTTAGCACGTAGCACGGAGCGCGAGGCGCGAGGCCAATCGGCGGAACATAGCAGAAAATATCCCGCAAAATTTATAAACGAGTAACGACACGGCATGAAAAAGAAGGTAGCTAGCAAAAATTTTAAAAAAAAGAAAGGAACATGAAGGCCAACCCGAAGTTCAGGCAAGAGTGCAGGGCGGCCTGGCCCGCCTTCGTGTGCGGGAGCAGGCGGGACAGCGTGCAGCGCTGTCCCGCATTCCTGCCTGTGCCTGGCGAGGCTAGGCAGGACGGCCGGCGTGCTGGCCCGCCCGCGTGTGCAGGAGGGCCTGCCAGTGGGAGGGCTGGCCCGCCTGGGCAGGGCTGGCAAGCTGCCCTGGCTCGCCTGAGTGCATGGCGGGGGAGGGCAAGCTGGCCAGCACGCAGCCCTGCCTGGCTCGCCAGCCAGCACCACGCAGGCAGGCCGCCAACCAGAGGTCAGCATAGGCATGCCAGCACGCCCAGCCGAGCTCTGCCGGCGGTGCTCACCCAGTCCGCGCAAAGGAGGCTAGGTCAGCGTGCTGTCTCGCCTGTCCGAGGCTCAGGATGGCTTCGCTGGGCCCGATCCAAATTTCGTTTGCAAAACCTTCGCACAGATAATAAGTTTTCCCGCACCATATTTTTTATACTGACTTTTACCATTTATTAGGAATTTTGTACCGAGTTCGTGATGCATTTGTTGAGTTTACATATTTTTTTGTATTTTTACGATTCTCGGACTTCGTAAATATGGAAAAAATACCTCCCGTGGGTAAAAGGTTACAATATTATATGGAAAAATCCCTTAATTCATGTTCTATCTACAGTCAAAATTTCAGGAAAAAACTCGAAGGTTTGACCGGTTTTTAAGGGGGTGTGGCTCCTGGAACAAATCGATGTCTGTTCCTCCCAGAGCTGGAAATGCTATAAGGACTACTATAGGGGGGTACACCTGCTCTCAACCCAGGCCACCACTGGGGCTGCGGGCCCGTGTTGGGTGCTTGCCGCATGCTCACCCCCTATCATGCGGGGAATTCAGCCACTAAGAACGTCCCGACCCGCCGCCCCTCCTCCGGCCGCCGCCCGCCGCCCCGCGCGGTGGCCGGAAAATCCAAAATTCTTAGAACGCTGAATTCCGCACCCCGAGAGCCCCTTTTCCCCCTGCCATTGTCGGAACCCGTTCGAATTGGGGGTAAAGTGGGTTTTCGGAGGCAACAACGTGCCCCGCGGCGCTGTCCCGATCGCTAGTGTGTGCCTACTCGTAGTTTTGTCTTGCATCGGATAGCTGATTGAGCTGCTCTCAGTGTGTATGGTGCTCGACTTCAAGCTGCTTTGGAAGCACGCCTAGAGTGAGGGGTAGCCGCTTGCTGCGCACGGTATCGGACGAGGGAAAAAGGAATTTCGGAAAAAAACGTTTCCGTGCCGTGCGCGATGTTGCTACATCCGTAGTTGCTTGCTGCGTGCGGTGTGGGGCGATGTAAAAATTGGAATTTGAAAATAAATTGTTTTCACGGAGTGCGCTTCGGCGTTGCCCCGATCGATAGTGCGTGTTGCTCGGTGGATTTGTTTGGCATTGGATAGCTGATCGAGTTGCTCTCGGTGTGAAGGTGCTGGACGTTGGTCCACTCGGCAGCCCGCCTTGAGTGAGGGGCAGTTGGTTGATGCGTGCGGTGCCATGTGGCGAAAAAATGGATTTCACTAAAAATGTCTTTCTTGCTGTGCGCTCAGGCGCCGTCCCGATCGGTAGTGTGCGTTATGTCCGGTGGTTCTGTTTGGCATCTGATAGCTGATCGAGTCGCTCTCAGTGTGTAAGGTGCTGGGTTTTGGACTATTTGGCTTGCCTGCCTATCGTGAGGAGCGGTTGCTTGGTGCGTACAGTTCCGGACGGCGAAAAATTGAATTCCAATAAAATATTATTCTCTCAACACGTGCTACTTTATCATGCGTTAAGGAATGTTCTCTCGCACACAGCGGTTCGGGCGATGTCTCTACTCGACGTTTCGGCACTGCTTGATTCGTTCTCGAAGACAGCAGTGCAGTTCGGGGGGTGGGGATGTTGCTTAATATACGCGGCGGTGCATGAGTGGTAAATAGGCCATTGGGGTTGGCAGGCTCTGTGCTAGCGCATCGAACTGTCGTACCTTGAGGCCACTCAGTGGTGTCCCGGAGGCGTATTGCTATGTCGGGCGGGGATGGTTTCTGTGTTGCATACCCGCGCAGTGGAATGGAATTTTGTTGCCAAGAAACATTCGTCCCGTGCCCTTTTAGGGGCGTCGGATGAACCATGCAGCAGCTCTCGTGTGCCGGGCATGCCTTTTTGGCTTCTCTGGCACATGTGAAGGTGCTCGTGCTCTCGGATGCGGAATGCTTTTGCGAGAGGAGGGATTGAGTTTCCTTTATGTGTTCTCGCTGTCCCTACATAAGAACCACCGTCCTTTCCGCACAGTGGCCTTGGTTGCTGCGGTGTACTATGTCTGCTTGCGGGTTAGGACGGCATGGAGGAATGCTACCTGGTTGATCCTGCCAGTAGTCATATGCTTGTCTCAAAGATTAAGCCATGCATGTGTAAGTATGAACTAATTCAGACTGTGAAACTGCGAATGGCTCATTAAATCAGTTATAGTTTGTTTGATGGTACCTGCTACTCGGATAACCGTAGTAATTCTAGAGCTAATACGTGCAACAAACCCCGACTTCTGGAAGGGATGCATTTATTAGATAAAAGGTCGACGCGGGCTCTGCCCGTTGCTCTGATGATTCATGATAACTCGACGGATCGCACGGCCATCGTGCTGGCGACGCATCATTCAAATTTCTGCCCTATCAACTTTCGATGGTAGGATAGAGGCCTACCATGGTGGTGACGGGTGACGGAGAATTAGGGTTCGATTCCGGAGAGGGAGCCTGAGAAACGGCTACCACATCCAAGGAAGGCAGCAGGCGCGCAAATTACCCAATCCTGACACGGGGAGGTAGTGACAATAAATAACAATACCGGGCTCTTCGAGTCTGGTAATTGGAATGAGTACAATCTAAATCCCTTAACGAGGATCCATTGGAGGGCAAGTCTGGTGCCAGCAGCCGCGGTAATTCCAGCTCCAATAGCGTATATTTAAGTTGTTGCAGTTAAAAAGCTCGTAGTTGGACCTTGGGTTGGGTCGACCGGTCCGCCTTACGGTGTGCACCTGTCGGCTCGTCCCTTCTGCCGGCGATGCGCTCCTGGCCTTAACTGGCCGGGTCGTGCCTCCGGCGCTGTTACTTTGAAGAAATTAGAGTGCTCAAAGCAAGCCTACGCTCTGTATACATTAGCATGGGATAACATCATAGGATTTCGGTCCTATTCTGTTGGCCTTCGGGATCGGAGTAATGATTAACAGGGACAGTCGGGGGCATTCGTATTTCATAGTCAGAGGTGAAATTCTTGGATTTATGAAAGACGAACAACTGCGAAAGCATTTGCCAAGGATGTTTTCATTAATCAAGAACGAAAGTTGGGGGCTCGAAGACGATCAGATACCGTCCTAGTCTCAACCATAAACGATGCCGACCAGGGATCGGCGGATGTTGCTTTTAGGACTCCGCCGGCACCTTATGAGAAATCAAAGTCTTTGGGTTCCGGGGGGAGTATGGTCGCAAGGCTGAAACTTAAAGGAATTGACGGAAGGGCACCACCAGGAGTGGAGCCTGCGGCTTAATTTGACTCAACACGGGGAAACTTACCAGGTCCAGACATAGTAAGGATTGACAGACTGAGAGCTCTTTCTTGATTCTATGGGTGGTGGTGCATGGCCGTTCTTAGTTGGTGGAGCGATTTGTCTGGTTAATTCCGTTAACGAACGAGACCTCAGCCTGCTAACTAGCTATGCGGAGGTATCCCTCCGCGGCCAGCTTCTTAGAGGGACTATGGCCTTCTAGGCCAAGGAAGTTTGAGGCAATAACAGGTCTGTGATGCCCTTAGATGTTCTGGGCCGCACGCGCGCTACACTGATGTATTCAACGAGTCTATAGCCTTGGCCGACAGGCCCGGGTAATCTTTGAAATTTCATCGTGATGGGGATAGATCATTGCAATTGTTGGTCTTCAACGAGGAATTCCTAGTAAGCGCGAGTCATCAGCTCGCGTTGACTACGTCCCTGCCCTTTGTACACACCGCCCGTCGCTCCTACCGATTGAATGGTCCGGTGAAGTGTTCGGATCGCGGCGACGTGGGCGGTTCGCCGCCGGCGACGTCGCGAGAAGTCCACTGAACCTTATCATTTAGAGGAAGGAGAAGTCGTAACAAGGTTTCCGTAGGTGAACCTGCGGAAGGATCATTGTCGAAACCTGCCAGCAGAATGACCCGCGAACGTGTTTATAAATCGAGGGGCCGCTGCAGGATTCATCCAGCGATGGCACCTCACTAGGGCCCTGGCAGGGGATGCGGTGCGGTGGGATCCACCGTTCCCTGCGATCTCCTGTTTGCGGCCTATTAACAAAACCCCGGCGCCGTACGCGCCAAGGAATTGTAAAAAAAGATTGTGCAGCCCGATCGCACTGGCAACGGTGTGGCGGGTTTCACTGCGCTTTGAGAACCAAAATGACTCTCGGCAACGGATATCTCGGCTCTCGCATCGATGAAGAACGCAGCGAAATGCGATACTTGGTGTGAATTGCAGGATCCCGCGAACCATCGAGTCTTTGAACGCAAGTTGCGCCCGAAGCCTTTCGGCCGAGGGCACGTCTGCCTGGGTGTCACTCAAACGTCGCTCCAAACCCCTTCCATCGGGAGGGGTATGCGGGGCGGATGCTGGCCTCCCGTGTGCGTATCGCTCGCGGTTGGCCGAAATTCCTAGTCCTCGGCACGACGCCACGGAATCGGTGGTTGCAAGACCCTCGGAGAAAGCCTTGTGCGCTTGTAAGCCCTTCCGGACCATGAGACCCCAGAGCGTACCTAGCACTGCGACCCCAGGTCAGGCGGGATTACCCGCTGAGTTTAAGCATATCAATAAGCGGAGGAAAAGAAACTTACCAGGATTCCCCTAGTAACGGCGAGCGAACCGGGAAGAGCCCAGCTTGAGAATCGTGCGCCTGCGGCGTTCGAATTGTAGTCTGGAGAAGCGTCCTCAGCGGCGGACCGGGCCCAAGTCCCCTGGAAGGGGGCGCCGGAGAGGGTGAGAGCCCCGTCGTGCCCGGACCCTGTCGCACCACGAGGCGCTGTCTACGAGTCGGGTTGTTTGGGAATGCAGCCCAAATCGGGCGGTAAATTCCGTCCAAGGCTAAATATGGGCGAGAGACCGATAGCGAACAAGTACCGCGAGGGAAAGATGAAAAGGACTTTGAAAAGAGAGTCAAAGAGTGCTTGAAATTGTCGGGAGGGAAGCGGATGGGGGCCGGCGATGCGCCCCGGTCGGATGTGGAACGGTGACAAGCCGGTCCGCCGATCGGCTCGGGGCGCGGACCGATACGGATTGAGGCGGCGGCGTAAGCCCAGGAATTTGAAACGCCTGTGGAGATGCCGTCGCAGCAATCGTGGAAAGCAGCACGCGCCGTCTCGGCGTGCCTCGGCACCTGCGTGCTACTGGTGTCGGCCAGCGGGCTCCCCATTCGGCCCGTCTTGAAACACGGACCAAGGAGTCTGACATGTGTGCGAGTCAACGGGCGAGTAAACCCGTAAGGCGCAAGGAAGCTGACTGGCGGGATCCCCTAGAGGGTTGCACCGCCGACCGACCTTGATCTTCTGAGAAGGGTTCGAGTGAGAGCATGCCTGTCGGGACCCGAAAGATGGTGAACTATGCCTGAGCGGGGCGAAGCCAGAGGAAACTCTGGTGGAGGCCCGCAGCGATACTGACGTGCAAATCGTTCGTCTGACTTGGGTATAGGGGCGAAAGACTAATCGAACCGTCTAGTAGCTGGTTCCCTCCGAAGTTTCCCTCAGGATAGCTGGAGCTCGGAACGAGTTCTATCGGGTAAAGCCAATGATTAGAGGCATCGGGGGCGCAACGCCCTCGACCTATTCTCAAACTTTAAATAGGTAGGACGGCGCGGCTGCTTCGTTGAGCCGCGCCACGGAATCGAGAGCTCCAAGTGGGCCATTTTTGGTAAGCAGAACTGGCGATGCGGGATGAACCGGAAGCCGGGTTACGGTGCCCAACTGCGCGCTAACCTAGAACCCACAAAGGGTGTTGGTCGATTAAGACAGCAGGACGGTGGTCATGGAAGTCGAAATCCGCTAAGGAGTGTGTAACAACTCACCTGCCGAATCAACTAGCCCCGAAAATGGATGGCGCTTAAGCGCGCGACCTATACCCGGCCGTCGGGGCAAGAGCCAGGCCCCGATGAGTAGGAGGGCGCGGCGGTCGCTGCAAAACCCAGGGCGCGAGCCCGGGCGGAGCGGCCGTCGGTGCAGATCTTGGTGGTAGTAGCAAATATTCAAATGAGAACTTTGAAGGCCGAAGAGGGGAAAGGTTCCATGTGAACGGCACTTGCACATGGGTTAGTCGATCCTAAGAGACGGGGGAAGCCCGTCCGACAGCGCGTCCGCGCGCGAGCTTCGAAAGGGAATCGGGTTAAAATTCCCGAACCGGGACGCGGCGGCTGACGGCAACGTTAGGGAGTCCGGAGACGTCGGCGGGGGCCTCGGGAAGAGTTATCTTTTCTGTTTAACAGCCCGCCCACCCTGGAAACGACTCAGTCGGAGGTAGGGTCCAGCGGCTGGAAGAGCACCGCACGTCGCGCGGTGTCCGGTGCGCCCCCGGCGGCCCGTGAAAATCCGGAGGACCGAGTGCCATCCACGCCCGGTCGTACTCATAACCGCATCAGGTCTCCAAGGTGAACAGCCTCTGGTCGATGGAACAATGTAGGCAAGGGAAGTCGGCAAAATGGATCCGTAACCTCGGGAAAAGGATTGGCTCTGAGGGCTGGGCCCGGGGGTCCCAGTCCCGAACCCGTCGGCTGTCGGCGGACTGCTCGAGCTGCTCCCGCGGCAAGAGCGGGTCGCTGCGTGCCGGCCGGGGGACGGATTGGGAACGGCCCCTCCGGGGGCCTTCCCCGGGCGTCGAACAGTCGACTCAGAACTGGTACGGACAAGGGGAATCCGACTGTTTAATTAAAACAAAGCATTGCGATGGTCCCTGCGGATGCTAACGCAATGTGATTTCTGCCCAGTGCTCTGAATGTCAAAGTGAAGAAATTCAACCAAGCGCGGGTAAACGGCGGGAGTAACTATGACTCTCTTAAGGTAGCCAAATGCCTCGTCATCTAATTAGTGACGCGCATGAATGGATTAACGAGATTCCCACTGTCCCTGTCTACTATCCAGCGAAACCACAGCCAAGGGAACGGGCTTGGCAGAATCAGCGGGGAAAGAAGACCCTGTTGAGCTTGACTCTAGTCCGACTTTGTGAAATGACTTGAGAGGTGTAGTATAAGTGGGAGCCGGAAACGGCGATAGTGAAATACCACTACTTTTAACGTTATTTTACTTATTCCGTGAATCGGAGGCGGGGCATTGCCCCTCTTTTTGGACCAAAGGCCGCTTCGCGGTCGATCCGGGCGGAAGACATTGTCAGGTGGGGAGTTTGGCTGGGGCGGCACATCTGTTAAAAGATAACGCAGGTGTCCTAAGATGAGCTCAACGAGAACAGAAATCTCGTGTGGAACAAAAGGGTAAAAGCTCGTTTGATTCTGATTTCCAGTACGAATACGAACCGTGAAAGCGTGGCCTATCGATCCTTTAGACCTTCGGAATTTGAAGCTAGAGGTGTCAGAAAAGTTACCACAGGGATAACTGGCTTGTGGCAGCCAAGCGTTCATAGCGACGTTGCTTTTTGATCCTTCGATGTCGGCTCTTCCTATCATTGTGAAGCAGAATTCACCAAGTGTTGGATTGTTCACCCACCAATAGGGAACGTGAGCTGGGTTTAGACCGTCGTGAGACAGGTTAGTTTTACCCTACTGATGACGGTGTCGCGATGGTAATTCAACCTAGTACGAGAGGAACCGTTGATTCGCACAATTGGTCATCGCGCTTGGTTGAAAAGCCAGTGGCGCGAAGCTACCGTGCGCTGGATTATGACTGAACGCCTCTAAGTCAGAATCCGGGCCAGAAGCGATGCATGCGTCCGCCGCTCGTTTGCCGACCCTCAGTAGGGGCCATCCGGCCCCCAAAGGCACGTGTCGTTGGCTAAGCCCTCGCGGCAGACAAGCCGTGCGGGCAGCCTTGAAGTACAATTCCCACCGGGCGGCGGGCAGAATCCTTTGCAGACGACTTAAATACGCGACGGGGTATTGTAAGTGGCAGAGTGGCCTTGCTGCCACGATCCACTGAGATTCAGCCCTTTGTCGCTCCGATTCGTCCCTCCCTCCCAAGAAACTTTTTCCATCTCAATAGAATATCGAGCGGAGGCTGGGGTCTCGATTTCCGCTCGTAGAAAGAGGGCCAAGGAGAGAACCCCGGTATTGCATATGGCACGGGGGAAGCAAGAGATTAAGCACACCGCGGGGTGGAAAAAGCATCTGCGCACGGGCAGGTTAGAAAAACATACAAGCGATGCATCCCTTCAATGTCCCTCGGCGTTGACCGCTTGTTCATTTTTTTTTCCGGCTTAATGCGTACGTTTCGAACCACTCGTACACCAAGGCTGCCGCGGCCACGCGTGCCTCACCGGTCGATGCGGTCTACTGCCTTCACAGGCGAGGCAGGAGCAGCATCGAATTCCTGCCCCGGCACCACGCACGAGGCCGGCGGTGCCTGCCTCTCGTGCGCAAGCGAGACCGATGCCAGCGCGGCTGACTGGCCTGAATAGGAGAGGCCAGTGCTAGTCCGCGCACGCCACATGCCCCTTCAAAGTTTCCCCGCATCGCTCGAGAATTTTACGGCAGGCCCGTTAAACATCTCCATCGGGAGAGAGAATTTGCATCTCCGCCCCAAGCGTCGATGCTAGTCCGCGCACGCCACATGCCCCTTCAAAGTTTCCCCGCACCGCTCGAGAATTTTATGGCAGACCCGTTAAATATCTCCACCGGGAGAGAAAATTTGCATCTCCGCCCCCAACCGTCGAGAAAAGATTAACACTTTTACCACCCCGAGGGTTGTACACACAGCCTCCTGAAACAAAGGGGCGGCAAAAACGCGTTTCGCCTCCAGTCCACGCTCGGTCTTTCGGAACGGGTTCAACGGCAGTTGATTTTATACCGGGTTGCCAAATTCTCACGAGGGCTCTGTATTCTTTTTCGTTTGTCAAGTTCCCGGCAGTCTTAAATACGAGAAAGGCCATCTCGAAGGTCCCGGCATTCTCACGAAGGTTCTAAATATATATTTTCCTTTTTTCAAGAGCTAGGACTTCTTAAAAATGGAAAAAATTATCCCCCGTGTCCGGAACGTCCCGGTAATGCATGTAGATTTCCCTTGAACCATGCTGGTCAGAATTTCTGCAAAAAAATGAAGGGTCTACGTATCTTCCGT-----------------------------------------------------------------------------------------CCCGAACTGTGGTCACAATTTCAGCAAAAAAAAAAAAATGAAGTCTTTACATAC

Euphorbia bicompacta var. bicompacta 2 TTTTTTCAAGTTTTCCGGGCCTTCCTCAAAATGGAAAAAGATATCGCCCATGTCCGAAAAGTCCCGGTATTACATGTAAATGTCCCTTTGAAAATGTTCGAACGGTGGTCGAAATTTCAACAAAAAGTGAAGGCTTTACATTTTTTTTTCCATTTTTTCCGAAACCCTTAAATATGGAAAAAATAACCCCCATGTCCGGAAAGCCCCGGTATTACATGTAAATGTCCCCTAAATAATGCTCGAACCGTGGTAAAAATTTCAGCTAAAAATGAAGGCTTTACATATTTTTTGCCATTTTCCCCGTAATTCCTAAAAAAGGAAAAAATACCCCCGTCGGGCGGATTGGCCCGATATTACACGTCAATTTCCCCCAAATCATGCTCGAACTGGGGTCGGAATTTCGGCAAAAAAGGAAGGCTCTGCATATTTTTTTCCATTTTTCCCGGAATTCTTAAAAATGGGAAAAATATCCCCCACGTCCGGAAACTCCCGATATCAAGTGTAAATGTCCCTCAAATCATGCTCGAACCGTGGTAAAAATTTCAGCGAAAAATGAAGGCGTTGCATATTTTTTTCCATTTTTCCAGTGCCCGGAATTCACAAAAATGGAAAAAATAGCCCCGTTGTCCGGGAAGTCCCGGGATTGCATGTAAATTTCCCTTAAATCATGCTCGAACTATGGTAAAAATTTCAGAAAAAATCTCCAAGTATAGAGCAGTTTTTGGGGGGGTGTGGCTCCTGGAACAAATCGATGTCCGTTCCTCCCAGAGCTGGATTTGTCATAAGAAATACTATAGGGGCTGCGCCAGCTCTCAACCTTGGCCCGCACTGGGCTGCGGGCCCGTGTTGGGTGCCTCCCGCATGCTCTCCCGCATTCAAGCGCATCACTCGGTCTCCCAGAAAGTCCCGACCCGCCGCCCCACCGAACGGAAGAATATCGTGGAAAAGCTAAGCCCAAAAGCACCACACGCGCGCGCGCGACGGCGCGGCGCGTTCGCGATTTAGGCGATTTAGGCACTTGGCACTTAGCACGTAGCACGGAGCGCGAGGCGCGAGGCCAATCGGCGGAACATAGCAGAAAATATCCCGCAAAATTTATAAACGAGTAACGACACGGCATGAAAAAGAAGGTAGCTAGCAAAAATTTTAAAAAAAAGAAAGGAACATGAAGGCCAACCCGAAGTTCAGGCAAGAGTGCAGGGCGGCCTGGCCCGCCTTCGTGTGCGGGAGCAGGCGGGACAGCGTGCAGCGCTGTCCCGCATTCCTGCCTGTGCCTGGCGAGGCTAGGCAGGACGGCCGGCGTGCTGGCCCGCCCGCGTGTGCAGGAGGGCCTGCCAGTGGGAGGGCTGGCCCGCCTGGGCAGGGCTGGCAAGCTGCCCTGGCTCGCCTGAGTGCATGGCGGGGGAGGGCAAGCTGGCCAGCACGCAGCCCTGCCTGGCTCGCCAGCCAGCACCACGCAGGCAGGCCGCCAACCAGAGGTCAGCATAGGCATGCCAGCACGCCCAGCCGAGCTCTGCCGGCGGTGCTCACCCAGTCCGCGCAAAGGAGGCTAGGTCAGCGTGCTGTCTCGCCTGTCCGAGGCTCAGGATGGCTTCGCTGGGCCCGATCCAAATTTCGTTTGCAAAACCTTCGCACAGATAATAAGTTTTCCCGCACCATATTTTTTATACTGACTTTTACCATTTATTAGGAATTTTGTACCGAGTTCGTGATGCATTTGTTGAGTTTACATATTTTTTTGTATTTTTACGATTCTCGGACTTCGTAAATATGGAAAAAATACCTCCCGTGGGTAAAAGGTTACAATATTATATGGAAAAATCCCTTAATTCATGTTCTATCTACAGTCAAAATTTCAGGAAAAAACTCGAAGGTTTGACCGGTTTTTAAGGGGGTGTGGCTCCTGGAACAAATCGATGTCTGTTCCTCCCAGAGCTGGAAATGCTATAAGGACTACTATAGGGGGGTACACCTGCTCTCAACCCAGGCCACCACTGGGGCTGCGGGCCCGTGTTGGGTGCTTGCCGCATGCTCACCCCCTATCATGCGGGGAATTCAGCCACTAAGAACGTCCCGACCCGCCGCCCCTCCTCCGGCCGCCGCCCGCCGCCCCGCGCGGTGGCCGGAAAATCCAAAATTCTTAGAACGCTGAATTCCGCACCCCGAGAGCCCCTTTTCCCCCTGCCATTGTCGGAACCCGTTCGAATTGGGGGTAAAGTGGGTTTTCGGAGGCAACAACGTGCCCCGCGGCGCTGTCCCGATCGCTAGTGTGTGCCTACTCGTAGTTTTGTCTTGCATCGGATAGCTGATTGAGCTGCTCTCAGTGTGTATGGTGCTCGACTTCAAGCTGCTTTGGAAGCACGCCTAGAGTGAGGGGTAGCCGCTTGCTGCGCACGGTATCGGACGAGGGAAAAAGGAATTTCGGAAAAAAACGTTTCCGTGCCGTGCGCGATGTTGCTACATCCGTAGTTGCTTGCTGCGTGCGGTGTGGGGCGATGTAAAAATTGGAATTTGAAAATAAATTGTTTTCACGGAGTGCGCTTCGGCGTTGCCCCGATCGATAGTGCGTGTTGCTCGGTGGATTTGTTTGGCATTGGATAGCTGATCGAGTTGCTCTCGGTGTGAAGGTGCTGGACGTTGGTCCACTCGGCAGCCCGCCTTGAGTGAGGGGCAGTTGGTTGATGCGTGCGGTGCCATGTGGCGAAAAAATGGATTTCACTAAAAATGTCTTTCTTGCTGTGCGCTCAGGCGCCGTCCCGATCGGTAGTGTGCGTTATGTCCGGTGGTTCTGTTTGGCATCTGATAGCTGATCGAGTCGCTCTCAGTGTGTAAGGTGCTGGGTTTTGGACTATTTGGCTTGCCTGCCTATCGTGAGGAGCGGTTGCTTGGTGCGTACAGTTCCGGACGGCGAAAAATTGAATTCCAATAAAATATTATTCTCTCAACACGTGCTACTTTATCATGCGTTAAGGAATGTTCTCTCGCACACAGCGGTTCGGGCGATGTCTCTACTCGACGTTTCGGCACTGCTTGATTCGTTCTCGAAGACAGCAGTGCAGTTCGGGGGGTGGGGATGTTGCTTAATATACGCGGCGGTGCATGAGTGGTAAATAGGCCATTGGGGTTGGCAGGCTCTGTGCTAGCGCATCGAACTGTCGTACCTTGAGGCCACTCAGTGGTGTCCCGGAGGCGTATTGCTATGTCGGGCGGGGATGGTTTCTGTGTTGCATACCCGCGCAGTGGAATGGAATTTTGTTGCCAAGAAACATTCGTCCCGTGCCCTTTTAGGGGCGTCGGATGAACCATGCAGCAGCTCTCGTGTGCCGGGCATGCCTTTTTGGCTTCTCTGGCACATGTGAAGGTGCTCGTGCTCTCGGATGCGGAATGCTTTTGCGAGAGGAGGGATTGAGTTTCCTTTATGTGTTCTCGCTGTCCCTACATAAGAACCACCGTCCTTTCCGCACAGTGGCCTTGGTTGCTGCGGTGTACTATGTCTGCTTGCGGGTTAGGACGGCATGGAGGAATGCTACCTGGTTGATCCTGCCAGTAGTCATATGCTTGTCTCAAAGATTAAGCCATGCATGTGTAAGTATGAACTAATTCAGACTGTGAAACTGCGAATGGCTCATTAAATCAGTTATAGTTTGTTTGATGGTACCTGCTACTCGGATAACCGTAGTAATTCTAGAGCTAATACGTGCAACAAACCCCGACTTCTGGAAGGGATGCATTTATTAGATAAAAGGTCGACGCGGGCTCTGCCCGTTGCTCTGATGATTCATGATAACTCGACGGATCGCACGGCCATCGTGCTGGCGACGCATCATTCAAATTTCTGCCCTATCAACTTTCGATGGTAGGATAGAGGCCTACCATGGTGGTGACGGGTGACGGAGAATTAGGGTTCGATTCCGGAGAGGGAGCCTGAGAAACGGCTACCACATCCAAGGAAGGCAGCAGGCGCGCAAATTACCCAATCCTGACACGGGGAGGTAGTGACAATAAATAACAATACCGGGCTCTTCGAGTCTGGTAATTGGAATGAGTACAATCTAAATCCCTTAACGAGGATCCATTGGAGGGCAAGTCTGGTGCCAGCAGCCGCGGTAATTCCAGCTCCAATAGCGTATATTTAAGTTGTTGCAGTTAAAAAGCTCGTAGTTGGACCTTGGGTTGGGTCGACCGGTCCGCCTTACGGTGTGCACCTGTCGGCTCGTCCCTTCTGCCGGCGATGCGCTCCTGGCCTTAACTGGCCGGGTCGTGCCTCCGGCGCTGTTACTTTGAAGAAATTAGAGTGCTCAAAGCAAGCCTACGCTCTGTATACATTAGCATGGGATAACATCATAGGATTTCGGTCCTATTCTGTTGGCCTTCGGGATCGGAGTAATGATTAACAGGGACAGTCGGGGGCATTCGTATTTCATAGTCAGAGGTGAAATTCTTGGATTTATGAAAGACGAACAACTGCGAAAGCATTTGCCAAGGATGTTTTCATTAATCAAGAACGAAAGTTGGGGGCTCGAAGACGATCAGATACCGTCCTAGTCTCAACCATAAACGATGCCGACCAGGGATCGGCGGATGTTGCTTTTAGGACTCCGCCGGCACCTTATGAGAAATCAAAGTCTTTGGGTTCCGGGGGGAGTATGGTCGCAAGGCTGAAACTTAAAGGAATTGACGGAAGGGCACCACCAGGAGTGGAGCCTGCGGCTTAATTTGACTCAACACGGGGAAACTTACCAGGTCCAGACATAGTAAGGATTGACAGACTGAGAGCTCTTTCTTGATTCTATGGGTGGTGGTGCATGGCCGTTCTTAGTTGGTGGAGCGATTTGTCTGGTTAATTCCGTTAACGAACGAGACCTCAGCCTGCTAACTAGCTATGCGGAGGTATCCCTCCGCGGCCAGCTTCTTAGAGGGACTATGGCCTTCTAGGCCAAGGAAGTTTGAGGCAATAACAGGTCTGTGATGCCCTTAGATGTTCTGGGCCGCACGCGCGCTACACTGATGTATTCAACGAGTCTATAGCCTTGGCCGACAGGCCCGGGTAATCTTTGAAATTTCATCGTGATGGGGATAGATCATTGCAATTGTTGGTCTTCAACGAGGAATTCCTAGTAAGCGCGAGTCATCAGCTCGCGTTGACTACGTCCCTGCCCTTTGTACACACCGCCCGTCGCTCCTACCGATTGAATGGTCCGGTGAAGTGTTCGGATCGCGGCGACGTGGGCGGTTCGCCGCCGGCGACGTCGCGAGAAGTCCACTGAACCTTATCATTTAGAGGAAGGAGAAGTCGTAACAAGGTTTCCGTAGGTGAACCTGCGGAAGGATCATTGTCGAAACCTGCCAGCAGAATGACCCGCGAACGTGTTTATAAATCGAGGGGCCGCTGCAGGATTCATCCAGCGATGGCACCTCACTAGGGCCCTGGCAGGGGATGCGGTGCGGTGGGATCCACCGTTCCCTGCGATCTCCTGTTTGCGGCCTATTAACAAAACCCCGGCGCCGTACGCGCCAAGGAATTGTAAAAAAAGATTGTGCAGCCCGATCGCACTGGCAACGGTGTGGCGGGTTTCACTGCGCTTTGAGAACCAAAATGACTCTCGGCAACGGATATCTCGGCTCTCGCATCGATGAAGAACGCAGCGAAATGCGATACTTGGTGTGAATTGCAGGATCCCGCGAACCATCGAGTCTTTGAACGCAAGTTGCGCCCGAAGCCTTTCGGCCGAGGGCACGTCTGCCTGGGTGTCACTCAAACGTCGCTCCAAACCCCTTCCATCGGGAGGGGTATGCGGGGCGGATGCTGGCCTCCCGTGTGCGTATCGCTCGCGGTTGGCCGAAATTCCTAGTCCTCGGCACGACGCCACGGAATCGGTGGTTGCAAGACCCTCGGAGAAAGCCTTGTGCGCTTGTAAGCCCTTCCGGACCATGAGACCCCAGAGCGTACCTAGCACTGCGACCCCAGGTCAGGCGGGATTACCCGCTGAGTTTAAGCATATCAATAAGCGGAGGAAAAGAAACTTACCAGGATTCCCCTAGTAACGGCGAGCGAACCGGGAAGAGCCCAGCTTGAGAATCGTGCGCCTGCGGCGTTCGAATTGTAGTCTGGAGAAGCGTCCTCAGCGGCGGACCGGGCCCAAGTCCCCTGGAAGGGGGCGCCGGAGAGGGTGAGAGCCCCGTCGTGCCCGGACCCTGTCGCACCACGAGGCGCTGTCTACGAGTCGGGTTGTTTGGGAATGCAGCCCAAATCGGGCGGTAAATTCCGTCCAAGGCTAAATATGGGCGAGAGACCGATAGCGAACAAGTACCGCGAGGGAAAGATGAAAAGGACTTTGAAAAGAGAGTCAAAGAGTGCTTGAAATTGTCGGGAGGGAAGCGGATGGGGGCCGGCGATGCGCCCCGGTCGGATGTGGAACGGTGACAAGCCGGTCCGCCGATCGGCTCGGGGCGCGGACCGATACGGATTGAGGCGGCGGCGTAAGCCCAGGAATTTGAAACGCCTGTGGAGATGCCGTCGCAGCAATCGTGGAAAGCAGCACGCGCCGTCTCGGCGTGCCTCGGCACCTGCGTGCTACTGGTGTCGGCCAGCGGGCTCCCCATTCGGCCCGTCTTGAAACACGGACCAAGGAGTCTGACATGTGTGCGAGTCAACGGGCGAGTAAACCCGTAAGGCGCAAGGAAGCTGACTGGCGGGATCCCCTAGAGGGTTGCACCGCCGACCGACCTTGATCTTCTGAGAAGGGTTCGAGTGAGAGCATGCCTGTCGGGACCCGAAAGATGGTGAACTATGCCTGAGCGGGGCGAAGCCAGAGGAAACTCTGGTGGAGGCCCGCAGCGATACTGACGTGCAAATCGTTCGTCTGACTTGGGTATAGGGGCGAAAGACTAATCGAACCGTCTAGTAGCTGGTTCCCTCCGAAGTTTCCCTCAGGATAGCTGGAGCTCGGAACGAGTTCTATCGGGTAAAGCCAATGATTAGAGGCATCGGGGGCGCAACGCCCTCGACCTATTCTCAAACTTTAAATAGGTAGGACGGCGCGGCTGCTTCGTTGAGCCGCGCCACGGAATCGAGAGCTCCAAGTGGGCCATTTTTGGTAAGCAGAACTGGCGATGCGGGATGAACCGGAAGCCGGGTTACGGTGCCCAACTGCGCGCTAACCTAGAACCCACAAAGGGTGTTGGTCGATTAAGACAGCAGGACGGTGGTCATGGAAGTCGAAATCCGCTAAGGAGTGTGTAACAACTCACCTGCCGAATCAACTAGCCCCGAAAATGGATGGCGCTTAAGCGCGCGACCTATACCCGGCCGTCGGGGCAAGAGCCAGGCCCCGATGAGTAGGAGGGCGCGGCGGTCGCTGCAAAACCCAGGGCGCGAGCCCGGGCGGAGCGGCCGTCGGTGCAGATCTTGGTGGTAGTAGCAAATATTCAAATGAGAACTTTGAAGGCCGAAGAGGGGAAAGGTTCCATGTGAACGGCACTTGCACATGGGTTAGTCGATCCTAAGAGACGGGGGAAGCCCGTCCGACAGCGCGTCCGCGCGCGAGCTTCGAAAGGGAATCGGGTTAAAATTCCCGAACCGGGACGCGGCGGCTGACGGCAACGTTAGGGAGTCCGGAGACGTCGGCGGGGGCCTCGGGAAGAGTTATCTTTTCTGTTTAACAGCCCGCCCACCCTGGAAACGACTCAGTCGGAGGTAGGGTCCAGCGGCTGGAAGAGCACCGCACGTCGCGCGGTGTCCGGTGCGCCCCCGGCGGCCCGTGAAAATCCGGAGGACCGAGTGCCATCCACGCCCGGTCGTACTCATAACCGCATCAGGTCTCCAAGGTGAACAGCCTCTGGTCGATGGAACAATGTAGGCAAGGGAAGTCGGCAAAATGGATCCGTAACCTCGGGAAAAGGATTGGCTCTGAGGGCTGGGCCCGGGGGTCCCAGTCCCGAACCCGTCGGCTGTCGGCGGACTGCTCGAGCTGCTCCCGCGGCAAGAGCGGGTCGCTGCGTGCCGGCCGGGGGACGGATTGGGAACGGCCCCTCCGGGGGCCTTCCCCGGGCGTCGAACAGTCGACTCAGAACTGGTACGGACAAGGGGAATCCGACTGTTTAATTAAAACAAAGCATTGCGATGGTCCCTGCGGATGCTAACGCAATGTGATTTCTGCCCAGTGCTCTGAATGTCAAAGTGAAGAAATTCAACCAAGCGCGGGTAAACGGCGGGAGTAACTATGACTCTCTTAAGGTAGCCAAATGCCTCGTCATCTAATTAGTGACGCGCATGAATGGATTAACGAGATTCCCACTGTCCCTGTCTACTATCCAGCGAAACCACAGCCAAGGGAACGGGCTTGGCAGAATCAGCGGGGAAAGAAGACCCTGTTGAGCTTGACTCTAGTCCGACTTTGTGAAATGACTTGAGAGGTGTAGTATAAGTGGGAGCCGGAAACGGCGATAGTGAAATACCACTACTTTTAACGTTATTTTACTTATTCCGTGAATCGGAGGCGGGGCATTGCCCCTCTTTTTGGACCAAAGGCCGCTTCGCGGTCGATCCGGGCGGAAGACATTGTCAGGTGGGGAGTTTGGCTGGGGCGGCACATCTGTTAAAAGATAACGCAGGTGTCCTAAGATGAGCTCAACGAGAACAGAAATCTCGTGTGGAACAAAAGGGTAAAAGCTCGTTTGATTCTGATTTCCAGTACGAATACGAACCGTGAAAGCGTGGCCTATCGATCCTTTAGACCTTCGGAATTTGAAGCTAGAGGTGTCAGAAAAGTTACCACAGGGATAACTGGCTTGTGGCAGCCAAGCGTTCATAGCGACGTTGCTTTTTGATCCTTCGATGTCGGCTCTTCCTATCATTGTGAAGCAGAATTCACCAAGTGTTGGATTGTTCACCCACCAATAGGGAACGTGAGCTGGGTTTAGACCGTCGTGAGACAGGTTAGTTTTACCCTACTGATGACGGTGTCGCGATGGTAATTCAACCTAGTACGAGAGGAACCGTTGATTCGCACAATTGGTCATCGCGCTTGGTTGAAAAGCCAGTGGCGCGAAGCTACCGTGCGCTGGATTATGACTGAACGCCTCTAAGTCAGAATCCGGGCCAGAAGCGATGCATGCGTCCGCCGCTCGTTTGCCGACCCTCAGTAGGGGCCATCCGGCCCCCAAAGGCACGTGTCGTTGGCTAAGCCCTCGCGGCAGACAAGCCGTGCGGGCAGCCTTGAAGTACAATTCCCACCGGGCGGCGGGCAGAATCCTTTGCAGACGACTTAAATACGCGACGGGGTATTGTAAGTGGCAGAGTGGCCTTGCTGCCACGATCCACTGAGATTCAGCCCTTTGTCGCTCCGATTCGTCCCTCCCTCCCAAGAAACTTTTTCCATCTCAATAGAATATCGAGCGGAGGCTGGGGTCTCGATTTCCGCTCGTAGAAAGAGGGCCAAGGAGAGAACCCCGGTATTGCATATGGCACGGGGGAAGCAAGAGATTAAGCACACCGCGGGGTGGAAAAAGCATCTGCGCACGGGCAGGTTAGAAAAACATACAAGCGATGCATCCCTTCAATGTCCCTCGGCGTTGACCGCTTGTTCATTTTTTTTTCCGGCTTAATGCGTACGTTTCGAACCACTCGTACACCAAGGCTGCCGCGGCCACGCGTGCCTCACCGGTCGATGCGGTCTACTGCCTTCACAGGCGAGGCAGGAGCAGCATCGAATTCCTGCCCCGGCACCACGCACGAGGCCGGCGGTGCCTGCCTCTCGTGCGCAAGCGAGACCGATGCCAGCGCGGCTGACTGGCCTGAATAGGAGAGGCCAGTGCTAGTCCGCGCACGCCACATGCCCCTTCAAAGTTTCCCCGCATCGCTCGAGAATTTTACGGCAGGCCCGTTAAACATCTCCATCGGGAGAGAGAATTTGCATCTCCGCCCCAAGCGTCGATGCTAGTCCGCGCACGCCACATGCCCCTTCAAAGTTTCCCCGCACCGCTCGAGAATTTTATGGCAGACCCGTTAAATATCTCCACCGGGAGAGAAAATTTGCATCTCCGCCCCCAACCGTCGAGAAAAGATTAACACTTTTACCACCCCGAGGGTTGTACACACAGCCTCCTGAAACAAAGGGGCGGCAAAAACGCGTTTCGCCTCCAGTCCACGCTCGGTCTTTCGGAACGGGTTCAACGGCAGTTGATTTTATACCGGGTTGCCAAATTCTCACGAGGGCTCTGTATTCTTTTTCGTTTGTCAAGTTCCCGGCAGTCTTAAATACGAGAAAGGCCATCTCGAAGGTCCCGGCATTCTCACGAAGGTTCTAAATATATATTTTCCTTTTTTCAAGAGCTAGGACTTCTTAAAAATGGAAAAAATTATCCCCCGTGTCCGGAACGTCCCGGTAATGCATGTAGATTTCCCTTGAACCATGCTGGTCAGAATTTCTGCAAAAAAATGAAGGGTCTACGTATCTTCCGT-----------------------------------------------------------------------------------------CCCGAACTGTGGTCACAATTTCAGCAAAAAAAAAAAAATGAAGTCTTTACATAC

Euphorbia bicompacta var. rubra TTTTTTCAAGTTTTCCGGGCCTTCCTCAAAATGGAAAAAGATATCGCCCATGTCCGAAAAGTCCCGGTATTACATGTAAATGTCCCTTTGAAAATGTTCGAACGGTGGTCGAAATTTCAACAAAAAGTGAAGGCTTTACATTTTTTTTTCCATTTTTTCCGAAACCCTTAAATATGGAAAAAATAACCCCCATGTCCGGAAAGCCCCGGTATTACATGTAAATGTCCCCTAAATAATGCTCGAACCGTGGTAAAAATTTCAGCTAAAAATGAAGGCTTTACATATTTTTTGCCATTTCCCCCGTAATTCCTAAAAATGGAAAAAATGCCCCCGTCGGGCGGATTGGCCCGATATTACACGTCAATTTCCCCCAAATCATGCTCGAACTGGGGTCGGAATTTCGGCAAAAAAGGAAGGCTCTGCATATTTTTTTCCATTTTTCCCGGAATTCTTAAAAATGGGAAAAATATCCCCCACGTCCGGAAACTCCCGATATCAAGTGTAAATGTCCCTCAAAGCATGCTCGAACCGTGGTAAAAATTTCAGCGAAAAATGAAGGCGTTGCATATTTTTTTCCATTTTTCCAGTGCCCGGAATTCACAAAAATGGAAAAAATAGCCCCGTTGTCCGGGAAGTCCCGGGATTGCATGTAAATTTCCCTTAAATCATGCTCGAACTATGGTAAAAATTTCAGAAAAAATCTCCAAGTATAGAGCAGTTTTTGGGGGGGTGTGGCTCCTGGAACAAATCGATGTCCGTTCCTCCCAGAGCTGGATTTGTCATAAGAAATACTATAGGGGCTGCGCCAGCTCTCAACCTTGGCCCGCACTGGGCTGCGGGCCCGTGGTGGGTGCCTCCCGCATGCTCTCCCGCATTCAAGCGCATCACTCGGTCTCCCAGAAAGTCCCGACCCGCCGCCCCACCGAACGGAAGAATATCGTGGAAAAGCTAAGCCCAAAAGCACCACACGCGCGCGCGCGACGGCGCGGCGCGTTCGCGATTTAGGCGATTTAGGCACTTGGCACTTAGCACGTAGCACGAAGCGCGAGGCGCGAGGCCAATCGGCGGAACATAGCAGGAAATATCCCGCAAAATTTATAAACGAGTAACGACACGGCATGAAAAAGAAGGTAGCTAGCAAAAATTTTAAAAAAAAGAAAGGAACATGAAGGCCAACCCGAAGTTCAGGCAAGAGTGCAGGGCGGCCTGGCCCGCCTTCGTGTGCGGGAGCAGGCGGGACAGCGTGCAGCGCTGTCCCGCATTCCTGCCTGTGCCTGGCGAGGCTAGGCAGGACGGCCGGCGTGCTGGCCCGCCCGCGTGTGCAGGAGGGCCTGCCAGTGGGAGGGCTGGCCCGCCTGGGCAGGGCTGGCAAGCTGCCCTGGCTCGCCTGAGTGCATGGCGGGGGAGGGCAAGCTGGCCAGCACGCAGCCCTGCCTGGCTCGCCAGCCAGCACCACGCAGGCAGGCCGCCAACCAGAGGTCAGCATAGGCATGCCAGCACGCCCAGCCGAGCTCTGCCGGCGGTGCCCACCCAGTCCGCGAAAAGGAGGCTAGGCCAGCCTGCTGTCTCGCCTGGCCGAGGCTCAGGATGGCTTCGCTGGGCCCGATCCAAATTTCGTTTGCAAAACCTTCGCACAGATAATAAGTTTTCCCGCACCATATTTTTTATACTGACTTTTACCATTTATTAGGAATTTTGTACCGAGTTCGTGATGCATTTGTTGAGTTTATATATTTTTTTGTATTTTTACGATTCTCGGACTTCGTAAATATGGAAAAAATACCTCCCGTGGGTAAAAGGTTACAATATTATATGGAAAAATCCCTTAATTCATGTTCTATCTACAGTCAAAATTTCAGGAAAAAACTCGAAGGTTTGACCGGTTTTTAAGGGGGTGTGGCTCCTGGAACAAATCGATGTCTGTTCCTCCCAGAGCTGGAAATGCTATAAGGACTACTATAGGGGGGTACACCTGCTCTCAACCCAGGCCACCACTGGGGCTGCGGGCCCGTGTTGGGTGCTTGCCGCATGCTCACCCCCTATCATGCGGGGAATTCAGCCACTAAGAACGTCCCGACCCGCCGCCCCTCCTCCGGCCGCCGGCCGCCGCCACGCGCGGTGGCCGGAAAATCCAAAATTCTTAGAACGCTGAATTCCGCACCCCGAGAGCCCCTTTTCCCCCTGCCATTGTCGGAACTCGTTCGAATTGGGGGTAAAGTGGGTTTTCGGAGGCAACAACGTGCCCCGCGGCGCTGTCCCGATCGCTAGTGTGTGCCTACTCGTAGTTTTGTCTTGCATCGGATAGCTGATTGAGCTGCTCTCAGTGTGTATGGTGCTCGACTTCAAGCTGCTTTGGAAGCACGCCTAGAGTGAGGGGTAGCCGCTTGCTGCGCACGGTATCGGACGAGGGAAAAAGGAATTTCGGAAAAAAACGTTTCCGTGCCGTGCGCGATGTTGCTACATCCGTAGTTGCTTGCTGCGTGCGGTGTGGGGCGATGCAAAAAATGGAATTTGAAAATAAATTGTTTTCACGGAGTGCGCTTCGGCGTTGCCCCGATCGATAGTGCGTGTTGCTCGGTGGATTTGTTTGGCATTGGATAGCTGATCGAGTTGCTCTCGGTGTGAAGGTGCTGGACGTTGGTCCACTCGGCAGCCCGCCTTGAGTGAGGGGCAGTTGGTTGATGCGTGCGGTGCCAAGTGGCGAAAAAATGGATTTCACTAAAAATGTCTTTCTTGCTGTGCGCTCAGGCGCCGTCCCGATCGGTAGTGTGCGTTATGTCCGGTGGTTCTGTTTGGCATCTGATAGCTGATCGAGTCGCTCTCAGTGTGTAAGGTGCTGGGTTTTGGACTATTTGGCTTGCCTGCCTATCGTGAGGAGCGGTTGCTTGGTGCGTACAGTTCCGGACGGCGAAAAATTGAATTCCAATAAAATATTATTCTCTCAACACGTGCTACTTTATCATGCGGTAAGGAATGTTCTCTCGCACACAGCGGTTCGGGCGATGTCTCTACTCGACGTTTCGGCACTGCTTGATTCGTTCTCGAAGACAGCAGTGCAGTTCGGGGGGTGGGGATGTTGCTCAATATACGCGGCGGTGCATGAGTGGTAAATAGGCCATTGGGGTTGGCAGGCTCTGTGCTAGCGCATCGAACTGTCGTACCTTGAGGCCACTCAGTGGTGTCCCGGAGGCGTATTGCTATGTCGGGCGGGGATGGTTTCTGTGTTGCATACCCGCGCAGTGGAATGGAATTTTGTTGCCAAGAAACATTCGTCCCGTGCCCTTTTAGGGGCGTCGGATGAACCATGCAGCAGCTCTCGTGTGCCGGGCATGCCTTTTTGGCTTCTCTGGCACATGTGAAGGTGCTCGTGCTCTCGGATGCGGAATGCTTTTGCGAGAGGAGGGATTGAGTTTCCTTTGTGTGTTCTCGCTGTCCCTACATAAGAACCACCGTCCTTTCCGCACAGTGGCCTTGGTTGCTGCGGTGTACTATGTCTGCTTGCGGGTTAGGACGGCATGGAGGAATGCTACCTGGTTGATCCTGCCAGTAGTCATATGCTTGTCTCAAAGATTAAGCCATGCATGTGTAAGTATGAACTAATTCAGACTGTGAAACTGCGAATGGCTCATTAAATCAGTTATAGTTTGTTTGATGGTACCTGCTACTCGGATAACCGTAGTAATTCTAGAGCTAATACGTGCAACAAACCCCGACTTCTGGAAGGGATGCATTTATTAGATAAAAGGTCGACGCGGGCTCTGCCCGTTGCTCTGATGATTCATGATAACTCGACGGATCGCACGGCCATCGTGCTGGCGACGCATCATTCAAATTTCTGCCCTATCAACTTTCGATGGTAGGATAGAGGCCTACCATGGTGGTGACGGGTGACGGAGAATTAGGGTTCGATTCCGGAGAGGGAGCCTGAGAAACGGCTACCACATCCAAGGAAGGCAGCAGGCGCGCAAATTACCCAATCCTGACACGGGGAGGTAGTGACAATAAATAACAATACCGGGCTCTTCGAGTCTGGTAATTGGAATGAGTACAATCTAAATCCCTTAACGAGGATCCATTGGAGGGCAAGTCTGGTGCCAGCAGCCGCGGTAATTCCAGCTCCAATAGCGTATATTTAAGTTGTTGCAGTTAAAAAGCTCGTAGTTGGACCTTGGGTTGGGTCGACCGGTCCGCCTTACGGTGTGCACCTGTCGGCTCGTCCCTTCTGCCGGCGATGCGCTCCTGGCCTTAACTGGCCGGGTCGTGCCTCCGGCGCTGTTACTTTGAAGAAATTAGAGTGCTCAAAGCAAGCCTACGCTCTGTATACATTAGCATGGGATAACATCATAGGATTTCGGTCCTATTCTGTTGGCCTTCGGGATCGGAGTAATGATTAACAGGGACAGTCGGGGGCATTCGTATTTCATAGTCAGAGGTGAAATTCTTGGATTTATGAAAGACGAACAACTGCGAAAGCATTTGCCAAGGATGTTTTCATTAATCAAGAACGAAAGTTGGGGGCTCGAAGACGATCAGATACCGTCCTAGTCTCAACCATAAACGATGCCGACCAGGGATCGGCGGATGTTGCTTTTAGGACTCCGCCGGCACCTTATGAGAAATCAAAGTCTTTGGGTTCCGGGGGGAGTATGGTCGCAAGGCTGAAACTTAAAGGAATTGACGGAAGGGCACCACCAGGAGTGGAGCCTGCGGCTTAATTTGACTCAACACGGGGAAACTTACCAGGTCCAGACATAGTAAGGATTGACAGACTGAGAGCTCTTTCTTGATTCTATGGGTGGTGGTGCATGGCCGTTCTTAGTTGGTGGAGCGATTTGTCTGGTTAATTCCGTTAACGAACGAGACCTCAGCCTGCTAACTAGCTATGCGGAGGTATCCCTCCGCGGCCAGCTTCTTAGAGGGACTATGGCCTTCTAGGCCAAGGAAGTTTGAGGCAATAACAGGTCTGTGATGCCCTTAGATGTTCTGGGCCGCACGCGCGCTACACTGATGTATTCAACGAGTCTATAGCCTTGGCCGACAGGCCCGGGTAATCTTTGAAATTTCATCGTGATGGGGATAGATCATTGCAATTGTTGGTCTTCAACGAGGAATTCCTAGTAAGCGCGAGTCATCAGCTCGCGTTGACTACGTCCCTGCCCTTTGTACACACCGCCCGTCGCTCCTACCGATTGAATGGTCCGGTGAAGTGTTCGGATCGCGGCGACGTGGGCGGTTCGCCGCCGGCGACGTCGCGAGAAGTCCACTGAACCTTATCATTTAGAGGAAGGAGAAGTCGTAACAAGGTTTCCGTAGGTGAACCTGCGGAAGGATCATTGTCGAAACCTGCCAGCAGAATGACCCGCGAACGTGTTTATAAATCGAGGGGCCGCTGCAGGATTCATCCAGCGATGGCACCTCACTAGGGCCCTGGCAGGGGATGCGGTGCGGTGGGATCCACCGTTCCCTGCGATCTCCTGTTTGCGGCCTATTAACAAAACCCCGGCGCCGTACGCGCCAAGGAATTGTAAAAAAAGATTGTGCAGCCCGATCGCACTGGCAACGGTGTGGCGGGTTTCACTGCGCTTTGAGAACCAAAATGACTCTCGGCAACGGATATCTCGGCTCTCGCATCGATGAAGAACGCAGCGAAATGCGATACTTGGTGTGAATTGCAGGATCCCGCGAACCATCGAGTCTTTGAACGCAAGTTGCGCCCGAAGCCTTTCGGCCGAGGGCACGTCTGCCTGGGTGTCACTCAAACGTCGCTCCAAACCCCTTCCATCGGGAGGGGTATGCGGGGCGGATGCTGGCCTCCCGTGTGCGTATCGCTCGCGGTTGGCCGAAATTCCTAGTCCTCGGCACGACGCCACGGAATCGGTGGTTGCAAGACCCTCGGAGAAAGCCTTGTGCGCTTGTAAGCCCTTTCGGACCATGAGACCCCAGAGCGTACCTAGCACTGCGACCCCAGGTCAGGCGGGATTACCCGCTGAGTTTAAGCATATCAATAAGCGGAGGAAAAGAAACTTACCAGGATTCCCCTAGTAACGGCGAGCGAACCGGGAAGAGCCCAGCTTGAGAATCGTGCGCCTGCGGCGTTCGAATTGTAGTCTGGAGAAGCGTCCTCAGCGGCGGACCGGGCCCAAGTCCCCTGGAAGGGGGCGCCGGAGAGGGTGAGAGCCCCGTCGTGCCCGGACCCTGTCGCACCACGAGGCGCTGTCTACGAGTCGGGTTGTTTGGGAATGCAGCCCAAATCGGGCGGTAAATTCCGTCCAAGGCTAAATATGGGCGAGAGACCGATAGCGAACAAGTACCGCGAGGGAAAGATGAAAAGGACTTTGAAAAGAGAGTCAAAGAGTGCTTGAAATTGTCGGGAGGGAAGCGGATGGGGGCCGGCGATGCGCCCCGGTCGGATGTGGAACGGTGACAAGCCGGTCCGCCGATCGGCTCGGGGCGCGGACCGATACGGATTGAGGCGGCGGCGTAAGCCCAGGAATTTGAAACGCCTGTGGAGATGCCGTCGCAGCAATCGTGGAAAGCAGCACGCGCCGTCTCGGCGTGCCTCGGCACCTGCGTGCTACTGGTGTCGGCCAGCGGGCTCCCCATTCGGCCCGTCTTGAAACACGGACCAAGGAGTCTGACATGTGTGCGAGTCAACGGGCGAGTAAACCCGTAAGGCGCAAGGAAGCTGACTGGCGGGATCCCCTAGAGGGTTGCACCGCCGACCGACCTTGATCTTCTGAGAAGGGTTCGAGTGAGAGCATGCCTGTCGGGACCCGAAAGATGGTGAACTATGCCTGAGCGGGGCGAAGCCAGAGGAAACTCTGGTGGAGGCCCGCAGCGATACTGACGTGCAAATCGTTCGTCTGACTTGGGTATAGGGGCGAAAGACTAATCGAACCGTCTAGTAGCTGGTTCCCTCCGAAGTTTCCCTCAGGATAGCTGGAGCTCGGAACGAGTTCTATCGGGTAAAGCCAATGATTAGAGGCATCGGGGGCGCAACGCCCTCGACCTATTCTCAAACTTTAAATAGGTAGGACGGCGCGGCTGCTTCGTTGAGCCGCGCCACGGAATCGAGAGCTCCAAGTGGGCCATTTTTGGTAAGCAGAACTGGCGATGCGGGATGAACCGGAAGCCGGGTTACGGTGCCCAACTGCGCGCTAACCTAGAACCCACAAAGGGTGTTGGTCGATTAAGACAGCAGGACGGTGGTCATGGAAGTCGAAATCCGCTAAGGAGTGTGTAACAACTCACCTGCCGAATCAACTAGCCCCGAAAATGGATGGCGCTTAAGCGCGCGACCTATACCCGGCCGTCGGGGCAAGAGCCAGGCCCCGATGAGTAGGAGGGCGCGGCGGTCGCTGCAAAACCCAGGGCGCGAGCCCGGGCGGAGCGGCCGTCGGTGCAGATCTTGGTGGTAGTAGCAAATATTCAAATGAGAACTTTGAAGGCCGAAGAGGGGAAAGGTTCCATGTGAACGGCACTTGCACATGGGTTAGTCGATCCTAAGAGACGGGGGAAGCCCGTCCGACAGCGCGTCCGCGCGCGAGCTTCGAAAGGGAATCGGGTTAAAATTCCCGAACCGGGACGCGGCGGCTGACGGCAACGTTAGGGAGTCCGGAGACGTCGGCGGGGGCCTCGGGAAGAGTTATCTTTTCTGTTTAACAGCCCGCCCACCCTGGAAACGACTCAGTCGGAGGTAGGGTCCAGCGGCTGGAAGAGCACCGCACGTCGCGCGGTGTCCGGTGCGCCCCCGGCGGCCCGTGAAAATCCGGAGGACCGAGTGCCATCCACGCCCGGTCGTACTCATAACCGCATCAGGTCTCCAAGGTGAACAGCCTCTGGTCGATGGAACAATGTAGGCAAGGGAAGTCGGCAAAATGGATCCGTAACCTCGGGAAAAGGATTGGCTCTGAGGGCTGGGCCCGGGGGTCCCAGTCCCGAACCCGTCGGCTGTCGGCGGACTGCTCGAGCTGCTCCCGCGGCAAGAGCGGGTCGCTGCGTGCCGGCCGGGGGACGGATTGGGAACGGCCCCTCTGGGGGCCTTCCCCGGGCGTCGAACAGTCGACTCAGAACTGGTACGGACAAGGGGAATCCGACTGTTTAATTAAAACAAAGCATTGCGATGGTCCCTGCGGATGCTAACGCAATGTGATTTCTGCCCAGTGCTCTGAATGTCAAAGTGAAGAAATTCAACCAAGCGCGGGTAAACGGCGGGAGTAACTATGACTCTCTTAAGGTAGCCAAATGCCTCGTCATCTAATTAGTGACGCGCATGAATGGATTAACGAGATTCCCACTGTCCCTGTCTACTATCCAGCGAAACCACAGCCAAGGGAACGGGCTTGGCAGAATCAGCGGGGAAAGAAGACCCTGTTGAGCTTGACTCTAGTCCGACTTTGTGAAATGACTTGAGAGGTGTAGTATAAGTGGGAGCCGGAAACGGCGATAGTGAAATACCACTACTTTTAACGTTATTTTACTTATTCCGTGAATCGGAGGCGGGGCATTGCCCCTCTTTTTGGACCAAAGGCCGCTTCGCGGTCGATCCGGGCGGAAGACATTGTCAGGTGGGGAGTTTGGCTGGGGCGGCACATCTGTTAAAAGATAACGCAGGTGTCCTAAGATGAGCTCAACGAGAACAGAAATCTCGTGTGGAACAAAAGGGTAAAAGCTCGTTTGATTCTGATTTCCAGTACGAATACGAACCGTGAAAGCGTGGCCTATCGATCCTTTAGACCTTCGGAATTTGAAGCTAGAGGTGTCAGAAAAGTTACCACAGGGATAACTGGCTTGTGGCAGCCAAGCGTTCATAGCGACGTTGCTTTTTGATCCTTCGATGTCGGCTCTTCCTATCATTGTGAAGCAGAATTCACCAAGTGTTGGATTGTTCACCCACCAATAGGGAACGTGAGCTGGGTTTAGACCGTCGTGAGACAGGTTAGTTTTACCCTACTGATGACGGTGTCGCGATGGTAATTCAACCTAGTACGAGAGGAACCGTTGATTCGCACAATTGGTCATCGCGCTTGGTTGAAAAGCCAGTGGCGCGAAGCTACCGTGCGCTGGATTATGACTGAACGCCTCTAAGTCAGAATCCGGGCCAGAAGCGATGCATGCGTCCGCCGCTCGTTTGCCGACCCTCAGTAGGGGCCGTCCGGCCCCCAAAGGCACGTGTCGTTGGCTAAGCCCTCGCGGCAGACAAGCCGTGCGGGCAGCCTTGAAGTACAATTCCCACCGGGCGGCGGGCAGAATCCTTTGCAGACGACTTAAATACGCGACGGGGTATTGTAAGTGGCAGAGTGGCCTTGCTGCCACGATCCACTGAGATTCAGCCCTTTGTCGCTCCGATTCGTCCCTCCCTCCCAAGAAACTTTTTCCATCTCAATAGAATATCGAGCGGAGGCTGGGGTCTCGATTTCCGCTCGTAGAAAGAGGGCCAAGGAG--------GGTATTGCATATGGCACGGGGGAAGCAAGAGATTAAGCACACCGCGGGGTGGAAAAAGCATCTGCGCACGGGCAGGTTAGAAAAACATACAAGCGATGCATCCCTTCAATGTCCCTCGGCATTGACCGCTTGTTCATTTTTTTTTCCGGCTTAATGCGTACGTTTCGAACCACTCGTACACCAAGGCTGCCGCGGCCACGCGTGCCTCACCGGTCGATGCGGTCTACTGCCTTCACAGGCGAGGCAGGAGCAGCATCGAATTCCTGCCCCGGCACCACGCACGAGGCCGGCGGTGCCTACCTCTCGTGCGCAAGCGAGACCGATGCCAGCGCGGCTGACTGGCCTGAATAGGAGAGGCCAGTGCTAGTCCGCGCACGCCACATGCCCCTTCAAAGTTTCCCCGCATCGCTCGAGAATTTTACGGCAGGCCCGTTGAACATCTCCATCGGGAGAGAGAATTTGCATCTCCGCCCCAAGCGTCGATGCTAGTCCGCGCACGCCACATGCCCCTACAAAGTTTCCCCGCACCGCTCGAGAATTTTATGGCAGACCCGTTAAATATCTCCATCGGGAGAGAAAATTTGCATCTCCGCCCCCAACCGTCGAGAAAAGATCAACACTTTTACCACCCCGAGGGTTGTACACACAGCCTCCTGAAACAAAGGGGCGGCAAAAACGCGTTTCGCCTCCAGTCCACGCTCGGTCTTTCGGAACGGGTTCAACGGCAGTTGATTTTATACCGAGTTGCCAAATTCTCACCAGGGCTCTGTATTCTTTTTCGTTTGTCAAGTTCCCGGCAGTCTTAAATACGAGAAAGGCCATCTCGAAGGTCCCGGCATTCTCACGAAGGTTCTAAATATATATTTTCCTTTTTTCAAGAGCTAGGACTTCTTAAAAATGGAAAAAATTATCCCCCGTGTCCGGAAAGTCCCGGTAATGCATGTAGATTTCCCTTGAACCATGCTGGTCAGAATTTCTGCAAAAAAATGAAGGGTCTACGTATCTTCTGTCCATTTTTCCCGGACTCCCTAAAAATGGGAAAAATACACCCCATGTTCGGAAAGTCCCGATATTACATGTAAATATCCCTTAAATCACACCCGAACCGTGGTCACAATTTCAGCAAAAAAAAAAAAATGAAGTCTTTACATAT

Euphorbia kirkii TTTTTTCAAGTTTTCCGGGCCTTCCTCAAAATGGAAAAAGATATCGCCCATGTCCGAAAAGTCCCGGTATTACATGTAAATGTCCCTTTGAAAATGTTCGAACGGTGGTCGAAATTTCAACAAAAAGTGAAGGCTTTACATTTTTTTTTCCATTTTTTCCGAAACCCTTAAATATGGAAAAAATAACCCCCATGTCCGGAAAGGCCCGGTATTACATGTAAATGTCCCCTAAATAATGCTCGAACCGTGGTAAAAATTTCAGCTAAAAATGAAGGCTTTACATATTTTTTGCCATTTTCCCCGTAATTCCTAAAAATGGAAAAAATACCCCCGTCGGGCGGATTGGCCCGATATTACACGTCAATTTCCCCCAAATCATGCTCGAACTGGGGTCGGAATTTCGGCAAAAAAGGAAGGCTCTGCATATTTTTTTCCATTTTTCCCGGAATTCTTAAAAATGGGAAAAATATCCCCGACGTCCGGAAACTCCCGATATCAGGTGTAAATGTCCCTCAAATCATGCTCGAACCGTGGTAAAAATTTCAGCGAAAAATGAAGGCGCTGCATATTTTTTTCCATTTTTCCAGTGCCCGGAATTCACAAAAATGGAAAAAATAGCCCCGTTGTCCGGGAAGTCCCGGGATTGCATGTAAATTTCCCTTAAATCATGCTCGAACTATGGTAAAAATTTCAGAAAAAATCTCCAAGTATAGAGCAGTTTTTGGGGGGGTGTGGCTCCTGGAACAAATCGATGTCCGTTCCTCCCAGAGCTGGATTTGTCATAAGAAATACTATAGGGGCTGCGCCAGCTCTCAACCTTGGCCCGCACTGGGCTGCGGGCCCGTGTTGGGTGCCTCCCGCATGCTCTCCCGCATTCAAGCGCATCACTCGGTCTCCCAGAAAGTCCCGACCCGCCGCCCCACCGAACGGAAGAATATCGTGGAAAAGCTAAGCCCAAAAGCACCACACGCGCGCGCGCGACGGCGCGGCGCGTTCGCGATTTAGGCGATTTAGGCACTTGGCACTTAGCACGTAGCACGAAGCGCGAGGCGCGAGGCCAATCGGCGGAACATAGCAGAAAATATCCCGCAAAATTTATAAACGAGTAACGACACGGCATGAAAAAGAAGGTAGCTAGCAAAAATTTTAAAAAAAAGAAAGGAACATGGAGGCCAACCCGAAGTTCAGGCAAGAGTGCAGGGCGGCCTGGCCCGCCTTCGTGTGCGGGAGCAGGCGGGACAGCGTGCAGCGCTGTCCCGCATTCCTGCCTGTGCCTGGCGAGGCTAGGCAGGACGGCCGGCGTGCTGGCCCGCCCGCGTGTGCAGGAGGGCCTGCCAGTGGGAGGGCTGGCCCGCCTGGGCAGGGCTGGCAAGCTGCCCTGGCTCGCCTGAGTGCATGGCGGGGGAGGGCAAGCTGGCCAGCACGCTGCCCTGCCTGGCTCGCCAGCCAGCACCGCGCAGGCAGGCCGCCAACCAGAGGTCAGCATAGGCATGCCAGCACGCCCAGCCGAGCTCTGCCGGCGGTGCTCACCCCGTCCGCGCAAAGGAGGCTAGGTCAGCGTGCTGTCTCGCCTGGCCGAGGCTCAGAATGGCTTCGCTGGGCCCGATCCAAATTTCGTTTGCAAAACCTTCGCACAGATAATAAGTTTGCCCGCACCATATTTTTAATACTGACTTTACCCATTTATTAGGAATTTTGTACCGAGTTTGGTATGCATTTGTTGAGTTTATATATTTTTTTGTATTTTTACGATTCTCGGACTTCGTAAATATGGAAAAAATACCTCCCGTGGGTAAAAGGTTACAATATTTTATGGAAAAATCCCTTAATTCATGTTCTATCTACAGTCAAAATTTCAGGAAAAAACTCGAAGGTTTGACCGGTTTTTAAGGGGGTGTGGCTCCTGGAACAAATCGATGTCTGTTCCTCCCAGAGCTGGAAATGCTATAAGGACTACTATAGGGGGGTACACCTGCTCTCAACCCAGGCCACCACTGGGGCTGCGGGCCCGTGTTGGGTGCTTGCCGCATGCTCACCCCCTATCATGCGGGGAATTCAGCCACTAAGAACGTCCCGACCCGCCGCCCCTCCTCCGGCCGCCGGCCGCCGCCCCGCGCGGTGGTCGGAAAATCCAAAATTCTTAGAACGCTGAATTCCGCACCCCGAGAGCCCCTTTTCCCCCTGCCATTGTCGGAACTCGTTCGAATTGGGGGTAAAGTGGGTTTTCGGAGGCAACAACGTGCCCCGCGGCGCTGTCCCGATCGCTAGTGTGTGCCTACTCGTAGTTTTGTCTTGCATCGGATAGCTGATCGAGCTGCTCTCAGTGTGTATGTTGCTCGACTTCAAGCTGCTTGGGAAGCACGCCTAGAGTGAGGGGTAGCCGCTTGCTGCGCACGGTATCGGACGAGGGAAAAAGGAATTTCGGAAAAAAACGTTTCCGTGCCGTGCGCGATGTTGCTACATCCGTAGTTGCTTGCTGCGTGCGGTGTGGGGCGATGTAAAAAATGGAATTTGAAAATAAATTGTTTTCACGGAGTGCGCTTCGGCGTTGCCCCGATCGATAGTGCGTGTTGCTCGGTGGATTTGTTTGGCATTGGATAGCCGATCGAGTTGCTCTCGGTGTGAAGGTGCTGGACGTTGGTCCACTCGGCAGCCCGCCTTGAGTGAGGGGCAGTTGGTTGATGCGTGCGTTGCCAAGTGGCGAAAAAATGGATTTCACTAAAAATGTCTTTCTTGCTGTGCGCTCAGGCGCCGTCCCGATCGGTAGTGTGCGTTATGTCCGGTGGTTCTGTTTGGCATCTGATAGCTGATCGAGTCGCTCTCAGTGTGTAAGGTGCTGGGTTTTGGACTATTTGGCTTGCCTGCCTATCGTGAGGAGCGGTTGCTTGGTGCGTACAGTTCCGGACGGCGAAAAATTGAATTCCAATAAGATATTATTTTCTCAACACGTGCTACTTTATCATGCGGTAAGGAATGTTCTCTCGCACACAGCGGTTCGGGCGATGTCTCTACTCGACGTTTCGGCACTGCTTGATTCGTTCTCGAAGACAGCAGTGCAGTTCGGGGGGTGGGGATGTTGCTCAATATACGCGGCGGTGCATGAGTGGTAAATAGGCCATTGGGGTTGGCAGGCTCTGTGCTAGCGCATCGAACTGTCGTACCTTGAGGCCACTCAGTGGTGTCCCGGAGGCGTATTGCTATGTCGGGCGGGGATGGTTTCTGTGTTGCATACCCGCGCAGTGGAATGGAATTTTGTTGCCAAGAAACATTCGTCCCGTGCCCTTTTAGGGGCGTCGGATGAACCATGCAGCAGCTCTCGTGTGCCGGGCATGCCTTTTTGGCTTCTCTGGCACATGTGAAGGTGCTCGTGCTCTCGGATGCGGAATGCTTTTGCGAGAGGAGGGATTGAGTTTCCTTTATGTGTTCTCGCTGTCCCTACATAAGAACCACCGTCCTTTCCGCACAGTGGCCTTGGTTGCTGCGGTGTACTATGTCTGCTTGCGGGTTAGGACGGCATGGAGGAATGCTACCTGGTTGATCCTGCCAGTAGTCATATGCTTGTCTCAAAGATTAAGCCATGCATGTGTAAGTATGAACTAATTCAGACTGTGAAACTGCGAATGGCTCATTAAATCAGTTATAGTTTGTTTGATGGTACCTGCTACTCGGATAACCGTAGTAATTCTAGAGCTAATACGTGCAACAAACCCCGACTTCTGGAAGGGATGCATTTATTAGATAAAAGGTCGACGCGGGCTCTGCCCGTTGCTCTGATGATTCATGATAACTCGACGGATCGCACGGCCATCGTGCTGGCGACGCATCATTCAAATTTCTGCCCTATCAACTTTCGATGGTAGGATAGAGGCCTACCATGGTGGTGACGGGTGACGGAGAATTAGGGTTCGATTCCGGAGAGGGAGCCTGAGAAACGGCTACCACATCCAAGGAAGGCAGCAGGCGCGCAAATTACCCAATCCTGACACGGGGAGGTAGTGACAATAAATAACAATACCGGGCTCTTCGAGTCTGGTAATTGGAATGAGTACAATCTAAATCCCTTAACGAGGATCCATTGGAGGGCAAGTCTGGTGCCAGCAGCCGCGGTAATTCCAGCTCCAATAGCGTATATTTAAGTTGTTGCAGTTAAAAAGCTCGTAGTTGGACCTTGGGTTGGGTCGACCGGTCCGCCTTACGGTGTGCACCTGTCGGCTCGTCCCTTCTGCCGGCGATGCGCTCCTGGCCTTAACTGGCCGGGTCGTGCCTCCGGCGCTGTTACTTTGAAGAAATTAGAGTGCTCAAAGCAAGCCTACGCTCTGTATACATTAGCATGGGATAACATCATAGGATTTCGGTCCTATTCTGTTGGCCTTCGGGATCGGAGTAATGATTAACAGGGACAGTCGGGGGCATTCGTATTTCATAGTCAGAGGTGAAATTCTTGGATTTATGAAAGACGAACAACTGCGAAAGCATTTGCCAAGGATGTTTTCATTAATCAAGAACGAAAGTTGGGGGCTCGAAGACGATCAGATACCGTCCTAGTCTCAACCATAAACGATGCCGACCAGGGATCGGCGGATGTTGCTTTTAGGACTCCGCCGGCACCTTATGAGAAATCAAAGTCTTTGGGTTCCGGGGGGAGTATGGTCGCAAGGCTGAAACTTAAAGGAATTGACGGAAGGGCACCACCAGGAGTGGAGCCTGCGGCTTAATTTGACTCAACACGGGGAAACTTACCAGGTCCAGACATAGTAAGGATTGACAGACTGAGAGCTCTTTCTTGATTCTATGGGTGGTGGTGCATGGCCGTTCTTAGTTGGTGGAGCGATTTGTCTGGTTAATTCCGTTAACGAACGAGACCTCAGCCTGCTAACTAGCTATGCGGAGGTATCCCTCCGCGGCCAGCTTCTTAGAGGGACTATGGCCTTCTAGGCCAAGGAAGTTTGAGGCAATAACAGGTCTGTGATGCCCTTAGATGTTCTGGGCCGCACGCGCGCTACACTGATGTATTCAACGAGTCTATAGCCTTGGCCGACAGGCCCGGGTAATCTTTGAAATTTCATCGTGATGGGGATAGATCATTGCAATTGTTGGTCTTCAACGAGGAATTCCTAGTAAGCGCGAGTCATCAGCTCGCGTTGACTACGTCCCTGCCCTTTGTACACACCGCCCGTCGCTCCTACCGATTGAATGGTCCGGTGAAGTGTTCGGATCGCGGCGACGTGGGCGGTTCGCCGCCGGCGACGTCGCGAGAAGTCCACTGAACCTTATCATTTAGAGGAAGGAGAAGTCGTAACAAGGTTTCCGTAGGTGAACCTGCGGAAGGATCATTGTCGAAACCTGCCAGCAGAATGACCCGCGAACGTGTTTATAAATCGAGGGGCCGCTGCAGGATTCATCCAGCGATGGCACCTCACTAGGGCCCTGGCAGGGGATGCGGTGCGGTGGGATCCACCGTTCCCTGCGATCTCCTGTTTGCGGCCTATTAACAAAACCCCGGCGCCGTACGCGCCAAGGAATTGTAAAAAAAGATTGTGCAGCCCGATCGCACTGGCAACGGTGTGGCGGGTTTCACTGCGCTTTGAGAACCAAAATGACTCTCGGCAACGGATATCTCGGCTCTCGCATCGATGAAGAACGCAGCGAAATGCGATACTTGGTGTGAATTGCAGGATCCCGCGAACCATCGAGTCTTTGAACGCAAGTTGCGCCCGAAGCCTTTCGGCCGAGGGCACGTCTGCCTGGGTGTCACTCAAACGTCGCTCCAAACCCCTTCCATCGGGAGGGGTATGCGGGGCGGATGCTGGCCTCCCGTGTGCGTATCGCTCGCGGTTGGCCGAAATTCCTAGTCCTCGGCACGACGCCACGGAATCGGTGGTTGCAAGACCCTCGGAGAAAGCCTTGTGCGCTTGTAAGCCCTTTCGGACCATGAGACCCCAGAGCGTACCTAGCACTGCGACCCCAGGTCAGGCGGGATTACCCGCTGAGTTTAAGCATATCAATAAGCGGAGGAAAAGAAACTTACCAGGATTCCCCTAGTAACGGCGAGCGAACCGGGAAGAGCCCAGCTTGAGAATCGTGCGCCTGCGGCGTTCGAATTGTAGTCTGGAGAAGCGTCCTCAGCGGCGGACCGGGCCCAAGTCCCCTGGAAGGGGGCGCCGGAGAGGGTGAGAGCCCCGTCGTGCCCGGACCCTGTCGCACCACGAGGCGCTGTCTACGAGTCGGGTTGTTTGGGAATGCAGCCCAAATCGGGCGGTAAATTCCGTCCAAGGCTAAATATGGGCGAGAGACCGATAGCGAACAAGTACCGCGAGGGAAAGATGAAAAGGACTTTGAAAAGAGAGTCAAAGAGTGCTTGAAATTGTCGGGAGGGAAGCGGATGGGGGCCGGCGATGCGCCCCGGTCGGATGTGGAACGGTGACAAGCCGGTCCGCCGATCGGCTCGGGGCGCGGACCGATACGGATTGAGGCGGCGGCGTAAGCCCAGGAATTTGAAACGCCTGTGGAGATGCCGTCGCAGCAATCGTGGAAAGCAGCACGCGCCGTCTCGGCGTGCCTCGGCACCTGCGTGCTACTGGTGTCGGCCAGCGGGCTCCCCATTCGGCCCGTCTTGAAACACGGACCAAGGAGTCTGACATGTGTGCGAGTCAACGGGCGAGTAAACCCGTAAGGCGCAAGGAAGCTGACTGGCGGGATCCCCTAGAGGGTTGCACCGCCGACCGACCTTGATCTTCTGAGAAGGGTTCGAGTGAGAGCATGCCTGTCGGGACCCGAAAGATGGTGAACTATGCCTGAGCGGGGCGAAGCCAGAGGAAACTCTGGTGGAGGCCCGCAGCGATACTGACGTGCAAATCGTTCGTCTGACTTGGGTATAGGGGCGAAAGACTAATCGAACCGTCTAGTAGCTGGTTCCCTCCGAAGTTTCCCTCAGGATAGCTGGAGCTCGGAACGAGTTCTATCGGGTAAAGCCAATGATTAGAGGCATCGGGGGCGCAACGCCCTCGACCTATTCTCAAACTTTAAATAGGTAGGACGGCGCGGCTGCTTCGTTGAGCCGCGCCACGGAATCGAGAGCTCCAAGTGGGCCATTTTTGGTAAGCAGAACTGGCGATGCGGGATGAACCGGAAGCCGGGTTACGGTGCCCAACTGCGCGCTAACCTAGAACCCACAAAGGGTGTTGGTCGATTAAGACAGCAGGACGGTGGTCATGGAAGTCGAAATCCGCTAAGGAGTGTGTAACAACTCACCTGCCGAATCAACTAGCCCCGAAAATGGATGGCGCTTAAGCGCGCGACCTATACCCGGCCGTCGGGGCAAGAGCCAGGCCCCGATGAGTAGGAGGGCGCGGCGGTCGCTGCAAAACCCAGGGCGCGAGCCCGGGCGGAGCGGCCGTCGGTGCAGATCTTGGTGGTAGTAGCAAATATTCAAATGAGAACTTTGAAGGCCGAAGAGGGGAAAGGTTCCATGTGAACGGCACTTGCACATGGGTTAGTCGATCCTAAGAGACGGGGGAAGCCCGTCCGACAGCGCGTCCGCGCGCGAGCTTCGAAAGGGAATCGGGTTAAAATTCCCGAACCGGGACGCGGCGGCTGACGGCAACGTTAGGGAGTCCGGAGACGTCGGCGGGGGCCTCGGGAAGAGTTATCTTTTCTGTTTAACAGCCCGCCCACCCTGGAAACGACTCAGTCGGAGGTAGGGTCCAGCGGCTGGAAGAGCACCGCACGTCGCGCGGTGTCCGGTGCGCCCCCGGCGGCCCGTGAAAATCCGGAGGACCGAGTGCCATCCACGCCCGGTCGTACTCATAACCGCATCAGGTCTCCAAGGTGAACAGCCTCTGGTCGATGGAACAATGTAGGCAAGGGAAGTCGGCAAAATGGATCCGTAACCTCGGGAAAAGGATTGGCTCTGAGGGCTGGGCCCGGGGGTCCCAGTCCCGAACCCGTCGGCTGTCGGCGGACTGCTCGAGCTGCTCCCGCGGCAAGAGCGGGTCGCTGCGTGCCGGCCGGGGGACGGATTGGGAACGGCCCCTCTGGGGGCCTTCCCCGGGCGTCGAACAGTCGACTCAGAACTGGTACGGACAAGGGGAATCCGACTGTTTAATTAAAACAAAGCATTGCGATGGTCCCTGCGGATGCTAACGCAATGTGATTTCTGCCCAGTGCTCTGAATGTCAAAGTGAAGAAATTCAACCAAGCGCGGGTAAACGGCGGGAGTAACTATGACTCTCTTAAGGTAGCCAAATGCCTCGTCATCTAATTAGTGACGCGCATGAATGGATTAACGAGATTCCCACTGTCCCTGTCTACTATCCAGCGAAACCACAGCCAAGGGAACGGGCTTGGCAGAATCAGCGGGGAAAGAAGACCCTGTTGAGCTTGACTCTAGTCCGACTTTGTGAAATGACTTGAGAGGTGTAGTATAAGTGGGAGCCGGAAACGGCGATAGTGAAATACCACTACTTTTAACGTTATTTTACTTATTCCGTGAATCGGAGGCGGGGCATTGCCCCTCTTTTTGGACCAAAGGCCGCTTCGCGGTCGATCCGGGCGGAAGACATTGTCAGGTGGGGAGTTTGGCTGGGGCGGCACATCTGTTAAAAGATAACGCAGGTGTCCTAAGATGAGCTCAACGAGAACAGAAATCTCGTGTGGAACAAAAGGGTAAAAGCTCGTTTGATTCTGATTTCCAGTACGAATACGAACCGTGAAAGCGTGGCCTATCGATCCTTTAGACCTTCGGAATTTGAAGCTAGAGGTGTCAGAAAAGTTACCACAGGGATAACTGGCTTGTGGCAGCCAAGCGTTCATAGCGACGTTGCTTTTTGATCCTTCGATGTCGGCTCTTCCTATCATTGTGAAGCAGAATTCACCAAGTGTTGGATTGTTCACCCACCAATAGGGAACGTGAGCTGGGTTTAGACCGTCGTGAGACAGGTTAGTTTTACCCTACTGATGACGGTGTCGCGATGGTAATTCAACCTAGTACGAGAGGAACCGTTGATTCGCACAATTGGTCATCGCGCTTGGTTGAAAAGCCAGTGGCGCGAAGCTACCGTGCGCTGGATTATGACTGAACGCCTCTAAGTCAGAATCCGGGCCAGAAGCGATGCATGCGTCCGCCGCTCGTTTGCCGACCCTCAGTAGGGGCCATCCGGCCCCCAAAGGCACGTGTCGTTGGCTAAGCCCTCGCGGCAGACAAGCCGTGCGGGCAGCCTTGAAGTACAATTCCCACCGGGCGGCGGGCAGAATCCTTTGCAGACGACTTAAATACGCGACGGGGTATTGTAAGTGGCAGAGTGGCCTTGCTGCCACGATCCACTGAGATTCAGCCCTTTGTCGCTCCGATTCGTCCCTCCCTCCCAAGAAACTTTTTCCATCTCAATAGAATATCGAGCGGAGGCTGGGGTCTCGATTTCCGCTCGTAGAAAGAGGGCCAAGGAGAGAACCCCGGTATTGCATATGGCACGGGGGAAGCAAGAGATTAAGCACACCGCGGGGTGGAAAAAGCATCTGCGCACGGGCAGGTTAGAAAAACATACAAGCGATGCATCCCTTCAATGTCCCTCGGCGTTGACCGCTTGTTCATTTTTTTTCCCGGCTTGATGCGTACGTTTCGAACCACTCGTACACCAAGGCTGCCGCGGCCACGCGTGCCTCACCGGTCGATGCGGTCTACTGCCTTCACAGGCGAGGCAGGAGCAGCATCGAATTCCTGCCCCGGCACCACGCACGAGGCCGGCGGTGCCTACCTCTCGTGCGCAAGCGAGACCGATGCCAGCGCGGCTGACTGGCCTGAATAGGAGAGGCCAGTGCTAGTCCGCGCACGCCACATGCCCCTTCAAAGTTTCCCCGCATCGCTCGAGAATTTTACGGCAGGCCCGTTAAACATCTCCATCGGGAGAGAGAATTTGCATCTCCGCCCCAAGCGTCGATGCTAGTCCGCGCACGCCACATGCCCCTTCAAAGTTTCCCCGCACCGCTCGAGAATTTTATGGCAGACCCGTTAAATATCTCCATCGGGAGAGAAAATTTGCATCTCCGCCCCCAACCGTCGAGAAAAGATTAACACTTTTACCACCCCGAGGGTTGTACACACAGCCTCCTGAAACAAAGGGGCGGCAAAAACGCGTTTCGCCTCCAGTCCACGCTCGGTCTTTCGGAACGGGTTCAACGGCAGTTGATTTTATACCGAGTTGCCAAATTCTCACGAGGGCTCTGTATTCTTTTTCGTTTGTCAAGTTCCCGGCAGTCTTAAATACGAGAAAGGCCATCTCGAAGGTCCCGGCATTTTCACGAAGGTTCTAAATATATATTTTCCTTTTTTCAAGAGCTAGGACTTCTTAAAAATGGAAAAAATTATCCCCCGTGTCCGGAAAGTCCCGATAATGCATGTAGATTTCCCTTGAACCATGCTGGTCAGAATTTCTGCAAAAAAAGGAAGGGTCTACGTATCTTCTGTCCATTTTTCCCGGACTCCCTAAAAATGGGAAAAATACCCCCCATGTTCGGAAAGTCCCGATATTACATGTAAATATCCCTTAAATCACACCCGAACTGTGGTCACAATTTCAGCAAAAAAAAAAAAATGAAGTCTTTACATAT

Euphorbia mbuinzauensis TTTTTTCAAGTTTTCCGGGCCTTCCTCAAAATGGAAAAAGATATCGCCCATGTCCGAAAAGTCCCGGTATTACATGTAAATGTCCCTTTGAAAATGTTCGAACGGTGGTCGAAATTTCAACAAAAAGTGAAGGCTTTACATTTTTTTTTCCATTTTTTCCGAAACCCTTAAATATGGAAAAAATAACCCCCATGTCCGGAAAGCCCCGGTATTACATGTAAATGTCCCCTAAATAATGCTCGAACCGTGGTAAAAATTTCAGCTAAAAATGAAGGCTTTACATATTTTTTGCCATTTTCCCCGTAATTCCTAAAAAAGGAAAAAATACCCCCGTCGGGCGGATTGGCCCGATATTACACGTCAATTTCCCCCAAATCATGCTCGAACTGGGGTCGGAATTTCGGCAAAAAAGGAAGGCTCTGCATATTTTTTTCCATTTTTCCCGGAATTCTTAAAAATGGGAAAAATATCCCCCACGTCCGGAAACTCCCGATATCAAGTGTAAATGTCCCTCAAATCATGCTCGAACCGTGGTAAAAATTTCAGCGAAAAATGAAGGCGTTGCATATTTTTTTCCATTTTTCCAGTGCCCGGAATTCACAAAAATGGAAAAAATAGCCCCGTTGTCCGGGAAGTCCCGGGATTGCATGTAAATTTCCCTTAAATCATGCTCGAACTATGGTAAAAATTTCAGAAAAAATCTCCAAGTATAGAGCAGTTTTTGGGGGGGTGTGGCTCCTGGAACAAATCGATGTCCGTTCCTCCCAGAGCTGGATTTGTCATAAGAAATACTATAGGGGCTGCGCCAGCTCTCAACCTTGGCCCGCACTGGGCTGCGGGCCCGTGTTGGGTGCCTCCCGCATGCTCTCCCGCATTCAAGCGCATCACTCGGTCTCCCAGAAAGTCCCGACCCGCCGCCCCACCGAACGGAAGAATATCGTGGAAAAGCTAAGCCCAAAAGCACCACACGCGCGCGCGCGACGGCGCGGCGCGTTCGCGATTTAGGCGATTTAGGCACTTGGCACTTAGCACGTAGCACGGAGCGCGAGGCGCGAGGCCAATCGGCGGAACATAGCAGAAAATATCCCGCAAAATTTATAAACGAGTAACGACACGGCATGAAAAAGAAGGTAGCTAGCAAAAATTTTAAAAAAAAGAAAGGAACATGAAGGCCAACCCGAAGTTCAGGCAAGAGTGCAGGGCGGCCTGGCCCGCCTTCGTGTGCGGGAGCAGGCGGGACAGCGTGCAGCGCTGTCCCGCATTCCTGCCTGTGCCTGGCGAGGCTAGGCAGGACGGCCGGCGTGCTGGCCCGCCCGCGTGTGCAGGAGGGCCTGCCAGTGGGAGGGCTGGCCCGCCTGGGCAGGGCTGGCAAGCTGCCCTGGCTCGCCTGAGTGCATGGCGGGGGAGGGCAAGCTGGCCAGCACGCAGCCCTGCCTGGCTCGCCAGCCAGCACCACGCAGGCAGGCCGCCAACCAGAGGTCAGCATAGGCATGCCAGCACGCCCAGCCGAGCTCTGCCGGCGGTGCTCACCCAGTCCGCGCAAAGGAGGCTAGGTCAGCGTGCTGTCTCGCCTGTCCGAGGCTCAGGATGGCTTCGCTGGGCCCGATCCAAATTTCGTTTGCAAAACCTTCGCACAGATAATAAGTTTTCCCGCACCATATTTTTTATACTGACTTTTACCATTTATTAGGAATTTTGTACCGAGTTCGTGATGCATTTGTTGAGTTTACATATTTTTTTGTATTTTTACGATTCTCGGACTTCGTAAATATGGAAAAAATACCTCCCGTGGGTAAAAGGTTACAATATTATATGGAAAAATCCCTTAATTCATGTTCTATCTACAGTCAAAATTTCAGGAAAAAACTCGAAGGTTTGACCGGTTTTTAAGGGGGTGTGGCTCCTGGAACAAATCGATGTCTGTTCCTCCCAGAGCTGGAAATGCTATAAGGACTACTATAGGGGGGTACACCTGCTCTCAACCCAGGCCACCACTGGGGCTGCGGGCCCGTGTTGGGTGCTTGCCGCATGCTCACCCCCTATCATGCGGGGAATTCAGCCACTAAGAACGTCCCGACCCGCCGCCCCTCCTCCGGCCGCCGGCCGCCGCCACGCGCGGTGGCCGGAAAATCCAAAATTCTTAGAACGCTGAATTCCGCACCCCGAGAGCCCCTTTTCCCCCTGCCATTGTCGGAACTCGTTCGAATTGGGGGTAAAGTGGGTTTTCGGAGGCAACAACGTGCCCCGCGGCGCTGTCCCGATCGCTAGTGTGTGCCTACTCGTAGTTTTGTCTTGCATCGGATAGCTGATTGAGCTGCTCTCAGTGTGTATGGTGCTCGACTTCAAGCTGCTTTGGAAGCACGCCTAGAGTGAGGGGTAGCCGCTTGCTGCGCACGGTATCGGACGAGGGAAAAAGGAATTTCGGAAAAAAACGTTTCCGTGCCGTGCGCGATGTTGCTACATCCGTAGTTGCTTGCTGCGTGCGGTGTGGGGCGATGTAAAAATTGGAATTTGAAAATAAATTGTTTTCACGGAGTGCGCTTCGGCGTTGCCCCGATCGATAGTGCGTGTTGCTCGGTGGATTTGTTTGGCATTGGATAGCTGATCGAGTTGCTCTCGGTGTGAAGGTGCTGGACGTTGGTCCACTCGGCAGCCCGCCTTGAGTGAGGGGCAGTTGGTTGATGCGTGCGGTGCCATGTGGCGAAAAAATGGATTTCACTAAAAATGTCTTTCTTGCTGTGCGCTCAGGCGCCGTCCCGATCGGTAGTGTGCGTTATGTCCGGTGGTTCTGTTTGGCATCTGATAGCTGATCGAGTCGCTCTCAGTGTGTAAGGTGCTGGGTTTTGGACTATTTGGCTTGCCTGCCTATCGTGAGGAGCGGTTGCTTGGTGCGTACAGTTCCGGACGGCGAAAAATTGAATTCCAATAAAATATTATTCTCTCAACACGTGCTACTTTATCATGCGTTAAGGAATGTTCTCTCGCACACAGCGGTTCGGGCGATGTCTCTACTCGACGTTTCGGCACTGCTTGATTCGTTCTCGAAGACAGCAGTGCAGTTCGGGGGGTGGGGATGTTGCTTAATATACGCGGCGGTGCATGAGTGGTAAATAGGCCATTGGGGTTGGCAGGCTCTGTGCTAGCGCATCGAACTGTCGTACCTTGAGGCCACTCAGTGGTGTCCCGGAGGCGTATTGCTATGTCGGGCGGGGATGGTTTCTGTGTTGCATACCCGCGCAGTGGAATGGAATTTTGTTGCCAAGAAACATTCGTCCCGTGCCCTTTTAGGGGCGTCGGATGAACCATGCAGCAGCTCTCGTGTGCCGGGCATGCCTTTTTGGCTTCTCTGGCACATGTGAAGGTGCTCGTGCTCTCGGATGCGGAATGCTTTTGCGAGAGGAGGGATTGAGTTTCCTTTATGTGTTCTCGCTGTCCCTACATAAGAACCACCGTCCTTTCCGCACAGTGGCCTTGGTTGCTGCGGTGTACTATGTCTGCTTGCGGGTTAGGACGGCATGGAGGAATGCTACCTGGTTGATCCTGCCAGTAGTCATATGCTTGTCTCAAAGATTAAGCCATGCATGTGTAAGTATGAACTAATTCAGACTGTGAAACTGCGAATGGCTCATTAAATCAGTTATAGTTTGTTTGATGGTACCTGCTACTCGGATAACCGTAGTAATTCTAGAGCTAATACGTGCAACAAACCCCGACTTCTGGAAGGGATGCATTTATTAGATAAAAGGTCGACGCGGGCTCTGCCCGTTGCTCTGATGATTCATGATAACTCGACGGATCGCACGGCCATCGTGCTGGCGACGCATCATTCAAATTTCTGCCCTATCAACTTTCGATGGTAGGATAGAGGCCTACCATGGTGGTGACGGGTGACGGAGAATTAGGGTTCGATTCCGGAGAGGGAGCCTGAGAAACGGCTACCACATCCAAGGAAGGCAGCAGGCGCGCAAATTACCCAATCCTGACACGGGGAGGTAGTGACAATAAATAACAATACCGGGCTCTTCGAGTCTGGTAATTGGAATGAGTACAATCTAAATCCCTTAACGAGGATCCATTGGAGGGCAAGTCTGGTGCCAGCAGCCGCGGTAATTCCAGCTCCAATAGCGTATATTTAAGTTGTTGCAGTTAAAAAGCTCGTAGTTGGACCTTGGGTTGGGTCGACCGGTCCGCCTTACGGTGTGCACCTGTCGGCTCGTCCCTTCTGCCGGCGATGCGCTCCTGGCCTTAACTGGCCGGGTCGTGCCTCCGGCGCTGTTACTTTGAAGAAATTAGAGTGCTCAAAGCAAGCCTACGCTCTGTATACATTAGCATGGGATAACATCATAGGATTTCGGTCCTATTCTGTTGGCCTTCGGGATCGGAGTAATGATTAACAGGGACAGTCGGGGGCATTCGTATTTCATAGTCAGAGGTGAAATTCTTGGATTTATGAAAGACGAACAACTGCGAAAGCATTTGCCAAGGATGTTTTCATTAATCAAGAACGAAAGTTGGGGGCTCGAAGACGATCAGATACCGTCCTAGTCTCAACCATAAACGATGCCGACCAGGGATCGGCGGATGTTGCTTTTAGGACTCCGCCGGCACCTTATGAGAAATCAAAGTCTTTGGGTTCCGGGGGGAGTATGGTCGCAAGGCTGAAACTTAAAGGAATTGACGGAAGGGCACCACCAGGAGTGGAGCCTGCGGCTTAATTTGACTCAACACGGGGAAACTTACCAGGTCCAGACATAGTAAGGATTGACAGACTGAGAGCTCTTTCTTGATTCTATGGGTGGTGGTGCATGGCCGTTCTTAGTTGGTGGAGCGATTTGTCTGGTTAATTCCGTTAACGAACGAGACCTCAGCCTGCTAACTAGCTATGCGGAGGTATCCCTCCGCGGCCAGCTTCTTAGAGGGACTATGGCCTTCTAGGCCAAGGAAGTTTGAGGCAATAACAGGTCTGTGATGCCCTTAGATGTTCTGGGCCGCACGCGCGCTACACTGATGTATTCAACGAGTCTATAGCCTTGGCCGACAGGCCCGGGTAATCTTTGAAATTTCATCGTGATGGGGATAGATCATTGCAATTGTTGGTCTTCAACGAGGAATTCCTAGTAAGCGCGAGTCATCAGCTCGCGTTGACTACGTCCCTGCCCTTTGTACACACCGCCCGTCGCTCCTACCGATTGAATGGTCCGGTGAAGTGTTCGGATCGCGGCGACGTGGGCGGTTCGCCGCCGGCGACGTCGCGAGAAGTCCACTGAACCTTATCATTTAGAGGAAGGAGAAGTCGTAACAAGGTTTCCGTAGGTGAACCTGCGGAAGGATCATTGTCGAAACCTGCCAGCAGAATGACCCGCGAACGTGTTTATAAATCGAGGGGCCGCTGCAGGATTCATCCAGCGATGGCACCTCACTAGGGCCCTGGCAGGGGATGCGGTGCGGTGGGATCCACCGTTCCCTGCGATCTCCTGTTTGCGGCCTATTAACAAAACCCCGGCGCCGTACGCGCCAAGGAATTGTAAAAAAAGATTGTGCAGCCCGATCGCACTGGCAACGGTGTGGCGGGTTTCACTGCGCTTTGAGAACCAAAATGACTCTCGGCAACGGATATCTCGGCTCTCGCATCGATGAAGAACGCAGCGAAATGCGATACTTGGTGTGAATTGCAGGATCCCGCGAACCATCGAGTCTTTGAACGCAAGTTGCGCCCGAAGCCTTTCGGCCGAGGGCACGTCTGCCTGGGTGTCACTCAAACGTCGCTCCAAACCCCTTCCATCGGGAGGGGTATGCGGGGCGGATGCTGGCCTCCCGTGTGCGTATCGCTCGCGGTTGGCCGAAATTCCTAGTCCTCGGCACGACGCCACGGAATCGGTGGTTGCAAGACCCTCGGAGAAAGCCTTGTGCGCTTGTAAGCCCTTCCGGACCATGAGACCCCAGAGCGTACCTAGCACTGCGACCCCAGGTCAGGCGGGATTACCCGCTGAGTTTAAGCATATCAATAAGCGGAGGAAAAGAAACTTACCAGGATTCCCCTAGTAACGGCGAGCGAACCGGGAAGAGCCCAGCTTGAGAATCGTGCGCCTGCGGCGTTCGAATTGTAGTCTGGAGAAGCGTCCTCAGCGGCGGACCGGGCCCAAGTCCCCTGGAAGGGGGCGCCGGAGAGGGTGAGAGCCCCGTCGTGCCCGGACCCTGTCGCACCACGAGGCGCTGTCTACGAGTCGGGTTGTTTGGGAATGCAGCCCAAATCGGGCGGTAAATTCCGTCCAAGGCTAAATATGGGCGAGAGACCGATAGCGAACAAGTACCGCGAGGGAAAGATGAAAAGGACTTTGAAAAGAGAGTCAAAGAGTGCTTGAAATTGTCGGGAGGGAAGCGGATGGGGGCCGGCGATGCGCCCCGGTCGGATGTGGAACGGTGACAAGCCGGTCCGCCGATCGGCTCGGGGCGCGGACCGATACGGATTGAGGCGGCGGCGTAAGCCCAGGAATTTGAAACGCCTGTGGAGATGCCGTCGCAGCAATCGTGGAAAGCAGCACGCGCCGTCTCGGCGTGCCTCGGCACCTGCGTGCTACTGGTGTCGGCCAGCGGGCTCCCCATTCGGCCCGTCTTGAAACACGGACCAAGGAGTCTGACATGTGTGCGAGTCAACGGGCGAGTAAACCCGTAAGGCGCAAGGAAGCTGACTGGCGGGATCCCCTAGAGGGTTGCACCGCCGACCGACCTTGATCTTCTGAGAAGGGTTCGAGTGAGAGCATGCCTGTCGGGACCCGAAAGATGGTGAACTATGCCTGAGCGGGGCGAAGCCAGAGGAAACTCTGGTGGAGGCCCGCAGCGATACTGACGTGCAAATCGTTCGTCTGACTTGGGTATAGGGGCGAAAGACTAATCGAACCGTCTAGTAGCTGGTTCCCTCCGAAGTTTCCCTCAGGATAGCTGGAGCTCGGAACGAGTTCTATCGGGTAAAGCCAATGATTAGAGGCATCGGGGGCGCAACGCCCTCGACCTATTCTCAAACTTTAAATAGGTAGGACGGCGCGGCTGCTTCGTTGAGCCGCGCCACGGAATCGAGAGCTCCAAGTGGGCCATTTTTGGTAAGCAGAACTGGCGATGCGGGATGAACCGGAAGCCGGGTTACGGTGCCCAACTGCGCGCTAACCTAGAACCCACAAAGGGTGTTGGTCGATTAAGACAGCAGGACGGTGGTCATGGAAGTCGAAATCCGCTAAGGAGTGTGTAACAACTCACCTGCCGAATCAACTAGCCCCGAAAATGGATGGCGCTTAAGCGCGCGACCTATACCCGGCCGTCGGGGCAAGAGCCAGGCCCCGATGAGTAGGAGGGCGCGGCGGTCGCTGCAAAACCCAGGGCGCGAGCCCGGGCGGAGCGGCCGTCGGTGCAGATCTTGGTGGTAGTAGCAAATATTCAAATGAGAACTTTGAAGGCCGAAGAGGGGAAAGGTTCCATGTGAACGGCACTTGCACATGGGTTAGTCGATCCTAAGAGACGGGGGAAGCCCGTCCGACAGCGCGTCCGCGCGCGAGCTTCGAAAGGGAATCGGGTTAAAATTCCCGAACCGGGACGCGGCGGCTGACGGCAACGTTAGGGAGTCCGGAGACGTCGGCGGGGGCCTCGGGAAGAGTTATCTTTTCTGTTTAACAGCCCGCCCACCCTGGAAACGACTCAGTCGGAGGTAGGGTCCAGCGGCTGGAAGAGCACCGCACGTCGCGCGGTGTCCGGTGCGCCCCCGGCGGCCCGTGAAAATCCGGAGGACCGAGTGCCATCCACGCCCGGTCGTACTCATAACCGCATCAGGTCTCCAAGGTGAACAGCCTCTGGTCGATGGAACAATGTAGGCAAGGGAAGTCGGCAAAATGGATCCGTAACCTCGGGAAAAGGATTGGCTCTGAGGGCTGGGCCCGGGGGTCCCAGTCCCGAACCCGTCGGCTGTCGGCGGACTGCTCGAGCTGCTCCCGCGGCAAGAGCGGGTCGCTGCGTGCCGGCCGGGGGACGGATTGGGAACGGCCCCTCCGGGGGCCTTCCCCGGGCGTCGAACAGTCGACTCAGAACTGGTACGGACAAGGGGAATCCGACTGTTTAATTAAAACAAAGCATTGCGATGGTCCCTGCGGATGCTAACGCAATGTGATTTCTGCCCAGTGCTCTGAATGTCAAAGTGAAGAAATTCAACCAAGCGCGGGTAAACGGCGGGAGTAACTATGACTCTCTTAAGGTAGCCAAATGCCTCGTCATCTAATTAGTGACGCGCATGAATGGATTAACGAGATTCCCACTGTCCCTGTCTACTATCCAGCGAAACCACAGCCAAGGGAACGGGCTTGGCAGAATCAGCGGGGAAAGAAGACCCTGTTGAGCTTGACTCTAGTCCGACTTTGTGAAATGACTTGAGAGGTGTAGTATAAGTGGGAGCCGGAAACGGCGATAGTGAAATACCACTACTTTTAACGTTATTTTACTTATTCCGTGAATCGGAGGCGGGGCATTGCCCCTCTTTTTGGACCAAAGGCCGCTTCGCGGTCGATCCGGGCGGAAGACATTGTCAGGTGGGGAGTTTGGCTGGGGCGGCACATCTGTTAAAAGATAACGCAGGTGTCCTAAGATGAGCTCAACGAGAACAGAAATCTCGTGTGGAACAAAAGGGTAAAAGCTCGTTTGATTCTGATTTCCAGTACGAATACGAACCGTGAAAGCGTGGCCTATCGATCCTTTAGACCTTCGGAATTTGAAGCTAGAGGTGTCAGAAAAGTTACCACAGGGATAACTGGCTTGTGGCAGCCAAGCGTTCATAGCGACGTTGCTTTTTGATCCTTCGATGTCGGCTCTTCCTATCATTGTGAAGCAGAATTCACCAAGTGTTGGATTGTTCACCCACCAATAGGGAACGTGAGCTGGGTTTAGACCGTCGTGAGACAGGTTAGTTTTACCCTACTGATGACGGTGTCGCGATGGTAATTCAACCTAGTACGAGAGGAACCGTTGATTCGCACAATTGGTCATCGCGCTTGGTTGAAAAGCCAGTGGCGCGAAGCTACCGTGCGCTGGATTATGACTGAACGCCTCTAAGTCAGAATCCGGGCCAGAAGCGATGCATGCGTCCGCCGCTCGTTTGCCGACCCTCAGTAGGGGCCATCCGGCCCCCAAAGGCACGTGTCGTTGGCTAAGCCCTCGCGGCAGACAAGCCGTGCGGGCAGCCTTGAAGTACAATTCCCACCGGGCGGCGGGCAGAATCCTTTGCAGACGACTTAAATACGCGACGGGGTATTGTAAGTGGCAGAGTGGCCTTGCTGCCACGATCCACTGAGATTCAGCCCTTTGTCGCTCCGATTCGTCCCTCCCTCCCAAGAAACTTTTTCCAACTCAATAGAATATCGAGCGGAGGCTGGGGTCTCGATTTCCGCTCGTAGAAAGAGGGCCAAGGAGAGAACCCCGGTATTGCATATGGCACGGGGGAAGCAAGAGATTAAGCACACCGCGGGGTGGAAAAAGCATCTGCGCACGGGCAGGTTAGAAAAACATACAAGCGATGCATCCCTTCAATGTCCCTCGGCGTTGACCGCTTGTTCATTTTTTTTTCCGGCTTAATGCGTACGTTTCGAACCACTCGTACACCAAGGCTGCCGCGGCCACGCGTGCCTCACCGGTCGATGCGGTCTACTGCCTTCACAGGCGAGGCAGGAGCAGCATCGAATTCCTGCCCCGGCACCACGCACGAGGCCGGCGGTGCCTACCTCTCGTGCGCAAGCGAGACCGATGCCAGCGCGGCTGACTGGCCTGAATAGGAGAGGCCAGTGCTAGTCCGCGCACGCCACATGCCCCTTCAAAGTTTCCCCGCATCGCTCGAGAATTTTACGGCAGGCCCGTTAAACATCTCCATCGGGAGAGAGAATTTGCATCTCCGCCCCAAGCGTCGATGCTAGTCCGCGCACGCCACATGCCCCTTCAAAGTTTCCCCGCACCGCTCGAGAATTTTATGGCAGACCCGTTAAATATCTCCACCGGGAGAGAAAATTTGCATCTCCGCCCCCAACCGTCGAGAAAAGATTAACACTTTTACCACCCCGAGGGTTGTACACACAGCCTCCTGAAACAAAGGGGCGGCAAAAACGCGTTTCGCCTCCAGTCCACGCTCGGTCTTTCGGAACGGGTTCAACGGCAGTTGATTTTATACCGGGTTGCCAAATTCTCACGAGGGCTCTGTATTCTTTTTCGTTTGTCAAGTTCCCGGCAGTCTTAAATACGAGAAAGGCCATCTCGAAGGTCCCGGCATTCTCACGAAGGTTCTAAATATATATTTTCCTTTTTTCAAGAGCTAGGACTTCTTAAAAATGGAAAAAATTATCCCCCGTGTCCGGAACGTCCCGGTAATGCATGTAGATTTCCCTTGAACCATGCTGGTCAGAATTTCTGCAAAAAAATGAAGGGTCTACGTATCTTCCGT-----------------------------------------------------------------------------------------CCCGAACTGTGGTCACAATTTCAGCAAAAAAAAAAAAATGAAGTCTTTACATAC

Euphorbia neocymosa TTTTTTCAAGTTTTCCGGGCCTTCCTCGAAATGGAAAAAGATATCGCCCATGTCCGAAAAGTCCCGGTATTACATGTAAATGTCCCTTTGAAAATGTTCGAACGGTGGTCGAAATTTCAACAAAAAGTGAAGGCTTTACATTTTTTTTTCCATTTTTTCCGAAACCCTTAAATATGGAAAAAATAACCCCCATGTCCGGAAAGCCCCGGTATTACATGTAAATGTCCCCTAAATAATGCTCGAACCGTGGTAAAAATTTCAGCTAAAAATGAAGGCTTTACATATTTTTTGCCATTTTCCCCGTAATTCCTAAAAATGGAAAAAATACCCCCGTCGGGCGGATTGGCCCGATATTACACGTCAATTTCCCCCAAATCATGCTCGAACTGGGGTCGGAATTTCGGCAAAAAAGGAAGGCTCTGCATATTTTTTTCCATTTTTCCCGGAATTCTTAAAAATGGGAAAAATATCCCCGACGTCCGGAAACTCCCGATATCAGGTGTAAATGTCCCTCAAATCATGCTCGAACCGTGGTAAAAATTTCAGCGAAAAATGAAGGCGCTGCATATTTTTTTCCATTTTTCCAGTGCCCGGAATTCACAAAAATGGAAAAAATAGCCCCGTTGTCCGGGAAGTCCCGGGATTGCATGTAAATTTCCCTTAAATCATGCTCGAACTATGGTAAAAATTTCAGAAAAAATCTCCAAGTATAGAGCAGTTTTTGGGGGGGTGTGGCTCCTGGAACAAATCGATGTCCGTTCCTCCCAGAGCTGGATTTGTCATAAGAAATACTATAGGGGCTGCGCCAGCTCTCAACCTTGGCCCGCACTGGGCTGCGGGCCCGTGTTGGGTGCCTCCAGCATGCTCTCCCGCATTCAAGCGCATCACTCGGTCTCCCAGAAAGTCCCGACCCGCCGCCCCACCGAACGGAAGAATATCGTGGAAAAGCTAAGCCCAAAAGCACCACACGCGCGCGCGCGACGGCGCGGCGCGTTCGCGATTTAGGCGATTTAGGCACTTGGCACTTAGCACGTAGCACGAAGCGCGAGGCGCGGGGCCAATCGGCGGAACATAGCAGAAAATATCCCGCAAAATTTATAAACGAGTAACGACACGGCATGAAAAAGGAGGTAGCTAGCAAAAATTTAAAAAAAAAGAAAGGAACATGGAGGCCAACCCGAAGTTCAGGCAAGAGTGCAGGGCGGCCTGGCCCGCCTTCGTGTGCGGGAGCAGGCGGGACAGCGTGCAGCGCTGTCCCGCATTCCTGCCTGTGCCTGGCGAGGCTAGGCAGGACGGCCGGCGTGCTGGCCCGCCCGCGTGTGCAGGAGGGCCTGCCAGTGGGAGGGCTGGCCCGCCTGGGCAGGGCTGGCAAGCTGCCCTGGCTCGCCTGAGTGCATGGCGGGGGAGGGCAAGCTGGCCAGCACGCTGCCCTGCCCGGCTCGCCAGCCAGCACCGCGCAGGCAGGCCGCCAACCAGAGGTCAGCATAGGCATGCCAGCACGCCCAGCCGAGCTCTGCCGGCGGTGCTCACCCCGTCCGCGCAAAGGAGGCTAGGCCAGCGTGCTGTCTCGCCTGGCCGAGGCTCAGAATGGCTTCGCTGGGCCCGATCCAAATTTCGTTTGCAAAACCTTCGCACAGATAATAAGTTTGCCCGCACCATATTTTTAATACTGACTTTACCCATTTATTAGGAATTTTGTACCGAGTTTGGTATGCATTTGTTGAGTTTATATATTTTTTTGTATTTTTACGATTCTCGGACTTCGTAAATATGGAAAAAATACCTCCCGTGGGTAAAAGGTTACAATATTATATGGAAAAATCCCTTAATTCATGTTCTATCTACAGTCAAAATTTCAGGAAAAAACTCGAAGGTTTGACCGGTTTTTAAGGGGGTGTGGCTCCTGGAACAAATCGATGTCTGTTCCTCCCAGAGCTGGAAATGCTATAAGGACTACTATAGGGGGGTACACCTGCTCTCAACCCAGGCCACCACTGGGGCTGCGGGCCCGTGTTGGGTGCTTGCCGCATGCTCACCCCCTATCATGCGGGGAATTCAGCCACTAAGAACGTCCCGACCCGCCGCCCCTCCTCCGGCCGCCGGCCGCCGCCCCGCGCGGTGGTCGGAAAATCCAAAATTCTTAGAACGCTGAATTCCGCACCCCGGGAGCCCCTTTTCCCCCTGCCATTGTCGGAACTCGTTCGAATTGGGGGTAAAGTGGGTTTTCGGAGGCAACAACGTGCCCCGCGGCGCTGTCCCGATCGCTAGTGTGTGCCTACTCGTAGTTTTGTCTTGCATCGGATAGCTGATCGAGCTGCTCTCAGTGTGTATGTTGCTCGACTTCAAGCTGCTTTGGAAGCACGCCTAGAGTGAGGGGTAGCCGCTTGCTGCGCACGGTATCGGACGAGGGAAAAAGGAATTTCGGAAAAAAACGTTTCCGTGCCGTGCGCGATGTTGCTACATCCGTAGTTGCTTGCTGCGTGCGGTGTGGGGCGATGTAAAAAATGGAATTTGAAAATAAATTGTTTTCACGGAGTGCGCTTCGGCGTTGCCCCGATCGATAGTGCGTGTTGCTCGGTGGATTTGTTTGGCATTGGATAGCCGATCGAGTTGCTCTCGGTGTGAAGGTGCTGGACGTTGGTCCACTCGGCAGCCCGCCTTGAGTGAGGGGCAGTTGGTTGATGCGTGCGTTGCCAAGTGGCGAAAAAATGGATTTCACTAAAAATGTCTTTCTTGCTGTGCGCTCAGGCGCCGTCCCGATCGGTAGTGTGCGTTATGTCCGGTGGTTCTGTTTGGCATCTGATAGCTGATCGAGTCGCTCTCAGTGTGTAAGGTGCTGGGTTTTGGACTATTCGGCTTGCCTGCCTATCGTGAGGAGCGGTTGCTTGGTGCGTACAGTTCCGGACGGCGAAAAATTGAATTCCAATAAGATATTATTTTCTCAACACGTGCTACTTTATCATGCGGTAAGGAATGTTCTCTCGCACACAGCGGTTCGGGCGATGTCTCTACTCGACGTTTCGGCACTGCTTGATTCGTTCTCGAAGACAGCAGTGCAGTTCGGGGGGTGGGGATGTTGCTCAATATACGCGGCGGTGCATGAGTGGTAAATAGGCCATTGGGGTTGGCAGGCTCTGTGCTAGCGCATCGAACTGTCGTACCTTGAGGCCACTCAGTGGTGTCCCGGAGGCGTATTGCTATGTCGGGCGGGGATGGTTTCTGTGTTGCATACCCGCGCAGTGGAATGGAATTTTGTTGCCAAGAAACATTCGTCCCGTGCCCTTTTAGGGGCGTCGGATGAACCATGCAGCAGCTCTCGTGTGCCGGGCATGCCTTTTTGGCTTCTCTGGCACATGTGAAGGTGCTCGTGCTCTCGGATGCGGAATGCTTTTGCGAGAGGAGGGATTGAGTTTCCTTTATGTGTTCTCGCTGTCCCTACATAAGAACCACCGTCCTTTCCGCACAGTGGCCTTGGTTGCTGCGGTGTACTATGTCTGCTTGCGGGTTAGGACGGCATGGAGGAATGCTACCTGGTTGATCCTGCCAGTAGTCATATGCTTGTCTCAAAGATTAAGCCATGCATGTGTAAGTATGAACTAATTCAGACTGTGAAACTGCGAATGGCTCATTAAATCAGTTATAGTTTGTTTGATGGTACCTGCTACTCGGATAACCGTAGTAATTCTAGAGCTAATACGTGCAACAAACCCCGACTTCTGGAAGGGATGCATTTATTAGATAAAAGGTCGACGCGGGCTCTGCCCGTTGCTCTGATGATTCATGATAACTCGACGGATCGCACGGCCATCGTGCTGGCGACGCATCATTCAAATTTCTGCCCTATCAACTTTCGATGGTAGGATAGAGGCCTACCATGGTGGTGACGGGTGACGGAGAATTAGGGTTCGATTCCGGAGAGGGAGCCTGAGAAACGGCTACCACATCCAAGGAAGGCAGCAGGCGCGCAAATTACCCAATCCTGACACGGGGAGGTAGTGACAATAAATAACAATACCGGGCTCTTCGAGTCTGGTAATTGGAATGAGTACAATCTAAATCCCTTAACGAGGATCCATTGGAGGGCAAGTCTGGTGCCAGCAGCCGCGGTAATTCCAGCTCCAATAGCGTATATTTAAGTTGTTGCAGTTAAAAAGCTCGTAGTTGGACCTTGGGTTGGGTCGACCGGTCCGCCTTACGGTGTGCACCTGTCGGCTCGTCCCTTCTGCCGGCGATGCGCTCCTGGCCTTAACTGGCCGGGTCGTGCCTCCGGCGCTGTTACTTTGAAGAAATTAGAGTGCTCAAAGCAAGCCTACGCTCTGTATACATTAGCATGGGATAACATCATAGGATTTCGGTCCTATTCTGTTGGCCTTCGGGATCGGAGTAATGATTAACAGGGACAGTCGGGGGCATTCGTATTTCATAGTCAGAGGTGAAATTCTTGGATTTATGAAAGACGAACAACTGCGAAAGCATTTGCCAAGGATGTTTTCATTAATCAAGAACGAAAGTTGGGGGCTCGAAGACGATCAGATACCGTCCTAGTCTCAACCATAAACGATGCCGACCAGGGATCGGCGGATGTTGCTTTTAGGACTCCGCCGGCACCTTATGAGAAATCAAAGTCTTTGGGTTCCGGGGGGAGTATGGTCGCAAGGCTGAAACTTAAAGGAATTGACGGAAGGGCACCACCAGGAGTGGAGCCTGCGGCTTAATTTGACTCAACACGGGGAAACTTACCAGGTCCGGACATAGTAAGGATTGACAGACTGAGAGCTCTTTCTTGATTCTATGGGTGGTGGTGCATGGCCGTTCTTAGTTGGTGGAGCGATTTGTCTGGTTAATTCCGTTAACGAACGAGACCTCAGCCTGCTAACTAGCTATGCGGAGGTATCCCTCCGCGGCCAGCTTCTTAGAGGGACTATGGCCTTCTAGGCCAAGGAAGTTTGAGGCAATAACAGGTCTGTGATGCCCTTAGATGTTCTGGGCCGCACGCGCGCTACACTGATGTATTCAACGAGTCTATAGCCTTGGCCGACAGGCCCGGGTAATCTTTGAAATTTCATCGTGATGGGGATAGATCATTGCAATTGTTGGTCTTCAACGAGGAATTCCTAGTAAGCGCGAGTCATCAGCTCGCGTTGACTACGTCCCTGCCCTTTGTACACACCGCCCGTCGCTCCTACCGATTGAATGGTCCGGTGAAGTGTTCGGATCGCGGCGACGTGGGCGGTTCGCCGCCGGCGACGTCGCGAGAAGTCCACTGAACCTTATCATTTAGAGGAAGGAGAAGTCGTAACAAGGTTTCCGTAGGTGAACCTGCGGAAGGATCATTGTCGAAACCTGCCAGCAGAATGACCCGCGAACGTGTTTATAAATCGAGGGGCCGCTGCAGGATTCATCCAGCGATGGCACCTCACTAGGGCCCTGGCAGGGGATGCGGTGCGGTGGGATCCACCGTTCCCTGCGATCTCCTGTTTGCGGCCTATTAACAAAACCCCGGCGCCGTACGCGCCAAGGAATTGTAAAAAAAGATTGTGCAGCCCGATCGCACTGGCAACGGTGTGGCGGGTTTCACTGCGCTTTGAGAACCAAAATGACTCTCGGCAACGGATATCTCGGCTCTCGCATCGATGAAGAACGCAGCGAAATGCGATACTTGGTGTGAATTGCAGGATCCCGCGAACCATCGAGTCTTTGAACGCAAGTTGCGCCCGAAGCCTTTCGGCCGAGGGCACGTCTGCCTGGGTGTCACTCAAACGTCGCTCCAAACCCCTTCCATCGGGAGGGGTATGCGGGGCGGATGCTGGCCTCCCGTGTGCGTATCGCTCGCGGTTGGCCGAAATTCCTAGTCCTCGGCACGACGCCACGGAATCGGTGGTTGCAAGACCCTCGGAGAAAGCCTTGTGCGCTTGTAAGCCCTTTCGGACCATGAGACCCCAGAGCGTACCTAGCACTGCGACCCCAGGTCAGGCGGGATTACCCGCTGAGTTTAAGCATATCAATAAGCGGAGGAAAAGAAACTTACCAGGATTCCCCTAGTAACGGCGAGCGAACCGGGAAGAGCCCAGCTTGAGAATCGTGCGCCTGCGGCGTTCGAATTGTAGTCTGGAGAAGCGTCCTCAGCGGCGGACCGGGCCCAAGTCCCCTGGAAGGGGGCGCCGGAGAGGGTGAGAGCCCCGTCGTGCCCGGACCCTGTCGCACCACGAGGCGCTGTCTACGAGTCGGGTTGTTTGGGAATGCAGCCCAAATCGGGCGGTAAATTCCGTCCAAGGCTAAATATGGGCGAGAGACCGATAGCGAACAAGTACCGCGAGGGAAAGATGAAAAGGACTTTGAAAAGAGAGTCAAAGAGTGCTTGAAATTGTCGGGAGGGAAGCGGATGGGGGCCGGCGATGCGCCCCGGTCGGATGTGGAACGGTGACAAGCCGGTCCGCCGATCGGCTCGGGGCGCGGACCGATACGGATTGAGGCGGCGGCGTAAGCCCAGGAATTTGAAACGCCTGTGGAGATGCCGTCGCAGCAATCGTGGAAAGCAGCACGCGCCGTCTCGGCGTGCCTCGGCACCTGCGTGCTACTGGTGTCGGCCAGCGGGCTCCCCATTCGGCCCGTCTTGAAACACGGACCAAGGAGTCTGACATGTGTGCGAGTCAACGGGCGAGTAAACCCGTAAGGCGCAAGGAAGCTGACTGGCGGGATCCCCTAGAGGGTTGCACCGCCGACCGACCTTGATCTTCTGAGAAGGGTTCGAGTGAGAGCATGCCTGTCGGGACCCGAAAGATGGTGAACTATGCCTGAGCGGGGCGAAGCCAGAGGAAACTCTGGTGGAGGCCCGCAGCGATACTGACGTGCAAATCGTTCGTCTGACTTGGGTATAGGGGCGAAAGACTAATCGAACCGTCTAGTAGCTGGTTCCCTCCGAAGTTTCCCTCAGGATAGCTGGAGCTCGGAACGAGTTCTATCGGGTAAAGCCAATGATTAGAGGCATCGGGGGCGCAACGCCCTCGACCTATTCTCAAACTTTAAATAGGTAGGACGGCGCGGCTGCTTCGTTGAGCCGCGCCACGGAATCGAGAGCTCCAAGTGGGCCATTTTTGGTAAGCAGAACTGGCGATGCGGGATGAACCGGAAGCCGGGTTACGGTGCCCAACTGCGCGCTAACCTAGAACCCACAAAGGGTGTTGGTCGATTAAGACAGCAGGACGGTGGTCATGGAAGTCGAAATCCGCTAAGGAGTGTGTAACAACTCACCTGCCGAATCAACTAGCCCCGAAAATGGATGGCGCTTAAGCGCGCGACCTATACCCGGCCGTCGGGGCAAGAGCCAGGCCCCGATGAGTAGGAGGGCGCGGCGGTCGCTGCAAAACCCAGGGCGCGAGCCCGGGCGGAGCGGCCGTCGGTGCAGATCTTGGTGGTAGTAGCAAATATTCAAATGAGAACTTTGAAGGCCGAAGAGGGGAAAGGTTCCATGTGAACGGCACTTGCACATGGGTTAGTCGATCCTAAGAGACGGGGGAAGCCCGTCCGACAGCGCGTCCGCGCGCGAGCTTCGAAAGGGAATCGGGTTAAAATTCCCGAACCGGGACGCGGCGGCTGACGGCAACGTTAGGGAGTCCGGAGACGTCGGCGGGGGCCTCGGGAAGAGTTATCTTTTCTGTTTAACAGCCCGCCCACCCTGGAAACGACTCAGTCGGAGGTAGGGTCCAGCGGCTGGAAGAGCACCGCACGTCGCGCGGTGTCCGGTGCGCCCCCGGCGGCCCGTGAAAATCCGGAGGACCGAGTGCCATCCACGCCCGGTCGTACTCATAACCGCATCAGGTCTCCAAGGTGAACAGCCTCTGGTCGATGGAACAATGTAGGCAAGGGAAGTCGGCAAAATGGATCCGTAACCTCGGGAAAAGGATTGGCTCTGAGGGCTGGGCCCGGGGGTCCCAGTCCCGAACCCGTCGGCTGTCGGCGGACTGCTCGAGCTGCTCCCGCGGCAAGAGCGGGTCGCTGCGTGCCGGCCGGGGGACGGATTGGGAACGGCCCCTCTGGGGGCCTTCCCCGGGCGTCGAACAGTCGACTCAGAACTGGTACGGACAAGGGGAATCCGACTGTTTAATTAAAACAAAGCATTGCGATGGTCCCTGCGGATGCTAACGCAATGTGATTTCTGCCCAGTGCTCTGAATGTCAAAGTGAAGAAATTCAACCAAGCGCGGGTAAACGGCGGGAGTAACTATGACTCTCTTAAGGTAGCCAAATGCCTCGTCATCTAATTAGTGACGCGCATGAATGGATTAACGAGATTCCCACTGTCCCTGTCTACTATCCAGCGAAACCACAGCCAAGGGAACGGGCTTGGCAGAATCAGCGGGGAAAGAAGACCCTGTTGAGCTTGACTCTAGTCCGACTTTGTGAAATGACTTGAGAGGTGTAGTATAAGTGGGAGCCGGAAACGGCGATAGTGAAATACCACTACTTTTAACGTTATTTTACTTATTCCGTGAATCGGAGGCGGGGCATTGCCCCTCTTTTTGGACCAAAGGCGGCTTCGCGGTCGATCCGGGCGGAAGACATTGTCAGGTGGGGAGTTTGGCTGGGGCGGCACATCTGTTAAAAGATAACGCAGGTGTCCTAAGATGAGCTCAACGAGAACAGAAATCTCGTGTGGAACAAAAGGGTAAAAGCTCGTTTGATTCTGATTTCCAGTACGAATACGAACCGTGAAAGCGTGGCCTATCGATCCTTTAGACCTTCGGAATTTGAAGCTAGAGGTGTCAGAAAAGTTACCACAGGGATAACTGGCTTGTGGCAGCCAAGCGTTCATAGCGACGTTGCTTTTTGATCCTTCGATGTCGGCTCTTCCTATCATTGTGAAGCAGAATTCACCAAGTGTTGGATTGTTCACCCACCAATAGGGAACGTGAGCTGGGTTTAGACCGTCGTGAGACAGGTTAGTTTTACCCTACTGATGACGGTGTCGCGATGGTAATTCAACCTAGTACGAGAGGAACCGTTGATTCGCACAATTGGTCATCGCGCTTGGTTGAAAAGCCAGTGGCGCGAAGCTACCGTGCGCTGGATTATGACTGAACGCCTCTAAGTCAGAATCCGGGCCAGAAGCGATGCATGCGTCCGCCGCTCGTTTGCCGACCCTCAGTAGGGGCCATCCGGCCCCCAAAGGCACGTGTCGTTGGCTAAGCCCTCGCGGCAGACAAGCCGTGCGGGCAGCCTTGAAGTACAATTCCCACCGGGCGGCGGGCAGAATCCTTTGCAGACGACTTAAATACGCGACGGGGTATTGTAAGTGGCAGAGTGGCCTTGCTGCCACGATCCACTGAGATTCAGCCCTTTGTCGCTCCGATTCGTCCCTCCCTCCCAAGAAACTTTTTCCATCTCAATAGAATATCGAGCGGAGGCTGGGGTCTCGATTTCCGCTCGTAGAAAGAGGGCCAAGGAGAGAACCCCGGTATTGCATATGGCACGGGGGAAGCAAGAGATTAAGCACACCGCGGGGTGGAAAAAGCATCTGCGCACGGGCAGGTTAGAAAAACATACAAGCGATGCATCCCTTCAATGTCCCTCGGCGTTGACCGCTTGTTCATTTTTTTTTCCGGCTTGGTGCGTACGTTTCGAACCACTCGTACACCAAGGCTGCCGCGGCCACGCGTGCCTCACCGGTCGATGCGGTCTACTGCCTTCACAGGCGAGGCAGGAGCAGCATCGAATTCCTGCCCCGGCACCACGCACGAGGCCGGCGGTGCCTACCTCTCGTGCGCAAGCGAGACCGATGCCAGCGCGGCTGACTGGCCTGAATAGGAGAGGCCAGTGCTAGTCCGCGCACGCCACATGCCCCTTCAAAGTTTCCCCGCATCGCTCGAGAATTTTACGGCAGGCCCGTTAAACATCTCCATCGGGAGAGAGAATTTGCATCTCCGCCCCAAGCGTCGATGCTAGTCCGCGCACGCCACATGCCCCTTCAAAGTTTCCCCGTACCGCTCGAGAATTTTATGGCAGACCCGTTAAATATCTCCATCGGGAGAGAAAATTTGCATCTCCGCCCCCAACCGTCGAGAAAAGATTAACACTTTTACCACCCCGAGGGTTGTACACACAGCCTCCTGAAACAAAGGGGCGGCAAGAACGCGTTTCGCCTCCAGTCCACGCTCGGTCTTTCGGAACGGGTTCAACGGCAGTTGATTTTATACCGAGTTGCCAAATTCTCACGAGGGCTCTGTATTCTTTTTCGTTTGTCAAGTTCCCGGCAGTCTTAAATACGAGAAAGGCCATCTCGAAGGTCCCGGCATTTTCACGAAGGTTCTAAATATATATTTTCCTTTTTTCAAGAGCTAGGACTTCTTAAAAATGGAAAAAATTATCCCCCGTGTCCGGAAAGTCCCGATAATGCATGTAGATTTCCCTTGAACCATGCTGGTCAGAATTTCTGCAAAAAAAGGAAGGGTCTACGTATCTTCTGTCCATTTTTCCCGGACTCCCTAAAAATGGGAAAAATACCCCCCATGTTCGGAAAGTCCCGATATTACATGTGAATATCCCTTAAATCACACCCGAACTGTGGTCACAATTTCAGCAAAAAAAAAAAAATGAAGTCTTTACATAT

Euphorbia neoglabrata TTTTTTCAAGTTTTCCGGGCCTTCCTCGAAATGGAAAAAGATATCGCCCATGTCCGAAAAGTCCCGGTATTACATGTAAATGTCCCTTTGAAAATGTTCGAACGGTGGTCGAAATTTCAACAAAAAGTGAAGGCTTTACATTTTTTTTTCCATTTTTTCCGAAACCCTTAAATATGGAAAAAATAACCCCCATGTCCGGAAAGCCCCGGTATTACATGTAAATGTCCCCTAAATAATGCTCGAACCGTGGTAAAAATTTCAGCTAAAAATGAAGGCTTTACATATTTTTTGCCATTTTCCCCGTAATTCCTAAAAATGGAAAAAATACCCCCGTCGGGCGGATTGGCCCGATATTACACGTCAATTTCCCCCAAATCATGCTCGAACTGGGGTCGGAATTTCGGCAAAAAAGGAAGGCTCTGCATATTTTTTTCCATTTTTCCCGGAATTCTTAAAAATGGGAAAAATATCCCCGACGTCCGGAAACTCCCGATATCAGGTGTAAATGTCCCTCAAATCATGCTCGAACCGTGGTAAAAATTTCAGCGAAAAATGAAGGCGCTGCATATTTTTTTCCATTTTTCCAGTGCCCGGAATTCACAAAAATGGAAAAAATAGCCCCGTTGTCCGGGAAGTCCCGGGATTGCATGTAAATTTCCCTTAAATCATGCTCGAACTATGGTAAAAATTTCAGAAAAAATCTCCAAGTATAGAGCAGTTTTTGGGGGGGTGTGGCTCCTGGAACAAATCGATGTCCGTTCCTCCCAGAGCTGGATTTGTCATAAGAAATACTATAGGGGCTGCGCCAGCTCTCAACCTTGGCCCGCACTGGGCTGCGGGCCCGTGTTGGGTGCCTCCCGCATGCTCTCCCGCATTCAAGCGCATCACTCGGTCTCCCAGAAAGTCCCGACCCGCCGCCCCACCGAACGGAAGAATATCGTGGAAAAGCTAAGCCCAAAAGCACCACACGCGCGCGCGCGACGGCGCGGCGCGTTCGCGATTTAGGCGATTTAGGCACTTGGCACTTAGCACGTAGCACGAAGCGCGAGGCGCGAGGCCAATCGGCGGAACATAGCAGAAAATATCCCGCAAAATTTATAAACGAGTAACGACACGGCATGAAAAAGGAGGTAGCTAGCAAAAATTTAAAAAAAAAGAAAGGAACATGGAGGCCAACCCGAAGTTCAGGCAAGAGTGCAGGGCGGCCTGGCCCGCCTTCGTGTGCGGGAGCAGGCGGGACAGCGTGCAGCGCTGTCCCGCATTCCTGCCTGTGCCTGGCGAGGC-------------------------------------------------------------------------------------------GCCCTGGCTCGCCTGAGTGCATGGCGGGGGAGGGCAAGCTGGCCAGCACGCTGCCCTGCCTGGCTCGCCAGCCAGCACCGGGCAGGCAGGCCGCCAACCAGAGGTCAGCATAGGCATGCCAGCACGCCCAGCCGAGCTCTGCCGGCGGTGCTCACCCCGTCCGCGCAAAGGAGGCTAGGTCAGCGTGCTGTCTCGCCTGGCCGAGGCTCAGAATGGCTTCGCTGGGCCCGATCCAAATTTCGTTTGCAAAACCTTCGCACAGATAATAAGTTTGCCCGCACCATATTTTTAATACTGACTTTACCCATTTATTAGGAATTTTGTACCGAGTTTGGTATGCATTTGTTGAGTTTATATATTTTTTTGTATTTTTACGATTCTCGGACTTCGTAAATATGGAAAAAATACCTCCCGTGGGTAAAAGGTTACAATATTATATGGAAAAATCCCTTAATTCATGTTCTATCTACAGTCAAAATTTCAGGAAAAAACTCGAAGGTTTGACCGGTTTTTAAGGGGGTGTGGCTCCTGGAACAAATCGATGTCTGTTCCTCCCAGAGCTGGAAATGCTATAAGGACTACTATAGGGGGGTACACCTGCTCTCAACCCAGGCCACCACTGGGGCTGCGGGCCCGTGTTGGGTGCTTGCCGCATGCTCACCCCCTATCATGCGGGGAATTCAGCCACTAAGAACGTCCCGACCCGCCGCCCCTCCT-------CCGGCCGCCGCCCCGCGCGGTGGTCGGAAAATCCGAAATTCTTAGAACGCTGAATTCCGCACCCCGAGAGCCCCTTTTCCCCCTGCCATTGTCGGAACTCGTTCGAATTGGGGGTAAAGTGGGTTTTCGGAGGCAACAACGTGCCCCGCGGCGCTGTCCCGATCGCTAGTGTGTGCCTACTCGTAGTTTTGTCTTGCATCGGATAGCTGATCGAGCTGCTCTCAGTGTGTATGTTGCTCGACTTCAAGCTGCTTTGGAAGCACGCCTAGAGTGAGGGGTAGCCGCTTGCTGCGCACGGTATCGGACGAGGGAAAAAGGAATTTCGGAAAAAAACGTTTCCGTGCCGTGCGCGATGTTGCTACATCCGTAGTTGCTTGCTGCGTGCGGTGTGGGGCGATGTAAAAAATGGAATTTGAAAATAAATTGTTTTCACGGAGTGCGCTTCGGCGTTGCCCCGATCGATAGTGCGTGTTGCTCGGTGGATTTGTTTGGCATTGGATAGCCGATCGAGTTGCTCTCGGTGTGAAGGTGCTGGACGTTGGTCCACTCGGCAGCCCGCCTTGAGTGAGGGGCAGTTGGTTGATGCGTGCGTTGCCAAGTGGCGAAAAAATGGATTTCACTAAAAATGTCTTTCTTGCTGTGCGCTCAGGCGCCGTCCCGATCGGTAGTGTGCGTTATGTCCGGTGGTTCTGTTTGGCATCTGATAGCTGATCGAGTCGCTCTCAGTGTGTAAGGTGCTGGGTTTTGGACTATTCGGCTTGCCTGCCTATCGTGAGGAGCGGTTGCTTGGTGCGTACAGTTCCGGACGGCGAAAAATTGAATTCCAATAAGATATTATTTTCTCAACACGTGCTACTTTATCATGCGGTAAGGAATGTTCTCTCGCACACAGCGGTTCGGGCGATGTCTCTACTCGACGTTTCGGCACTGCTTGATTCGTTCTCGAAGACAGCAGTGCAGTTCGGGGGGTGGGGATGTTGCTCAATATACGCGGCGGTGCATGAGTGGTAAATAGGCCATTGGGGTTGGCAGGCTCTGTGCTAGCGCATCGAACTGTCGTACCTTGAGGCCACTCAGTGGTGTCCCGGAGGCGTATTGCTATGTCGGGCGGGGATGGTTTCTGTGTTGCATACCCGCGCAGTGGAATGGAATTTTGTTGCCAAGAAACATTCGTCCCGTGCCCTTTTAGGGGCGTCGGATGAACCATGCAGCAGCTCTCGTGTGCCGGGCATGCCTTTTTGGCTTCTCTGGCACATGTGAAGGTGCTCGTGCTCTCGGATGCGGAATGCTTTTGCGAGAGGAGGGATTGAGTTTCCTTTATGTGTTCTCGCTGTCCCTACATAAGAACCACCGTCCTTTCCGCACAGTGGCCTTGGTTGCTGCGGTGTACTATGTCTGCTTGCGGGTTAGGACGGCATGGAGGAATGCTACCTGGTTGATCCTGCCAGTAGTCATATGCTTGTCTCAAAGATTAAGCCATGCATGTGTAAGTATGAACTAATTCAGACTGTGAAACTGCGAATGGCTCATTAAATCAGTTATAGTTTGTTTGATGGTACCTGCTACTCGGATAACCGTAGTAATTCTAGAGCTAATACGTGCAACAAACCCCGACTTCTGGAAGGGATGCATTTATTAGATAAAAGGTCGACGCGGGCTCTGCCCGTTGCTCTGATGATTCATGATAACTCGACGGATCGCACGGCCATCGTGCTGGCGACGCATCATTCAAATTTCTGCCCTATCAACTTTCGATGGTAGGATAGAGGCCTACCATGGTGGTGACGGGTGACGGAGAATTAGGGTTCGATTCCGGAGAGGGAGCCTGAGAAACGGCTACCACATCCAAGGAAGGCAGCAGGCGCGCAAATTACCCAATCCTGACACGGGGAGGTAGTGACAATAAATAACAATACCGGGCTCTTCGAGTCTGGTAATTGGAATGAGTACAATCTAAATCCCTTAACGAGGATCCATTGGAGGGCAAGTCTGGTGCCAGCAGCCGCGGTAATTCCAGCTCCAATAGCGTATATTTAAGTTGTTGCAGTTAAAAAGCTCGTAGTTGGACCTTGGGTTGGGTCGACCGGTCCGCCTTACGGTGTGCACCTGTCGGCTCGTCCCTTCTGCCGGCGATGCGCTCCTGGCCTTAACTGGCCGGGTCGTGCCTCCGGCGCTGTTACTTTGAAGAAATTAGAGTGCTCAAAGCAAGCCTACGCTCTGTATACATTAGCATGGGATAACATCATAGGATTTCGGTCCTATTCTGTTGGCCTTCGGGATCGGAGTAATGATTAACAGGGACAGTCGGGGGCATTCGTATTTCATAGTCAGAGGTGAAATTCTTGGATTTATGAAAGACGAACAACTGCGAAAGCATTTGCCAAGGATGTTTTCATTAATCAAGAACGAAAGTTGGGGGCTCGAAGACGATCAGATACCGTCCTAGTCTCAACCATAAACGATGCCGACCAGGGATCGGCGGATGTTGCTTTTAGGACTCCGCCGGCACCTTATGAGAAATCAAAGTCTTTGGGTTCCGGGGGGAGTATGGTCGCAAGGCTGAAACTTAAAGGAATTGACGGAAGGGCACCACCAGGAGTGGAGCCTGCGGCTTAATTTGACTCAACACGGGGAAACTTACCAGGTCCAGACATAGTAAGGATTGACAGACTGAGAGCTCTTTCTTGATTCTATGGGTGGTGGTGCATGGCCGTTCTTAGTTGGTGGAGCGATTTGTCTGGTTAATTCCGTTAACGAACGAGACCTCAGCCTGCTAACTAGCTATGCGGAGGTATCCCTCCGCGGCCAGCTTCTTAGAGGGACTATGGCCTTCTAGGCCAAGGAAGTTTGAGGCAATAACAGGTCTGTGATGCCCTTAGATGTTCTGGGCCGCACGCGCGCTACACTGATGTATTCAACGAGTCTATAGCCTTGGCCGACAGGCCCGGGTAATCTTTGAAATTTCATCGTGATGGGGATAGATCATTGCAATTGTTGGTCTTCAACGAGGAATTCCTAGTAAGCGCGAGTCATCAGCTCGCGTTGACTACGTCCCTGCCCTTTGTACACACCGCCCGTCGCTCCTACCGATTGAATGGTCCGGTGAAGTGTTCGGATCGCGGCGACGTGGGCGGTTCGCCGCCGGCGACGTCGCGAGAAGTCCACTGAACCTTATCATTTAGAGGAAGGAGAAGTCGTAACAAGGTTTCCGTAGGTGAACCTGCGGAAGGATCATTGTCGAAACCTGCCAGCAGAATGACCCGCGAACGTGTTTATAAATCGAGGGGCCGCTGCAGGATTCATCCAGCGATGGCACCTCACTGGGGCCCTGGCAGGGGATGCGGTGCGGTGGGATCCACCGTTCCCTGCGATCTCCTGTTTGCGGCCTATTAACAAAACCCCGGCGCCGTACGCGCCAAGGAATTGTAAAAAAAGATTGTGCAGCCCGATCGCACTGGCAACGGTGTGGCGGGTTTCACTGCGCTTTGAGAACCAAAATGACTCTCGGCAACGGATATCTCGGCTCTCGCATCGATGAAGAACGCAGCGAAATGCGATACTTGGTGTGAATTGCAGGATCCCGCGAACCATCGAGTCTTTGAACGCAAGTTGCGCCCGAAGCCTTTCGGCCGAGGGCACGTCTGCCTGGGTGTCACTCAAACGTCGCTCCAAACCCCTTCCATCGGGAGGGGTATGCGGGGCGGATGCTGGCCTCCCGTGTGCGTATCGCTCGCGGTTGGCCGAAATTCCTAGTCCTCGGCACGACGCCACGGAATCGGTGGTTGCAAGACCCTCGGAGAAAGCCTTGTGCGCTTGTAAGCCCTTTCGGACCATGAGACCCCAGAGCGTACCTAGCACTGCGACCCCAGGTCAGGCGGGATTACCCGCTGAGTTTAAGCATATCAATAAGCGGAGGAAAAGAAACTTACCAGGATTCCCCTAGTAACGGCGAGCGAACCGGGAAGAGCCCAGCTTGAGAATCGTGCGCCTGCGGCGTTCGAATTGTAGTCTGGAGAAGCGTCCTCAGCGGCGGACCGGGCCCAAGTCCCCTGGAAGGGGGCGCCGGAGAGGGTGAGAGCCCCGTCGTGCCCGGACCCTGTCGCACCACGAGGCGCTGTCTACGAGTCGGGTTGTTTGGGAATGCAGCCCAAATCGGGCGGTAAATTCCGTCCAAGGCTAAATATGGGCGAGAGACCGATAGCGAACAAGTACCGCGAGGGAAAGATGAAAAGGACTTTGAAAAGAGAGTCAAAGAGTGCTTGAAATTGTCGGGAGGGAAGCGGATGGGGGCCGGCGATGCGCCCCGGTCGGATGTGGAACGGTGACAAGCCGGTCCGCCGATCGGCTCGGGGCGCGGACCGATACGGATTGAGGCGGCGGCGTAAGCCCAGGAATTTGAAACGCCTGTGGAGATGCCGTCGCAGCAATCGTGGAAAGCAGCACGCGCCGTCTCGGCGTGCCTCGGCACCTGCGTGCTACTGGTGTCGGCCAGCGGGCTCCCCATTCGGCCCGTCTTGAAACACGGACCAAGGAGTCTGACATGTGTGCGAGTCAACGGGCGAGTAAACCCGTAAGGCGCAAGGAAGCTGACTGGCGGGATCCCCTAGAGGGTTGCACCGCCGACCGACCTTGATCTTCTGAGAAGGGTTCGAGTGAGAGCATGCCTGTCGGGACCCGAAAGATGGTGAACTATGCCTGAGCGGGGCGAAGCCAGAGGAAACTCTGGTGGAGGCCCGCAGCGATACTGACGTGCAAATCGTTCGTCTGACTTGGGTATAGGGGCGAAAGACTAATCGAACCGTCTAGTAGCTGGTTCCCTCCGAAGTTTCCCTCAGGATAGCTGGAGCTCGGAACGAGTTCTATCGGGTAAAGCCAATGATTAGAGGCATCGGGGGCGCAACGCCCTCGACCTATTCTCAAACTTTAAATAGGTAGGACGGCGCGGCTGCTTCGTTGAGCCGCGCCACGGAATCGAGAGCTCCAAGTGGGCCATTTTTGGTAAGCAGAACTGGCGATGCGGGATGAACCGGAAGCCGGGTTACGGTGCCCAACTGCGCGCTAACCTAGAACCCACAAAGGGTGTTGGTCGATTAAGACAGCAGGACGGTGGTCATGGAAGTCGAAATCCGCTAAGGAGTGTGTAACAACTCACCTGCCGAATCAACTAGCCCCGAAAATGGATGGCGCTTAAGCGCGCGACCTATACCCGGCCGTCGGGGCAAGAGCCAGGCCCCGATGAGTAGGAGGGCGCGGCGGTCGCTGCAAAACCCAGGGCGCGAGCCCGGGCGGAGCGGCCGTCGGTGCAGATCTTGGTGGTAGTAGCAAATATTCAAATGAGAACTTTGAAGGCCGAAGAGGGGAAAGGTTCCATGTGAACGGCACTTGCACATGGGTTAGTCGATCCTAAGAGACGGGGGAAGCCCGTCCGACAGCGCGTCCGCGCGCGAGCTTCGAAAGGGAATCGGGTTAAAATTCCCGAACCGGGACGCGGCGGCTGACGGCAACGTTAGGGAGTCCGGAGACGTCGGCGGGGGCCTCGGGAAGAGTTATCTTTTCTGTTTAACAGCCCGCCCACCCTGGAAACGACTCAGTCGGAGGTAGGGTCCAGCGGCTGGAAGAGCACCGCACGTCGCGCGGTGTCCGGTGCGCCCCCGGCGGCCCGTGAAAATCCGGAGGACCGAGTGCCATCCACGCCCGGTCGTACTCATAACCGCATCAGGTCTCCAAGGTGAACAGCCTCTGGTCGATGGAACAATGTAGGCAAGGGAAGTCGGCAAAATGGATCCGTAACCTCGGGAAAAGGATTGGCTCTGAGGGCTGGGCCCGGGGGTCCCAGTCCCGAACCCGTCGGCTGTCGGCGGACTGCTCGAGCTGCTCCCGCGGCAAGAGCGGGTCGCTGCGTGCCGGCCGGGGGACGGATTGGGAACGGCCCCTCTGGGGGCCTTCCCCGGGCGTCGAACAGTCGACTCAGAACTGGTACGGACAAGGGGAATCCGACTGTTTAATTAAAACAAAGCATTGCGATGGTCCCTGCGGATGCTAACGCAATGTGATTTCTGCCCAGTGCTCTGAATGTCAAAGTGAAGAAATTCAACCAAGCGCGGGTAAACGGCGGGAGTAACTATGACTCTCTTAAGGTAGCCAAATGCCTCGTCATCTAATTAGTGACGCGCATGAATGGATTAACGAGATTCCCACTGTCCCTGTCTACTATCCAGCGAAACCACAGCCAAGGGAACGGGCTTGGCAGAATCAGCGGGGAAAGAAGACCCTGTTGAGCTTGACTCTAGTCCGACTTTGTGAAATGACTTGAGAGGTGTAGTATAAGTGGGAGCCGGAAACGGCGATAGTGAAATACCACTACTTTTAACGTTATTTTACTTATTCCGTGAATCGGAGGCGGGGCATTGCCCCTCTTTTTGGACCAAAGGCCGCTTCGCGGTCGATCCGGGCGGAAGACATTGTCAGGTGGGGAGTTTGGCTGGGGCGGCACATCTGTTAAAAGATAACGCAGGTGTCCTAAGATGAGCTCAACGAGAACAGAAATCTCGTGTGGAACAAAAGGGTAAAAGCTCGTTTGATTCTGATTTCCAGTACGAATACGAACCGTGAAAGCGTGGCCTATCGATCCTTTAGACCTTCGGAATTTGAAGCTAGAGGTGTCAGAAAAGTTACCACAGGGATAACTGGCTTGTGGCAGCCAAGCGTTCATAGCGACGTTGCTTTTTGATCCTTCGATGTCGGCTCTTCCTATCATTGTGAAGCAGAATTCACCAAGTGTTGGATTGTTCACCCACCAATAGGGAACGTGAGCTGGGTTTAGACCGTCGTGAGACAGGTTAGTTTTACCCTACTGATGACGGTGTCGCGATGGTAATTCAACCTAGTACGAGAGGAACCGTTGATTCGCACAATTGGTCATCGCGCTTGGTTGAAAAGCCAGTGGCGCGAAGCTACCGTGCGCTGGATTATGACTGAACGCCTCTAAGTCAGAATCCGGGCCAGAAGCGATGCATGCGTCCGCCGCTCGTTTGCCGACCCTCAGTAGGGGCCATCCGGCCCCCAAAGGCACGTGTCGTTGGCTCAGCCCTCGCGGCAGACAAGCCGTGCGGGCAGCCTTGAAGTACAATTCCCACCGGGCGGCGGGCAGAATCCTTTGCAGACGACTTAAATACGCGACGGGGTATTGTAAGTGGCAGAGTGGCCTTGCTGCCACGATCCACTGAGATTCAGCCCTTTGTCGCTCCGATTCGTCCCTCCCTCCCAAGAAACTTTTTCCATCTCAATAGAATATCGAGCGGAGGCTGGGGTCTCGATTTCCGCTCGTAGAAAGAGGGCCAAGGAGAGAACCCCGGTATTGCATATGGCACGGGGGAAGCAAGAGATTAAGCACACCGCGGGGTGGAAAAAGCATCTGCGCACGGGCAGGTTAGAAAAACATACAAGCGATGCATCCCTTCAATGTCCCTCGGCGTTGACCGCTTGTTCATTTTTTTTTCCGGCTTGATGCGTACGTTTCGAACCACTCGTACACCAAGGCTGCCGCGGCCACGCGTGCCTCACCGGTCGATGCGGTCTACTGCCTTCACAGGCGAGGCAGGAGCAGCATCGAATTCCTGCCCCGGCACCACGCACGAGGCCGGCGGTGCCTACCTCTCGTGCGCAAGCGAGACCGATGCCAGCGCGGCTGACTGGCCTGAATAGGAGAGGCCAGTGCTAGTCCGCGCACGCCACATGCCCCTTCAAAGTTTCCCCGCATCGCTCGAGAATTTTACGGCAGGCCCGTTAAACATCTCCATCGGGAGAGAGAATTTGCATCTCCGCCCCAAGCGTCGATGCTAGTCCGCGCACGCCACATGCCCCTTCAAAGTTTCCCCGTACCGCTCGAGAATTTTATGGCAGACCCGTTAAATATCTCCATCGGGAGAGAAAATTTGCATCTCCGCCCCCAACCGTCGAGAAAAGATTAACACTTTTACCACCCCGAGGGTTGTACACACAGCCTCCTGAAACAAAGGGGCGGCAAAAACGCGTTTCGCCTCCAGTCCACGCTCGGTCTTTCGGAACGGGTTCAACGGCAGTTGATTTTATACCGAGTTGCCAAATTCTCACGAGGGCTCTGTATTCTTTTTCGTTTGTCAAGTTCCCGGCAGTCTTAAATACGAGAAAGGCCATCTCGAAGGTCCCGGCATTTTCACGAAGGTTCTAAATATATATTTTCCTTTTTTCAAGAGCTAGGACTTCTTAAAAATGGAAAAAATTATCCCCCGTGTCCGGAAAGTCCCGATAATGCATGTAGATTTCCCTTGAACCATGCTGGTCAGAATTTCTGCAAAAAAAGGAAGGGTCTACGTATCTTCTGTCCATTTTTCCCGGACTCCCTAAAAATGGGAAAAATACCCCCCATGTTCGGAAAGTCCCGATATTACATGTAAATATCCCTTAAATCACACCCGAACTGTGGTCACAATTTCAGCAAAAAAAAAAAAATGAAGTCTTTACATAT

Euphorbia neoglaucescens TTTTTTCAAGTTTTCCGGGCCTTCCTCAAAATGGAAAAAGATATCGCCCATGTCCGAAAAGTCCCGGTATTACATGTAAATGTCCCTTTGAAAATGTTCGAACGGTGGTCGAAATTTCAACAGAAAGTGAAGGCTTTACATTTTTTTTTCCATTTTTTCCGAAACCCTTAAATATGGAAAAAATAACCCCCATGTCCGGAAAGCCCCGGTATTACATGTAAATGTCCCCTAAATAATGCTCGAACCGTGGTAAAAATTTCAGCTAAAAATGAAGGCTTTACATATTTTTTGCCATTTCCCCCGTAATTCCTAAAAATGGAAAAAATGCCCCCGTCGGGCGGATTGGCCCGATATTACACGTCAATTTCCCCCAAATCATGCTCGAACTGGGGTCGGAATTTCGGCAAAAAAGGAAGGCTCTGCATATTTTTTTCCATTTTTCCCGGAATTCTTAAAAATGGGAAAAATATCGCCCACGTCCGGAAACTCCCGATATCAAGTGTAAATGTCCCTCAAATCATGCTCGAACCGTGGTAAAAATTTCAGCGAAAAATGAAGGCGTTGCATATTTTTTTCCATTTTTCCAGTGCCCGGAATTCACAAAAATGGAAAAAATAGCCCCGTTGTCCGGGAAGTCCCGGGATTGCATGTAAATTTCCCTTAAATCATGCTCGAACTATGGTAAAAATTTCAGAAAAAATCTCCAAGTATAGAGCAGTTTTTGGGGGGGTGTGGCTCCTGGAACAAATCGATGTCCGTTCCTCCCAGAGCTGGATTTGTCATAAGAAATACTATAGGGGCTGCGCCAGCTCTCAACCTTGGCCCGCACTGGGCTGCGGGCCCGTGGTGGGTGCCTCCCGCATGCTCTCCCGCATTCAAGCGCATCACTCGGTCTCCCAGAAAGTCCCGACCCGCCGCCCCACCGAACGGAAGAATATCGTGGAAAAGCTAAGCCCAAAAGCACCACACGCGCGCGCGCGACGGCGCGGCGCGTTCGCGATTTAGGCGATTTAGGCACTTGGCACTTAGCACGTAGCACGAAGCGCGAGGCGCGAGGCCAATCGGCGGAACATAGCAGAAAATATCCCGCAAAATTTATAAACGAGTAACGACACGGCATGAAAAAGAAGGTAGCTAGCAAAAATTTTAAAAAAAAGAAAGGAACATGAAGGCCAACCCGAAGTTCAGGCAAGAGTGCAGGGCGGCCTGGCCCGCCTTCGTGTGCGGGAGCAGGCGGGACAGCGTGCAGCGCTGTCCCGCATTCCTGCCTGTGCCTGGCGAGGCTAGGCAGGACGGCCGGCGTGCTGGCCCGCCCGCGTGTGCAGGAGGGACTGCCAGTGGGAGGGCTGGCCCGCCTGGGCAGGGCTGGCAAGCTGCCCTGGCTCGCCTGAGTGCATGGCGGGGGAGGGCAAGCTGGCCAGCACGCAGCCCTGCCTGGCTCGCCAGCCAGCACCACGCAGGCAGGCCGCCAACCAGAGGTCAGCATAGGCATGCCAGCACGCCCAGCCGAGCTCTGCCGGCGGTGCTCACCCAGTCCGCGAAAAGGAGGCTAGGTCAGCGTGCTGTCTCGCCTGGCCGAGGCTCAGGATGGCTTCGCTGGGCCCGATCCAAATTTCGTTTGCAAAACCTTCGCACAGATAATAAGTTTTCCCGCACCATATTTTTTATACTGACTTTTACCATTTATTAGGAATTTTGTACCGAGTTCGTGATGCATTTGTTGAGTTTATATATTTTTTTGTATTTTTACGATTCTCGGACTTCGTAAATATGGAAAAAATACCTCCCGTGGGTAAAAGGTTACAATATTATATGGAAAAATCCCTTAATTCATGTTCTATCTACAGTCAAAATTTCAGGAAAAAACTCGAAGGTTTGACCGGTTTTTAAGGGGGTGTGGCTCCTGGAACAAATCGATGTCTGTTCCTCCCAGAGCTGGAAATGCTATAAGGACTACTATAGGGGGGTACACCTGCTCTCAACCCAGGCCACCACTGGGGCTGCGGGCCCGTGTAGGGTGCTTGCCGCATGCTCACCCCCTATCATGCGGGGAATTCAGCCACTAAGAACGTCCCGACCCGCCGCCCCTCCTCCGGCCGCCGGCCGCCGCCCCGCGCGGTGGCCGGAAAATCCAAAATTCTTAGAACGCTGAATTCCGCACCCCGAGAGCCCCTTTTCCCCCTGCCATTGTCGGAACTCGTTCGAATTGGGGGTAAAGTGGGTTTTCGGAGGCAACAACGTGCCCCGCGGCGCTGTCCCGATCGCTAGTGTGTGCCTACTCGTAGTTTTGTCTTGCATCGGATAGCTGATTGAGCTGCTCTCAGTGTGTATGGTGCTCGACTTCAAGCTGCTTTGGAAGCACGCCTAGAGTGAGGGGTAGCCGCTTGCTGCGCACGGTATCGGACGAGGGAAAAAGGAATTTCGGAAAAAAACGTTTCCGTGCCGTGCGCGATGTTGCTACATCCGTAGCTGCTTGCTGCGTGCGGTGTGGGGCGATGTAAAAAATGGAATTTGAAAATAAAATGTTTTCACGGAGTGCGCTTCGGCGTTGCCCCGATCGATAGTGCGTGTTGCTCGGTGGATTTGTTTGGCATTGGATAGCCGATCGAGTTGCTCTCGGTGTGAAGGTGCTGGACGTTGGTCCACTCGGCAGCCCGCCTTGAGTGAGGGGCAGTTGGTTGATGCGTGCGGTGCCAAGTGGCGAAAAAATGGATTTCACTAAAAATGTCTTTCTTGCTGTGCGCTCAGGCGCCGTCCCGATCGGTAGTGTGCGTTATGTCCGGTGGTTCTGTTTGGCATCTGATAGCTGATCGAGTCGCTCTCAGTGTGTAAGGTGCTGGGTTTTGGACTATTTGGCTTGCCTGCCTATCGTGAGGAGCGGTTGCTTGGTGCGTACAGTTCCGGACGGCGAAAAATTGAATTCCAATAAAATATTATTCTCTCAACACGTGCTACTTTATCATGCGGTAAGGAATGTTCTCTCGCACACAGCGGTTCGGGCGATGTCTCTACTCGACGTTTCGGCACTGCTTGATTCGTTCTCGAAGACAGCAGTGCAGTTCGGGGGGTGGGGATGTTGCTCAATATACGCGGCGGTGCATGAGTGGTAAATAGGCCATTGGGGTTGGCAGGCTCTGTGCTAGCGCATCGAACTGTCGTACCTTGAGGCCACTCAGTGGTGTCCCGGAGGCGTATTGCTATGTCGGGCGGGGATGGTTTCTGTGTTGCATACCCGCGCAGTGGAATGGAATTTTGTTGCCAAGAAACATTCGTCCCGTGCCCTTTTAGGGGCGTCGGATGAACCATGCAGCAGCTCTCGTGTGCCGGGCATGCCTTTTTGGCTTCTCTGGCACATGTGAAGGTGCTCGTGCTCTCGGATGCGGAATGCTTTTGCGAGAGGAGGGATTGAGTTTCCTTTATGTGTTCTCGCTGTCCCTACATAAGAACCACCGTCCTTTCCGCACAGTGGCCTTGGTTGCTGCGGTGTACTATGTCTGCTTGCGGGTTAGGACGGCATGGAGGAATGCTACCTGGTTGATCCTGCCAGTAGTCATATGCTTGTCTCAAAGATTAAGCCATGCATGTGTAAGTATGAACTAATTCAGACTGTGAAACTGCGAATGGCTCATTAAATCAGTTATAGTTTGTTTGATGGTACCTGCTACTCGGATAACCGTAGTAATTCTAGAGCTAATACGTGCAACAAACCCCGACTTCTGGAAGGGATGCATTTATTAGATAAAAGGTCGACGCGGGCTCTGCCCGTTGCTCTGATGATTCATGATAACTCGACGGATCGCACGGCCATCGTGCTGGCGACGCATCATTCAAATTTCTGCCCTATCAACTTTCGATGGTAGGATAGAGGCCTACCATGGTGGTGACGGGTGACGGAGAATTAGGGTTCGATTCCGGAGAGGGAGCCTGAGAAACGGCTACCACATCCAAGGAAGGCAGCAGGCGCGCAAATTACCCAATCCTGACACGGGGAGGTAGTGACAATAAATAACAATACCGGGCTCTTCGAGTCTGGTAATTGGAATGAGTACAATCTAAATCCCTTAACGAGGATCCATTGGAGGGCAAGTCTGGTGCCAGCAGCCGCGGTAATTCCAGCTCCAATAGCGTATATTTAAGTTGTTGCAGTTAAAAAGCTCGTAGTTGGACCTTGGGTTGGGTCGACCGGTCCGCCTTACGGTGTGCACCTGTCGGCTCGTCCCTTCTGCCGGCGATGCGCTCCTGGCCTTAACTGGCCGGGTCGTGCCTCCGGCGCTGTTACTTTGAAGAAATTAGAGTGCTCAAAGCAAGCCTACGCTCTGTATACATTAGCATGGGATAACATCATAGGATTTCGGTCCTATTCTGTTGGCCTTCGGGATCGGAGTAATGATTAACAGGGACAGTCGGGGGCATTCGTATTTCATAGTCAGAGGTGAAATTCTTGGATTTATGAAAGACGAACAACTGCGAAAGCATTTGCCAAGGATGTTTTCATTAATCAAGAACGAAAGTTGGGGGCTCGAAGACGATCAGATACCGTCCTAGTCTCAACCATAAACGATGCCGACCAGGGATCGGCGGATGTTGCTTTTAGGACTCCGCCGGCACCTTATGAGAAATCAAAGTCTTTGGGTTCCGGGGGGAGTATGGTCGCAAGGCTGAAACTTAAAGGAATTGACGGAAGGGCACCACCAGGAGTGGAGCCTGCGGCTTAATTTGACTCAACACGGGGAAACTTACCAGGTCCAGACATAGTAAGGATTGACAGACTGAGAGCTCTTTCTTGATTCTATGGGTGGTGGTGCATGGCCGTTCTTAGTTGGTGGAGCGATTTGTCTGGTTAATTCCGTTAACGAACGAGACCTCAGCCTGCTAACTAGCTATGCGGAGGTATCCCTCCGCGGCCAGCTTCTTAGAGGGACTATGGCCTTCTAGGCCAAGGAAGTTTGAGGCAATAACAGGTCTGTGATGCCCTTAGATGTTCTGGGCCGCACGCGCGCTACACTGATGTATTCAACGAGTCTATAGCCTTGGCCGACAGGCCCGGGTAATCTTTGAAATTTCATCGTGATGGGGATAGATCATTGCAATTGTTGGTCTTCAACGAGGAATTCCTAGTAAGCGCGAGTCATCAGCTCGCGTTGACTACGTCCCTGCCCTTTGTACACACCGCCCGTCGCTCCTACCGATTGAATGGTCCGGTGAAGTGTTCGGATCGCGGCGACGTGGGCGGTTCGCCGCCGGCGACGTCGCGAGAAGTCCACTGAACCTTATCATTTAGAGGAAGGAGAAGTCGTAACAAGGTTTCCGTAGGTGAACCTGCGGAAGGATCATTGTCGAAACCTGCCAGCAGAATGACCCGCGAACGTGTTTATAAATCGAGGGGCCGCTGCAGGATTCATCCAGCGATGGCACCTCACTAGGGCCCTGGCAGGGGATGCGGTGCGGTGGGATCCACCGTTCCCTGCGATCTCCTGTTTGCGGCCTATTAACAAAACCCCGGCGCCGTACGCGCCAAGGAATTGTAAAAAAAGATTGTGCAGCCCGATCGCACTGGCAACGGTGTGGCGGGTTTCACTGCGCTTTGAGAACCAAAATGACTCTCGGCAACGGATATCTCGGCTCTCGCATCGATGAAGAACGCAGCGAAATGCGATACTTGGTGTGAATTGCAGGATCCCGCGAACCATCGAGTCTTTGAACGCAAGTTGCGCCCGAAGCCTTTCGGCCGAGGGCACGTCTGCCTGGGTGTCACTCAAACGTCGCTCCAAACCCCTTCCATCGGGAGGGGTATGCGGGGCGGATGCTGGCCTCCCGTGTGCGTATCGCTCGCGGTTGGCCGAAATTCCTAGTCCTCGGCACGACGCCACGGAATCGGTGGTTGCAAGACCCTCGGAGAAAGCCTTGTGCGCTTGTAAGCCCTTTCGGACCATGAGACCCCAGAGCGTACCTAGCACTGCGACCCCAGGTCAGGCGGGATTACCCGCTGAGTTTAAGCATATCAATAAGCGGAGGAAAAGAAACTTACCAGGATTCCCCTAGTAACGGCGAGCGAACCGGGAAGAGCCCAGCTTGAGAATCGTGCGCCTGCGGCGTTCGAATTGTAGTCTGGAGAAGCGTCCTCAGCGGCGGACCGGGCCCAAGTCCCCTGGAAGGGGGCGCCGGAGAGGGTGAGAGCCCCGTCGTGCCCGGACCCTGTCGCACCACGAGGCGCTGTCTACGAGTCGGGTTGTTTGGGAATGCAGCCCAAATCGGGCGGTAAATTCCGTCCAAGGCTAAATATGGGCGAGAGACCGATAGCGAACAAGTACCGCGAGGGAAAGATGAAAAGGACTTTGAAAAGAGAGTCAAAGAGTGCTTGAAATTGTCGGGAGGGAAGCGGATGGGGGCCGGCGATGCGCCCCGGTCGGATGTGGAACGGTGACAAGCCGGTCCGCCGATCGGCTCGGGGCGCGGACCGATACGGATTGAGGCGGCGGCGTAAGCCCAGGAATTTGAAACGCCTGTGGAGATGCCGTCGCAGCAATCGTGGAAAGCAGCACGCGCCGTCTCGGCGTGCCTCGGCACCTGCGTGCTACTGGTGTCGGCCAGCGGGCTCCCCATTCGGCCCGTCTTGAAACACGGACCAAGGAGTCTGACATGTGTGCGAGTCAACGGGCGAGTAAACCCGTAAGGCGCAAGGAAGCTGACTGGCGGGATCCCCTAGAGGGTTGCACCGCCGACCGACCTTGATCTTCTGAGAAGGGTTCGAGTGAGAGCATGCCTGTCGGGACCCGAAAGATGGTGAACTATGCCTGAGCGGGGCGAAGCCAGAGGAAACTCTGGTGGAGGCCCGCAGCGATACTGACGTGCAAATCGTTCGTCTGACTTGGGTATAGGGGCGAAAGACTAATCGAACCGTCTAGTAGCTGGTTCCCTCCGAAGTTTCCCTCAGGATAGCTGGAGCTCGGAACGAGTTCTATCGGGTAAAGCCAATGATTAGAGGCATCGGGGGCGCAACGCCCTCGACCTATTCTCAAACTTTAAATAGGTAGGACGGCGCGGCTGCTTCGTTGAGCCGCGCCACGGAATCGAGAGCTCCAAGTGGGCCATTTTTGGTAAGCAGAACTGGCGATGCGGGATGAACCGGAAGCCGGGTTACGGTGCCCAACTGCGCGCTAACCTAGAACCCACAAAGGGTGTTGGTCGATTAAGACAGCAGGACGGTGGTCATGGAAGTCGAAATCCGCTAAGGAGTGTGTAACAACTCACCTGCCGAATCAACTAGCCCCGAAAATGGATGGCGCTTAAGCGCGCGACCTATACCCGGCCGTCGGGGCAAGAGCCAGGCCCCGATGAGTAGGAGGGCGCGGCGGTCGCTGCAAAACCCAGGGCGCGAGCCCGGGCGGAGCGGCCGTCGGTGCAGATCTTGGTGGTAGTAGCAAATATTCAAATGAGAACTTTGAAGGCCGAAGAGGGGAAAGGTTCCATGTGAACGGCACTTGCACATGGGTTAGTCGATCCTAAGAGACGGGGGAAGCCCGTCCGACAGCGCGTCCGCGCGCGAGCTTCGAAAGGGAATCGGGTTAAAATTCCCGAACCGGGACGCGGCGGCTGACGGCAACGTTAGGGAGTCCGGAGACGTCGGCGGGGGCCTCGGGAAGAGTTATCTTTTCTGTTTAACAGCCCGCCCACCCTGGAAACGACTCAGTCGGAGGTAGGGTCCAGCGGCTGGAAGAGCACCGCACGTCGCGCGGTGTCCGGTGCGCCCCCGGCGGCCCGTGAAAATCCGGAGGACCGAGTGCCATCCACGCCCGGTCGTACTCATAACCGCATCAGGTCTCCAAGGTGAACAGCCTCTGGTCGATGGAACAATGTAGGCAAGGGAAGTCGGCAAAATGGATCCGTAACCTCGGGAAAAGGATTGGCTCTGAGGGCTGGGCCCGGGGGTCCCAGTCCCGAACCCGTCGGCTGTCGGCGGACTGCTCGAGCTGCTCCCGCGGCAAGAGCGGGTCGCTGCGTGCCGGCCGGGGGACGGATTGGGAACGGCCCCTCCGGGGGCCTTCCCCGGGCGTCGAACAGTCGACTCAGAACTGGTACGGACAAGGGGAATCCGACTGTTTAATTAAAACAAAGCATTGCGATGGTCCCTGCGGATGCTAACGCAATGTGATTTCTGCCCAGTGCTCTGAATGTCAAAGTGAAGAAATTCAACCAAGCGCGGGTAAACGGCGGGAGTAACTATGACTCTCTTAAGGTAGCCAAATGCCTCGTCATCTAATTAGTGACGCGCATGAATGGATTAACGAGATTCCCACTGTCCCTGTCTACTATCCAGCGAAACCACAGCCAAGGGAACGGGCTTGGCAGAATCAGCGGGGAAAGAAGACCCTGTTGAGCTTGACTCTAGTCCGACTTTGTGAAATGACTTGAGAGGTGTAGTATAAGTGGGAGCCGGAAACGGCGATAGTGAAATACCACTACTTTTAACGTTATTTTACTTATTCCGTGAATCGGAGGCGGGGCATTGCCCCTCTTTTTGGACCAAAGGCCGCTTCGCGGTCGATCCGGGCGGAAGACATTGTCAGGTGGGGAGTTTGGCTGGGGCGGCACATCTGTTAAAAGATAACGCAGGTGTCCTAAGATGAGCTCAACGAGAACAGAAATCTCGTGTGGAACAAAAGGGTAAAAGCTCGTTTGATTCTGATTTCCAGTACGAATACGAACCGTGAAAGCGTGGCCTATCGATCCTTTAGACCTTCGGAATTTGAAGCTAGAGGTGTCAGAAAAGTTACCACAGGGATAACTGGCTTGTGGCAGCCAAGCGTTCATAGCGACGTTGCTTTTTGATCCTTCGATGTCGGCTCTTCCTATCATTGTGAAGCAGAATTCACCAAGTGTTGGATTGTTCACCCACCAATAGGGAACGTGAGCTGGGTTTAGACCGTCGTGAGACAGGTTAGTTTTACCCTACTGATGACGGTGTCGCGATGGTAATTCAACCTAGTACGAGAGGAACCGTTGATTCGCACAATTGGTCATCGCGCTTGGTTGAAAAGCCAGTGGCGCGAAGCTACCGTGCGCTGGATTATGACTGAACGCCTCTAAGTCAGAATCCGGGCCAGAAGCGATGCATGCGTCCGCCGCTCGTTTGCCGACCCTCAGTAGGGGCCATCCGGCCCCCAAAGGCACGTGTCGTTGGCTAAGCCCTCGCGGCAGACAAGCCGTGCGGGCAGCCTTGAAGTACAATTCCCACCGGGCGGCGGGCAGAATCCTTTGCAGACGACTTAAATACGCGACGGGGTATTGTAAGTGGCAGAGTGGCCTTGCTGCCACGATCCACTGAGATTCAGCCCTTTGTCGCTCCGATTCGTCCCTCCCTCCCAAGAAACTTTTTCCATCTCAAAAGAATATCGAGCGGAGGCTGGGGTCTCGATTTCCGCTCGTAGAAAGAGGGCCAAGGAGAGAACCCCGGTATTGCATATGGCACGGGGGAAGCAAGAGATTAAGCACACCGCGGGGTGGAAAAAGCATCTGCGCACGGGCAGGTTAGAAAAACATACAAGCGATGCATCCCTTCAATGTCCCTCGGCATTGACCGCTTGTTCATTTTTTTTTCCGGCTTAATGCGTACGTTTCGAACCACTCGTACACCAAGGCTGCCGCGGCCACGCGTGCCTCACCGGTCGATGCGGTCCACTGCCTTCACAGGCGAGGCAGGAGCAGCATCGAATTCCTGCCCCGGCACCACGCACGAGGCCGGCGGTGCCTACCTCTCGTGCGCAAGCGAGACCGATGCCAGCGCGGCTGACTGGCCTGAATAGGAGAGGCCAGTGCTAGTCCGCGCACGCCACATGCCCCTTCAAAGTTTCCCCGCATCGCTCGAGAATTTTACGGCAGGCCCGTTGAACATCTCCATCGGGAGAGAGAATTTGCATCTCCGCCCCAAGCGTCGATGCTAGTCCGCGCACGCCACATGCCCCTTCAAAGTTTCCCCGCACCGCTCGAGAATTTTATGGCAGACCCGTTAAATATCTCCATCGGGAGAGAAAATTTGCATCTCCGCCCCCAACCGTCGAGAAAAGATCAACACTTTTACCATCCCGAGGGTTGTACACACAGCCTCCTGAAACAAAGGGGCGGCAAAAACGCGTTTCGCCTCCAGTCCACGCTCGGTCTTTCGGAACGGGTTCAACGGCAGTTGATTTTATACCGAGTTGCCAAATTCTCACGAGGGCTCTGTATTCTTTTTCGTTTGTCAAGTTCCCGGCAGTCTTAAATACGAGAAAGGCCATCTCGAAGGTCCCGGCATTCTCACGAAGGTTCTAAATATATATTTTCCTTTTTTCAAGAGCTAGGACTTCTTAAAAATGGAAAAAATTATCCCCCGTGTCCGGAAAGTCCCGGTAATGCATGTAGATTTCCCTTGAACCATGCTGGTCAGAATTTCTGCAAAAAAATGAAGGGTCTACGTATCTTCTGTCCATTTTTCCCGGACTCCCTAAAAATGGGAAAAATACCCCCCATGTTCGGAAAGTCCCGATATTACATGTAAATATCCCTTAAATCACACCCGAACTGCGGTCACAATTTCAGCAAAAAAAAAAAAATGAAGTCTTTACATAT

Euphorbia neogossweileri TTTTTTCAAGTTTTCCGGGCCTTCCTC-AAATGGAAAAAAATATCGCCCATGTCCGAAAAGTCCCGGTATTACATGTAAATGTCCCTTCGAAAATGTTCGAACGGTGGTCGAAATTTCAACAAAAAATGAAGGATTTACATATTTGTTTCCATTTATTCCGAAAACCTTAAAAATGGAAAAAAGTAACCCCACTTTCGGAAAGCCCCGGTATTACATGTAAATGTCCCGTAAACAATGCTCGAATCGTGGTAAAAATTTCAGCTAAAAAAGAAGGCTTTACATATTTTTTACCATTTTTCCCGGAATTCCTAAAAATGGAAAAAATACCCCCGTCGGGCGGATTGGCCCTATATTACACGCAAATTTCCCCCAAATCATGCTCGAACTGTGGTAGGAATTTCGGCAAAAAAAGAAGGATCTGCATATTTTTTTCCATTTTTTCCGGAATTCCTAAAAATGGAAAAAATACCTCCCACGTCCGGAAAGTCCCGATATTACGTGTAAATGTCCCTCAAATCATGCTCGAAGCGTGGTAAAAATTTCAGAAAAAAATAAAGGCTTTACATATTTTTTTCCACTTTTCCAGTGCCCAGAATTCTCAAGAATGGAAAAAATAGCCCCGTTGTCCGGGAAGTCCCGGGATTGCATGAAAATTTCCCTTAAATCATGCTCGAACTATGGTAAAAATTTCAGCAAAAAACTCCAAGTATAGAGCAGTTTTTGGGGGGGTGTGGCTCCTGGAACAAATCGACGTCCGTTCCTCCCAGAGCTGGATTTGTCATAAGAAATACTATAGGGGCTGCGCCAGCTCTCAACCTTGGCCCGCACTGGGCTGCGGGCCCGTGTTGGGTGCCTCCCGCATGATCTCCCGCATTCAAGCGCATCACTCGGTCTCCCAGAAAGTCCCGACCCGCTGCCCCACCGAACGGAAGAATATCGTGGAAAAGCTAAGCCCAAAAGCACCACACGCGCGCGCGCG-CGGCGCGGCGCGTTCGCGATTTAGGCGATTTAGGCACTTGGCACTTAGCACGAAGCACGAAGCGCGAGGCGCGAGGCCAATCGGCAGAACATCGCAGAAAATATCCCTCGAAATTTATAAACGAG------------------------------------------AAAACGTGTGCGGGAGCAGGCGGGACGGCCTCAAGTTCAAGCCAGAGTGCAGGGCGGCCGGGCCCGCCTTCGTGTGCGGGAGCAGGCGGGACAGCGTGCAGCGCTGTCCCGCTGTCCTGCCAGTGCCTGGCGAGGCTAGGCAGGACGGCCAGCGTGCTGGCCCGCCCGCATGCGCAGGAGGGACTGCCAGCGGGCGGCCTGGCCTGCCTGGGCAGGGCTGGCAAGCTGCCCTGGCTCGCCAGAGTGCATGGCGAGGGAGGGCAGGCTGGCCAGCACGCTGCCCTGCTTGGCTCGCCAGCCAGCACCGCGCAGGGGGGCCGCCAGCCGGAGGTCAGCACG---------------------CAGCCCTGCAGGCGGTGTTCACCCAGCCCGCGCAAAGGAGGTTAGGTATGCGTGCTGTCTCGCCTGGACGAGGTTCAGTGCCGCTTCGCAGGGCCCGATCCAAATTTCGTTTACAAAACCGTCGCACAGATGATAAGTTTTCCCGCACCAGATTTTTTCTACAGACTTGTCCCATTTATTAGGAATTCTGTACCGAGTTTGACATGTTTTC-TTCTGTTTATTTATTTTTTTATATTTTTACGATTCTCGGACTTCGTAAATACGGAAAAAATACCTCCCGTGGTTAAAAAGTTCCAATATCATATGGAAAAATCCCTTAATTCATGTTCTATCTACAGTCAAAATTTCAGGAAAAAATTCGAAGGTTAGTTCGGTTTTTGAGGGGGTGTGGCTCCTGGAACAAATCGATGTCTGTTCCTCCCAGAGCTGGAAATGCTATAAGGACTACTATAGGGGGGTACACCTGCTCTCAACCCTGGCCACCGCTGGGGCTGCGGGCCCGTGTTGGGTGCTTGCCGCATGCTCACCCCCTATCATGCGGGGAATTTAGCCACTAAGAACGTCCCGACCCGCCGCCTCTCCTCCGGCCGCCGGCCACCGCCCTGCGCGGTGGTCGGAAAATTCAAAATTCTTAGATTGCTGAATTCCGCACCCCGAGAGCCCCTTTTCCCCCTGCCATTGTCGGAACTCGGTAGGATTGGGGGTAGAGTGGGTTTTCGGAGGCAACAACGTGCCCCGCGGCGCTGTCCCGATCGCTAATGTGTGCTTACCTGTAGTTTTGTCTGGCATCGGATAGCTGATTGAGCTGCTCTCAGTGTGTATGGTGCTCGACTCCAAGCTACTTTGGAACCACGCTTAGGGTGAGGGGTAGCCGCTTGCTGTGTACGGTATCGGACGAGGGAAAAAGGAATTTCGAAAAAAGGTGTTTCCGTGCCGTGCGTGATGGTGCTACATCCGTAGTTGCTTGCTGCGTGCGGTGTCGGGCGAGGTAAAAAACGGGATCTGAAAATAAGTCATTTTCACGAAGTGCGCTTCGGCATTGCCCCGATCGATTGTGTGTGTTGCTCGGTAGATTTGTTTGGCATTGGATAGCCGGTCGAGTTGCTCTCGGTGTGAAGATGCTGGACGTTGGTCCACTGGGTAGCTCGCCTTGAGTGAGGGGAAGTTGGTTGATGCGTGCGGTGCTGGATGTCGAAAAAATCGATTTCACTACAGGCAACTTTCTTGCTGTGCGCTCAGGTGCCGTCCCGATCGGTAGTGCATGTTATGCCCGGTGGTTCTGTTTGGCATCTGATAGCTGATTGAGTCGCTCTCAGTGTGTAAGGTGCTGGGTTTTGGACTATTTGGCTTGCCTGCCTATTGTGAGGAGCGGTTGCTTGGTGTGTGCAGTTTCGGACGGCGAAAAATAGAATTTCTATAAAAGATGGTTTTCTCAACACGTGCTGTTCTGTCATGCGGTAAGGAATGTTCTCTCGCACACAGCGGTTCGGGTGATATCTCTACTCGACGTTTCGGCACTGCTTGATTCGTTCTCGAAGACAGCGGTGCAGTTCGGGGGGTGGGGATGTTGCTCAATATACGCGGCGGTGCATGAGTGGTACATAGGCCATTGGGGTCGGCAGGCTCTGTGCTAACGCATCGAACTGTCGTACCTCGAGGCCACTCAGTGGTGTCCCGGAGGCGTATTGCTATGTCGGGCGGGCATGGTTTCTGTGTTGCATACCCGTGCAGTGGAATGGAATTTTGTTGCTAAGAAACATTCGTCTCGAGCCCCTTTGGGGGCGTCGGATGAACCATGCAGCAGCTCTCGTGTGCTGGGTATGCCTTTTTGGCTTCTCCGGCACATGTGAAGGTGCTCGTGCTCTCGGATGTGGAATGCTTTTGCGAGAGGAGGGATTGAGTTTCCTTTGTGTGTTCTCGCTGTCCCTACATAAGAAACACCGTCCTTCCCGCACAGTGGACTTGGTTGCTGCGGTGTACTAAGTCTGCTTGCGGGTTAGGACGGGACGGAGGAATGCTACCTGGTTGATCCTGCCAGTAGTCATATGCTTGTCTCAAAGATTAAGCCATGCATGTGTAAGTATGAACCAATTCAGACTGTGAAACTGCGAATGGCTCATTAAATCAGTTATAGTTTGTTTGATGGTACCTGCTACTCGGATAACCGTAGTAATTCTAGAGCTAATACGTGCAACAAACCCCGACTTCTGGAAGGGATGCATTTATTAGATAAAAGGTCGACGCGGGCTCTGCCCGTTGCTCTGATGATTCATGATAACTCGACGGATCGCACGGCCATCGTGCTGGCGACGCATCATTCAAATTTCTGCCCTATCAACTTTCGATGGTAGGATAGAGGCCTACCATGGTGGTGACGGGTGACGGAGAATTAGGGTTCGATTCCGGAGAGGGAGCCTGAGAAACGGCTACCACATCCAAGGAAGGCAGCAGGCGCGCAAATTACCCAATCCTGACACGGGGAGGTAGTGACAATAAATAACAATACCGGGCTCTTCGAGTCTGGTAATTGGAATGAGTACAATCTAAATCCCTTAACGAGGATCCATTGGAGGGCAAGTCTGGTGCCAGCAGCCGCGGTAATTCCAGCTCCAATAGCGTATATTTAAGTTGTTGCAGTTAAAAAGCTCGTAGTTGGACCTTGGGTTGGGTCGACCGGTCCGCCTTGCGGTGTGCACCTGTCGGCTCGTCCCTTCTGCCGGCGATGCGCTCCTGGCCTTAACTGGCCGGGTCGTGCCTCCGGCGCTGTTACTTTGAAGAAATTAGAGTGCTCAAAGCAAGCCTACGCTCTGTATACATTAGCATGGGATAACATCATAGGATTTCGGTCCTATTCTGTTGGCCTTCGGGATCGGAGTAATGATTAACAGGGACAGTCGGGGGCATTCGTATTTCATAGTCAGAGGTGAAATTCTTGGATTTATGAAAGACGAACAACTGCGAAAGCATTTGCCAAGGATGTTTTCATTAATCAAGAACGAAAGTTGGGGGCTCGAAGACGATCAGATACCGTCCTAGTCTCAACCATAAACGATGCCGACCAGGGATCGGCGGATGTTGCTTTTAGGACTCCGCCGGCACCTTATGAGAAATCAAAGTCTTTGGGTTCCGGGGGGAGTATGGTCGCAAGGCTGAAACTTAAAGGAATTGACGGAAGGGCACCACCAGGAGTGGAGCCTGCGGCTTAATTTGACTCAACACGGGGAAACTTACCAGGTCCAGACATAGTAAGGATTGACAGACTGAGAGCTCTTTCTTGATTCTATGGGTGGTGGTGCATGGCCGTTCTTAGTTGGTGGAGCGATTTGTCTGGTTAATTCCGTTAACGAACGAGACCTCAGCCTGCTAACTAGCTATGCGGAGGTATCCCTCCGCGGCCAGCTTCTTAGAGGGACTATGGCCTTCTAGGCCAAGGAAGTTTGAGGCAATAACAGGTCTGTGATGCCCTTAGATGTTCTGGGCCGCACGCGCGCTACACTGATGTATTCAACGAGTCTATAGCCTTGGCCGACAGGCCCGGGTAATCTTTGAAATTTCATCGTGATGGGGATAGATCATTGCAATTGTTGGTCTTCAACGAGGAATTCCTAGTAAGCGCGAGTCATCAGCTCGCGTTGACTACGTCCCTGCCCTTTGTACACACCGCCCGTCGCTCCTACCGATTGAATGGTCCGGTGAAGTGTTCGGATCGCGGCGACGTGGGCGGTTCGCCGCCGGCGACGTCGCGAGAAGTCCACTGAACCTTATCATTTAGAGGAAGGAGAAGTCGTAACAAGGTTTCCGTAGGTGAACCTGCGGAAGGATCATTGTCGAAACCTGCCAGCAGAATGACCCGCGAACGTGTTTATAAATCGAGGGGCCGCTGCAGGATTCATCCGGCGACGGCACCTCACTGGGGCCCTGGCAGGGGATGCAGTGCGGTGGGATCCACCGTTCCCTGTGATCTCCTGTTTGTGGCCTGTTAACAAAACCCCGGCGCCGTACGCGCCAAGGAATTGCAAAAAAAGATTGTGCAGCCCGATCGCACTGGCAACGGTGTGGCGGGTTTCACTGCGCTTTGAGAACCAAAATGACTCTCGGCAACGGATATCTCGGCTCTCGCATCGATGAAGAACGCAGCGAAATGCGATACTTGGTGTGAATTGCAGGATCCCGCGAACCATCGAGTCTTTGAACGCAAGTTGCGCCCGAAGCCTTTCGGCCGAGGGCACGTCTGCCTGGGTGTCACTCAAACGTTGCTCCAAACCCCTTCTATTGGGAGGGGCATGCGGGGCGGATGCTGGCCTCCCGTGCGCGTGTCGCTCGCGGTTGGCCGAAATTTCTGGTCCTCGGCACGACGCCACGGAATCGGTGGTTGCAAGACCCTCGGAGAAAGCCTTGTGCGCTTGTAAGCCCTTTCGGACCATGGGACCCCAGAGCGTACCTAGCACTGCGACCCCAGGTCAGGCGGGATTACCCGCCGAGTTTAAGCATATCAATAAGCGGAGGAAAAGAAACTTACCAGGATTCCCCTAGTAACGGCGAGCGAACCGGGAAGAGCCCAGCTTGAGAATCGTGCGCCTGCGGCGTTCGAATTGTAGTCTGGAGAAGCGTCCTCAGCGGCGGACCGGGCCCAAGTCCCCTGGAAGGGGGCGCCGGAGAGGGTGAGAGCCCCGTCGTGCCCGGACCCTGTCGCACCACGAGGCGCTGTCTACGAGTCGGGTTGTTTGGGAATGCAGCCCAAATCGGGCGGTAAATTCCGTCCAAGGCTAAATATGGGCGAGAGACCGATAGCGAACAAGTACCGCGAGGGAAAGATGAAAAGGACTTTGAAAAGAGAGTCAAAGAGTGCTTGAAATTGTCGGGAGGGAAGCGGATGGGGGCCGGCGATGTGCCCCGGTCGGATGTGGAACGGTGATAAGCCGGTCCGCCGATCGGCTCGGGGTGCGGACCGATACGGATTGAGGCGGCGGCGTAAGCCCAGGGATTTGAAATGCCTGTGGAGATGCCGTCGCAGCAATCGTGGAAAGCAGCACGCGCCGTCTCGGCGTGCCTCGGCACCTGCGTGCTACTGGTGTCGGCCAGCGGGCTCCCCATCCGGCCCGTCTTGAAACACGGACCAAGGAGTCTGACATGTGTGCGAGTCAACGGGCGAGTAAACCCGTAAGGCGCAAGGAAGCTGACTGGCGGGATCCCCTAGAGGGTTGCACTGCCGACCGACCTTGATCTTCTGAGAAGGGTTCGAGTGAGAGCATGCCTGTCGGGACCCGAAAGATGGTGAACTATGCCTGAGCGGGGCGAAGCCAGAGGAAACTCTGGTGGAGGCCCGCAGCGATACTGACGTGCAAATCGTTCGTCTGACTTGGGTATAGGGGCGAAAGACTAATCGAACCGTCTAGTAGCTGGTTCCCTCCGAAGTTTCCCTCAGGATAGCTGGAGCTCGGAACGAGTTCTATCGGGTAAAGCCAATGATTAGAGGCATCGGGGGCGCAACGCCCTCGACCTATTCTCAAACTTTAAATAGGTAGGACGGCGTGGCTGCTTTGTTGAGCCGCGACACGGAATCGAGAGCTCCAAGTGGGCCATTTTTGGTAAGCAGAACTGGCGATGCGGGATGAACCGGAAGCCGGGTTACGGTGCCCAACTGTGCGCTAACCTAGAACCCACAAAGGGTGTTGGTCGATTAAGACAGCAGGACGGTGGTCATGGAAGTCGAAATCCGCTAAGGAGTGTGTAACAACTCACCTGCCGAATCAACTAGCCCCGAAAATGGATGGCGCTTAAGCGTGCGACCTATACCCGGCCGTCGGGGCAAGAGCCAGGCCGCGATGAGTAGGAGGGCGCGGCGGTCGCTGCAAAACCCAGGGCGCGAGCCCGGGCGGAGCGGCCGTCGGTGCAGATCTTGGTGGTAGTAGCAAATATTCAAATGAGAACTTTGAAGGCCGAAGAGGGGAAAGGTTCCATGTGAACGGCACTTGCACATGGGTTAGTCGATCCTAAGAGACGGGGGAAGCCCGTCCGACAGCGCGTCCGCGCGCGAGCTTCGAAAGGGAATCGGGTTAAAATTCCCGAACCGGGACGCGGCGGCTGACGGCAACGTTAGGGAGTCCGGAGACGTCGGCGGGGGCCTCGGGAAGAGTTATCTTTTCTGTTTAACAGCCCGCCCACCCTGGAAACGACTCAGTCGGAGGTAGGGTCCAGCGGCTGGAAGAGCACCGCACGTCGCGCGGTGTCCGGTGCGCCCCCGGCGGCCCGTGAAAATCCGGAGGACCGAGTGCCATCCACGCCCGGTCGTACTCATAACCGCATCAGGTCTCCAAGGTGAACAGCCTCTGGTCGATGGAACAATGTAGGCAAGGGAAGTCGGCAAAATGGATCCGTAACCTAGGGAAAAGGATTGGCTCTGAGGGCTGGGCCCGGGGGTCCCAGTCCCGAACCCGTCGGCTGTCGGCGGACTGCTCGAGCTGCTCCCGCGGCAAGAGCGGGTCGCTGCGTGCCGGCCGGGGGACGGATTGGGAACGGCCCCTCCGGGGGCCTTCCCCGGGCGTCGAACAGTCGACTCAGAACTGGTACGGACAAGGGGAATCCGACTGTTTAATTAAAACAAAGCATTGCGATGGTCCCTGCGGATGCTAACGCAATGTGATTTCTGCCCAGTGCTCTGAATGTCAAAGTGAAGAAATTCAACCAAGCGCGGGTAAACGGCGGGAGTAACTATGACTCTCTTAAGGTAGCCAAATGCCTCGTCATCTAATTAGTGACGCGCATGAATGGATTAACGAGATTCCCACTGTCCCTGTCTACTATCCAGCGAAACCACAGCCAAGGGAACGGGCTTGGCAGAATCAGCGGGGAAAGAAGACCCTGTTGAGCTTGACTCTAGTCCGACTTTGTGAAATGACTTGAGAGGTGTAGTATAAGTGGGAGTCGGAAACGGCGATAGTGAAATACCACTACTTTTAACGTTATTTTACTTATTCCGTGAATCGGAGGCGGGGCATTGCCCCTCTTTTTGGACCCAAGGCCGCTTCGCGGTCGATCCGGGCGGAAGACATTGTCAGGTGGGGAGTTTGGCTGGGGCGGCACATCTGTTAAAAGATAACGCAGGTGTCCTAAGATGAGCTCAACGAGAACAGAAATCTCGTGTGGAACAAAAGGGTAAAAGCTCGTTTGATTCTGATTTCCAGTACGAATACGAACCGTGAAAGCGTGGCCTATCGATCCTTTAGACCTTCGGAATTTGAAGCTAGAGGTGTCAGAAAAGTTACCACAGGGATAACTGGCTTGTGGCAGCCAAGCGTTCATAGCGACGTTGCTTTTTGATCCTTCGATGTCGGCTCTTCCTATCATTGTGAAGCAGAATTCACCAAGTGTTGGATTGTTCACCCACCAATAGGGAACGTGAGCTGGGTTTAGACCGTCGTGAGACAGGTTAGTTTTACCCTACTGATGACGGTGTCGCGATGGTAATTCAACCTAGTACGAGAGGAACCGTTGATTCGCACAATTGGTCATCGCGCTTGGTTGAAAAGCCAGTGGCGCGAAGCTACCGTGCGCTGGATTATGACTGAACGCCTCTAAGTCAGAATCCGGGCCAGAAGCGATGCATGCGTCCGCCGCTCGTTTGCCGACCCTCAGTAGGGGCCGTCCGGCCCCCAAAGGCACGTGTCGTTGGCGAAGCCCTCGCGGCAGACAAGCCGTGGGGGCAGCCTTGAAGTACAATTCCCACCGGGCGGCGGGCAGAATCCTTTGCAGACGACTTAAATACGCGACGGGGTATTGTAAGTGGCAGAGTGGCCTTGCTGCCACGATCCACTGAGATTCAGCCCTTTGTCGCTCCGATTCGTCCCTCCCTCCCAAGAAGCATTTTCAATCTCAGTAGAGTATCGAGCGGAGGCTGGGGTCTCGATTTCCGCTCGTAGAAAGAAGGCCAAGTAGAGAACCCCAGTATTGCATATGGCACGGGGGAAGCAAGAGATTAAGCACACCGCGGGGTGGAAAAAGCATCTGCGCATGGGCACGGCAGAGAAACATACAGGCGATGCATCCCTTCAA-GTCCCTCGGCGATCACCGCCTGTTCA-TTTTTTTTCCAGCTTAATGCGTACGTTTCGAACCACTCGTACACTAA---------GGCCACGCGTG----------CGACGCGGTCTACTGCCTTCACGGGCGAGACTGGAGCGGCATCGAATTCCTGTCCCGGCTCCACGCACGAGGCCGGCGGTGCCTACCTCTCGTGCGCAAGCGAGACAAATGCCAGCACAGCTGACTGGCCTGCAGAGGAGAGGCCGGTGCTAGTCCGCGCACGCCACATGCCCCTTCAAAGTTTCCCCGCATCGCTCGAGAATTTTATGGCAGGCCCGTTAGACATCTCCATCGGGAGAGAAATTTTGCGTCTCCGCCCCAAGCGTCGATGCTAGTCCGCGCACGCCACATGCCCCTTCAGGGTTTCCCCGCATCGCTCGAGAATTTTATGGCAGGCCCGTTAGACATCTCCATCGGGAGAGAAAATTTGCATCTCCG-CCACAAGCGTCGAGAAAAAATTAACACTTTTACCACCCCGATGGCTGTACACACAGCCTCCTGAAACAAAGGGGCGGC-AAAATGCGTTTCGCCTCCAGTCCACGCTCGGTCTTTCGGAACGGGTTCAACGGCAGTTGATTTTATACCGAGTTGCCAAATTCTCACGAGGGCTCTGTATTCTTTTTCGTTTGTCAAGTTTCCGGCAGTCATAAATACGAGAAAGGCCATCTAGAAGGTCCCGGCATTTTCATGGGGGTTCTAAATATATGTTTTCCTTTTTTCAAGAGCTCGGACTTCCTACAAAAAGGGAAA--------------------------------------AAATTTCCCTTGAACCACGCTGGTCAGAATTTCTGCAAAAAAATGAAGGGTCTACGTATCTTTTTTCCATTTTTCCCGGACTTCTTAAAAATGGGAAAAATATCCCCCATGTTCGGAAATTCCCGGTATTCCATGTAAGTATCTCTTAAATCACACCCGAACTGTGGTCTCAATTTCAGC-----AAAAAAAATGAAGTATGTACATAT

Euphorbia neospinescens TTTTTTCAAGTTTTCCGGGCCTTCCTCAAAATGGAAAAAAATACCGCCCATGTCCGAGAAGTCCCGGTATTACATGTAAATGTCCCTTCGAAAATG-TCGGACGGTGGTCGAAATTTCAACAAAAAAGGAAGGCTTTACATATTTTTTTCCATTTTTTCCGAAACCCTTAAAGATGGAAAAAATAGCCCCCATCTCCGGAAAGCCCCGGTATTACTTGTAAATGTCCCTTATATAATGCTCGAACCGTGGTAAAAATTACAGCTTAAAATGGAGGATTTACATATTTTTTTCCATTTTCCCCAGAATTCCTAAAAATGGAAAAAATGCCCCCGTCGGGCGGATTGGCCCGATATTACGTGTAAATGTCCATCAAATCTTGCTCGAACCGTGGTAAAAATTTCAGCAAAAAATGAAGGCTCTGCGCATTTTTTTCCATTTTTACCGGAATTCTTAAAAATGGGAAAAATATCCCCGACGTCCGGATTGGCCCGATATTACGTGTAAATGTCCATCAAATCATGCTCGAACCGTGGTAAAAATTTCAGCAAAAAACGAAGGCTTTACATATTTTTTTCCATTTTTCCAGTGCCCGGAATTCTAAAAAATGGCAAAAATAGCCCCGTTGTCCGGCAAGTCCCGGGACTGCATGTAAATTTCCCTTAAATCATGCTCGAACTATGGTAAAAATTTCAGCAAAAAAATCCAAGTATAGAGCAGTTTTTGGGGGGGTGTGGCTCCTGGAACAAATCGATGTCTGTTCCTCCCAGAGCTGGATTTGTCATAAGAACTACTATAAGGGCTGCGCCAGCTCTCAACCTTGGCCCGCACTGGGCTGCGGGCCCGTGTTGGGTGCCTCCCGCGTGCTCTCCCGCATTCAAGCGCATCACTCGGTGTCCCAGAAAGTCCCGACCCGCCGCCCCACCGAACGGAAGAATACCGTGGAAAAGCCAAGCCCAAAGGCACCACACGCGCGCGCGCGACGGCGCGGCGCGTTCGCGATTTAGGCGATTTAGGCACTTGGCACTTAGCACGAAGCGCGAAGCGCGAGGCGCGAGGCCAATCGGCAGGACATCGCAGAAAATATCCCGCAAAATTTATAAACGAGTAACGGCACGGCATGAAA-AGAAGCTAGCTAGCAAAATTTTTAAAAAATA--------AACGAAGCCCAACCTGAAGTTCAGGCATGAGTGCAGGGCGGCCTGGCCCGCCTTCGTGTGCGGGAGCAGGCGGGACAGCGGGCAGCGCTGTCCCGCTTTCCTGCCTGTGCCTGGCGAGGCTAGGCAGGACGGCCAGCGTGCTGGCCCGCCCGCCTGCGCAGGAGGGCCTGCCAGTGGTAGGCCTGGCCCGCCTGCGCAGGGCTGGCAACCTGCCCTGGCTCGCCTGAGTGCATGGCGAGGCAGGGCGAGCTGGCCAGCACGCAGCCCTGCCTGGCTCGCCAGCCTGCACCACGCAGGGAGGCCGCCAACCAGAGGCCAGCATAGGCGTGCCAGCACGCCCAGCCGAGCTCTGCGGGCGGTGTTCAGCCAGCACGCACAAAGGAGGCTGGGTCTGCGTGGTGTCCCGCCTGGAAGAGGCTCAGACTGGCTCCGCAGGGCCCGATCCAAATTATTTTTACAAAACCTCCGCACAGATAATAAGTTTTCCCGCACCATATTTTGTATGAAGACGTTTCCCATTTATTAGGAATTATGTACCGAGTTTCGTATGTTTTCGTTGAGTTTATATATTTTTTTATATTTTTTCGATTCTCGGACTTCGAAAATACGGAAAAAATACCTCCCGTGGTTAAAAAGTTCCAATATTATATGGAAAAATCCCTTAATTCATGTTCTATCTACAGTCAAAATTTCAGGAAAAAATTCGAAGGTTTGGTCGGTTTTTAAGGGGGTGTGGCTCCTGGAACAAATCGATGTCTGTTCCTCCCAGAGCTGGAAATGCTATAAGGACTACTATAGGGGGGTACACCTGCTCTCAACCCTGGCCACCACTGGGGCTGCGGGCCCGTGTTGGGTGCTTGCCGCATGCTCACCCCCCATCATGCGGGGAATTCAGCCACTAAGAACGTCCCGACCCGCCGCCCCTCCTCCGGCCGCCGGCCGCCGCCCCGCGCGGTGGCCGGAAAATTCAAAATTCTTAGAACGCTGAATTCCGCACCCCGAGAGCCCCTTTTCCCCCTGCCATTGTCGGAACTCGATCGAATTGGGGGTAAAGTGGGTTTTCGGAGGCAACAACGTGCCCCGCGGCGCTGTCCCGATCGCTAGTGTGTGCCTACACGTAGTTTTGTCTTGCATCGGATAGCTGATTGAGCTGCTCTCAGTGTGTATGTTGCTCGACTTCAAGCTGCCTTGGAAGCACGCCTAGAGTGAGGGGTAGCCGCTTGCTGCGCACGGTATCGGACGAGGGAAAAAGGAATTTCGGAAAAAAACGTTTCCGTGCCGTGCGCGATGGTGCTACATCCGTAGTTGCTTGCTGCGTGCGGTGTGGGGCGAGGTAAAAAACGGAATTTGGAAATAAATTGTTTTCACGAAGTGCGCTTCGGCGTTGTCCCGATCGATGGTGCGTGTTGCTTGGTAGATTTGTTTGGCATTGGATAGCTGATCGAGTAGCTCTCAGTGTGAAGGTGCTGGACGTTGGTCCACTCGGCAGCCCGCCTTGAGTGAGGGGCAGTTGGTTGATGCGTGCGGTGCCGGATGGCGAAAAAATGGATTTCACTAAAAATGACTTTCTTGCTGTGCGCTCAGGCGTCGTCCCGATCGGTAGTGTGCGTTATGCCCGGTGGTTCCGTCTGGCATCTGATAGCTGATCGAGTCGCTCTCAGTGTGTAAGGTGCTGGGTTTTGGACTGTTTGGCTTGCCTGCCTATCGTGAGGAGCGTTTGCTTGGTGTGTACAGTTCGGGACGGTGAAAAATTGAATTTGCATAAAAGATGATTTTCTCAACACGTGCTGCTCTATCATGCGGTAAGGAATGTTCTCTCGCACACAGCGGTTCGGGCGATGTCTCCACTCGACGTTTCGGCACTGCTTGATTCGTTCTCGAAGTCAGCAGTGCAGTTCGGGGGGTGGGGATGTTGCTCAATATACGCGGCGGTGCATGAGTGGTAAATAGGCCATTGGGGTTGGCAGGCTCTGTGCTAGCGCATCGAACTGTCGTACCTCTAGGCCACTCAGTGGTGTCCCGGAGGCGTATTGCTATGTCGGGCGGGGATGGTTTCTGTGTTGCGTACCCGCGTAGTGGAATGGTATTTTGTTGCCAAGAAACATTCGTCTCGTGCCCCTTTAGGGGCGTCGGATGAACCATGCAGCAGCTCTCGTGTGCCGGGCATGCCTTTTTGGCTTCTTCGGCACATGTGAAGGTGCTCGTGCTCTCGGATGCGGAATGCTTTTGCGAGAGGAGGGATTGAGTTTCCTCTATGTGTTCTCGCTGTCCCTACATAAGAACCACCGTCCTTCCCGCACAGTGGCCTTGGTTGCTGCGGTGTACTGTGTCTGCTTGCGGGTTAGGACGGCACGGAGGAATGCTACCTGGTTGATCCTGCCAGTAGTCATATGCTTGTCTCAAAGATTAAGCCATGCATGTGTAAGTATGAACTAATTCAGACTGTGAAACTGCGAATGGCTCATTAAATCAGTTATAGTTTGTTTGATGGTACCTGCTACTCGGATAACCGTAGTAATTCTAGAGCTAATACGTGCAACAAACCCCGACTTCTGGAAGGGATGCATTTATTAGATAAAAGGTCGACGCGGGCTCTGCCCGTTGCTCTGATGATTCATGATAACTCGACGGATCGCACGGCCATCGTGCTGGCGACGCATCATTCAAATTTCTGCCCTATCAACTTTCGATGGTAGGATAGAGGCCTACCATGGTGGTGACGGGTGACGGAGAATTAGGGTTCGATTCCGGAGAGGGAGCCTGAGAAACGGCTACCACATCCAAGGAAGGCAGCAGGCGCGCAAATTACCCAATCCTGACACGGGGAGGTAGTGACAATAAATAACAATACCGGGCTCTTCGAGTCTGGTAATTGGAATGAGTACAATCTAAATCCCTTAACGAGGATCCATTGGAGGGCAAGTCTGGTGCCAGCAGCCGCGGTAATTCCAGCTCCAATAGCGTATATTTAAGTTGTTGCAGTTAAAAAGCTCGTAGTTGGACCTTGGGTTGGGTCGACCGGTCCGCCTTGCGGTGTGCACCTGTCGGCTCGTCCCTTCTGCCGGCGATGCGCTCCTGGCCTTAACTGGCCGGGTCGTGCCTCCGGCGCTGTTACTTTGAAGAAATTAGAGTGCTCAAAGCAAGCCTACGCTCTGTATACATTAGCATGGGATAACATCATAGGATTTCGGTCCTATTCTGTTGGCCTTCGGGATCGGAGTAATGATTAACAGGGACAGTCGGGGGCATTCGTATTTCATAGTCAGAGGTGAAATTCTTGGATTTATGAAAGACGAACAACTGCGAAAGCATTTGCCAAGGATGTTTTCATTAATCAAGAACGAAAGTTGGGGGCTCGAAGACGATCAGATACCGTCCTAGTCTCAACCATAAACGATGCCGACCAGGGATCGGCGGATGTTGCTTTTAGGACTCCGCCGGCACCTTATGAGAAATCAAAGTCTTTGGGTTCCGGGGGGAGTATGGTCGCAAGGCTGAAACTTAAAGGAATTGACGGAAGGGCACCACCAGGAGTGGAGCCTGCGGCTTAATTTGACTCAACACGGGGAAACTTACCAGGTCCAGACATAGTAAGGATTGACAGACTGAGAGCTCTTTCTTGATTCTATGGGTGGTGGTGCATGGCCGTTCTTAGTTGGTGGAGCGATTTGTCTGGTTAATTCCGTTAACGAACGAGACCTCAGCCTGCTAACTAGCTATGCGGAGGTATCCCTCCGCGGCCAGCTTCTTAGAGGGACTATGGCCTTCTAGGCCAAGGAAGTTTGAGGCAATAACAGGTCTGTGATGCCCTTAGATGTTCTGGGCCGCACGCGCGCTACACTGATGTATTCAACGAGTCTATAGCCTTGGCCGACAGGCCCGGGTAATCTTTGAAATTTCATCGTGATGGGGATAGATCATTGCAATTGTTGGTCTTCAACGAGGAATTCCTAGTAAGCGCGAGTCATCAGCTCGCGTTGACTACGTCCCTGCCCTTTGTACACACCGCCCGTCGCTCCTACCGATTGAATGGTCCGGTGAAGTGTTCGGATCGCGGCGACGTGGGCGGTTCGCCGCCGGCGACGTCGCGAGAAGTCCACTGAACCTTATCATTTAGAGGAAGGAGAAGTCGTAACAAGGTTTCCGTAGGTGAACCTGCGGAAGGATCATTGTCGAAACCTGCCAGCAGAATGACCCGCGAACGTGTTTATAAATCGAGGGGCCGCTGCAGGATTCATCCAGCGACGGCACCTCATTAGGGCCCTGGCAGGGGATGCGGTGCGGTGGGATCCACCGTTCCCTGCGGCCTCCTGCTTGCGGCCTATTAACAAAACCCCGGCGCCGTACGCGCCAAGGAATTGCAAAAAAAGATTGTGCAGTCCGATCGCACTGGCAACGGTGTGGCGGGTTTCACTAGGCTTTGAGAACCAAAATGACTCTCGGCAACGGATATCTCGGCTCTCGCATCGATGAAGAACGCAGCGAAATGCGATACTTGGTGTGAATTGCAGGATCCCGCGAACCATCGAGTCTTTGAACGCAAGTTGCGCCCAAAGCCTTTCGGCCGAGGGCACGTCTGCCTGGGTGTCACTCAAACGTCGCTCCAAACCCCTTCCATTTGGAGGGGTATGCAGGGCGGATGCTGGCCTCCCGTGCGCGTATCGCTCGCGGTTGGCCGAAATTCGGAGTCCTCGGCGCGACGCCACGGAATCGGTGGTTGCAAGACCCTCGGAGAAAGCCTTGTGCGCTTGTAAGCCCTTTCGGACCATGAGACCCCAGAGCGTACCTAGCACTGCGACCCCAGGTCAGGCGGGATTACCCGCTGAGTTTAAGCATATCAATAAGCGGAGGAAAAGAAACTTACCAGGATTCCCCTAGTAACGGCGAGCGAACCGGGAAGAGCCCAGCTTGAGAATCGTGCGCCTGCGGCGTTCGAATTGTAGTCTGGAGAAGCGTCCTCAGCGGCGGACCGGGCCCAAGTCCCCTGGAAGGGGGCGCCGGAGAGGGTGAGAGCCCCGTCGTGCCCGGACCCTGTCGCACCACGAGGCGCTGTCTACGAGTCGGGTTGTTTGGGAATGCAGCCCAAATCGGGCGGTAAATTCCGTCCAAGGCTAAATATGGGCGAGAGACCGATAGCGAACAAGTACCGCGAGGGAAAGATGAAAAGGACTTTGAAAAGAGAGTCAAAGAGTGCTTGAAATTGTCGGGAGGGAAGCGGATGGGGGCCGGCGATGTGCCCCGGTCGGATGTGGAACGGTGACAAGCCGGTCCGCCGATCGGCTCGGGGTGCGGACCGATACGGATTGAGGCGGCGGCGTAAGCCCAGGAATTTGAAACGCCTGTGGAGATGCCGTCGCAGCAATCGTGGAAAGCAGCACGCGCCGTCTCGGCGTGCCTCGGCACCCGCGTGCTACTGGTGTCGGCCAGCGGGCTCCCCATTCGGCCCGTCTTGAAACACGGACCAAGGAGTCTGACATGTGTGCGAGTCAACGGGCGAGTAAACCCGTAAGGCGCAAGGAAGCTGACTGGCGGGATCCCCAAGAGGGTTGCACCGCCGACCGACCTTGATCTTCTGAGAAGGGTTCGAGTGAGAGCATGCCTGTCGGGACCCGAAAGATGGTGAACTATGCCTGAGCGGGGCGAAGCCAGAGGAAACTCTGGTGGAGGCCCGCAGCGATACTGACGTGCAAATCGTTCGTCTGACTTGGGTATAGGGGCGAAAGACTAATCGAACCGTCTAGTAGCTGGTTCCCTCCGAAGTTTCCCTCAGGATAGCTGGAGCTCGGAACGAGTTCTATCGGGTAAAGCCAATGATTAGAGGCATCGGGGGCGCAACGCCCTCGACCTATTCTCAAACTTTAAATAGGTAGGACGGCGCGGCTGCTTCGTTGAGCCGCACCACGGAATCGAGAGCTCCAAGTGGGCCATTTTTGGTAAGCAGAACTGGCGATGCGGGATGAACCGGAAGCCGGGTTACGGTGCCCAACTGCGCGCTAACCTAGAACCCACAAAGGGTGTTGGTCGATTAAGACAGCAGGACGGTGGTCATGGAAGTCGAAATCCGCTAAGGAGTGTGTAACAACTCACCTGCCGAATCAACTAGCCCCGAAAATGGATGGCGCTTAAGCGCGCGACCTATACCCGGCCGTCGGGGCAAGAGCCAGGCCCCGATGAGTAGGAGGGCGCGGCGGTCGCTGCAAAACCCAGGGCGCGAGCCCGGGCGGAGCGGCCGTCGGTGCAGATCTTGGTGGTAGTAGCAAATATTCAAATGAGAACTTTGAAGGCCGAAGAGGGGAAAGGTTCCATGTGAACGGCACTTGCACATGGGTTAGTCGATCCTAAGAGACGGGGGAAGCCCGTCCGACAGCGCGTCCGCGCGCGAGCTTCGAAAGGGAATCGGGTTAAAATTCCCGAACCGGGACGCGGCGGCTGACGGCAACGTTAGGGAGTCCGGAGACGTCGGCGGGGGCCTCGGGAAGAGTTATCTTTTCTGTTTAACAGCCCGCCCACCCTGGAAACGACTCAGTCGGAGGTAGGGTCCAGCGGCTGGAAGAGCACCGCACGTCGCGCGGTGTCCGGTGCGCCCCCGGCGGCCCGTGAAAATCCGGAGGACCGAGTGCCATCCACGCCCGGTCGTACTCATAACCGCATCAGGTCTCCAAGGTGAACAGCCTCTGGTCGATGGAACAATGTAGGCAAGGGAAGTCGGCAAAATGGATCCGTAACCTCGGGAAAAGGATTGGCTCTGAGGGCTGGGCCCGGGGGTCCCAGTCCCGAACCCGTCGGCTGTCGGCGGACTGCTCGAGCTGCTCCCGCGGCAAGAGCGGGTCGCTGCGTGCCGGCCGGGGGACGGATTGGGAACGGCCCCTCTGGGGGCCTTCCCCGGGCGTCGAACAGTCGACTCAGAACTGGTACGGACAAGGGGAATCCGACTGTTTAATTAAAACAAAGCATTGCGATGGTCCCTGCGGATGCTAACGCAATGTGATTTCTGCCCAGTGCTCTGAATGTCAAAGTGAAGAAATTCAACCAAGCGCGGGTAAACGGCGGGAGTAACTATGACTCTCTTAAGGTAGCCAAATGCCTCGTCATCTAATTAGTGACGCGCATGAATGGATTAACGAGATTCCCACTGTCCCTGTCTACTATCCAGCGAAACCACAGCCAAGGGAACGGGCTTGGCAGAATCAGCGGGGAAAGAAGACCCTGTTGAGCTTGACTCTAGTCCGACTTTGTGAAATGACTTGAGAGGTGTAGTATAAGTGGGAGTCGGAAACGGCGATAGTGAAATACCACTACTTTTAACGTTATTTTACTTATTCCGTGAATCGGAGGCGGGGCATTGCCCCTCTTTTTGGACCCAAGGCCGCTTCGCGGTCGATCCGGGCGGAAGACATTGTCAGGTGGGGAGTTTGGCTGGGGCGGCACATCTGTTAAAAGATAACGCAGGTGTCCTAAGATGAGCTCAACGAGAACAGAAATCTCGTGTGGAACAAAAGGGTAAAAGCTCGTTTGATTCTGATTTCCAGTACGAATACGAACCGTGAAAGCGTGGCCTATCGATCCTTTAGACCTTCGGAATTTGAAGCTAGAGGTGTCAGAAAAGTTACCACAGGGATAACTGGCTTGTGGCAGCCAAGCGTTCATAGCGACGTTGCTTTTTGATCCTTCGATGTCGGCTCTTCCTATCATTGTGAAGCAGAATTCACCAAGTGTTGGATTGTTCACCCACCAATAGGGAACGTGAGCTGGGTTTAGACCGTCGTGAGACAGGTTAGTTTTACCCTACTGATGACGGTGTCGCGATGGTAATTCAACCTAGTACGAGAGGAACCGTTGATTCGCACAATTGGTCACCGCGCTTGGTTGAAAAGCCAGTGGCGCGAAGCTACCGTGCGCTGGATTATGACTGAACGCCTCTAAGTCAGAATCCGGGCCAGAAGCGATGCATGCGTCCGCCGCTCGTTTGCCGACCCTCAGTAGGGGCCATCCGGCCCCCAAAGGCACGTGTCGTTGGCTAAGCCCTCGCGGCAGAGGAGCCGCGCGGGCAGCCTTGAAGTACAATTCCCACCGGGCGGCGGGCAGAATCCTTTGCAGACGACTTAAATACGCGACGGGGTATTGTAAGTGGCAGAGTGGCCTTGCTGCCACGATCCACTGAGATTCAGCCCTTTGTCGCTCCGATTCGTCCCTCCCTCCCAAGAAACACGTCGCGTCTCAATAGAATATCGAGCGGAGGCTGGGGTCTCGATTTCCGCTCGTACAAAGAAGGCCAAGTAGGGAACCCCGGTATTGCATATGGCACGGGGGAAGCGAGAGATTAAGCACACCGCGGGGTGGAAAAAGCATCTGCGGATGGGCAGGTTAGAAAAACACACAAGCGATGCATCCCTTCAA-GTCCCTCGGCGTTTGCCGCTTGTTCAATTTCTTTTCCAGCTTAATGCGTACGTTTCGAACCACCCGTGCACCAAGGCTGCCGCGGCCACGCGTGCCTCACCGGTCGATGCGGTCTACTGCCTTCACGGGCGAGGCTGGAGCAGCATCGAATTCCTGCCCCGGCTCCACGCACGAGGCCGGCGGTGCCTACCTCTCGTGCGCAAGCGAGACCGATGCCAGCGCGGCTCACTGGCCTGCATAGGAGAGGCCGGTGCTAGTCCGCGCACGCCACATGCCCCTTCAAAGTTTCCCCGCACCGCTCGAGAATTCTATGGCAGGCCCGTTAAACATCTCCATCGGGAGAGAAAAATTGCAATCCCGCCCCAAGCGTCGATGCTAGTCTGCGCACGCCACATGCCCCTTCAAAGTTCCCCCGCACCGCTCGAGAATTTTATGGCAGGCCCGTTAAATATCTCCATCGGGAGAGAGAATTTGCATCTCCG-CCCCAAGCGTCGAGAAAAAATCAACACTTTTACCACCCCGAGGGTTGTACACGCAGCCTCCTGAAACAAAGGGGCAGCAAAAACGCGTTTCGCCTCCAGTCCACGCAGGGTCTTTCGGAACGGGTTCAACGGCAGTTGATTTTATACCGAGTTGCCAAACTCTCACGAGGGCTCTGTATTCTTTTTCGTTTGTCAAGTTCCCGGCAGTCTTAAATACGAGAAAGGCCATCTAGAAGGTCCCGGCATTTTCACGAAGGTTCTAAATATATATTTTCCTTTTTCCAAGAGCTCGGACTTCTTAAAAATGGAAAAAATTATCCCCCGTGTCCGGAAAGTCCCGGCAATGCACGTAGATTTCCCTCAAACCGTACTGGTCAGAATTTCTGC-AAAAAATGAAGGGTCTACGTATCTTTTGTCCATTTTTCCCGGACTTCTTAAAAATGGGAAATATATCCCCCATGTTCGGAAAGCCCCGATATTACATGTAAATATCCCTTGAATCACACCCGAACTGTGGGCACAATTTCAGTAACAAAAAAAAATTGAAGTCTTTACATGT

Euphorbia pereskiifolia TTTTTTCAAGTTTTCCGGGCCTTCCTCAAAATGGAAAAAGATATCGCCCATGTCCGAAAAGTCCCGGTATTACATGTAAATGTCCCTTTGAAAATGTTCGAACGGTGGTCGAAATTTCAACAGAAAGTGAAGGCTTTACATTTTTTTTTCCATTTTTTCCGAAACCCTTAAATATGGAAAAAATAACCCCCATGTCCGGAAAGCCCCGGTATTACATGTAAATGTCCCCTAAATAATGCTCGAACCGTGGTAAAAATTTCAGCTAAAAATGAAGGCTTTACATATTTTTTGCCATTTCCCCCGTAATTCCTAAAAATGGAAAAAATGCCCCCGTCGGGCGGATTGGCCCGATATTACACGTCAATTTCCCCCAAATCATGCTCGAACTGGGGTCGGAATTTCGGCAAAAAAGGAAGGCTCTGCATATTTTTTTCCATTTTTCCCGGAATTCTTAAAAATGGGAAAAATATCGCCCACGTCCGGAAACTCCCGATATCAAGTGTAAATGTCCCTCAAATCATGCTCGAACCGTGGTAAAAATTTCAGCGAAAAATGAAGGCGTTGCATATTTTTTTCCATTTTTCCAGTGCCCGGAATTCACAAAAATGGAAAAAATAGCCCCGTTGTCCGGGAAGTCCCGGGATTGCATGTAAATTTCCCTTAAATCATGCTCGAACTATGGTAAAAATTTCAGAAAAAATCTCCAAGTATAGAGCAGTTTTTGGGGGGGTGTGGCTCCTGGAACAAATCGATGTCCGTTCCTCCCAGAGCTGGATTTGTCATAAGAAATACTATAGGGGCTGCGCCAGCTCTCAACCTTGGCCCGCACTGGGCTGCGGGCCCGTGGTGGGTGCCTCCCGCATGCTCTCCCGCATTCAAGCGCATCACTCGGTCTCCCAGAAAGTCCCGACCCGCCGCCCCACCGAACGGAAGAATATCGTGGAAAAGCTAAGCCCAAAAGCACCACACGCGCGCGCGCGACGGCGCGGCGCGTTCGCGATTTAGGCGATTTAGGCACTTGGCACTTAGCACGTAGCACGAAGCGCGAGGCGCGAGGCCAATCGGCGGAACGTAGCAGAAAATATCCCGCAAAATTTATAAACGAGTAACGACACGGCATGAAAAAGAAGGTAGCTAGCAAAAATTTTAAAAAAAAGAAAGGAACATGAAGGCCAACCCGAAGTTCAGGCAAGAGTGCAGGGCGGCCTGGCCCGCCTTCGTGTGCGGGAGCAGGCGGGACAGCGTGCAGCGCTGTCCCGCATTCCTGCCTGTGCCTGGCGAGGCTAGGCAGGACGGCCGGCGTGCTGGCCCGCCCGCGTGTGCAGGAGGGACTGCCAGTGGGAGGGCTGGCCCGCCTGGGCAGGGCTGGCAAGCTGCCCTGGCTCGCCTGAGTGCATGGCGGGGGAGGGCAAGCTGGCCAGCACGCAGCCCTGCCTGGCTCGCCAGCCAGCACCACGCAGGCAGGCCGCCAACCAGAGGTCAGCATAGGCATGCCAGCACGCCCAGCCGAGCTCTGCCGGCGGTGCTCACCCAGTCCGCGAAAAGGAGGCTAGGTCAGCGTGCTGTCTCGCCTGGCCGAGGCTCAGGATGGCTTCGCTGGGCCCGATCCAAATTTCGTTTGCAAAACCTTCGCACAGATAATAAGTTTTCCCGCACCATATTTTTTATACTGACTTTTACCATTTATTAGGAATTTTGTACCGAGTTCGTGATGCATTTGTTGAGTTTATATATTTTTTTGTATTTTTACGATTCTCGGACTTCGTAAATATGGAAAAAATACCTCCCGTGGGTAAAAGGTTACAATATTATATGGAAAAATCCCTTAATTCATGTTCTATCTACAGTCAAAATTTCAGGAAAAAACTCGAAGGTTTGACCGGTTTTTAAGGGGGTGTGGCTCCTGGAACAAATCGATGTCTGTTCCTCCCAGAGCTGGAAATGCTATAAGGACTACTATAGGGGGGTACACCTGCTCTCAACCCAGGCCACCACTGGGGCTGCGGGCCCGTGTAGGGTGCTTGCCGCATGCTCACCCCCTATCATGCGGGGAATTCAGCCACTAAGAACGTCCCGACCCGCCGCCCCTCCTCCGGCCGCCGGCCGCCGCCCCGCGCGGTGGCCGGAAAATCCAAAATTCTTAGAACGCTGAATTCCGCACCCCGAGAGCCCCTTTTCCCCCTGCCATTGTCGGAACTCGTTCGAATTGGGGGTAAAGTGGGTTTTCGGAGGCAACAACGTGCCCCGCGGCGCTGTCCCGATCGCTAGTGTGTGCCTACTCGTAGTTTTGTCTTGCATCGGATAGCTGATTGAGCTGCTCTCAGTGTGTATGGTGCTCGACTTCAAGCTGCTTTGGAAGCACGCCTAGAGTGAGGGGTAGCCGCTTGCTGCGCACGGTATCGGACGAGGGAAAAAGGAATTTCGGAAAAAAACGTTTCCGTGCCGTGCGCGATGTTGCTACATCCGTAGCTGCTTGCTGCGTGCGGTGTGGGGCGATGTAAAAAATGGAATTTGAAAATAAAATGTTTTCACGGAGTGCGCTTCGGCGTTGCCCCGATCGATAGTGCGTGTTGCTCGGTGGATTTGTTTGGCATTGGATAGCCGATCGAGTTGCTCTCGGTGTGAAGGTGCTGGACGTTGGTCCACTCGGCAGCCCGCCTTGAGTGAGGGGCAGTTGGTTGATGCGTGCGGTGCCAAGTGGCGAAAAAATGGATTTCACTAAAAATGTCTTTCTTGCTGTGCGCTCAGGCGCCGTCCCGATCGGTAGTGTGCGTTATGTCCGGTGGTTCTGTTTGGCATCTGATAGCTGATCGAGTCGCTCTCAGTGTGTAAGGTGCTGGGTTTTGGACTATTTGGCTTGCCTGCCTATCGTGAGGAGCGGTTGCTTGGTGCGTACAGTTCCGGACGGCGAAAAATTGAATTCCAATAAAATATTATTCTCTCAACACGTGCTACTTTATCATGCGGTAAGGAATGTTCTCTCGCACACAGCGGTTCGGGCGATGTCTCTACTCGACGTTTCGGCACTGCTTGATTCGTTCTCGAAGACAGCAGTGCAGTTCGGGGGGTGGGGATGTTGCTCAATATACGCGGCGGTGCATGAGTGGTAAATAGGCCATTGGGGTTGGCAGGCTCTGTGCTAGCGCATCGAACTGTCGTACCTTGAGGCCACTCAGTGGTGTCCCGGAGGCGTATTGCTATGTCGGGCGGGGATGGTTTCTGTGTTGCATACCCGCGCAGTGGAATGGAATTTTGTTGCCAAGAAACATTCGTCCCGTGCCCTTTTAGGGGCGTCGGATGAACCATGCAGCAGCTCTCGTGTGCCGGGCATGCCTTTTTGGCTTCTCTGGCACATGTGAAGGTGCTCGTGCTCTCGGATGCGGAATGCTTTTGCGAGAGGAGGGATTGAGTTTCCTTTATGTGTTCTCGCTGTCCCTACATAAGAACCACCGTCCTTTCCGCACAGTGGCCTTGGTTGCTGCGGTGTACTATGTCTGCTTGCGGGTTAGGACGGCATGGAGGAATGCTACCTGGTTGATCCTGCCAGTAGTCATATGCTTGTCTCAAAGATTAAGCCATGCATGTGTAAGTATGAACTAATTCAGACTGTGAAACTGCGAATGGCTCATTAAATCAGTTATAGTTTGTTTGATGGTACCTGCTACTCGGATAACCGTAGTAATTCTAGAGCTAATACGTGCAACAAACCCCGACTTCTGGAAGGGATGCATTTATTAGATAAAAGGTCGACGCGGGCTCTGCCCGTTGCTCTGATGATTCATGATAACTCGACGGATCGCACGGCCATCGTGCTGGCGACGCATCATTCAAATTTCTGCCCTATCAACTTTCGATGGTAGGATAGAGGCCTACCATGGTGGTGACGGGTGACGGAGAATTAGGGTTCGATTCCGGAGAGGGAGCCTGAGAAACGGCTACCACATCCAAGGAAGGCAGCAGGCGCGCAAATTACCCAATCCTGACACGGGGAGGTAGTGACAATAAATAACAATACCGGGCTCTTCGAGTCTGGTAATTGGAATGAGTACAATCTAAATCCCTTAACGAGGATCCATTGGAGGGCAAGTCTGGTGCCAGCAGCCGCGGTAATTCCAGCTCCAATAGCGTATATTTAAGTTGTTGCAGTTAAAAAGCTCGTAGTTGGACCTTGGGTTGGGTCGACCGGTCCGCCTTACGGTGTGCACCTGTCGGCTCGTCCCTTCTGCCGGCGATGCGCTCCTGGCCTTAACTGGCCGGGTCGTGCCTCCGGCGCTGTTACTTTGAAGAAATTAGAGTGCTCAAAGCAAGCCTACGCTCTGTATACATTAGCATGGGATAACATCATAGGATTTCGGTCCTATTCTGTTGGCCTTCGGGATCGGAGTAATGATTAACAGGGACAGTCGGGGGCATTCGTATTTCATAGTCAGAGGTGAAATTCTTGGATTTATGAAAGACGAACAACTGCGAAAGCATTTGCCAAGGATGTTTTCATTAATCAAGAACGAAAGTTGGGGGCTCGAAGACGATCAGATACCGTCCTAGTCTCAACCATAAACGATGCCGACCAGGGATCGGCGGATGTTGCTTTTAGGACTCCGCCGGCACCTTATGAGAAATCAAAGTCTTTGGGTTCCGGGGGGAGTATGGTCGCAAGGCTGAAACTTAAAGGAATTGACGGAAGGGCACCACCAGGAGTGGAGCCTGCGGCTTAATTTGACTCAACACGGGGAAACTTACCAGGTCCAGACATAGTAAGGATTGACAGACTGAGAGCTCTTTCTTGATTCTATGGGTGGTGGTGCATGGCCGTTCTTAGTTGGTGGAGCGATTTGTCTGGTTAATTCCGTTAACGAACGAGACCTCAGCCTGCTAACTAGCTATGCGGAGGTATCCCTCCGCGGCCAGCTTCTTAGAGGGACTATGGCCTTCTAGGCCAAGGAAGTTTGAGGCAATAACAGGTCTGTGATGCCCTTAGATGTTCTGGGCCGCACGCGCGCTACACTGATGTATTCAACGAGTCTATAGCCTTGGCCGACAGGCCCGGGTAATCTTTGAAATTTCATCGTGATGGGGATAGATCATTGCAATTGTTGGTCTTCAACGAGGAATTCCTAGTAAGCGCGAGTCATCAGCTCGCGTTGACTACGTCCCTGCCCTTTGTACACACCGCCCGTCGCTCCTACCGATTGAATGGTCCGGTGAAGTGTTCGGATCGCGGCGACGTGGGCGGTTCGCCGCCGGCGACGTCGCGAGAAGTCCACTGAACCTTATCATTTAGAGGAAGGAGAAGTCGTAACAAGGTTTCCGTAGGTGAACCTGCGGAAGGATCATTGTCGAAACCTGCCAGCAGAATGACCCGCGAACGTGTTTATAAATCGAGGGGCCGCTGCAGGATTCATCCAGCGATGGCACCTCACTAGGGCCCTGGCAGGGGATGCGGTGCGGTGGGATCCACCGTTCCCTGCGATCTCCTGTTTGCGGCCTATTAACAAAACCCCGGCGCCGTACGCGCCAAGGAATTGTAAAAAAAGATTGTGCAGCCCGATCGCACTGGCAACGGTGTGGCGGGTTTCACTGCGCTTTGAGAACCAAAATGACTCTCGGCAACGGATATCTCGGCTCTCGCATCGATGAAGAACGCAGCGAAATGCGATACTTGGTGTGAATTGCAGGATCCCGCGAACCATCGAGTCTTTGAACGCAAGTTGCGCCCGAAGCCTTTCGGCCGAGGGCACGTCTGCCTGGGTGTCACTCAAACGTCGCTCCAAACCCCTTCCATCGGGAGGGGTATGCGGGGCGGATGCTGGCCTCCCGTGTGCGTATCGCTCGCGGTTGGCCGAAATTCCTAGTCCTCGGCACGACGCCACGGAATCGGTGGTTGCAAGACCCTCGGAGAAAGCCTTGTGCGCTTGTAAGCCCTTTCGGACCATGAGACCCCAGAGCGTACCTAGCACTGCGACCCCAGGTCAGGCGGGATTACCCGCTGAGTTTAAGCATATCAATAAGCGGAGGAAAAGAAACTTACCAGGATTCCCCTAGTAACGGCGAGCGAACCGGGAAGAGCCCAGCTTGAGAATCGTGCGCCTGCGGCGTTCGAATTGTAGTCTGGAGAAGCGTCCTCAGCGGCGGACCGGGCCCAAGTCCCCTGGAAGGGGGCGCCGGAGAGGGTGAGAGCCCCGTCGTGCCCGGACCCTGTCGCACCACGAGGCGCTGTCTACGAGTCGGGTTGTTTGGGAATGCAGCCCAAATCGGGCGGTAAATTCCGTCCAAGGCTAAATATGGGCGAGAGACCGATAGCGAACAAGTACCGCGAGGGAAAGATGAAAAGGACTTTGAAAAGAGAGTCAAAGAGTGCTTGAAATTGTCGGGAGGGAAGCGGATGGGGGCCGGCGATGCGCCCCGGTCGGATGTGGAACGGTGACAAGCCGGTCCGCCGATCGGCTCGGGGCGCGGACCGATACGGATTGAGGCGGCGGCGTAAGCCCAGGAATTTGAAACGCCTGTGGAGATGCCGTCGCAGCAATCGTGGAAAGCAGCACGCGCCGTCTCGGCGTGCCTCGGCACCTGCGTGCTACTGGTGTCGGCCAGCGGGCTCCCCATTCGGCCCGTCTTGAAACACGGACCAAGGAGTCTGACATGTGTGCGAGTCAACGGGCGAGTAAACCCGTAAGGCGCAAGGAAGCTGACTGGCGGGATCCCCTAGAGGGTTGCACCGCCGACCGACCTTGATCTTCTGAGAAGGGTTCGAGTGAGAGCATGCCTGTCGGGACCCGAAAGATGGTGAACTATGCCTGAGCGGGGCGAAGCCAGAGGAAACTCTGGTGGAGGCCCGCAGCGATACTGACGTGCAAATCGTTCGTCTGACTTGGGTATAGGGGCGAAAGACTAATCGAACCGTCTAGTAGCTGGTTCCCTCCGAAGTTTCCCTCAGGATAGCTGGAGCTCGGAACGAGTTCTATCGGGTAAAGCCAATGATTAGAGGCATCGGGGGCGCAACGCCCTCGACCTATTCTCAAACTTTAAATAGGTAGGACGGCGCGGCTGCTTCGTTGAGCCGCGCCACGGAATCGAGAGCTCCAAGTGGGCCATTTTTGGTAAGCAGAACTGGCGATGCGGGATGAACCGGAAGCCGGGTTACGGTGCCCAACTGCGCGCTAACCTAGAACCCACAAAGGGTGTTGGTCGATTAAGACAGCAGGACGGTGGTCATGGAAGTCGAAATCCGCTAAGGAGTGTGTAACAACTCACCTGCCGAATCAACTAGCCCCGAAAATGGATGGCGCTTAAGCGCGCGACCTATACCCGGCCGTCGGGGCAAGAGCCAGGCCCCGATGAGTAGGAGGGCGCGGCGGTCGCTGCAAAACCCAGGGCGCGAGCCCGGGCGGAGCGGCCGTCGGTGCAGATCTTGGTGGTAGTAGCAAATATTCAAATGAGAACTTTGAAGGCCGAAGAGGGGAAAGGTTCCATGTGAACGGCACTTGCACATGGGTTAGTCGATCCTAAGAGACGGGGGAAGCCCGTCCGACAGCGCGTCCGCGCGCGAGCTTCGAAAGGGAATCGGGTTAAAATTCCCGAACCGGGACGCGGCGGCTGACGGCAACGTTAGGGAGTCCGGAGACGTCGGCGGGGGCCTCGGGAAGAGTTATCTTTTCTGTTTAACAGCCCGCCCACCCTGGAAACGACTCAGTCGGAGGTAGGGTCCAGCGGCTGGAAGAGCACCGCACGTCGCGCGGTGTCCGGTGCGCCCCCGGCGGCCCGTGAAAATCCGGAGGACCGAGTGCCATCCACGCCCGGTCGTACTCATAACCGCATCAGGTCTCCAAGGTGAACAGCCTCTGGTCGATGGAACAATGTAGGCAAGGGAAGTCGGCAAAATGGATCCGTAACCTCGGGAAAAGGATTGGCTCTGAGGGCTGGGCCCGGGGGTCCCAGTCCCGAACCCGTCGGCTGTCGGCGGACTGCTCGAGCTGCTCCCGCGGCAAGAGCGGGTCGCTGCGTGCCGGCCGGGGGACGGATTGGGAACGGCCCCTCCGGGGGCCTTCCCCGGGCGTCGAACAGTCGACTCAGAACTGGTACGGACAAGGGGAATCCGACTGTTTAATTAAAACAAAGCATTGCGATGGTCCCTGCGGATGCTAACGCAATGTGATTTCTGCCCAGTGCTCTGAATGTCAAAGTGAAGAAATTCAACCAAGCGCGGGTAAACGGCGGGAGTAACTATGACTCTCTTAAGGTAGCCAAATGCCTCGTCATCTAATTAGTGACGCGCATGAATGGATTAACGAGATTCCCACTGTCCCTGTCTACTATCCAGCGAAACCACAGCCAAGGGAACGGGCTTGGCAGAATCAGCGGGGAAAGAAGACCCTGTTGAGCTTGACTCTAGTCCGACTTTGTGAAATGACTTGAGAGGTGTAGTATAAGTGGGAGCCGGAAACGGCGATAGTGAAATACCACTACTTTTAACGTTATTTTACTTATTCCGTGAATCGGAGGCGGGGCATTGCCCCTCTTTTTGGACCAAAGGCCGCTTCGCGGTCGATCCGGGCGGAAGACATTGTCAGGTGGGGAGTTTGGCTGGGGCGGCACATCTGTTAAAAGATAACGCAGGTGTCCTAAGATGAGCTCAACGAGAACAGAAATCTCGTGTGGAACAAAAGGGTAAAAGCTCGTTTGATTCTGATTTCCAGTACGAATACGAACCGTGAAAGCGTGGCCTATCGATCCTTTAGACCTTCGGAATTTGAAGCTAGAGGTGTCAGAAAAGTTACCACAGGGATAACTGGCTTGTGGCAGCCAAGCGTTCATAGCGACGTTGCTTTTTGATCCTTCGATGTCGGCTCTTCCTATCATTGTGAAGCAGAATTCACCAAGTGTTGGATTGTTCACCCACCAATAGGGAACGTGAGCTGGGTTTAGACCGTCGTGAGACAGGTTAGTTTTACCCTACTGATGACGGTGTCGCGATGGTAATTCAACCTAGTACGAGAGGAACCGTTGATTCGCACAATTGGTCATCGCGCTTGGTTGAAAAGCCAGTGGCGCGAAGCTACCGTGCGCTGGATTATGACTGAACGCCTCTAAGTCAGAATCCGGGCCAGAAGCGATGCATGCGTCCGCCGCTCGTTTGCCGACCCTCAGTAGGGGCCATCCGGCCCCCAAAGGCACGTGTCGTTGGCTAAGCCCTCGCGGCAGACAAGCCGTGCGGGCAGCCTTGAAGTACAATTCCCACCGGGCGGCGGGCAGAATCCTTTGCAGACGACTTAAATACGCGACGGGGTATTGTAAGTGGCAGAGTGGCCTTGCTGCCACGATCCACTGAGATTCAGCCCTTTGTCGCTCCGATTCGTCCCTCCCTCCCAAGAAACTTTTTCCATCTCAAAAGAATATCGAGCGGAGGCTGGGGTCTCGATTTCCGCTCGTAGAAAGAGGGCCAAGGAGAGAACCCCGGTATTGCATATGGCACGGGGGAAGCAAGAGATTAAGCACACCGCGGGGTGGAAAAAGCATCTGCGCACGGGCAGGTTAGAAAAACATACAAGCGATGCATCCCTTCAATGTCCCTCGGCATTGACCGCTTGTTCATTTTTTTTTCCGGCTTAATGCGTACGTTTCGAACCACTCGTACACCAAGGCTGCCGCGGCCACGCGTGCCTCACCGGTCGATGCGGTCCACTGCCTTCACAGGCGAGGCAGGAGCAGCATCGAATTCCTGCCCCGGCACCACGCACGAGGCCGGCGGTGCCTACCTCTCGTGCGCAAGCGAGACCGATGCCAGCGCGGCTGACTGGCCTGAATAGGAGAGGCCAGTGCTAGTCCGCGCACGCCACATGCCCCTTCAAAGTTTCCCCGCATCGCTCGAGAATTTTACGGCAGGCCCGTTGAACATCTCCATCGGGAGAGAGAATTTGCATCTCCGCCCCAAGCGTCGATGCTAGTCCGCGCACGCCACATGCCCCTTCAAAGTTTCCCCGCACCGCTCGAGAATTTTATGGCAGACCCGTTAAATATCTCCATCGGGAGAGAAAATTTGCATCTCCGCCCCCAACCGTCGAGAAAAGATCAACACTTTTACCATCCCGAGGGTTGTACACACAGCCTCCTGAAACAAAGGGGCGGCAAAAACGCGTTTCGCCTCCAGTCCACGCTCGGTCTTTCGGAACGGGTTCAACGGCAGTTGATTTTATACCGAGTTGCCAAATTCTCACGAGGGCTCTGTATTCTTTTTCGTTTGTCAAGTTCCCGGCAGTCTTAAATACGAGAAAGGCCATCTCGAAGGTCCCGGCATTCTCACGAAGGTTCTAAATATATATTTTCCTTTTTTCAAGAGCTAGGACTTCTTAAAAATGGAAAAAATTATCCCCCGTGTCCGGAAAGTCCCGGTAATGCATGTAGATTTCCCTTGAACCATGCTGGTCAGAATTTCTGCAAAAAAATGAAGGGTCTACGTATCTTCTGTCCATTTTTCCCGGACTCCCTAAAAATGGGAAAAATACCCCCCATGTTCGGAAAGTCCCGATATTACATGTAAATATCCCTTAAATCACACCCGAACTGCGGTCACAATTTCAGCAAAAAAAAAAAAATGAAGTCTTTACATAT

Euphorbia pseudomollis TTTTTTCAAGTTTTCCGGGCCTTCCTCAAAATGGAAAAAGATATCGCCCATGTCCGAAAAGTCCCGGTATTACATGTAAATGTCCCTTTGAAAATGTTCGAACGGTGGTCGAAATTTCAACAAAAAGTGAAGGCTTTACATTTTTTTTTCCATTTTTTCCGAAACCCTTAAATATGGAAAAAATAACCCCCATGTCCGGAAAGCCCCGGTATTACATGTAAATGTCCCCTAAATAATGCTCGAACCGTGGTAAAAATTTCAGCTAAAAATGAAGGCTTTACATATTTTTTGCCATTTCCCCCGTAATTCCTAAAAATGGAAAAAATGCCCCCGTCGGGCGGATTGGCCCGATATTACACGTCAATTTCCCCCAAATCATGCTCGAACTGGGGTCGGAATTTCGGCAAAAAAGGAAGGCTCTGCATATTTTTTTCCATTTTTCCCGGAATTCTTAAAAATGGGAAAAATATCCCCCACGTCCGGAAACTCCCGATATCAAGTGTAAATGTCCCTCAAAGCATGCTCGAACCGTGGTAAAAATTTCAGCGAAAAATGAAGGCGTTGCATATTTTTTTCCATTTTTCCAGTGCCCGGAATTCACAAAAATGGAAAAAATAGCCCCGTTGTCCGGGAAGTCCCGGGATTGCATGTAAATTTCCCTTAAATCATGCTCGAACTATGGTAAAAATTTCAGAAAAAATCTCCAAGTATAGAGCAGTTTTTGGGGGGGTGTGGCTCCTGGAACAAATCGATGTCCGTTCCTCCCAGAGCTGGATTTGTCATAAGAAATACTATAGGGGCTGCGCCAGCTCTCAACCTTGGCCCGCACTGGGCTGCGGGCCCGTGGTGGGTGCCTCCCGCATGCTCTCCCGCATTCAAGCGCATCACTCGGTCTCCCAGAAAGTCCCGACCCGCCGCCCCACCGAACGGAAGAATATCGTGGAAAAGCTAAGCCCAAAAGCACCACACGCGCGCGCGCGACGGCGCGGCGCGTTCGCGATTTAGGCGATTTAGGCACTTGGCACTTAGCACGTAGCACGAAGCGCGAGGCGCGAGGCCAATCGGCGGAACATAGCAGGAAATATCCCGCAAAATTTATAAACGAGTAACGACACGGCATGAAAAAGAAGGTAGCTAGCAAAAATTTTAAAAAAAAGAAAGGAACATGAAGGCCAACCCGAAGTTCAGGCAAGAGTGCAGGGCGGCCTGGCCCGCCTTCGTGTGCGGGAGCAGGCGGGACAGCGTGCAGCGCTGTCCCGCATTCCTGCCTGTGCCTGGCGAGGCTAGGCAGGACGGCCGGCGTGCTGGCCCGCCCGCGTGTGCAGGAGGGCCTGCCAGTGGGAGGGCTGGCCCGCCTGGGCAGGGCTGGCAAGCTGCCCTGGCTCGCCTGAGTGCATGGCGGGGGAGGGCAAGCTGGCCAGCACGCAGCCCTGCCTGGCTCGCCAGCCAGCACCACGCAGGCAGGCCGCCAACCAGAGGTCAGCATAGGCATGCCAGCACGCCCAGCCGAGCTCTGCCGGCGGTGCCCACCCAGTCCGCGAAAAGGAGGCTAGGCCAGCCTGCTGTCTCGCCTGGCCGAGGCTCAGGATGGCTTCGCTGGGCCCGATCCAAATTTCGTTTGCAAAACCTTCGCACAGATAATAAGTTTTCCCGCACCATATTTTTTATACTGACTTTTACCATTTATTAGGAATTTTGTACCGAGTTCGTGATGCATTTGTTGAGTTTATATATTTTTTTGTATTTTTACGATTCTCGGACTTCGTAAATATGGAAAAAATACCTCCCGTGGGTAAAAGGTTACAATATTATATGGAAAAATCCCTTAATTCATGTTCTATCTACAGTCAAAATTTCAGGAAAAAACTCGAAGGTTTGACCGGTTTTTAAGGGGGTGTGGCTCCTGGAACAAATCGATGTCTGTTCCTCCCAGAGCTGGAAATGCTATAAGGACTACTATAGGGGGGTACACCTGCTCTCAACCCAGGCCACCACTGGGGCTGCGGGCCCGTGTTGGGTGCTTGCCGCATGCTCACCCCCTATCATGCGGGGAATTCAGCCACTAAGAACGTCCCGACCCGCCGCCCCTCCTCCGGCCGCCGGCCGCCGCCACGCGCGGTGGCCGGAAAATCCAAAATTCTTAGAACGCTGAATTCCGCACCCCGAGAGCCCCTTTTCCCCCTGCCATTGTCGGAACTCGTTCGAATTGGGGGTAAAGTGGGTTTTCGGAGGCAACAACGTGCCCCGCGGCGCTGTCCCGATCGCTAGTGTGTGCCTACTCGTAGTTTTGTCTTGCATCGGATAGCTGATTGAGCTGCTCTCAGTGTGTATGGTGCTCGACTTCAAGCTGCTTTGGAAGCACGCCTAGAGTGAGGGGTAGCCGCTTGCTGCGCACGGTATCGGACGAGGGAAAAAGGAATTTCGGAAAAAAACGTTTCCGTGCCGTGCGCGATGTTGCTACATCCGTAGTTGCTTGCTGCGTGCGGTGTGGGGCGATGCAAAAAATGGAATTTGAAAATAAATTGTTTTCACGGAGTGCGCTTCGGCGTTGCCCCGATCGATAGTGCGTGTTGCTCGGTGGATTTGTTTGGCATTGGATAGCTGATCGAGTTGCTCTCGGTGTGAAGGTGCTGGACGTTGGTCCACTCGGCAGCCCGCCTTGAGTGAGGGGCAGTTGGTTGATGCGTGCGGTGCCAAGTGGCGAAAAAATGGATTTCACTAAAAATGTCTTTCTTGCTGTGCGCTCAGGCGCCGTCCCGATCGGTAGTGTGCGTTATGTCCGGTGGTTCTGTTTGGCATCTGATAGCTGATCGAGTCGCTCTCAGTGTGTAAGGTGCTGGGTTTTGGACTATTTGGCTTGCCTGCCTATCGTGAGGAGCGGTTGCTTGGTGCGTACAGTTCCGGACGGCGAAAAATTGAATTCCAATAAAATATTATTCTCTCAACACGTGCTACTTTATCATGCGGTAAGGAATGTTCTCTCGCACACAGCGGTTCGGGCGATGTCTCTACTCGACGTTTCGGCACTGCTTGATTCGTTCTCGAAGACAGCAGTGCAGTTCGGGGGGTGGGGATGTTGCTCAATATACGCGGCGGTGCATGAGTGGTAAATAGGCCATTGGGGTTGGCAGGCTCTGTGCTAGCGCATCGAACTGTCGTACCTTGAGGCCACTCAGTGGTGTCCCGGAGGCGTATTGCTATGTCGGGCGGGGATGGTTTCTGTGTTGCATACCCGCGCAGTGGAATGGAATTTTGTTGCCAAGAAACATTCGTCCCGTGCCCTTTTAGGGGCGTCGGATGAACCATGCAGCAGCTCTCGTGTGCCGGGCATGCCTTTTTGGCTTCTCTGGCACATGTGAAGGTGCTCGTGCTCTCGGATGCGGAATGCTTTTGCGAGAGGAGGGATTGAGTTTCCTTTGTGTGTTCTCGCTGTCCCTACATAAGAACCACCGTCCTTTCCGCACAGTGGCCTTGGTTGCTGCGGTGTACTATGTCTGCTTGCGGGTTAGGACGGCATGGAGGAATGCTACCTGGTTGATCCTGCCAGTAGTCATATGCTTGTCTCAAAGATTAAGCCATGCATGTGTAAGTATGAACTAATTCAGACTGTGAAACTGCGAATGGCTCATTAAATCAGTTATAGTTTGTTTGATGGTACCTGCTACTCGGATAACCGTAGTAATTCTAGAGCTAATACGTGCAACAAACCCCGACTTCTGGAAGGGATGCATTTATTAGATAAAAGGTCGACGCGGGCTCTGCCCGTTGCTCTGATGATTCATGATAACTCGACGGATCGCACGGCCATCGTGCTGGCGACGCATCATTCAAATTTCTGCCCTATCAACTTTCGATGGTAGGATAGAGGCCTACCATGGTGGTGACGGGTGACGGAGAATTAGGGTTCGATTCCGGAGAGGGAGCCTGAGAAACGGCTACCACATCCAAGGAAGGCAGCAGGCGCGCAAATTACCCAATCCTGACACGGGGAGGTAGTGACAATAAATAACAATACCGGGCTCTTCGAGTCTGGTAATTGGAATGAGTACAATCTAAATCCCTTAACGAGGATCCATTGGAGGGCAAGTCTGGTGCCAGCAGCCGCGGTAATTCCAGCTCCAATAGCGTATATTTAAGTTGTTGCAGTTAAAAAGCTCGTAGTTGGACCTTGGGTTGGGTCGACCGGTCCGCCTTACGGTGTGCACCTGTCGGCTCGTCCCTTCTGCCGGCGATGCGCTCCTGGCCTTAACTGGCCGGGTCGTGCCTCCGGCGCTGTTACTTTGAAGAAATTAGAGTGCTCAAAGCAAGCCTACGCTCTGTATACATTAGCATGGGATAACATCATAGGATTTCGGTCCTATTCTGTTGGCCTTCGGGATCGGAGTAATGATTAACAGGGACAGTCGGGGGCATTCGTATTTCATAGTCAGAGGTGAAATTCTTGGATTTATGAAAGACGAACAACTGCGAAAGCATTTGCCAAGGATGTTTTCATTAATCAAGAACGAAAGTTGGGGGCTCGAAGACGATCAGATACCGTCCTAGTCTCAACCATAAACGATGCCGACCAGGGATCGGCGGATGTTGCTTTTAGGACTCCGCCGGCACCTTATGAGAAATCAAAGTCTTTGGGTTCCGGGGGGAGTATGGTCGCAAGGCTGAAACTTAAAGGAATTGACGGAAGGGCACCACCAGGAGTGGAGCCTGCGGCTTAATTTGACTCAACACGGGGAAACTTACCAGGTCCAGACATAGTAAGGATTGACAGACTGAGAGCTCTTTCTTGATTCTATGGGTGGTGGTGCATGGCCGTTCTTAGTTGGTGGAGCGATTTGTCTGGTTAATTCCGTTAACGAACGAGACCTCAGCCTGCTAACTAGCTATGCGGAGGTATCCCTCCGCGGCCAGCTTCTTAGAGGGACTATGGCCTTCTAGGCCAAGGAAGTTTGAGGCAATAACAGGTCTGTGATGCCCTTAGATGTTCTGGGCCGCACGCGCGCTACACTGATGTATTCAACGAGTCTATAGCCTTGGCCGACAGGCCCGGGTAATCTTTGAAATTTCATCGTGATGGGGATAGATCATTGCAATTGTTGGTCTTCAACGAGGAATTCCTAGTAAGCGCGAGTCATCAGCTCGCGTTGACTACGTCCCTGCCCTTTGTACACACCGCCCGTCGCTCCTACCGATTGAATGGTCCGGTGAAGTGTTCGGATCGCGGCGACGTGGGCGGTTCGCCGCCGGCGACGTCGCGAGAAGTCCACTGAACCTTATCATTTAGAGGAAGGAGAAGTCGTAACAAGGTTTCCGTAGGTGAACCTGCGGAAGGATCATTGTCGAAACCTGCCAGCAGAATGACCCGCGAACGTGTTTATAAATCGAGGGGCCGCTGCAGGATTCATCCAGCGATGGCACCTCACTAGGGCCCTGGCAGGGGATGCGGTGCGGTGGGATCCACCGTTCCCTGCGATCTCCTGTTTGCGGCCTATTAACAAAACCCCGGCGCCGTACGCGCCAAGGAATTGTAAAAAAAGATTGTGCAGCCCGATCGCACTGGCAACGGTGTGGCGGGTTTCACTGCGCTTTGAGAACCAAAATGACTCTCGGCAACGGATATCTCGGCTCTCGCATCGATGAAGAACGCAGCGAAATGCGATACTTGGTGTGAATTGCAGGATCCCGCGAACCATCGAGTCTTTGAACGCAAGTTGCGCCCGAAGCCTTTCGGCCGAGGGCACGTCTGCCTGGGTGTCACTCAAACGTCGCTCCAAACCCCTTCCATCGGGAGGGGTATGCGGGGCGGATGCTGGCCTCCCGTGTGCGTATCGCTCGCGGTTGGCCGAAATTCCTAGTCCTCGGCACGACGCCACGGAATCGGTGGTTGCAAGACCCTCGGAGAAAGCCTTGTGCGCTTGTAAGCCCTTTCGGACCATGAGACCCCAGAGCGTACCTAGCACTGCGACCCCAGGTCAGGCGGGATTACCCGCTGAGTTTAAGCATATCAATAAGCGGAGGAAAAGAAACTTACCAGGATTCCCCTAGTAACGGCGAGCGAACCGGGAAGAGCCCAGCTTGAGAATCGTGCGCCTGCGGCGTTCGAATTGTAGTCTGGAGAAGCGTCCTCAGCGGCGGACCGGGCCCAAGTCCCCTGGAAGGGGGCGCCGGAGAGGGTGAGAGCCCCGTCGTGCCCGGACCCTGTCGCACCACGAGGCGCTGTCTACGAGTCGGGTTGTTTGGGAATGCAGCCCAAATCGGGCGGTAAATTCCGTCCAAGGCTAAATATGGGCGAGAGACCGATAGCGAACAAGTACCGCGAGGGAAAGATGAAAAGGACTTTGAAAAGAGAGTCAAAGAGTGCTTGAAATTGTCGGGAGGGAAGCGGATGGGGGCCGGCGATGCGCCCCGGTCGGATGTGGAACGGTGACAAGCCGGTCCGCCGATCGGCTCGGGGCGCGGACCGATACGGATTGAGGCGGCGGCGTAAGCCCAGGAATTTGAAACGCCTGTGGAGATGCCGTCGCAGCAATCGTGGAAAGCAGCACGCGCCGTCTCGGCGTGCCTCGGCACCTGCGTGCTACTGGTGTCGGCCAGCGGGCTCCCCATTCGGCCCGTCTTGAAACACGGACCAAGGAGTCTGACATGTGTGCGAGTCAACGGGCGAGTAAACCCGTAAGGCGCAAGGAAGCTGACTGGCGGGATCCCCTAGAGGGTTGCACCGCCGACCGACCTTGATCTTCTGAGAAGGGTTCGAGTGAGAGCATGCCTGTCGGGACCCGAAAGATGGTGAACTATGCCTGAGCGGGGCGAAGCCAGAGGAAACTCTGGTGGAGGCCCGCAGCGATACTGACGTGCAAATCGTTCGTCTGACTTGGGTATAGGGGCGAAAGACTAATCGAACCGTCTAGTAGCTGGTTCCCTCCGAAGTTTCCCTCAGGATAGCTGGAGCTCGGAACGAGTTCTATCGGGTAAAGCCAATGATTAGAGGCATCGGGGGCGCAACGCCCTCGACCTATTCTCAAACTTTAAATAGGTAGGACGGCGCGGCTGCTTCGTTGAGCCGCGCCACGGAATCGAGAGCTCCAAGTGGGCCATTTTTGGTAAGCAGAACTGGCGATGCGGGATGAACCGGAAGCCGGGTTACGGTGCCCAACTGCGCGCTAACCTAGAACCCACAAAGGGTGTTGGTCGATTAAGACAGCAGGACGGTGGTCATGGAAGTCGAAATCCGCTAAGGAGTGTGTAACAACTCACCTGCCGAATCAACTAGCCCCGAAAATGGATGGCGCTTAAGCGCGCGACCTATACCCGGCCGTCGGGGCAAGAGCCAGGCCCCGATGAGTAGGAGGGCGCGGCGGTCGCTGCAAAACCCAGGGCGCGAGCCCGGGCGGAGCGGCCGTCGGTGCAGATCTTGGTGGTAGTAGCAAATATTCAAATGAGAACTTTGAAGGCCGAAGAGGGGAAAGGTTCCATGTGAACGGCACTTGCACATGGGTTAGTCGATCCTAAGAGACGGGGGAAGCCCGTCCGACAGCGCGTCCGCGCGCGAGCTTCGAAAGGGAATCGGGTTAAAATTCCCGAACCGGGACGCGGCGGCTGACGGCAACGTTAGGGAGTCCGGAGACGTCGGCGGGGGCCTCGGGAAGAGTTATCTTTTCTGTTTAACAGCCCGCCCACCCTGGAAACGACTCAGTCGGAGGTAGGGTCCAGCGGCTGGAAGAGCACCGCACGTCGCGCGGTGTCCGGTGCGCCCCCGGCGGCCCGTGAAAATCCGGAGGACCGAGTGCCATCCACGCCCGGTCGTACTCATAACCGCATCAGGTCTCCAAGGTGAACAGCCTCTGGTCGATGGAACAATGTAGGCAAGGGAAGTCGGCAAAATGGATCCGTAACCTCGGGAAAAGGATTGGCTCTGAGGGCTGGGCCCGGGGGTCCCAGTCCCGAACCCGTCGGCTGTCGGCGGACTGCTCGAGCTGCTCCCGCGGCAAGAGCGGGTCGCTGCGTGCCGGCCGGGGGACGGATTGGGAACGGCCCCTCTGGGGGCCTTCCCCGGGCGTCGAACAGTCGACTCAGAACTGGTACGGACAAGGGGAATCCGACTGTTTAATTAAAACAAAGCATTGCGATGGTCCCTGCGGATGCTAACGCAATGTGATTTCTGCCCAGTGCTCTGAATGTCAAAGTGAAGAAATTCAACCAAGCGCGGGTAAACGGCGGGAGTAACTATGACTCTCTTAAGGTAGCCAAATGCCTCGTCATCTAATTAGTGACGCGCATGAATGGATTAACGAGATTCCCACTGTCCCTGTCTACTATCCAGCGAAACCACAGCCAAGGGAACGGGCTTGGCAGAATCAGCGGGGAAAGAAGACCCTGTTGAGCTTGACTCTAGTCCGACTTTGTGAAATGACTTGAGAGGTGTAGTATAAGTGGGAGCCGGAAACGGCGATAGTGAAATACCACTACTTTTAACGTTATTTTACTTATTCCGTGAATCGGAGGCGGGGCATTGCCCCTCTTTTTGGACCAAAGGCCGCTTCGCGGTCGATCCGGGCGGAAGACATTGTCAGGTGGGGAGTTTGGCTGGGGCGGCACATCTGTTAAAAGATAACGCAGGTGTCCTAAGATGAGCTCAACGAGAACAGAAATCTCGTGTGGAACAAAAGGGTAAAAGCTCGTTTGATTCTGATTTCCAGTACGAATACGAACCGTGAAAGCGTGGCCTATCGATCCTTTAGACCTTCGGAATTTGAAGCTAGAGGTGTCAGAAAAGTTACCACAGGGATAACTGGCTTGTGGCAGCCAAGCGTTCATAGCGACGTTGCTTTTTGATCCTTCGATGTCGGCTCTTCCTATCATTGTGAAGCAGAATTCACCAAGTGTTGGATTGTTCACCCACCAATAGGGAACGTGAGCTGGGTTTAGACCGTCGTGAGACAGGTTAGTTTTACCCTACTGATGACGGTGTCGCGATGGTAATTCAACCTAGTACGAGAGGAACCGTTGATTCGCACAATTGGTCATCGCGCTTGGTTGAAAAGCCAGTGGCGCGAAGCTACCGTGCGCTGGATTATGACTGAACGCCTCTAAGTCAGAATCCGGGCCAGAAGCGATGCATGCGTCCGCCGCTCGTTTGCCGACCCTCAGTAGGGGCCATCCGGCCCCCAAAGGCACGTGTCGTTGGCTAAGCCCTCGCGGCAGACAAGCCGTGCGGGCAGCCTTGAAGTACAATTCCCACCGGGCGGCGGGCAGAATCCTTTGCAGACGACTTAAATACGCGACGGGGTATTGTAAGTGGCAGAGTGGCCTTGCTGCCACGATCCACTGAGATTCAGCCCTTTGTCGCTCCGATTCGTCCCTCCCTCCCAAGAAACTTTTTCCATCTCAATAGAATATCGAGCGGAGGCTGGGGTCTCGATTTCCGCTCGTAGAAAGAGGGCCAAGGAGAGAACCCCGGTATTGCATATGGCACGGGGGAAGCAAGAGATTAAGCACACCGCGGGGTGGAAAAAGCATCTGCGCACGGGCAGGTTAGAAAAACATACAAGCGATGCATCCCTTCAATGTCCCTCGGCATTGACCGCTTGTTCATTTTTTTTTCCGGCTTAATGCGTACGTTTCGAACCACTCGTACACCAAGGCTGCCGCGGCCACGCGTGCCTCACCGGTCGATGCGGTCTACTGCCTTCACAGGCGAGGCAGGAGCAGCATCGAATTCCTGCCCCGGCACCACGCACGAGGCCGGCGGTGCCTACCTCTCGTGCGCAAGCGAGACCGATGCCAGCGCGGCTGACTGGCCTGAATAGGAGAGGCCAGTGCTAGTCCGCGCACGCCACATGCCCCTTCAAAGTTTCCCCGCATCGCTCGAGAATTTTACGGCAGGCCCGTTGAACATCTCCATCGGGAGAGAGAATTTGCATCTCCGCCCCAAGCGTCGATGCTAGTCCGCGCACGCCACATGCCCCTACAAAGTTTCCCCGCACCGCTCGAGAATTTTATGGCAGACCCGTTAAATATCTCCATCGGGAGAGAAAATTTGCATCTCCGCCCCCAACCGTCGAGAAAAGATCAACACTTTTACCACCCCGAGGGTTGTACACACAGCCTCCTGAAACAAAGGGGCGGCAAAAACGCGTTTCGCCTCCAGTCCACGCTCGGTCTTTCGGAACGGGTTCAACGGCAGTTGATTTTATACCGAGTTGCCAAATTCTCACGAGGGCTCTGTATTCTTTTTCGTTTGTCAAGTTCCCGGCAGTCTTAAATACGAGAAAGGCCATCTCGAAGGTCCCGGCATTCTCACGAAGGTTCTAAATATATATTTTCCTTTTTTCAAGAGCTAGGACTTCTTAAAAATGGAAAAAATTATCCCCCGTGTCCGGAAAGTCCCGGTAATGCATGTAGATTTCCCTTGAACCATGCTGGTCAGAATTTCTGCAAAAAAATGAAGGGTCTACGTATCTTCTGTCCATTTTTCCCGGACTCCCTAAAAATGGGAAAAATACACCCCATGTTCGGAAAGTCCCGATATTACATGTAAATATCCCTTAAATCACACCCGAACCGTGGTCACAATTTCAGCAAAAAAAAAAAAATGAAGTCTTTACATAT

Euphorbia resinifera GCGATGCAAGGCATGCATGCCTCCCACGAAGCGGACATGCGTGCAGCTCACATGCGGGCAGTCCCTCCCCTGCACACACATGCTTTCTCACGCATGTTCACGTGAAAGACGCAAGGTCCACAAGGTGTCAAGAGTCGACTTGCTACTTGCCCCGCATGCCAACCCACCCGAGGCT-----AAGGCATACATGCTTCGGGAACGTTCACAATTTACGTAACCAAATCTTTTTAATATCATCATAATTTCACGATAGTTTTCAACTTAATTTAACAATTGTAAACCAAGTTTGTCATTCT-------ATACTTCTTCGCTGAAAAAAT-------------------------TTTAAAATAAAAATTTGAATTAAAATAGACCAAAAC---AACTG-AATTTCGACACAAAAAAAAAATTGCACATATTTGAATTAAAATAGACCAAAACAACTGAATTTCGACACAAAACCTCGAACATATAAAAAATTGC-ACATCACATACAAGAGTGCCACGAGGCAAGCCTGAGGCGTGGTACAGACCTCGATAGAACATAGAG-------------TTTTCTGCCTCGCTAGTGCAAGCTAAGCT-AAGGCTGGCAAAACAAGCCTACTTGCCTCGCATGCCTCAGTGCTGCGTGTGCATTTTCACAACTTCACATATGAAAAATGGAAAAAA----AAAAACAAAAACCACAGATGAAAAAGTTATT-----GCTTCAATTACAAAAAAAAACCAATAATTATTTCTTCTAGAAAAAAATTCGCTGATGAAAATTTTAT------TGCATCAATTAAAAAACGCAACTTGCCCATGCATGCGGGCAGGCGTGCAGTGCTGCCTGCCCATGCATCCGGACTAGCGTGCAGTGCTGATTGCCTCAGGTAATCTCGTATCACTGTGCCAATGGATATTAAAATGCCGTAGATGCGTTTTTTGTTCGAGTACTACATGCGCATGCGGGCAGGCGTGCAGTGTTCACAACTTCACGGATGAAAAATTTATTGCTTTAATTGGGAAAAAAAAACAAAAAATGCGAGCAGTGCTTGCGGGGGCATGCGTGAAGTGCCCTCCCATGCCTGTGCACGCG-----GTGTGGCAGGCAGCGGTGCCTGCCTCGCATGCATGTG----------------CATGCGGGCGGGCTGTGCATGCGGGCTGGCGTGCAGCGCTGCCTGCCCCGCACCCGCATGCCTGTGCATGCGGGCTGGCGTGCGGCGCTGCCTGCCCCGCATGCCTGTGCAGGCCTGGCCTGTGCATGCGGGCTGGCGTGCGGCGCTGCCTGCCTATGCGGGCAGGGCTGGGGGCAGCCGTCCAGCGCTGCCTGCGTAGGGGTGGCATGCAGCGCTGGGCTGCCCGGCTGTGCAGCCCTGCAGCGCTGGCCTGCCGGCCTGCAGCGCTGCCTGCCTGGCCGTGCAGCGCTGCCTGCGTAGGCTGCCAACCCGAGGTTACGCGAGGCATGC--------------CAGTGCTGCATGGGCACGCATGCCAGCCAGCCTACAAGAGGTTAGGCTAGCCTGCCAGCCTGCCTACCTGAGGCTAAGGCAGGCACCGGCGGGCCCGTTGAAAATTCTTGTAACAAAAGCCTCGCACAAATCTAAAATTTTTGTGCATTATATTTTCACAGTAGCTTTCCTACATTATTTTATAATTGTATACCGAATTTGGCGATGTTTGATAAAGGTTTTCTATTTTTTTCTATTTTTCCCGTTGTAGACATATTTAAATATGAAAAAAATAGTTCTAATGTCTCAAAAATCTCAAAATTTTTCCTGGATATACCTTGTCCCATGGTCTAACTACGGTCAAAATTTTAGAAGAAAATTCAAAGTTTTAGGCAGTTTTCGAAGGGGTGTGGCTCCTGAGACATGTTGATGGTTCTTCCTCCCACCCTTGAAGTCGGTGTAAGCACTACTATAGGGGGGTACACCTACTCTCAGCCCTGGCCAGCGCTGGGG-TGGGGGCTGGTGCAGGGTGCTGGGTGTATTCATGACTCCCCTAACGCGGCGAATTCGAT-----------------------------------------------------------------------------------------------------------------------------------------------------------------ATTTTTTGAAATTCTATCATGCCCCGCAGCATTTATGCGGACAACG-------------------------------------------------------------------TCTCCGCCTTTGGCCGGAGCAGCACCGCGCTAGGGGCA--------------------------------------AAAATGGAATTTTTTGATTTGAATTTTGCACAACGTATGCGATGAT----------------------------------------------------------------------------------------GGCATTGCCCCGATCAGTAGTGTTGGTTGCCCGGTGGTTTTGTGCGGCATCGGATAGCCGCT--------TTGCGGTGTGAAGGTGTTG---CTCGGACTGTTTGGCAGCCCGCCTATCGTGAGGGGCA--TTGCTTATGCGTGCGGTCCCGGTTGGCGGAAAAAT---------------------------CATGCGACGAGGCATTGCCCCGATCAGTAGTGTTGGT--TGCCCGGTGGTTTTGTGCGGCATCGGATAGCCGCT--------TTGCGGTGTGTAAGGTGTTG---CTCGGACTGTTTGGC-AGCCCGCCTGTCGTGAGGGGCA-TTGCTT-ATGCGTGCGGTCCCGGTTGGCGGTAAA-----------------ATTATTTTCAAGGCTCATTCGGCGGTGGT-----------AATTTTCTCTCGCACCCAGCGGTTAGGGCAATTTTTATACCCGGCGTTTCTGCATTGCTAGTTTCTTTTATGAAAACAGCAGCGCAGTTCGGAGGGTGTAGGTGTTGCTCAATATACGTGGCGGTGCATGAGTGGTAATTTAGCTTTCGGGGTTGGCAGGCTCTGTGCTCGCGCATCGAACTGTT-TACCTCAAGGCAATTCAGTGGTTTCACGAAGGCATGTTGCTCTCTCGTGCGTGGATGGTTCCTGTGTTGTATACCCACATAGCGGAATGTCATTGTGTTGCCTAAAGCTATTCACCTAGTGCCTTGTTAAAGGTATTGGATGAACCTTGCAGCGGCTCTTGTGTGCCCAGCATGCCAATTTGGTTGCTCCGGCACATTGGAAGGCGTTCGTGGTCTCGGATGCGGAATG-GTTTACGAGTAGAGGGATTAAGCTCCCTTTA--TGGTCTCGTTGTCCCTACATAAGAACAACCGTCTCTCTTGTTCAATGGCCTTGGTTGCTGCGGTGCTCTTTGGCTGCTTGCTTGAAGGGACGGTAGGAAGGAATGCTACCTGGTTGATCCTGCCAGTAGTCATATGCTTGTCTCAAAGATTAAGCCATGCATGTGTAAGTATGAACTAATTTATACTGTGAAACTGCGAATGGCTCATTAAATCAGTTATAGTTTGTTTGATGGTACCTGCTACTCGGATAACCGTAGTAATTCTAGAGCTAATACGTGCAACAAACCCCGACTTCTGGAAGGGATGCATTTATTAGATAAAAGGTCAACGCGGGCTCTGCCCGTTGCTCTGATGATTCATGATAACTCGACGGATCGCACGGCCATCGTGCTGGCGACGCATCATTCAAATTTCTGCCCTATCAACTTTCGATGGTAGGATAGAGGCCTACCATGGTGGTGACGGGTGACGGAGAATTAGGGTTCGATTCCGGAGAGGGAGCCTGAGAAACGGCTACCACATCCAAGGAAGGCAGCAGGCGCGCAAATTACCCAATCCTGACACGGGGAGGTAGTGACAATAAATAACAATACCGGGCTCTTCGAGTCTGGTAATTGGAATGAGTACAATCTAAATCCCTTAACGAGGATCCATTGGAGGGCAAGTCTGGTGCCAGCAGCCGCGGTAATTCCAGCTCCAATAGCGTATATTTAAGTTGTTGCAGTTAAAAAGCTCGTAGTTGGACCTTGGGTTGGGTCGACCGGTCCGCCTTGCGGTGTGCACCTGTCGGCTTGTCCCTTTTGCCGGCGATGCGCTCCTGGCCTTAACTGGCCGGGTCGTGCCTCCGGCGCTGTTACTTTGAAGAAATTAGAGTGCTCAAAGCAAGCCTACGCTCTGTATACATTAGCATGGGATAACATCATAGGATTTCGGTCCTATTCTGTTGGCCTTCGGGATCGGAGTAATGATTAACAGGGACAGTCGGGGGCATTCGTATTTCATAGTCAGAGGTGAAATTCTTGGATTTATGAAAGACGAACAACTGCGAAAGCATTTGCCAAGGATGTTTTCATTAATCAAGAACGAAAGTTGGGGGCTCGAAGACGATCAGATACCGTCCTAGTCTCAACCATAAACGATGCCGACCAGGGATCGGCGGATGTTGCTTTTAGGACTCCGCCGGCACCTTATGAGAAATCAAAGTCTTTGGGTTCCGGGGGGAGTATGGTCGCAAGGCTGAAACTTAAAGGAATTGACGGAAGGGCACCACCAGGAGTGGAGCCTGCGGCTTAATTTGACTCAACACGGGGAAACTTACCAGGTCCAGACATAGTAAGGATTGACAGACTGAGAGCTCTTTCTTGATTCTATGGGTGGTGGTGCATGGCCGTTCTTAGTTGGTGGAGCGATTTGTCTGGTTAATTCCGTTAACGAACGAGACCTCAGCCTGCTAACTAGCTATGCGGAGGTATCCCTCCGCGGCCAGCTTCTTAGAGGGACTATGGCCTTCTAGGCCAAGGAAGTTTGAGGCAATAACAGGTCTGTGATGCCCTTAGATGTTCTGGGCCGCACGCGCGCTACACTGATGTATTCAACGAGTCTATAGCCTTGGCCGACAGGCCCGGGTAATCTTTGAAATTTCATCGTGATGGGGATAGATCATTGCAATTGTTGGTCTTCAACGAGGAATTCCTAGTAAGCGCGAGTCATCAGCTCGCGTTGACTACGTCCCTGCCCTTTGTACACACCGCCCGTCGCTCCTACCGATTGAATGGTCCGGTGAAGTGTTCGGATCGCGGCGACGTGGGCGGTTCGCTGCCGGCGACGTCGCGAGAAGTCCACTGAACCTTATCATTTAGAGGAAGGAGAAGTCGTAACAAGGTTTCCGTAGGTGAACCTGCGGAAGGATCATTGTCGAATCCTGCGAACAGAATGACCCGTGAACATGTTTATAAATCGATGGGCTGCTGCAGGATTTATCCGGCATCAGCACCTCATTAGGACGCAAGCAGGGGATGCGAGTGCTTGCATCTGCCCTGCCCTCGTGATGTCCTGTTTGCGGCCTACTAACCAAACCCCGACGCCATACGCGTCAAGGAACTACAAAAAATATTTGCATGCCCCTAGCACACCGGAAACGGTGTGACAGGAAGCGTTGCACTGTGATAACAAAAACGACTCTCGGCAACGGATATCTCGGCTCTCGCATCGATGAAGAACGCAGCGAAATGCGATACTTGGTGTGAATTGCAGGATCCCGCGAACCATCGAGTCTTTGAACGCAAGTTGCGCCCTAAGCCTTCCGGCCGAGGGCACGTCTGCCTGGGTGTCATTCGACTGTCGCTTCAGCCCCTTCTAATTGGAAGGGGCATGCGGGGCGGATGTTGGCCTCCCGTGTGCTCTTTGCTTCCGGTTGGCCCAAATTTCTGGTCACTGGCACGATGCCACAGAATCGGTGGTTGTAAAGCACTCGCAAAAATCTGTGTGCACTCGAAAGCCCATTCAGACCATGAGACCCCAAAAAGTACCTAACACAGCGACCCCAGGTCAGGCGGGATTACCCGCTGAGTTTAAGCATATCAATAAGCGGAGGAAAAGAAACTTACCAGGATTCCCCTAGTAACGGCGAGCGAACCGGGAAGAGCCCAGCTTGAGAATCGAGCGCCTTCGGTGTTCGAATTGTAGTCTGGAGAAGCGTCCTCAGCGGCGGACCGGGCCCAAGTCCCCTGGAAGGGGGCGCCGGAGAGGGTGAGAGCCCCGTCGTGCCCGGACCCTGTCGCACCACGAGGCGCTGTCTGCGAGTCGGGTTGTTTGGGAATGCAGCCCAAATCGGGCGGTAAATTCCGTCCAAGGCTAAATACGGGCGAGAGACCGATAGCGAACAAGTACCGCGAGGGAAAGATGAAAAGGACTTTGAAAAGAGAGTCAAAGAGTGCTTGAAATTGTCGGGAGGGAAGCGGATGGGGGCCGGCGATGCGCCCCGGTCGGATGTGGAACGGTCATAAGCCGGTCCGCCGATCGGCTCGGGGTGCGGACCGATGCGGATTGAGGCAGCGGCCAAAGCCCAGGCCTTTGAAACGCCTGTGGAGATGCTGTTGCAGCGATCGTGGAAAGCAGCGCGCGCCATCAAGGCGTGCCTCGGCACCTGCGCGCTCCTGGCATCGGCCAGCGGGTACCCCATTCGGCCCGTCTTGAAACACGGACCAAGGAGTCTGACATGTGTGCGAGTCAACGGGCGAGTAAACCCGTAAGGCGCAAGGAAGCTGACTGGCGGGATCCCCTCGAGGGTTGCACCGCCGACCGACCTTGATCTTCTGAGAAGGGTTCGAGTGAGAGCATGCCTGTCGGGACCCGAAAGATGGTGAACTATGCCTGAGCGGGGCGAAGCCAGAGGAAACTCTGGTGGAGGCCCGCAGCGATACTGACGTGCAAATCGTTCGTCTGACTTGGGTATAGGGGCGAAAGACTAATCGAACCGTCTAGTAGCTGGTTCCCTCCGAAGTTTCCCTCAGGATAGCTGGAGCTCGGGACGAGTTCTATCGGGTAAAGCCAATGATTAGAGGCATCGGGGGCGCAACGCCTTCGACCTATTCTCAAACTTTAAATAGGTAGGACGGCATGGCTGCTTCGTTGAGCCATGCCATGGAATCGAGAGCTCCAAGTGGGCCATTTTTGGTAAGCAGAACTGGCGATGCGGGATGAACCGGAAGCCGGGTTACGGTGCCTAACTGTGCGCTAACCTAGAACCCACAAAGGGTGTTGGTCGATTAAGACAGCAGGACGGTGGTCATGGAAGTCGAAATCCGCTAAGGAGTGTGTAACAACTCACCTGCCGAATCAACTAGCCCCGAAAATGGATGGCGCTTAAGCGCGCGACCTATACCCGGCCATCAGGGCAAGAGCCAAGCCCTGATGAGTAGGAGGGCGCGGCGGTCGCTGCAAAACCCAGGGCGTGAGCCCGGGCGGAGCGGCCGTCGGTGCAGATCTTGGTGGTAGTAGCAAATATTCAAATGAGAACTTTGAAGGCCGAAGAGGGGAAAGGTTCCATGTGAACGGCACTTGCACATGGGTTAGTCGATCCTAAGAGACGGGGGAAGCCCGTCCGATAGCGTGCCCGCACGCGAGCTTCGAAAGGGAATCGGGTTAAAATTCCCGAACCGGGACGTGGCGGCTGACGGCAACGTTAGGGAGTCCGGAGACGTTGGCGGGGGCCTCGGGAAGAGTTATCTTTTCTGTTTAACAGCCCGCCCACCCTGGAAACGACTCAGTCGGAGGTAGGGTCCAGCGGCTGGAAGAGCACCGCACGTCGCGCGGTGTCCGGTGCGCCCCCGGCGACCCTTGAAAATCCGGAGGACCGAGTGCCATCCACGCCCGGTCGTACTCATAACCGCATCAGGTCTCCAAGGTGAACAGCCTCTGGTCGATGGAACAATGTAGGCAAGGGAAGTCGGCAAAATGGATCCGTAACCTCGGGAAAAGGATTGGCTCTGAGGGCTGGGCCCGGGGGTCCCAGTCCCGAACCCGTCGGCTGTCGGCGGACTGCTCGAGCTGCTCCCGCGGCGAGAGCGGGTCGCCGCGTGCCGGCCGGGGGACGGACTGGGAACGGCCCTTCCGGGGGCCTTCCCCGGGCGTCGAACAGTCGACTCAGAACTGGTACGGACAAGGGGAATCCGACTGTTTAATTAAAACAAAGCATTGCGATGGTCCCTGCGGATGCTAACGCAATGTGATTTCTGCCCAGTGCTCTGAATGTCAAAGTGAAGAAATTCAACCAAGCGCGGGTAAACGGCGGGAGTAACTATGACTCTCTTAAGGTAGCCAAATGCCTCGTCATCTAATTAGTGACGCGCATGAATGGATTAACGAGATTCCCACTGTCCCTGTCTACTATCCAGCGAAACCACAGCCAAGGGAACGGGCTTGGCGGAATCAGCGGGGAAAGAAGACCCTGTTGAGCTTGACTCTAGTCCGACTTTGTGAAATGACTTGAGAGGTGTAGGATAAGTGGGAGCCGGAAACGGTGACAGTGAAATACCACTACTTTTAACGTTATTTTACTTATTCCGTGAATCGGAGGCGGGGCAATGCCCCTCTTTTTGGACCCAAGGTCGCTTCGCGGCCGATCCGGGCGGAAGACATTGTCAGGTGGGGAGTTTGGCTGGGGCGGCACATCTGTTAAAAGATAACGCAGGTGTCCTAAGATGAGCTCAACGAGAACAGAAATCTCGTGTGGAACAAAAGGGTAAAAGCTCGTTTGATTCTGATTTCCAGTACGAATACGAACCGTGAAAGCGTGGCCTATCGATCCTTTAGACCTTCGGAATTTGAAGCTAGAGGTGTCAGAAAAGTTACCACAGGGATAACTGGCTTGTGGCAGCCAAGCGTTCATAGCGACGTTGCTTTTTGATCCTTCGATGTCGGCTCTTCCTATCATTGTGAAGCAGAATTCACCAAGTGTTGGATTGTTCACCCACCAATAGGGAACGTGAGCTGGGTTTAGACCGTCGTGAGACAGGTTAGTTTTACCCTACTGATGACGGTGTCGCAATGGTAATTCAACCTAGTACGAGAGGAACCGTTGATTCGCACAATTGGTCATCGCGCTTGGTTGAAAAGCCAGTGGCGCGAAGCTACCGTGCGCTGGATTATGACTGAACGCCTCTAAGTCAGAATCCGGGCCAGAAGCGACGCATGCGCCTGCCGCTCGTTTGCCGACCCTCAGTAGGGGCCATCCGGCCCCCAAAGGCACGTGTCGTTGGCCAAGCCCTCGTGGCAGACAAGCTGCGTGGGCCGCCTTGAAGTATAATTCCCACCGGGCGGCGGGCAGAATCCTTTGCAGACGACTTAAATACGCGACGGGGTATTGTAAGTGGCAGAGTGGCCTTGCTGCCACGATCCACTGAGATTCAGCCCTTTGTCGCTCCGATTCGTCCCTCCCCCACAGCGTCTAACTCTATTCTTTGTAAAATTTCAAACAGAGGTTCATGCTCTCGTATCTGCAAGGCGATGGAAAACCAAAATGAAAACCAATGTGCCACACATTGCACGAGTTATGAAAAATATCAAGCG-ACACAGTTGTCACAACGCCGCCTGC-------CACGAACCCAAGGCATGCATGCGCTGTCTCACCAGCACAAATCGAGGCTAGGACCGGCAGCGAGCATGCATGCCAGGGGCAGTGCATGCGCAAGCGATGCTTCGCAAGGCAGGCCTGCACACGGCGCGCCTGCCTCATCGGTGAAATCCCACCCCGCCAACCCGAGGTTAAGCAAGGCGTGCCACGGGCCCCTTGGCAAACAC--CGTCTTATGTTTGCATTGCCTAGCTGTGCTGCCTAGGCGATGCCAAGGCATGCATGACCGTGCCGCCTGCAACGGCGAGGCTAAGGCATGCGTGCAAGCGC-ACATGGAGCCGGAAGCCATGCACGCAACGCTCTCGGCTGGGCCAGCGAGGCTAAGGCATGCGTGCAAGCGCACACGCGGCCTGCACCAGCTCTGTAACAATCGATGCCAAGCCATGCGTGCTGGCTGCCTCAGCTATGCCGGCCAGGCTAATGCATGGGTCCAAGCGCACATGCGTCCTGCACCAGCTCCGGAAC---------------------------------------------------------------------------------------------------------------------------------------------------------------------------------------------------------------------------------------------------------------------------------------------------------------------------------------------------------------------------------------------------------------------------------------------------------------------------------------------------------------------------------------------------------------------------------------------------

Euphorbia syncameronii TTTTTTCAAGTTTTCCGGGCCTTCCTCAAAATGGAAAAAGATATCGCCCATGTCCGAAAAGTCCCGGTATTACATGTAAATGTCCCTTTGAAAATGTTCGAACGGTGGTCGAAATTTCAACAAAAAGTGAAGGCTTTACATTTTTTTTTCCATTTTTTCCGAAACCCTTAAATATGGAAAAAATAACCCCCATGTCCGGAAAGCCCCGGTATTACATGTAAATGTCCCCTAAATAATGCTCGAACCGTGGTAAAAATTTCAGCTAAAAATGAAGGCTTTACATATTTTTTGCCATTTTCCCCGTAATTCCTAAAAATGGAAAAAATACCCCCGTCGGGCGGATTGGCCCGATATTACACGTCAATTTCCCCCAAATCATGCTCGAACTGGGGTCGGAATTTCGGCAAAAAAGGAAGGCTCTGCATATTTTTTTCCATTTTTCCCGGAATTCTTAAAAATGGGAAAAATATCCCCGACGTCCGGAAACTCCCGATATCAGGTGTAAATGTCCCTCAAATCATGCTCGAACCGTGGTAAAAATTTCAGCGAAAAATGAAGGCGCTGCATATTTTTTTCCATTTTTCCAGTGCCCGGAATTCACAAAAATGGAAAAAATAGCCCCGTTGTCCGGGAAGTCCCGGGATTGCATGTAAATTTCCCTTAAATCATGCTCGAACTATGGTAAAAATTTCAGAAAAAATCTCCAAGTATAGAGCAGTTTTTGGGGGGGTGTGGCTCCTGGAACAAATCGATGTCCGTTCCTCCCAGAGCTGGATTTGTCATAAGAAATACTATAGGGGCTGCGCCAGCTCTCAACCTTGGCCCGCACTGGGCTGCGGGCCCGTGTTGGGTGCCTCCCGCATGCTCTCCCGCATTCAAGCGCATCACTCGGTCTCCCAGAAAGTCCCGACCCGCCGCCCCACCGAACGGAAGAATATCGTGGAAAAGCTAAGCCCAAAAGCACCACACGCGCGCGCGCGACGGCGCGGCGCGTTCGCGATTTAGGCGATTTAGGCACTTGGCACTTAGCACGTAGCACGAAGCGCGAGGCGCGAGGCCAATCGGCGGAACATAGCAGAAAATATCCCGCAAAATTTATAAACGAGTAACGACACGGCATGAAAAAGAAGGTAGCTAGCAAAAATTTAAAAAGAAAGAAAGGAACATGGAGGCCAACCCGAAGTTCAGGCAAGAGTGCAGGGCGGCCTGGCCCGCCTTCGTGTGCGGGAGCAGGCGGGACAGCGTGCAGCGCTGTCCCGCATTCCTGCCTGTGCCTGGCGAGGCTAGGCAGGACGGCCGGCGTGCTGGCCCGCCCGCGTGTGCAGGAGGGCCTGCCAGTGGG-------------------AGGGCTGGCAAGCTGCCCTGGCTCGCCTGAGTGCATGGCGGGGGAGGGCAAGCTGGCCAGCACGCTGCCCTGCCTGGCTCGCCAGCCAGCACCGCGCAGGCAGGCCGCCAACCAGAGGTCAGCATAGGCATGCCAGCACGCCCAGCCGAGCTCTGCCGGCGGTGCTCACCCCGTCCGCGCAAAGGAGGCTAGGTCAGCGTGCTGTCTCGCCTGGCCGAGGCTCAGAATGGCTTCGCTGGGCCCGATCCAAATTTCGTTTGCAAAACCTTCGCACAGATAATAAGTTTGCCCGCACCATATTTTTAATACTGACTTTACCCATTTATTAGGAATTCTGTACCGAGTTTGGTATGCATTTGTTGAGTTTATATATTTTTTTGTATTTTTACGATTCTCGGACTTCGTAAATATGGAAAAAATACCTCCCGTGGGTAAAAGGTTACAATATTATATGGAAAAATCCCTTAATTCATGTTCTATCTACTGTCAAAATTTCAGGAAAAAACTCGAAGGTTTGACCCGTTTTTAAGGGGGTGTGGCTCCTGGAACAAATCGATGTCTGTTCCTCCCAGAGCTGGAAATGCTATAAGGACTACTATAGGGGGGTACACCTGCTCTCAACCCAGGCCACCACTGGGGCTGCGGGCCCGTGTTGGGTGCTTGCCGCATGCTCACCCCCTATCATGCGGGGAATTCAGCCACTAAGAACGTCCCGACCCGCCGCCCCTCCTCCGGCCGCCGGCCGCCGCCCCGCGCGGTGGTCGGAAAATCCAAAATTCTTAGAACGCTGAATTCCGCACCCCGAGAGCCCCTTTTCCCCCTGCCATTGTCGGAACTCGTTCGAATTGGGGGTAAAGTGGGTTTTCGGAGGCAACAACGTGCCCCGCGGCGCTGTCCCGATCGCTAGTGTGTGCCTACTCGTAGTTTTGTCTTGCATCGGATAGCTGATCGAGCTGCTCTCAGTGTGTATGTTGCTCGACTTCAAGCTGCTTTGGAAGCACGCCTAGAGTGAGGGGTAGCCGCTTGCTGCGCACGGTATCGGACGAGGGAAAAAGGAATTTCGGAAGAAAACGTTTCCGTGCCGTGCGCGATGTTGCTACATCCGTAGTTGCTTGCTGCGTGCGGTGTGGGGCGATGTAAAAAATGGAATTTGAAAATAAATTGTTTTCACGGAGTGCGCTTCGGCGTTGCCCCGATCGATAGTGCGTGTTGCTCGGTGGATTTGTTTGGCATTGGATAGCCGATCGAGTTGCTCTCGGTGTGAAGGTGCTGGACGTTGGTCCACTCGGCAGCCCGCCTTGAGTGAGGGGCAGTTGGTTGATGCGTGCGTTGCCAAGTGGCGAAAAAATGGATTTCACTAAAAATGTCTTTCTTGCTGTGCGCTCAGGCGCCGTCCCGATCGGTAGTGTGCGTTATGTCCGGTGGTTCTGTTTGGCATCTGATAGCTGATCGAGTCGCTCTCAGTGTGTAAGGTGCTGGGTTTTGGACTATTTGGCTTGCCTGCCTATCGTGAGGAGCGGTTGCTTGGTGCGTACAGTTCCGGACGGCGAAAAATTGAATTCCAATAAGATATTATTTTCTCAACACGTGCTACTTTATCATGCGGTAAGGAATGTTCTCTCGCACACAGCGGTTCGGGCGATGTCTCTACTCGACGTTTCGGCACTGCTTGATTCGTTCTCGAAGACAGCAGTGCAGTTCGGGGGGTGGGGATGTTGCTCAATATACGCGGCGGTGCATGAGTGGTAAATAGGCCATTGGGGTTGGCAGGCTCTGTGCTAGCGCATCGAACTGTCGTACCTTGAGGCCACTCAGTGGTGTCCCGGAGGCGTATTGCTATGTCGGGCGGGGATGGTTTCTGTGTTGCATACCCGCGCAGTGGAATGGAATTTTGTTGCCAAGAAACATTCGTCCCGTGCCCTTTTAGGGGCGTCGGATGAACCATGCAGCAGCTCTCGTGTGCCGGGCATGCCTTTATGGCTTCTCTGGCACATGTGAAGGTGCTCGTGCTCTCGGATGCGGAATGCTTTTGCGAGAGGAGGGATTGAGTTTCCTTTATGTGTTCTCGCTGTCCCTACATAAGAACCACCGTCCTTTCCGCACAGTGGCCTTGGTTGCTGCGGTGTACTATGTCTGCTTGCGGGTTAGGACGGCATGGAGGAATGCTACCTGGTTGATCCTGCCAGTAGTCATATGCTTGTCTCAAAGATTAAGCCATGCATGTGTAAGTATGAACTAATTCAGACTGTGAAACTGCGAATGGCTCATTAAATCAGTTATAGTTTGTTTGATGGTACCTGCTACTCGGATAACCGTAGTAATTCTAGAGCTAATACGTGCAACAAACCCCGACTTCTGGAAGGGATGCATTTATTAGATAAAAGGTCGACGCGGGCTCTGCCCGTTGCTCTGATGATTCATGATAACTCGACGGATCGCACGGCCATCGTGCTGGCGACGCATCATTCAAATTTCTGCCCTATCAACTTTCGATGGTAGGATAGAGGCCTACCATGGTGGTGACGGGTGACGGAGAATTAGGGTTCGATTCCGGAGAGGGAGCCTGAGAAACGGCTACCACATCCAAGGAAGGCAGCAGGCGCGCAAATTACCCAATCCTGACACGGGGAGGTAGTGACAATAAATAACAATACCGGGCTCTTCGAGTCTGGTAATTGGAATGAGTACAATCTAAATCCCTTAACGAGGATCCATTGGAGGGCAAGTCTGGTGCCAGCAGCCGCGGTAATTCCAGCTCCAATAGCGTATATTTAAGTTGTTGCAGTTAAAAAGCTCGTAGTTGGACCTTGGGTTGGGTCGACCGGTCCGCCTTACGGTGTGCACCTGTCGGCTCGTCCCTTCTGCCGGCGATGCGCTCCTGGCCTTAACTGGCCGGGTCGTGCCTCCGGCGCTGTTACTTTGAAGAAATTAGAGTGCTCAAAGCAAGCCTACGCTCTGTATACATTAGCATGGGATAACATCATAGGATTTCGGTCCTATTCTGTTGGCCTTCGGGATCGGAGTAATGATTAACAGGGACAGTCGGGGGCATTCGTATTTCATAGTCAGAGGTGAAATTCTTGGATTTATGAAAGACGAACAACTGCGAAAGCATTTGCCAAGGATGTTTTCATTAATCAAGAACGAAAGTTGGGGGCTCGAAGACGATCAGATACCGTCCTAGTCTCAACCATAAACGATGCCGACCAGGGATCGGCGGATGTTGCTTTTAGGACTCCGCCGGCACCTTATGAGAAATCAAAGTCTTTGGGTTCCGGGGGGAGTATGGTCGCAAGGCTGAAACTTAAAGGAATTGACGGAAGGGCACCACCAGGAGTGGAGCCTGCGGCTTAATTTGACTCAACACGGGGAAACTTACCAGGTCCAGACATAGTAAGGATTGACAGACTGAGAGCTCTTTCTTGATTCTATGGGTGGTGGTGCATGGCCGTTCTTAGTTGGTGGAGCGATTTGTCTGGTTAATTCCGTTAACGAACGAGACCTCAGCCTGCTAACTAGCTATGCGGAGGTATCCCTCCGCGGCCAGCTTCTTAGAGGGACTATGGCCTTCTAGGCCAAGGAAGTTTGAGGCAATAACAGGTCTGTGATGCCCTTAGATGTTCTGGGCCGCACGCGCGCTACACTGATGTATTCAACGAGTCTATAGCCTTGGCCGGCAGGCCCGGGTAATCTTTGAAATTTCATCGTGATGGGGATAGATCATTGCAATTGTTGGTCTTCAACGAGGAATTCCTAGTAAGCGCGAGTCATCAGCTCGCGTTGACTACGTCCCTGCCCTTTGTACACACCGCCCGTCGCTCCTACCGATTGAATGGTCCGGTGAAGTGTTCGGATCGCGGCGACGTGGGCGGTTCGCCGCCGGCGACGTCGCGAGAAGTCCACTGAACCTTATCATTTAGAGGAAGGAGAAGTCGTAACAAGGTTTCCGTAGGTGAACCTGCGGAAGGATCATTGTCGAAACCTGCCAGCAGAATGACCCGCGAACGTGTTTATAAATCGAGGGGCCGCTGCAGGATTCATCCAGCGATGGCACCTCACTAGGGCCCTGGCAGGGGATGCGGTGCGGTGGGATCCACCGTTCCCTGCGATCTCCTGTTTGCGGCCTATTAACAAAACCCCGGCGCCGTACGCGCCAAGGAATTGTAAAAAAAGATTGTGCAGCCCGATCGCACTGGCAACGGTGTGGCGGGTTTCACTGCGCTTTGAGAACCAAAATGACTCTCGGCAACGGATATCTCGGCTCTCGCATCGATGAAGAACGCAGCGAAATGCGATACTTGGTGTGAATTGCAGGATCCCGCGAACCATCGAGTCTTTGAACGCAAGTTGCGCCCGAAGCCTTTCGGCCGAGGGCACGTCTGCCTGGGTGTCACTCAAACGTCGCTCCAAACCCCTTCCATCGGGAGGGGTATGCGGGGCGGATGCTGGCCTCCCGTGTGCGTATCGCTCGCGGTTGGCCGAAATTCCTAGTCCTCGGCACGATGCCACGGAATCGGTGGTTGCAAGACCCTCGGAGAAAGCCTTGTGCGCTTGTAAGCCCTTTCGGACCATGAGACCCCAGAGCGTACCTAGCACTGCGACCCCAGGTCAGGCGGGATTACCCGCTGAGTTTAAGCATATCAATAAGCGGAGGAAAAGAAACTTACCAGGATTCCCCTAGTAACGGCGAGCGAACCGGGAAGAGCCCAGCTTGAGAATCGTGCGCCTGCGGCGTTCGAATTGTAGTCTGGAGAAGCGTCCTCAGCGGCGGACCGGGCCCAAGTCCCCTGGAAGGGGGCGCCGGAGAGGGTGAGAGCCCCGTCGTGCCCGGACCCTGTCGCACCACGAGGCGCTGTCTACGAGTCGGGTTGTTTGGGAATGCAGCCCAAATCGGGCGGTAAATTCCGTCCAAGGCTAAATATGGGCGAGAGACCGATAGCGAACAAGTACCGCGAGGGAAAGATGAAAAGGACTTTGAAAAGAGAGTCAAAGAGTGCTTGAAATTGTCGGGAGGGAAGCGGATGGGGGCCGGCGATGCGCCCCGGTCGGATGTGGAACGGTGACAAGCCGGTCCGCCGATCGGCTCGGGGCGCGGACCGATACGGATTGAGGCGGCGGCGTAAGCCCAGGAATTTGAAACGCCTGTGGAGATGCCGTCGCAGCAATCGTGGAAAGCAGCACGCGCCGTCTCGGCGTGCCTCGGCACCTGCGTGCTACTGGTGTCGGCCAGCGGGCTCCCCATTCGGCCCGTCTTGAAACACGGACCAAGGAGTCTGACATGTGTGCGAGTCAACGGGCGAGTAAACCCGTAAGGCGCAAGGAAGCTGACTGGCGGGATCCCCTAGAGGGTTGCACCGCCGACCGACCTTGATCTTCTGAGAAGGGTTCGAGTGAGAGCATGCCTGTCGGGACCCGAAAGATGGTGAACTATGCCTGAGCGGGGCGAAGCCAGAGGAAACTCTGGTGGAGGCCCGCAGCGATACTGACGTGCAAATCGTTCGTCTGACTTGGGTATAGGGGCGAAAGACTAATCGAACCGTCTAGTAGCTGGTTCCCTCCGAAGTTTCCCTCAGGATAGCTGGAGCTCGGAACGAGTTCTATCGGGTAAAGCCAATGATTAGAGGCATCGGGGGCGCAACGCCCTCGACCTATTCTCAAACTTTAAATAGGTAGGACGGCGCGGCTGCTTCGTTGAGCCGCGCCACGGAATCGAGAGCTCCAAGTGGGCCATTTTTGGTAAGCAGAACTGGCGATGCGGGATGAACCGGAAGCCGGGTTACGGTGCCCAACTGCGCGCTAACCTAGAACCCACAAAGGGTGTTGGTCGATTAAGACAGCAGGACGGTGGTCATGGAAGTCGAAATCCGCTAAGGAGTGTGTAACAACTCACCTGCCGAATCAACTAGCCCCGAAAATGGATGGCGCTTAAGCGCGCGACCTATACCCGGCCGTCGGGGCAAGAGCCAGGCCCCGATGAGTAGGAGGGCGCGGCGGTCGCTGCAAAACCCAGGGCGCGAGCCCGGGCGGAGCGGCCGTCGGTGCAGATCTTGGTGGTAGTAGCAAATATTCAAATGAGAACTTTGAAGGCCGAAGAGGGGAAAGGTTCCATGTGAACGGCACTTGCACATGGGTTAGTCGATCCTAAGAGACGGGGGAAGCCCGTCCGACAGCGCGTCCGCGCGCGAGCTTCGAAAGGGAATCGGGTTAAAATTCCCGAACCGGGACGCGGCGGCTGACGGCAACGTTAGGGAGTCCGGAGACGTCGGCGGGGGCCTCGGGAAGAGTTATCTTTTCTGTTTAACAGCCCGCCCACCCTGGAAACGACTCAGTCGGAGGTAGGGTCCAGCGGCTGGAAGAGCACCGCACGTCGCGCGGTGTCCGGTGCGCCCCCGGCGGCCCGTGAAAATCCGGAGGACCGAGTGCCATCCACGCCCGGTCGTACTCATAACCGCATCAGGTCTCCAAGGTGAACAGCCTCTGGTCGATGGAACAATGTAGGCAAGGGAAGTCGGCAAAATGGATCCGTAACCTCGGGAAAAGGATTGGCTCTGAGGGCTGGGCCCGGGGGTCCCAGTCCCGAACCCGTCGGCTGTCGGCGGACTGCTCGAGCTGCTCCCGCGGCAAGAGCGGGTCGCTGCGTGCCGGCCGGGGGACGGATTGGGAACGGCCCCTCTGGGGGCCTTCCCCGGGCGTCGAACAGTCGACTCAGAACTGGTACGGACAAGGGGAATCCGACTGTTTAATTAAAACAAAGCATTGCGATGGTCCCTGCGGATGCTAACGCAATGTGATTTCTGCCCAGTGCTCTGAATGTCAAAGTGAAGAAATTCAACCAAGCGCGGGTAAACGGCGGGAGTAACTATGACTCTCTTAAGGTAGCCAAATGCCTCGTCATCTAATTAGTGACGCGCATGAATGGATTAACGAGATTCCCACTGTCCCTGTCTACTATCCAGCGAAACCACAGCCAAGGGAACGGGCTTGGCAGAATCAGCGGGGAAAGAAGACCCTGTTGAGCTTGACTCTAGTCCGACTTTGTGAAATGACTTGAGAGGTGTAGTATAAGTGGGAGCCGGAAACGGCGATAGTGAAATACCACTACTTTTAACGTTATTTTACTTATTCCGTGAATCGGAGGCGGGGCATTGCCCCTCTTTTTGGACCAAAGGCCGCTTCGCGGTCGATCCGGGCGGAAGACATTGTCAGGTGGGGAGTTTGGCTGGGGCGGCACATCTGTTAAAAGATAACGCAGGTGTCCTAAGATGAGCTCAACGAGAACAGAAATCTCGTGTGGAACAAAAGGGTAAAAGCTCGTTTGATTCTGATTTCCAGTACGAATACGAACCGTGAAAGCGTGGCCTATCGATCCTTTAGACCTTCGGAATTTGAAGCTAGAGGTGTCAGAAAAGTTACCACAGGGATAACTGGCTTGTGGCAGCCAAGCGTTCATAGCGACGTTGCTTTTTGATCCTTCGATGTCGGCTCTTCCTATCATTGTGAAGCAGAATTCACCAAGTGTTGGATTGTTCACCCACCAATAGGGAACGTGAGCTGGGTTTAGACCGTCGTGAGACAGGTTAGTTTTACCCTACTGATGACGGTGTCGCGATGGTAATTCAACCTAGTACGAGAGGAACCGTTGATTCGCACAATTGGTCATCGCGCTTGGTTGAAAAGCCAGTGGCGCGAAGCTACCGTGCGCTGGATTATGACTGAACGCCTCTAAGTCAGAATCCGGGCCAGAAGCGATGCATGCGTCCGCCGCTCGTTTGCCGACCCTCAGTAGGGGCCATCCGGCCCCCAAAGGCACGTGTCGTTGGCTAAGCCCTCGCGGCAGACAAGCCGTGCGGGCAGCCTTGAAGTACAATTCCCACCGGGCGGCGGGCAGAATCCTTTGCAGACGACTTAAATACGCGACGGGGTATTGTAAGTGGCAGAGTGGCCTTGCTGCCACGATCCACTGAGATTCAGCCCTTTGTCGCTCCGATTCGTCCCTCCCTCCCAAGAAACTTTTTCCATCTCAATAGAATATCGAGCGGAGGCTGGGGTCTCGATTTCCGCTCGTAGAAAGAGGGCCAAGGAGAGAACCCCGGTATTGCATATGGCACGGGGGAAGCAAGAGATTAAGCACACCGCGGGGTGGAAAAAGCATCTGCGCACGGGCAGGTTAGAAAAACATACAAGCGATGCATCCCTTCAATGTCCCTCGGCGTTGACCGCTTGTTCATTTTTTTTTCCGGCTTGATGCGTACGTTTCGAACCACTCGTACACCAAGGCTGCCGCGGCCACGCGTGCCTCACCGGTCGATGCGGTCTACTGCCTTCACAGGCGAGGCAGGAGCAGCATCGAATTCCTGCCCCGGCACCACGCACGAGGCCGGCGGTGCCTACCTCTCGTGCGCAAGCGAGACCGATGCCAGCGCGGCTGACTGGCCTGAATAGGAGAGGCCAGTGCTAGTCCGCGCACGCCACATGCCCCTTCAAAGTTTCCCCGCATCGCTCGAGAATTTTACGGCAGGCCCGTTAAACATCTCCATCGGGAGAGAGAATTTGCATCTCCGCCCCAAGCGTCGATGCTAGTCCGCGCACGCCACATGCCCCTTCAAAGTTTCCCCGCACCGCTCGAGAATTTTATGGCAGACCCGTTAAATATCTCCATCGGGAGAGAAAATTTGCATCTCCGCCCCCAACCGTCGAGAAAAGATTAACACTTTTACCACCCCGAGGGTTGTACACACAGCCTCCTGAAACAAAGGGGCGGCAAAAACGCGTTTCGCCTCCAGTCCACGCTCGGTCTTTCGGAACGGGTTCAACGGCAGTTGATTTTATACCGAGTTGCCAAATTCTCACGAGGGCTCTGTATTCTTTTTCGTTTGTCAAGTTCCCGGCAGTCTTAAATACGAGAAAGGCCATCTCGAAGGTCCCGGCATTTTCACGAAGGTTCTAAATATATATTTTCCTTTTTTCAAGAGCTAGGACTTCTTAAAAATGGAAAAAATTATCCCCCGTGTCCGGAAAGTCCCGGTAATGCATGTAGATTTCCCTTGAACCATGCTGGTCAGAATTTCTGCAAAAAAAGGAAGGGTCTACGTATCTTCTGTCCATTTTTCCCGGACTCCCTAAAAATGGGAAAAATACCCCCCATGTTCGGAAAGTCCCGATATTACATGTAAATATCCCTTAAATCATACCCGAACTGTGGTCACAGTTTCAGC--AAAAAAAAAAATGAAGTCTTTACATAT

Euphorbia umbellata TTTTTTCAAGTTTTCCGGGCCTTCCTCAAAATGGAAAAAGATATCGCCCATGTCCGAAAAGTCCCGGTATTACATGTAAATGTCCCTTTGAAAATGTTCGAACGGTGGTCGAAATTTCAACAAAAAGTGAAGGCTTTACATTTTTTTTTCCATTTTTTCCGAAACCCTTAAATATGGAAAAAATAACCCCCATGTCCGGAAAGCCCCGGTATTACATGTAAATGTCCCCTAAATAATGCTCGAACCGTGGTAAAAATTTCAGCTAAAAATGAAGGCTTTACATATTTTTTGCCATTTCCCCCGTAATTCCTAAAAATGGAAAAAATGCCCCCGTCGGGCGGATTGGCCCGATATTACACGTCAATTTCCCCCAAATCATGCTCGAACTGGGGTCGGAATTTCGGCAAAAAAGGAAGGCTCTGCATATTTTTTTCCATTTTTCCCGGAATTCTTAAAAATGGGAAAAATATCGCCCACGTCCGGAAACTCCCGATATCAAGTGTAAATGTCCCTCAAATCATGCTCGAACCGTGGTAAAAATTTCAGCGAAAAATGAAGGCGTTGCATATTTTTTTCCATTTTTCCAGTGCCCGGAATTCACAAAAATGGAAAAAATAGCCCCGTTGTCCGGGAAGTCCCGGGATTGCATGTAAATTTCCCTTAAATCATGCTCGAACTATGGTAAAAATTTCAGAAAAAATCTCCAAGTATAGAGCAGTTTTTGGGGGGGTGTGGCTCCTGGAACAAATCGATGTCCGTTCCTCCCAGAGCTGGATTTGTCATAAGAAATACTATAGGGGCTGCGCCAGCTCTCAACCTTGGCCCGCACTGGGCTGCGGGCCCGTGGTGGGTGCCTCCCGCATGCTCTCCCGCATTCAAGCGCATCACTCGGTCTCCCAGAAAGTCCCGACCCGCCGCCCCACCGAACGGAAGAATATCGTGGAAAAGCTAAGCCCAAAAGCACCACACGCGCGCGCGCGACGGCGCGGCGCGTTCGCGATTTAGGCGATTTAGGCACTTGGCACTTAGCACGTAGCACGAAGCGCGAGGCGCGAGGCCAATCGGCGGAACGTAGCAGAAAATATCCCGCAAAATTTATAAACGAGTAACGACACGGCATGAAAAAGAAGGTAGCTAGCAAAAATTTTAAAAAAAAGAAAGGAACATGAAGGCCAACCCGAAGTTCAGGCAAGAGTGCAGGGCGGCCTGGCCCGCCTTCGTGTGCGGGAGCAGGCGGGACAGCGTGCAGCGCTGTCCCGCATTCCTGCCTGTGCCTGGCGAGGCTAGGCAGGACGGCCGGCGTGCTGGCCCGCCCGCGTGTGCAGGAGGGACTGCCAGTGGGAGGGCTGGCCCGCCTGGGCAGGGCTGGCAAGCTGCCCTGGCTCGCCTGAGTGCATGGCGGGGGAGGGCAAGCTGGCCAGCACGCAGCCCTGCCTGGCTCGCCAGCCAGCACCACGCAGGCAGGCCGCCAACCAGAGGTCAGCATAGGCATGCCAGCACGCCCAGCCGAGCTCTGCCGGCGGTGCTCACCCAGTCCGCGAAAAGGAGGCTAGGTCAGCGTGCTGTCTCGCCTGGCCGAGGCTCAGGATGGCTTCGCTGGGCCCGATCCAAATTTCGTTTGCAAAACCTTCGCACAGATAATAAGTTTTCCCGCACCATATTTTTTATACTGACTTTTACCATTTATTAGGAATTTTGTACCGAGTTCGTGATGCATTTGTTGAGTTTATATATTTTTTTGTATTTTTACGATTCTCGGACTTCGTAAATATGGAAAAAATACCTCCCGTGGGTAAAAGGTTACAATATTATATGGAAAAATCCCTTAATTCATGTTCTATCTACAGTCAAAATTTCAGGAAAAAACTCGAAGGTTTGACCGGTTTTTAAGGGGGTGTGGCTCCTGGAACAAATCGATGTCTGTTCCTCCCAGAGCTGGAAATGCTATAAGGACTACTATAGGGGGGTACACCTGCTCTCAACCCAGGCCACCACTGGGGCTGCGGGCCCGTGTAGGGTGCTTGCCGCATGCTCACCCCCTATCATGCGGGGAATTCAGCCACTAAGAACGTCCCGACCCGCCGCCCCTCCTCCGGCCGCCGGCCGCCGCCCCGCGCGGTGGCCGGAAAATCCAAAATTCTTAGAACGCTGAATTCCGCACCCCGAGAGCCCCTTTTCCCCCTGCCATTGTCGGAACTCGTTCGAATTGGGGGTAAAGTGGGTTTTCGGAGGCAACAACGTGCCCCGCGGCGCTGTCCCGATCGCTAGTGTGTGCCTACTCGTAGTTTTGTCTTGCATCGGATAGCTGATTGAGCTGCTCTCAGTGTGTATGGTGCTCGACTTCAAGCTGCTTTGGAAGCACGCCTAGAGTGAGGGGTAGCCGCTTGCTGCGCACGGTATCGGACGAGGGAAAAAGGAATTTCGGAAAAAAACGTTTCCGTGCCGTGCGCGATGTTGCTACATCCGTAGTTGCTTGCCGCGTGCGGTGTGGGGCGATGTAAAAAATGGAATTTGAAAATAAATTGTTTTCACGGAGTGCGCTTCGGCGTTGCCCCGATCGATAGTGCGTGTTGCTCGGTGGATTTGTTTGGCATTGGATAGCTGATCGAGTTGCTCTCGGTGTGAAGGTGCTGGACGTTGGTCCACTCGGCAGCCCGCCTTGAGTGAGGGGCAGTTGGTTGATGCGTGCGGTGCCAAGTGGCGAAAAAATGGATTTCACTAAAAATGTCTTTCTTGCTGTGCGCTCAGGCGCCGTCCCGATCGGTAGTGTGCGTTATGTCCGGTGGTTCTGTTTGGCATCTGATAGCTGATCGAGTCGCTCTCAGTGTGTAAGGTGCTGGGTTTTGGACTATTTGGCTTGCCTGCCTATCGTGAGGAGCGGTTGCTTGGTGCGTACAGTTCCGGACGGCGAAAAATTGAATTCCAATAAAATATTATTCTCTCAACACGTGCTACTTTATCATGCGGTAAGGAATGTTCTCTCGCACACAGCGGTTCGGGCGATGTCTCTACTCGACGTTTCGGCACTGCTTGATTCGTTCTCGAAGACAGCAGTGCAGTTCGGGGGGTGGGGATGTTGCTCAATATACGCGGCGGTGCATGAGTGGTAAATAGGCCATTGGGGTTGGCAGGCTCTGTGCTAGCGCATCGAACTGTCGTACCTTGAGGCCACTCAGTGGTGTCCCGGAGGCGTATTGCTATGTCGGGCGGGGATGGTTTCTGTGTTGCATACCCGCGCAGTGGAATGGAATTTTGTTGCCAAGAAACATTCGTCCCGTGCCCTTTTAGGGGCGTCGGATGAACCATGCAGCAGCTCTCGTGTGCCGGGCATGCCTTTTTGGCTTCTCTGGCACATGTGAAGGTGCTCGTGCTCTCGGATGCGGAATGCTTTTGCGAGAGGAGGGATTGAGTTTCCTTTATGTGTTCTCGCTGTCCCTACATAAGAACCACCGTCCTTTCCGCACAGTGGCCTTGGTTGCTGCGGTGTACTATGTCTGCTTGCGGGTTAGGACGGCATGGAGGAATGCTACCTGGTTGATCCTGCCAGTAGTCATATGCTTGTCTCAAAGATTAAGCCATGCATGTGTAAGTATGAACTAATTCAGACTGTGAAACTGCGAATGGCTCATTAAATCAGTTATAGTTTGTTTGATGGTACCTGCTACTCGGATAACCGTAGTAATTCTAGAGCTAATACGTGCAACAAACCCCGACTTCTGGAAGGGATGCATTTATTAGATAAAAGGTCGACGCGGGCTCTGCCCGTTGCTCTGATGATTCATGATAACTCGACGGATCGCACGGCCATCGTGCTGGCGACGCATCATTCAAATTTCTGCCCTATCAACTTTCGATGGTAGGATAGAGGCCTACCATGGTGGTGACGGGTGACGGAGAATTAGGGTTCGATTCCGGAGAGGGAGCCTGAGAAACGGCTACCACATCCAAGGAAGGCAGCAGGCGCGCAAATTACCCAATCCTGACACGGGGAGGTAGTGACAATAAATAACAATACCGGGCTCTTCGAGTCTGGTAATTGGAATGAGTACAATCTAAATCCCTTAACGAGGATCCATTGGAGGGCAAGTCTGGTGCCAGCAGCCGCGGTAATTCCAGCTCCAATAGCGTATATTTAAGTTGTTGCAGTTAAAAAGCTCGTAGTTGGACCTTGGGTTGGGTCGACCGGTCCGCCTTACGGTGTGCACCTGTCGGCTCGTCCCTTCTGCCGGCGATGCGCTCCTGGCCTTAACTGGCCGGGTCGTGCCTCCGGCGCTGTTACTTTGAAGAAATTAGAGTGCTCAAAGCAAGCCTACGCTCTGTATACATTAGCATGGGATAACATCATAGGATTTCGGTCCTATTCTGTTGGCCTTCGGGATCGGAGTAATGATTAACAGGGACAGTCGGGGGCATTCGTATTTCATAGTCAGAGGTGAAATTCTTGGATTTATGAAAGACGAACAACTGCGAAAGCATTTGCCAAGGATGTTTTCATTAATCAAGAACGAAAGTTGGGGGCTCGAAGACGATCAGATACCGTCCTAGTCTCAACCATAAACGATGCCGACCAGGGATCGGCGGATGTTGCTTTTAGGACTCCGCCGGCACCTTATGAGAAATCAAAGTCTTTGGGTTCCGGGGGGAGTATGGTCGCAAGGCTGAAACTTAAAGGAATTGACGGAAGGGCACCACCAGGAGTGGAGCCTGCGGCTTAATTTGACTCAACACGGGGAAACTTACCAGGTCCAGACATAGTAAGGATTGACAGACTGAGAGCTCTTTCTTGATTCTATGGGTGGTGGTGCATGGCCGTTCTTAGTTGGTGGAGCGATTTGTCTGGTTAATTCCGTTAACGAACGAGACCTCAGCCTGCTAACTAGCTATGCGGAGGTATCCCTCCGCGGCCAGCTTCTTAGAGGGACTATGGCCTTCTAGGCCAAGGAAGTTTGAGGCAATAACAGGTCTGTGATGCCCTTAGATGTTCTGGGCCGCACGCGCGCTACACTGATGTATTCAACGAGTCTATAGCCTTGGCCGACAGGCCCGGGTAATCTTTGAAATTTCATCGTGATGGGGATAGATCATTGCAATTGTTGGTCTTCAACGAGGAATTCCTAGTAAGCGCGAGTCATCAGCTCGCGTTGACTACGTCCCTGCCCTTTGTACACACCGCCCGTCGCTCCTACCGATTGAATGGTCCGGTGAAGTGTTCGGATCGCGGCGACGTGGGCGGTTCGCCGCCGGCGACGTCGCGAGAAGTCCACTGAACCTTATCATTTAGAGGAAGGAGAAGTCGTAACAAGGTTTCCGTAGGTGAACCTGCGGAAGGATCATTGTCGAAACCTGCCAGCAGAATGACCCGCGAACGTGTTTATAAATCGAGGGGCCGCTGCAGGATTCATCCAGCGATGGCACCTCACTAGGGCCCTGGCAGGGGATGCGGTGCGGTGGGATCCACCGTTCCCTGCGATCTCCTGTTTGCGGCCTATTAACAAAACCCCGGCGCCGTACGCGCCAAGGAATTGTAAAAAAAGATTGTGCAGCCCGATCGCACTGGCAACGGTGTGGCGGGTTTCACTGCGCTTTGAGAACCAAAATGACTCTCGGCAACGGATATCTCGGCTCTCGCATCGATGAAGAACGCAGCGAAATGCGATACTTGGTGTGAATTGCAGGATCCCGCGAACCATCGAGTCTTTGAACGCAAGTTGCGCCCGAAGCCTTTCGGCCGAGGGCACGTCTGCCTGGGTGTCACTCAAACGTCGCTCCAAACCCCTTCCATCGGGAGGGGTATGCGGGGCGGATGCTGGCCTCCCGTGTGCGTATCGCTCGCGGTTGGCCGAAATTCCTAGTCCTCGGCACGACGCCACGGAATCGGTGGTTGCAAGACCCTCGGAGAAAGCCTTGTGCGCTTGTAAGCCCTTTCGGACCATGAGACCCCAGAGCGTACCTAGCACTGCGACCCCAGGTCAGGCGGGATTACCCGCTGAGTTTAAGCATATCAATAAGCGGAGGAAAAGAAACTTACCAGGATTCCCCTAGTAACGGCGAGCGAACCGGGAAGAGCCCAGCTTGAGAATCGTGCGCCTGCGGCGTTCGAATTGTAGTCTGGAGAAGCGTCCTCAGCGGCGGACCGGGCCCAAGTCCCCTGGAAGGGGGCGCCGGAGAGGGTGAGAGCCCCGTCGTGCCCGGACCCTGTCGCACCACGAGGCGCTGTCTACGAGTCGGGTTGTTTGGGAATGCAGCCCAAATCGGGCGGTAAATTCCGTCCAAGGCTAAATATGGGCGAGAGACCGATAGCGAACAAGTACCGCGAGGGAAAGATGAAAAGGACTTTGAAAAGAGAGTCAAAGAGTGCTTGAAATTGTCGGGAGGGAAGCGGATGGGGGCCGGCGATGCGCCCCGGTCGGATGTGGAACGGTGACAAGCCGGTCCGCCGATCGGCTCGGGGCGCGGACCGATACGGATTGAGGCGGCGGCGTAAGCCCAGGAATTTGAAACGCCTGTGGAGATGCCGTCGCAGCAATCGTGGAAAGCAGCACGCGCCGTCTCGGCGTGCCTCGGCACCTGCGTGCTACTGGTGTCGGCCAGCGGGCTCCCCATTCGGCCCGTCTTGAAACACGGACCAAGGAGTCTGACATGTGTGCGAGTCAACGGGCGAGTAAACCCGTAAGGCGCAAGGAAGCTGACTGGCGGGATCCCCTAGAGGGTTGCACCGCCGACCGACCTTGATCTTCTGAGAAGGGTTCGAGTGAGAGCATGCCTGTCGGGACCCGAAAGATGGTGAACTATGCCTGAGCGGGGCGAAGCCAGAGGAAACTCTGGTGGAGGCCCGCAGCGATACTGACGTGCAAATCGTTCGTCTGACTTGGGTATAGGGGCGAAAGACTAATCGAACCGTCTAGTAGCTGGTTCCCTCCGAAGTTTCCCTCAGGATAGCTGGAGCTCGGAACGAGTTCTATCGGGTAAAGCCAATGATTAGAGGCATCGGGGGCGCAACGCCCTCGACCTATTCTCAAACTTTAAATAGGTAGGACGGCGCGGCTGCTTCGTTGAGCCGCGCCACGGAATCGAGAGCTCCAAGTGGGCCATTTTTGGTAAGCAGAACTGGCGATGCGGGATGAACCGGAAGCCGGGTTACGGTGCCCAACTGCGCGCTAACCTAGAACCCACAAAGGGTGTTGGTCGATTAAGACAGCAGGACGGTGGTCATGGAAGTCGAAATCCGCTAAGGAGTGTGTAACAACTCACCTGCCGAATCAACTAGCCCCGAAAATGGATGGCGCTTAAGCGCGCGACCTATACCCGGCCGTCGGGGCAAGAGCCAGGCCCCGATGAGTAGGAGGGCGCGGCGGTCGCTGCAAAACCCAGGGCGCGAGCCCGGGCGGAGCGGCCGTCGGTGCAGATCTTGGTGGTAGTAGCAAATATTCAAATGAGAACTTTGAAGGCCGAAGAGGGGAAAGGTTCCATGTGAACGGCACTTGCACATGGGTTAGTCGATCCTAAGAGACGGGGGAAGCCCGTCCGACAGCGCGTCCGCGCGCGAGCTTCGAAAGGGAATCGGGTTAAAATTCCCGAACCGGGACGCGGCGGCTGACGGCAACGTTAGGGAGTCCGGAGACGTCGGCGGGGGCCTCGGGAAGAGTTATCTTTTCTGTTTAACAGCCCGCCCACCCTGGAAACGACTCAGTCGGAGGTAGGGTCCAGCGGCTGGAAGAGCACCGCACGTCGCGCGGTGTCCGGTGCGCCCCCGGCGGCCCGTGAAAATCCGGAGGACCGAGTGCCATCCACGCCCGGTCGTACTCATAACCGCATCAGGTCTCCAAGGTGAACAGCCTCTGGTCGATGGAACAATGTAGGCAAGGGAAGTCGGCAAAATGGATCCGTAACCTCGGGAAAAGGATTGGCTCTGAGGGCTGGGCCCGGGGGTCCCAGTCCCGAACCCGTCGGCTGTCGGCGGACTGCTCGAGCTGCTCCCGCGGCAAGAGCGGGTCGCTGCGTGCCGGCCGGGGGACGGATTGGGAACGGCCCCTCTGGGGGCCTTCCCCGGGCGTCGAACAGTCGACTCAGAACTGGTACGGACAAGGGGAATCCGACTGTTTAATTAAAACAAAGCATTGCGATGGTCCCTGCGGATGCTAACGCAATGTGATTTCTGCCCAGTGCTCTGAATGTCAAAGTGAAGAAATTCAACCAAGCGCGGGTAAACGGCGGGAGTAACTATGACTCTCTTAAGGTAGCCAAATGCCTCGTCATCTAATTAGTGACGCGCATGAATGGATTAACGAGATTCCCACTGTCCCTGTCTACTATCCAGCGAAACCACAGCCAAGGGAACGGGCTTGGCAGAATCAGCGGGGAAAGAAGACCCTGTTGAGCTTGACTCTAGTCCGACTTTGTGAAATGACTTGAGAGGTGTAGTATAAGTGGGAGCCGGAAACGGCGATAGTGAAATACCACTACTTTTAACGTTATTTTACTTATTCCGTGAATCGGAGGCGGGGCATTGCCCCTCTTTTTGGACCAAAGGCCGCTTCGCGGTCGATCCGGGCGGAAGACATTGTCAGGTGGGGAGTTTGGCTGGGGCGGCACATCTGTTAAAAGATAACGCAGGTGTCCTAAGATGAGCTCAACGAGAACAGAAATCTCGTGTGGAACAAAAGGGTAAAAGCTCGTTTGATTCTGATTTCCAGTACGAATACGAACCGTGAAAGCGTGGCCTATCGATCCTTTAGACCTTCGGAATTTGAAGCTAGAGGTGTCAGAAAAGTTACCACAGGGATAACTGGCTTGTGGCAGCCAAGCGTTCATAGCGACGTTGCTTTTTGATCCTTCGATGTCGGCTCTTCCTATCATTGTGAAGCAGAATTCACCAAGTGTTGGATTGTTCACCCACCAATAGGGAACGTGAGCTGGGTTTAGACCGTCGTGAGACAGGTTAGTTTTACCCTACTGATGACGGTGTCGCGATGGTAATTCAACCTAGTACGAGAGGAACCGTTGATTCGCACAATTGGTCATCGCGCTTGGTTGAAAAGCCAGTGGCGCGAAGCTACCGTGCGCTGGATTATGACTGAACGCCTCTAAGTCAGAATCCGGGCCAGAAGCGATGCATGCGTCCGCCGCTCGTTTGCCGACCCTCAGTAGGGGCCATCCGGCCCCCAAAGGCACGTGTCGTTGGCTAAGCCCTCGCGGCAGACAAGCCGTGCGGGCAGCCTTGAAGTACAATTCCCACCGGGCGGCGGGCAGAATCCTTTGCAGACGACTTAAATACGCGACGGGGTATTGTAAGTGGCAGAGTGGCCTTGCTGCCACGATCCACTGAGATTCAGCCCTTTGTCGCTCCGATTCGTCCCTCCCTCCCAAGAAACTTTTTCCATCTCAAAAGAATATCGAGCGGAGGCTGGGGTCTCGATTTCCGCTCGTAGAAAGAGGGCCAAGGAGAGAACCCCGGTATTGCATATGGCACGGGGGAAGCAAGAGATTAAGCACACCGCGGGGTGGAAAAAGCATCTGCGCACGGGCAGGTTAGAAAAACATACAAGCGATGCATCCCTTCAATGTCCCTCGGCATTGACCGCTTGTTCATTTTTTTTTCCGGCTTAATGCGTACGTTTCGAACCACTCGTACACCAAGGCTGCCGCGGCCACGCGTGCCTCACCGGTCGATGCGGTCCACTGCCTTCACAGGCGAGGCAGGAGCAGCATCGAATTCCTGCCCCGGCACCACGCACGAGGCCGGCGGTGCCTACCTCTCGTGCGCAAGCGAGACCGATGCCAGCGCGGCTGACTGGCCTGAATAGGAGAGGCCAGTGCTAGTCCGCGCACGCCACATGCCCCTTCAAAGTTTCCCCGCATCGCTCGAGAATTTTACGGCAGGCCCGTTGAACATCTCCATCGGGAGAGAGAATTTGCATCTCCGCCCCAAGCGTCGATGCTAGTCCGCGCACGCCACATGCCCCTTCAAAGTTTCCCCGCACCGCTCGAGAATTTTATGGCAGACCCGTTAAATATCTCCATCGGGAGAGAAAATTTGCATCTCCGCCCCCAACCGTCGAGAAAAGATCAACACTTTTACCATCCCGAGGGTTGTACACACAGCCTCCTGAAACAAAGGGGCGGCAAAAACGCGTTTCGCCTCCAGTCCACGCTCGGTCTTTCGGAACGGGTTCAACGGCAGTTGATTTTATACCGAGTTGCCAAATTCTCACGAGGGCTCTGTATTCTTTTTCGTTTGTCAAGTTCCCGGCAGTCTTAAATACGAGAAAGGCCATCTCGAAGGTCCCGGCATTCTCACGAAGGTTCTAAATATATATTTTCCTTTTTTCAAGAGCTAGGACTTCTTAAAAATGGAAAAAATTATCCCCCGTGTCCGGAAAGTCCCGGTAATGCATGTAGATTTCCCTTGAACCATGCTGGTCAGAATTTCTGCAAAAAAATGAAGGGTCTACGTATCTTCTGTCCATTTTTCCCGGACTCCCTAAAAATGGGAAAAATACCCCCCATGTTCGGAAAGTCCCGATATTACATGTAAATATCCCTTAAATCACACCCGAACTGTGGTCACAATTTCCGCAAAAAAAAAAAAATGAAGTCTTTACATAT

;

END;
